# Impact of the COVID-19 pandemic on maternal mental health, early childhood development, and parental practices: a global scoping review

**DOI:** 10.1186/s12889-023-15003-4

**Published:** 2023-02-24

**Authors:** Ana Luiza Penna, Camila Machado de Aquino, Maria Suelly Nogueira Pinheiro, Rodrigo Leão Ferreira do Nascimento, Simone Farias-Antúnez, David Augusto Batista Sá Araújo, Carol Mita, Marcia Maria Tavares Machado, Marcia C. Castro

**Affiliations:** 1 Department of Global Health and Population, Harvard T. H. Chan School of Public Health, Boston, USA; 2 Department of Community Health, Federal University of Ceará, Fortaleza, Brazil; 3 Pontifical Catholic University, Rio de Janeiro, Brazil; 4 Department of Health Sciences, Federal University of Santa Catarina, Araranguá, Brazil; 5 Countway Library, Harvard Medical School, Boston, USA

**Keywords:** COVID-19, Maternal mental health, Early childhood development, Parenting practices, Review

## Abstract

**Background:**

In March 2020, the COVID-19 outbreak was declared a pandemic by the World Health Organization (WHO), generating stark economic and social repercussions that directly or indirectly affected families’ wellbeing and health status.

**Aims:**

This review aims at mapping the existing evidence on the impact of the COVID-19 pandemic on maternal mental health, early childhood development, and parental practices, worldwide, to identify evidence gaps and better inform future delivery of care and health policy measures.

**Methods:**

Following the protocol defined by PRISMA-ScR, this scoping review has searched for relevant studies published between January 2020 and June 2021, selecting evidence sources based on pre-established criteria. From a total of 2,308 articles, data were extracted from 537 publications from 35 countries on all three health domains.

**Results:**

The combined stressors brought forth by the pandemic have exerted a heavy burden on the mental health of mothers and the development of young children, partly mediated by its impact on parental practices.

**Conclusions:**

Despite remaining gaps, we have identified sufficient evidence pointing to an urgent need for more concerted global research efforts and rapid policy responses to timely address severe and pervasive negative impacts to the mental health of mothers and children at a key developmental stage.

**Supplementary Information:**

The online version contains supplementary material available at 10.1186/s12889-023-15003-4.

## Background

### Rationale

In March 2020, the COVID-19 outbreak was declared a pandemic by the World Health Organization (WHO). Following a complex interplay among social, cultural, and economic local contexts, regional-level infection rates developed differently, largely in response to administrative and public health measures. The economic and social impact of the pandemic on population wellbeing differed between and within countries, largely influenced by levels of employment insecurity, financial hardship, elevated morbidity and mortality burdens, requirement for prolonged social isolation measures, and overwhelmed health systems, contributing as significant stressor factors endured by families, globally.

Given the unprecedented nature of the COVID-19 pandemic, large evidence gaps exist on how the mental health of caregivers, as well as the early development of children, might have been impacted since the onset of the pandemic through various pathways—particularly through changes in key parental practices that directly affect children's wellbeing and the establishment of parental bonds. Prolonged adverse social conditions and family experiences, leading to toxic stress exposure, are particularly detrimental during the first years of children's lives, deeply affecting their present wellbeing as well as generating lifelong health consequences. The impact of the pandemic on these outcomes is, thus, deeply permeated by social determinants at play, which in turn delineates the unique cluster of social and biological factors interacting and jointly producing the impacts on health status and caregiving practices, captured by this review [227].

### Objectives

The main goal of this review was to address the question: what is the current evidence of the effects of the COVID-19 pandemic on maternal mental health, early child development (ECD), parenting practices, and the establishment of parental bonds, worldwide?

This study employs the nurturing care framework [228] as the basis upon which the three areas of interest are conceptualized and defined, i.e., early childhood development (ECD), parental practices, and maternal mental health. Early childhood development, therefore, is defined as the group of processes comprised within the first years of life, consisting of several interdependent domains, including sensory-motor, cognitive, and social-emotional [229–231]. Such rapid and critical developmental processes are sustained by nurturing care dispensed to young children, here defined as inter-related behaviours, attitudes, and knowledge regarding caregiving practices; stimulation; responsiveness, and safety [228]. Parental practices, therefore, are conceptualized in our review as the group of caregiving practices that promote a stable environment sensitive to children’s health needs, offer opportunities for early learning, and provide security, support, and adequate stimulation [228]. Accordingly, maternal mental health is here defined as the wellbeing and mental health status of mothers, as primary caregivers to young children, and its role in fostering or hindering their capacity to adequately provide nurturing care. Under such assumptions, parental practices are hypothesized to impact ECD both directly, when primarily performed by either mothers or other caregivers, and indirectly, when its impact is moderated by maternal mental health [233–235].

### Relevance

Defining the evidence gap around the possible effects of the pandemic on the mental health of mothers and on the development of children to better inform future delivery of care and health policy measures.

### Materials and methods

We followed the protocol defined by the Preferred Reporting Items for Systematic Reviews extension for Scoping Reviews (PRISMA-ScR) [232]. This review was not registered in PROSPERO since the platform does not accept the registration of scoping reviews.

Importantly, scoping reviews differ from systematic reviews in design, purpose, and structure. Following this study’s goal of mapping existing evidence on the field of interest, a scoping review has been deemed a more appropriate choice. The PRISMA-ScR defines as potential goals of scoping reviews the following: examining the extent, range, and nature of the evidence on a topic or question; summarizing findings from a body of knowledge that is heterogeneous in methods or discipline; or identifying gaps in the literature to aid the planning and commissioning of future research, including systematic reviews [232]. Conversely, systematic reviews are the appropriate methodology for addressing questions that aim at informing clinical decision-making, such as determining the feasibility, appropriateness, meaningfulness, or effectiveness of a particular intervention, potentially employing risk-of-bias assessment and meta-analysis [232, 236].

### Eligibility Criteria

Publications were eligible for inclusion if they contributed with scientific evidence regarding at least one of our three categories in relation to the COVID-19 pandemic, i.e., maternal mental health, early childhood development, and parental practices when concerning our population of interest, that is, mothers and/or caregivers, as well as children from 0–10 years old, worldwide. No age delimitation was defined for mothers and/or caregivers, and no specification was made for children and caregivers’ ethnicity and socioeconomic status. The lack of delimitations reflected the review aim of encompassing existing evidence regarding a broad range of populations, to better map gaps in the field. Peer-reviewed publications were included if they were published between January 1st, 2020, and June 9th, 2021. No language constraints were applied. Publications were required to comply with the following criteria:

1. Empirical studies that: (i) included our specified population of interest, and (ii) collected data and performed quantitative or qualitative analysis concerning at least one of the three outcome categories of our review.
2. Case reports describing health outcomes of neonates born to COVID-19 positive mothers.
3. Systematic reviews that: (i) included at least one of our outcome categories of interest, and (ii) presented detailed replicable search and selection methodology.

Given the unprecedented nature of the COVID-19 pandemic, case reports based on clinical records were included to assess initial documentation of its potential impact on early childhood development and the extent to which hypotheses raised by such reports were addressed by empirical research studies. Finally, rigorous systematic reviews aided our assessment of the evidence gap concerning our research question.

Regarding the age delimitation for studies concerning early childhood development, the choice for including children up to 10 years old reflects the goal of the scoping review to exhaustively identify relevant evidence concerning the outcomes of interest. The establishment of such a goal incurs a limitation to exclude studies that included in their sample children whose age exceeded the cutoff of 5 years old, which traditionally defines early childhood. This limitation is justified by the impossibility to derive from the findings of such studies those that pertained only to children 0–5 years old, given that findings were not always stratified by age sub-range. The choice for employing flexibility in the target age range addressed the characteristics of the nascent state of the field, which struggled to capture the rapid changes in population health status during the pandemic, incurring added difficulties in sampling, enrollment, and data collection procedures, prioritizing access to available studies’ subjects over rigor on sampling criteria.

Finally, while narrowing children’s age range was found to be unfeasible, the study opted for delimitating the analysis of caregivers’ mental health to mothers only. While examining the changes in the mental health status of fathers and other caregivers is undoubtedly relevant to better understanding the impact of the pandemic on ECD, parental practices, and family health, such delimitation reflected the need for processing available evidence on the initially settled outcomes on a pace that accompanied the articles production volume, over the inclusion of an additional outcome category which can be addressed by future research.

### Information sources

A search strategy was constructed to retrieve studies addressing the following parameters about the COVID-19 pandemic: mental health of mothers and pregnant women; parenting; perinatal, infant, and childhood development. The following databases were searched: PubMed/Medline (National Library of Medicine, NCBI), Embase (Elsevier, Embase.com), PsycInfo (American Psychological Association), and CINAHL (CINAHL Plus with Full Text, Ebsco). Controlled vocabulary terms (i.e., MeSH, Emtree, APA Thesaurus of Psychological Index Terms, CINAHL subject headings) were included when available and appropriate. The search strategies were designed and executed by a librarian (Carol Mita) on December 8th, 2020. Subsequently, an updated search was conducted on June 9th, 2021, utilizing the same methods. The exact search terms used for each of the databases are provided in a supplementary document. The electronic database search was supplemented by searching the WHO Global Research on the COVID-19 database and scanning relevant reviews published within the pre-defined period.

### Selection of publications

Search results were imported to the Covidence reference manager platform (Covid Systematic Review Software, Veritas Health Innovation, Melbourne Australia), and duplicates were removed. Initial selection by title and abstract and subsequent full-text review of remaining articles was conducted blindly by four independent reviewers with expert knowledge of the research area, following pre-established selection criteria as presented in the Eligibility Criteria section. Each selected publication was then individually appraised by two reviewers blindly. Divergent votes of inclusion based on pre-established criteria were settled through discussion and consensus among the reviewer’s team.

Articles excluded from either abstract or full-text selection were categorized as such:

1. Empirical research, case reports, or systematic literature review with no inclusion of population of interest.
2. Literature review not specifying the methodology for selection.
3. Empirical research, case reports, or literature review with no inclusion of any of the outcome categories of interest.
4. Clinical or hospital guidelines.
5. Commentaries letters to the editors, or any other publication not complying with the categories of empirical research, case report, or systematic literature review.
6. Study protocol.

### Data charting

A data charting Microsoft Excel^@^ table was jointly developed by two reviewers to categorize the variables to extract from selected publications, concerning the three categories of interest of the review. The table was updated based on an iterative process as data extraction proceeded and new variables were deemed relevant. A separate table was created to categorize excluded publications. All charting tables can be found in the Supplementary material.

The selection of variables of empirical reports and systematic reviews followed an iterative process, which began by testing a simplified data charting form with 3 variables: outcome category, place, and date of study publication. This initial form was tested with a sample of 30 articles by two reviewers, independently, with the joint purpose of examining the adequacy of the 3 prespecified variables and identifying other key characteristics. After the initial testing, it was determined by consensus among the reviewers and the study team the following:

1. While the three prespecified variables were adequate for assessing empirical reports, registering a place of publication for systematic reviews was deemed less relevant, given the collaborative nature of such works, often involving international research centers. The country of publication was used as a proxy for the country of data collection within empirical studies, informing about countries’ capacity for undertaking scientific research during the pandemic, data that were later examined against countries’ level of income, informing the relation between economic development and potential for undertaking scientific research, regardless of its quality or relevance. It also allowed direct mapping of evidence gaps concerning less studied populations given the data collection procedures. Systematic reviews did not allow for such approximations.
2. While a formal risk-of-bias assessment lies within the goals of systematic rather than scoping reviews, the team concluded that it was necessary to register major study characteristics that could inform the internal validity of empirical studies, given the pace of publication and heightened interest of the scientific community for the field. Following this conclusion, variables concerning study design, sampling method, and utilization of validated instruments were included.

This evaluation process was repeated one more time with a larger sample of 60 articles, resulting in the addition of the variables concerning sample size and population. Throughout both piloting rounds, a third independent reviewer acted as an arbitrator when a lack of consensus occurred. During the data charting of included articles, 5 additional independent reviewers were included in the analysis team.

Similarly, data charting for evidence gaps was executed through an iterative process involving a team of 7 independent reviewers and a single arbitrator. During data charting reviewers were asked to register aspects of each paper that they found could be better developed or at least minimally addressed. After charting was concluded, reviewers gathered and presented their lists of such missing aspects, which were registered as single entries. Subsequently, the team jointly analyzed such entries to identify common themes within each outcome category. Lastly, themes were organized by domains following the conceptual framework that structured data charting of included articles. These data are presented as evidence gaps.

### Data items

Nine variables were extracted from the final sample of selected studies: (i) inclusion criteria, i.e., primary research studies, case reports, or systematic reviews, (ii) country of publication, (iii) date of publication, (iv) study design, e.g., cross-sectional, prospective cohort, case–control, (v) type of sampling method, e.g., convenience sampling, open-access online form, (vi) utilization of validated instruments, e.g., scales measuring presence and severity of depressive symptoms, such as Edinburgh Postnatal Depression Scale (EPDS), (vii) primary outcome variables followed by scale utilized for measurement, if any, e.g., postnatal depression (EPDS), (viii) sample size of both reference and control groups when present, and (ix) study population, e.g., pregnant women, postnatal women, mothers of children 5–9 years old. Except for variables (i), (iii), (vii), and (ix), all apply to empirical research studies or case reports, not to systematic reviews.

### Critical appraisal of publications

We have conducted a frequency assessment of three main characteristics that inform the strength of the evidence produced by the selected empirical research studies, concerning their design, sampling, and measurement. These data were extracted from the data charting table and were selected as a way of appraising the quality of evidence production within the context of heightened interest of the scientific community around COVID-19, reflected on the accelerated rate of publications as the pandemic unfolded. They especially help to inform the validity and reliability of evidence concerning etiologic questions around the research area. Furthermore, we conducted a detailed validity assessment of all included review papers according to the PRISMA 2020 Checklist, utilizing the PRISMA application (PRISMA Checklist). The checklist contains 42 items in total, which we have used to generate a quality score categorizing review publications' quality by score terciles: 0–14 indicates low quality, 15–28 medium quality, and 29–42 high quality. We only included findings from medium and high-quality reviews in our final analysis. Individual quality assessment reports can be found in the Supplementary material.

### Synthesis of results

We present summarized publications` results by (1) main relevant characteristics, e.g., type, date, and country of publication, and (2) the three main outcome categories they refer to, i.e., maternal mental health, ECD, and parental practices. Supplementary systematic review findings, identified by scanning the WHO website, are included in the results as well.

## Results

### Selection of sources of evidence

**Fig. 1:**
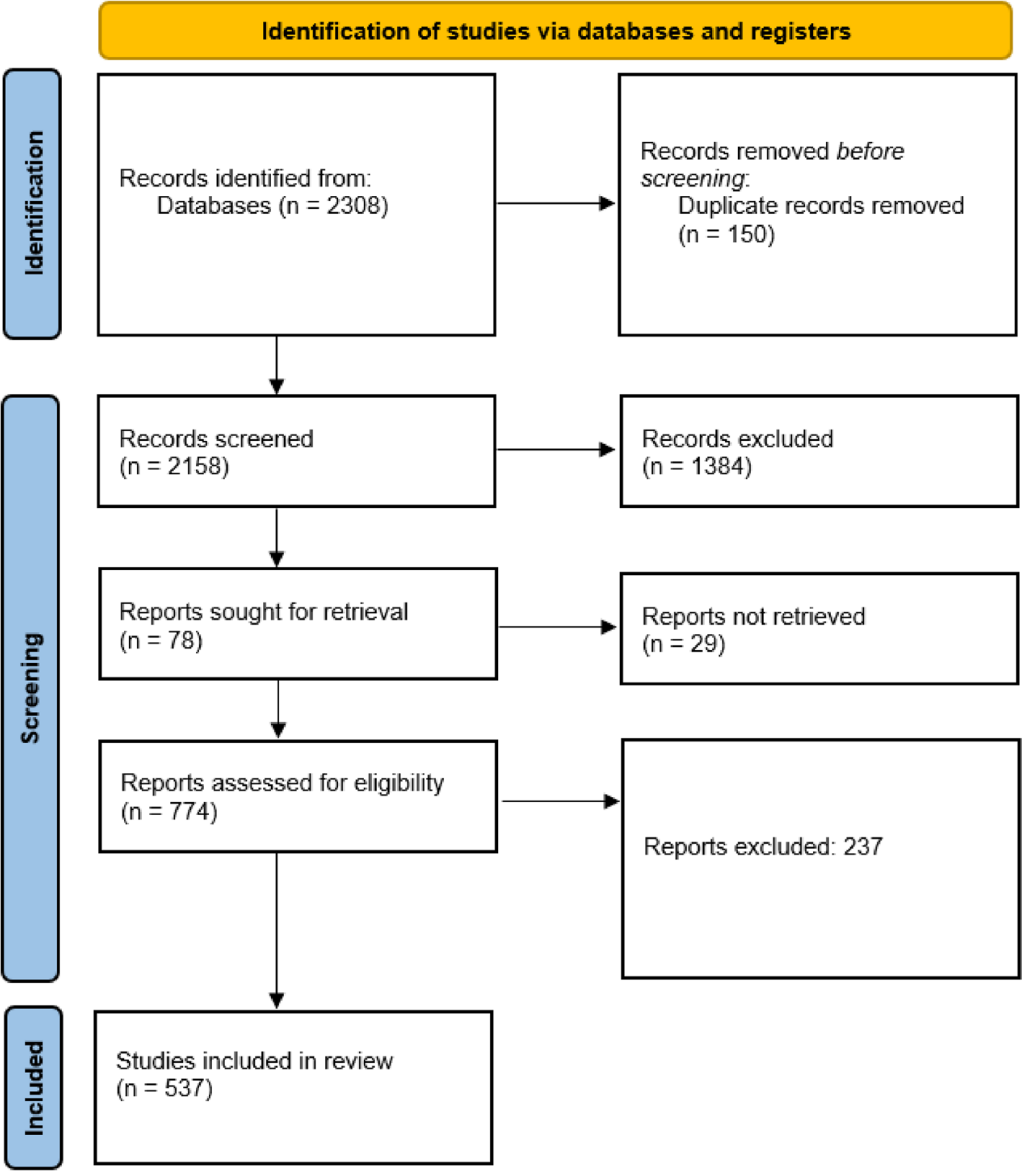
Flow diagram of the selection process showing identification, screening, and final inclusion of publications

Figure 1 shows the detailed publications selection flow diagram. Categories of exclusion are detailed in the Materials and Methods section, Selection of Publications sub-section.

### Characteristics and critical appraisal of evidence sources

**Fig. 2:**
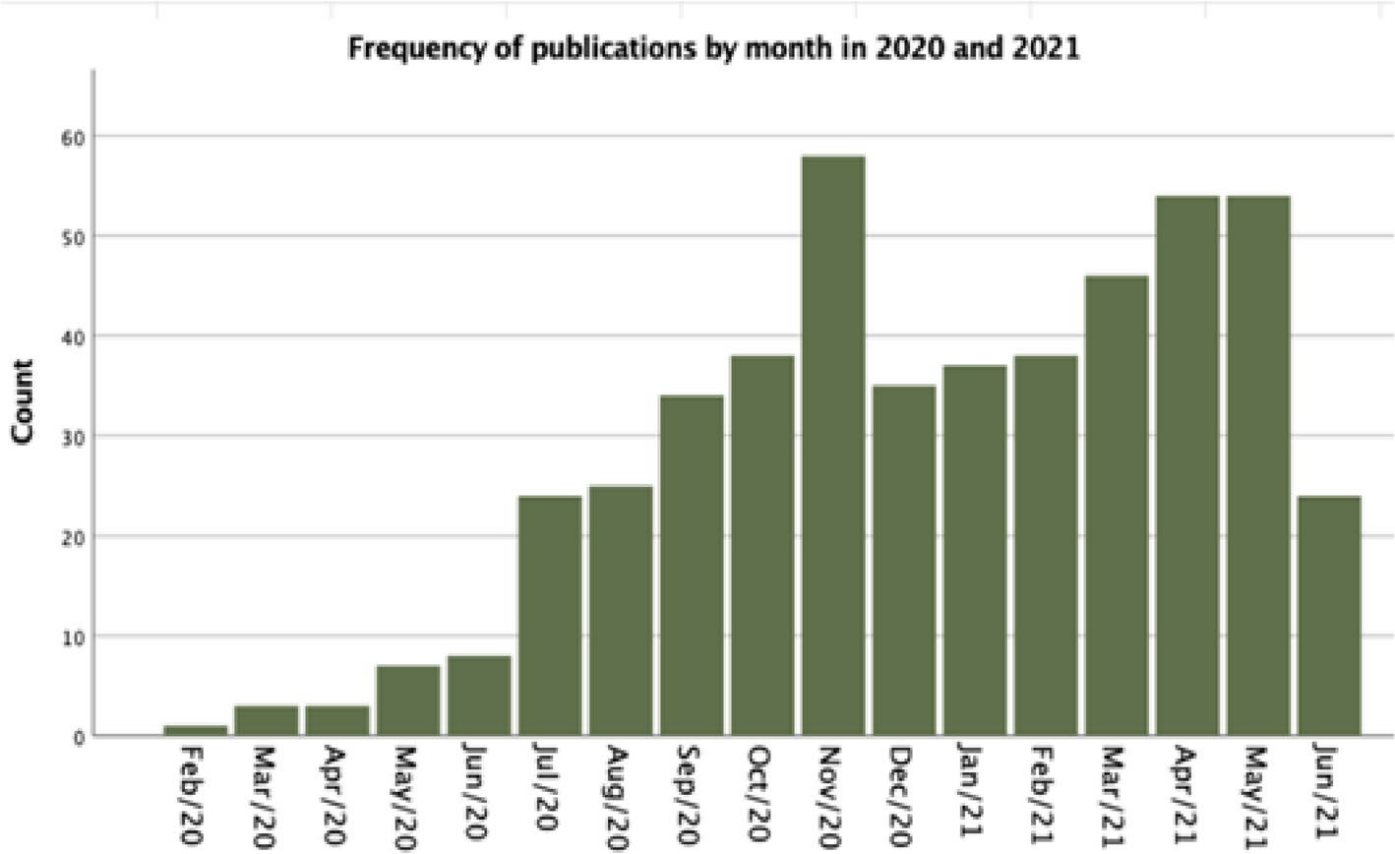
Distribution of publications by month, from January 1st, 2020, until June 9th, 2021

Individual characteristics of each included publication can be found in the Supplementary material. Figure 2 displays the distribution of eligible publications by month, from January 1st, 2020, until June 9th, 2021. The accelerated growth of the publication rate from July 2020 on is distinguishable, being especially pronounced in November 2020, as well as in April and May 2021.

Summarized characteristics of interest of the selected empirical research studies (*n* = 442) and case reports (*n* = 55) are:

1. Publication frequency by country-level income group, as categorized by the World Bank (2021): 63.19% of all selected publications were conducted in high-income countries, 28% in upper-middle-income countries, 5.23% in lower-middle, and 0.97% in low-income countries.
2. Countries with the highest number of publications: U.S. (21.2%), China (13.8%), Italy (8.9%), Turkey (5.5%), U.K (5.5%).
3. Main characteristics of empirical research studies: 67.4% employed a cross-sectional design, 34% utilized convenience sampling methods based on online open access links disseminated through social media platforms, and 52.2% used validated instruments of measurement concerning either maternal mental health, ECD, and/or parental practices.
4. The most frequently utilized validated instruments/scales were the Edinburgh Postnatal Depression scale – EPDS (11,9%), General Anxiety Disorder-7 – GAD (7%), Parenting Stress Index – PSI (4.2%), State-Trait Anxiety Inventory- STAI (4%), Strengths and Difficulties Questionnaire – SDQ (3,2%), and Zung Self-Rating Anxiety Scale – SAS (2,4%).
5. The most assessed variables concerning maternal mental health were stress (28,3%), depression (29,1%), and anxiety (30,1%).

### Results by categories and domains

Summarized findings concerning the three main outcome categories analysed by this review are presented next. Table 1 displays the most reported factors, categorized by domains, impacting maternal mental health, early child development and parenting practices.

**Table 1:** Most reported factors impacting maternal mental health, early child development and parenting practices, categorized by domains

Domains	Maternal Mental Health	Early Childhood Development	Parenting Practices
Behavioral	**Disrupted sleep patterns and/or decreased sleep duration**^**a**^ (Nicholas Carroll et al., 2020 [24, 25]; Cellini et al., 2021 [26]; Farewell et al., 2020 [60]; Kinser et al., 2021 [92]; Kracht et al., 2021 [95]; C. Li et al., 2021 [102]; Peltz et al., 2020 [152]; Romero-Gonzalez et al., 2020 [168, 169]; Shinomiya et al., 2021 [179]; Talbot et al., 2021 [188]; Xie et al., 2021 [206]; Xu et al., 2021 [207]; Zeng et al., 2020 [221]; Zhang et al., 2021 [222]; Çolak et al., 2021 [226]) **Disrupted dietary habits**^**a**^ (Campagnaro et al., 2020 [22]; N. Carroll et al., 2020 [24, 25]; Farewell et al., 2020 [60]; Ferrante et al., 2021b [66]; Kinser et al., 2021 [92]; Mahmoud et al., 2021 [109]; Malkawi et al., 2020 [110]; Philippe et al., 2021 [156]; Thompson & Bardone-Cone, 2021 [194]; S. D. Wang et al., 2021 [202]) **Excessive media exposure**^**a**^ (N. Carroll et al., 2020 [24, 25]; Kinser et al., 2021 [92]; Lemieux et al., 2020 [101]; Yue et al., 2020 [217]; Zhang et al., 2021 [222]; Zhou et al., 2021 [224]) **Regular physical activity**^**2**^ (Nicholas Carroll et al., 2020 [24, 25]; Farewell et al., 2020 [60]; Gildner et al., 2020 [74]; Kinser et al., 2021 [92]; Kracht et al., 2021 [95]; Limbers et al., 2020 [105]) **Psychological/emotional resources—resilience, perceived self-efficacy, self-kindness, psychological flexibility**^**b**^ (Boivin et al., 2020 [16]; Chasson et al., 2020 [29]; Daks et al., 2020 [44]; Farewell et al., 2020 [60]; Guo et al., 2021 [76]; Gur et al., 2020 [77]; Kinser et al., 2021 [92]; Marchetti, Fontanesi, Mazza, et al., 2020 [115, 116]; Mazza et al., 2020 [121]; Mo et al., 2021[125]; Nodoushan et al., 2020 [141]; Peltz et al., 2020 [152]; Taubman-Ben-Ari et al., 2020 [190, 191]; Valero-Moreno et al., 2021 [196]; Xue et al., 2021 [210, 211])	**Disrupted sleep patterns and/or decreased sleep duration**^**b**^ (Auðardóttir & Rúdólfsdóttir, 2020 [11]; N. Carroll et al., 2020 [24, 25]; Cellini et al., 2021 [21]; Dellagiulia et al., 2020 [48]; Horiuchi et al., 2020 [84]; Z. Liu et al., 2020 [108]; Nassar et al., 2021 [137]; Shinomiya et al., 2021 [179]; Zreik et al., 2020 [225]) **Disrupted dietary habits**^**b**^ (Ares et al., 2021 [9]; Auðardóttir & Rúdólfsdóttir, 2020 [11]; Nicholas Carroll et al., 2020 [24, 25]; Ferrante et al., 2021a [65]; Jansen et al., 2021 [87]; Mahmoud et al., 2021 [109]; Philippe et al., 2021 [156]; Sama et al., 2020 [174]; S. D. Wang et al., 2021 [202]) **Excessive media exposure**^**b**^ (Poulain et al., 2021 [158]; Yue et al., 2020 [217]) **Increased screen time**^**b**^ (N. Carroll et al., 2020 [24, 25]; de Sá et al., 2020 [46]; Horiuchi et al., 2020 [84]; W. Li et al., 2021 [103]; Ozturk Eyimaya & Yalçin Irmak, 2020 [148]; Sama et al., 2020 [174]) **Regular physical activity**^**a**^ (Nicholas Carroll et al., 2020 [24, 25]; de Sá et al., 2020 [46]; Horiuchi et al., 2020 [84]; W. Li et al., 2021 [103]; Moore et al., 2020; Sama et al., 2020 [127]) **Loss of playing time outdoors**^**b**^ (de Sá et al., 2020 [46]; Poulain et al., 2021 [158]; Sama et al., 2020 [174]) **Psychological/emotional resources- emotional regulation**^**a**^ (Spinelli, Lionetti, Setti, et al., 2020 [183, 184])	**Changes in feeding practices**^**c**^ (Farghaly et al., 2020 [61]; Jansen et al., 2021 [87]; Nguyen et al., 2021 [140]; Sánchez-Luna et al., 2021 [187]; Vazquez-Vazquez et al., 2021 [198]; S. D. Wang et al., 2021 [202]) **Breastfeeding**^**c**^ (DeYoung & Mangum, 2021 [49]; Ferrazzi et al., 2020 [67]; Litmanovitz et al., 2021 [106]; Oncel et al., 2020 [145]; Peng et al., 2020 [154]; Ronchi et al., 2021 [170]; Sabharwal et al., 2021 [172]; Zanardo et al., 2020 [220]) **Maternal-newborn separation days**^**b**^ (Ashini et al., 2021 [10]; Bender et al., 2020 [14]; Conti et al., 2021 [38]; Farhadi et al., 2021 [62]; Franchi et al., 2020 [71]; Litmanovitz et al., 2021 [106]; W. Liu et al., 2020 [107]; Scala et al., 2020 [175]; Wang et al., 2020 [201, 203])
Health and development		**Reading and math skills**^**b**^ (Bao et al., 2020; Quenzer-Alfred et al., 2021 [163]) **Immunization delays**^**b**^ (Alsuhaibani & Alaqeel, 2020 [6])	**Caregiving concerning children with neurodevelopmental disabilities and/or chronic illnesses**^**c**^ (Ademhan Tural et al., 2020 [3]; Ayas et al., 2020 [12]; Bentenuto et al., 2021 [15]; Brisca et al., 2021 [17]; Chan & Fung, 2021 [27]; Chen et al., 2020 [30]; Di Riso et al., 2021 [52]; Grumi et al., 2020 [75]; Mirlashari et al., 2020 [123]; Neece et al., 2020 [139]; Odeh et al., 2020 [143]; Scala et al., 2020 [175]) **Neonatal care**^**c**^ (Congdon et al., 2021; Sabharwal et al., 2021 [36]) **Immunization delays**^**a**^ (Alsuhaibani & Alaqeel, 2020 [6])
Relational	**Barriers to information on COVID-19 related health risks and/or inconsistent health related messages**^**b**^ (Ding et al., 2021 [54]; Farewell et al., 2020 [60]; Kahyaoglu Sut & Kucukkaya, 2020 [89]) **Fear of infection and vertical transmission**^**b**^ (Ahorsu et al., 2020 [4]; Chasson et al., 2020 [29]; Ding et al., 2021 [54]; Fan et al., 2021 [59]; Farewell et al., 2020 [60]; Gur et al., 2020 [77]; Kahyaoglu Sut & Kucukkaya, 2020 [89]; Mappa et al., 2020 [114]; Mo et al., 2021 [125]; Molgora & Accordini, 2020 [126]; Naurin et al., 2020 [138]; Rhodes et al., 2020 [165]; Thompson & Bardone-Cone, 2021 [194]; Yirmiya et al., 2021 [216]) **Fear of adverse birth outcomes**^**b**^ (Ahorsu et al., 2020 [4]; Fan et al., 2021 [59]; Gur et al., 2020 [77]; Kahyaoglu Sut & Kucukkaya, 2020 [89]; Kinser et al., 2021 [92]; Mappa et al., 2020 [114]; Molgora & Accordini, 2020 [126]; Naurin et al., 2020 [138]; Rhodes et al., 2020 [165]; Yirmiya et al., 2021 [216]) **Family conflict and intimate partner violence**^**b**^ (Chapman et al., 2021 [28]; Chmielewska et al., 2021 [32]; Daks et al., 2020 [44]; Guo et al., 2021 [76]; Hamadani et al., 2020 [80]; Muldoon et al., 2021 [134]; Naghizadeh et al., 2021 [136]; Sakalidis et al., 2021 [173]; Xie et al., 2021 [206]) **Lack of partner and/or family support**^**b**^ (Daks et al., 2020 [44]; Fan et al., 2021 [59]; Farewell et al., 2020 [60]; Guo et al., 2021 [76]; Kotlar et al., 2021 [93]; Manja et al., 2020 [113]; Marchetti, Fontanesi, Mazza, et al., 2020 [115, 116]; Mazza et al., 2020 [121]; Pınar Senkalfa et al., 2020 [157]; Xu, Wu, Levkoff, et al., 2020 [208, 209]) **Unbalanced and increased caregiving and housework responsibilities during lockdown**^**b**^ (Auðardóttir & Rúdólfsdóttir, 2020 [11]; N. Carroll et al., 2020 [24, 25]; Chmielewska et al., 2021 [32]; Costoya et al., 2021 [39]; Craig & Churchill, 2020 [40]; Del Boca et al., 2020 [47]; Ferns et al., 2021 [64]; Fosco et al., 2021 [70]; Gassman-Pines et al., 2020 [73]; Halley et al., 2021 [79]; Hjálmsdóttir & Bjarnadóttir, 2020 [83]; Malkawi et al., 2020 [110]; Marchetti, Fontanesi, Mazza, et al., 2020 [115, 116]; Miller et al., 2020 [122]; Mousavi, 2020 [130]; Pınar Senkalfa et al., 2020 [157]; Shafer et al., 2020 [177]; Shockley et al., 2020 [180]; S. D. Wang et al., 2021 [202]; Xue & McMunn, 2021 [210, 211]; Yamamura & Tsustsui, 2021 [212]; Yerkes et al., 2020 [214]; Yildirim & Eslen-Ziya, 2020 [215]; Zamarro & Prados, 2021 [219]) **Number of children in household**^**b**^ (Ding et al., 2021 [54]; Guo et al., 2021 [76]; Marchetti, Fontanesi, Mazza, et al., 2020 [115, 116]; Pınar Senkalfa et al., 2020 [157]; Yirmiya et al., 2021 [216]) **Caregiving responsibilities concerning children with neurodevelopmental disorders and/or chronic illnesses**^**c**^ (Chen et al., 2020 [30]; Faccioli et al., 2021 [58]; Grumi et al., 2020 [75]; Marchetti, Fontanesi, Mazza, et al., 2020 [115, 116]; Neece et al., 2020 [139]; Pınar Senkalfa et al., 2020 [157]; Ren et al., 2020; Rogers et al., 2021 [167]) **Household children's age**^**a**^ (Marchetti, Fontanesi, Mazza, et al., 2020 [115, 116]; Mazza et al., 2020 [121]; Pınar Senkalfa et al., 2020 [157]) **Family cohesion**^**a**^ (Daks et al., 2020 [44]; Fosco et al., 2021 [70]; Xie et al., 2021 [206])	**Caregivers’ mental health: elevated stress, depressive and/or anxiety symptoms**^**b**^ (Calvano et al., 2021 [20]; Cheng et al., 2021 [30]; DeYoung & Mangum, 2021 [49]; Gadermann et al., 2021 [72]; Lee et al., 2020 [98]; Patrick et al., 2020 [151]; Russell et al., 2020 [171]; Spinelli, Lionetti, Pastore, et al., 2020 [183, 184]) **Secondary caregiver mental health grandparents**^**c**^ (Wu et al., 2021[205]; Xu, Wu, Jedwab, et al., 2020 [208, 209]; Xu, Wu, Levkoff, et al., 2020 [208, 209]) **Family conflict / abuse / harsh parenting**^**a**^ (Brown et al., 2020 [18, 19]; Calvano et al., 2021 [20]; Chung et al., 2020 [34]; Connell & Strambler, 2021 [37]; Fosco et al., 2021 [70]; Kovler et al., 2020 [94]; Lawson et al., 2020 [96]; Lee et al., 2021 [98]; W. Li et al., 2021 [103]; Martins-Filho et al., 2020 [118]; Waller et al., 2021 [200]) **Frequent parents-child dialogues about COVID-19**^**a**^ (Choi et al., 2021 [33]; Cohodes et al., 2021 [35]; Tang et al., 2020 [189]) **Decreased social interaction/ isolation**^**b**^ (Farghaly et al., 2020 [61]; Lee et al., 2020 [98]; Z. Liu et al., 2020 [108]; Moore et al., 2020 [127]; Ozturk Eyimaya & Yalçin Irmak, 2020 [148]; Sama et al., 2020 [174])	**Maternal-infant attachment**^**c**^ (Farhadi et al., 2021 [62]; Fernandes et al., 2021 [63]; Liang et al., 2021 [104]; Mayopoulos et al., 2021 [120]; Oskovi-Kaplan et al., 2020 [146]; Peng et al., 2021 [153]; Steinberg et al., 2021 [185]; Suzuki, 2020 [186]; Taubman-Ben-Ari & Ben-Yaakov, 2020 [190, 191]) **Family dynamics / parenting styles**^**c**^ (Altena et al., 2020 [7]; Choi et al., 2021 [33]; Cohodes et al., 2021 [35]; Daks et al., 2020 [44]; Fernandes et al., 2021 [63]; Forbes et al., 2021 [69]; Fosco et al., 2021 [70]; Mahmoud et al., 2021 [109]; Marchetti, Fontanesi, Di Giandomenico, et al., 2020 [115, 116]; Romero et al., 2020 [168, 169]; Waller et al., 2021 [200]; Xu, Wu, Jedwab, et al., 2020 [208, 209]; Xu, Wu, Levkoff, et al., 2020 [208, 209]) **Family conflict / abuse / harsh parenting**^**a**^ (Brown et al., 2020 [18, 19]; Calvano et al., 2021 [20]; Chung et al., 2020 [34]; Connell & Strambler, 2021 [37]; Fosco et al., 2021 [70]; Kovler et al., 2020 [94]; Lawson et al., 2020 [96]; Lee et al., 2021 [98]; W. Li et al., 2021 [103]; Martins-Filho et al., 2020 [118]; Waller et al., 2021 [200]; Xu, Wu, Jedwab, et al., 2020 [208, 209]) **Frequent parents-child dialogues about COVID-19**^**a**^ (Choi et al., 2021 [33]; Cohodes et al., 2021 [35]; Tang et al., 2020 [189]) **Maternal unbalanced and increased caregiving and housework responsibilities during lockdown**^**b**^ (Auðardóttir & Rúdólfsdóttir, 2020 [11]; Nicholas Carroll et al., 2020 [24, 25]; Chmielewska et al., 2021 [32]; Costoya et al., 2021 [39]; Craig & Churchill, 2020 [40]; Del Boca et al., 2020 [47]; Ferns et al., 2021 [64]; Fosco et al., 2021 [70]; Gassman-Pines et al., 2020 [73]; Halley et al., 2021 [79]; Hjálmsdóttir & Bjarnadóttir, 2020 [83]; Malkawi et al., 2020 [110]; Marchetti, Fontanesi, Mazza, et al., 2020 [115, 116]; Miller et al., 2020 [122]; Mousavi, 2020 [130]; Pınar Senkalfa et al., 2020 [157]; Shafer et al., 2020 [177]; Shockley et al., 2020 [180]; S. D. Wang et al., 2021 [202]; Xue & McMunn, 2021 [210, 211]; Yamamura & Tsustsui, 2021 [212]; Yerkes et al., 2020 [214]; Yildirim & Eslen-Ziya, 2020 [215]; Zamarro & Prados, 2021 [219])
Demographic	**Gender inequality**^**b**^ (Costoya et al., 2021 [39]; Craig & Churchill, 2020 [40]; Del Boca et al., 2020 [47]; Ferns et al., 2021 [64]; Hjálmsdóttir & Bjarnadóttir, 2020 [83]; Marshall et al., 2020 [117]; Miller et al., 2020 [122]; Mousavi, 2020 [130]; Shafer et al., 2020 [177]; Shockley et al., 2020 [180]; Xue & McMunn, 2021 [210, 211]; Yamamura & Tsustsui, 2021 [212]; Yerkes et al., 2020 [214]; Yildirim & Eslen-Ziya, 2020 [215]) **Ethnicity- Black or Latinx**^**b**^ (Gildner et al., 2020 [74]; Gur et al., 2020 [77]; Quandt et al., 2020 [162])	**Ethnicity**^**c**^ (Quandt et al., 2020 [162])	
Economic	**Economic recession**^**b**^ (Ding et al., 2021 [54]; Silverman et al., 2020 [182]; Spinelli, Lionetti, Setti, et al., 2020 [183, 184]) **Financial hardship and/or strain**^**b**^ (N. Carroll et al., 2020 [24, 25]; Chung et al., 2020 [34]; Daulay, 2021 [45]; Dhiman et al., 2020 [50]; Gassman-Pines et al., 2020 [73]; Gildner et al., 2020 [74]; Halley et al., 2021 [79]; Hamadani et al., 2020 [80]; Khoury et al., 2021 [91]; Kinser et al., 2021 [92]; Mulale et al., 2021 [133]; Perzow et al., 2021; Sakalidis et al., 2021 [173]; Silverman et al., 2020 [182]; Thayer & Gildner, 2020 [193]; S. D. Wang et al., 2021 [202]; Wimberly et al., 2021 [204]) **Employment insecurity**^**b**^ (Gassman-Pines et al., 2020 [73]; Guo et al., 2021 [76]; Gur et al., 2020 [77]; Halley et al., 2021 [79]; Kinser et al., 2021 [92]; Moyer et al., 2020 [131]; Quandt et al., 2020 [162]; Valero-Moreno et al., 2021 [196]; Vasilevski et al., 2021 [197]; S. D. Wang et al., 2021 [202]; Wu et al., 2021 [205]) **Food insecurity**^**b**^ (N. Carroll et al., 2020 [24, 25]; Dumbre et al., 2020 [56]; Ferrante et al., 2021a [65]; Hamadani et al., 2020 [80]; Moyer et al., 2020 [131]; Patrick et al., 2020 [151]; Rocha et al., 2021 [166])	**Income inequality**^**b**^ (W. Li et al., 2021 [103]) **Financial hardship and/or strain**^**b**^ (Cheng et al., 2021 [30]; Chung et al., 2020 [34]; Dumbre et al., 2020 [56]; Fisher et al., 2021 [68]; Gassman-Pines et al., 2020 [73]; Lawson et al., 2020 [96]; Quandt et al., 2020 [162]) **Employment insecurity**^**b**^ (Lawson et al., 2020 [96]; Quandt et al., 2020 [162]) **Food insecurity**^**b**^ (Nicholas Carroll et al., 2020 [24, 25]; Nguyen et al., 2021 [140]; Patrick et al., 2020 [151]; Rocha et al., 2021 [166])	
Social and Cultural	**Isolation / social distancing measures/ lockdown**^**b**^ (Achterberg et al., 2021 [2]; Bender et al., 2020 [14]; Brown & Shenker, 2020 [18, 19]; Chan & Fung, 2021 [27]; Chapman et al., 2021 [28]; Dickerson et al., 2020 [53]; El-Osta et al., 2021 [57]; Farghaly et al., 2020 [61]; Hailemariam et al., 2021 [78]; Khoury et al., 2021 [91]; Mangiavacchi et al., 2021 [111]; Mizrak Sahin & Kabakci, 2020 [124]; Nomura et al., 2021 [142]; Overbeck et al., 2020 [147]; Protudjer et al., 2020 [161]; Ren et al., 2020 [30, 164]; Talbot et al., 2021 [188]; TG et al., 2021 [192]; Q. Wang et al., 2021 [125, 201]) **Access barriers to health services**^**b**^ (Cameron et al., 2020; Hailemariam et al., 2021 [78]; Masters et al., 2021 [119]; Moyer et al., 2020 [131]; TG et al., 2021 [192]; Vasilevski et al., 2021 [197]; Q. Wang et al., 2021 [125, 201]) **Lack of social support**^**b**^ (Chasson et al., 2020 [29]; Fan et al., 2021 [59]; Farewell et al., 2020 [60]; Gur et al., 2020 [77]; Kassaw & Pandey, 2020 [90]; Lebel et al., 2020 [97]; Marchetti, Fontanesi, Mazza, et al., 2020 [115, 116]; Rhodes et al., 2020 [165]; Taubman-Ben-Ari et al., 2020 [190, 191]; Vazquez-Vazquez et al., 2021 [198]; Zhang & Ma, 2020 [223]) **Maternal education level**^**c**^ (Aksoy Derya et al., 2020 [5]; Daulay, 2021 [45]; Gildner et al., 2020 [74]; Guo et al., 2021 [76]; Kassaw & Pandey, 2020 [90]; Lee et al., 2020 [98]; W. Li et al., 2021 [103]; Mangolian Shahrbabaki et al., 2021 [112]; Quandt et al., 2020 [162]; Scarpellini et al., 2021 [176])	**Social distancing measures/ Lockdown**^**b**^ (Auðardóttir & Rúdólfsdóttir, 2020 [11]; Dumbre et al., 2020 [56]; Farghaly et al., 2020 [61]; Lee et al., 2020 [98]; Z. Liu et al., 2020 [108]; Mangiavacchi et al., 2021 [111]; Moore et al., 2020 [127]; Neece et al., 2020 [139]; Ozturk Eyimaya & Yalçin Irmak, 2020 [148]; Quandt et al., 2020 [162]; Sama et al., 2020 [174]; Shah et al., 2021 [178]) **Disrupted early childhood education- school and daycare centers closures**^**b**^ (Bao et al., 2020; Cui et al., 2021 [41]; Lee et al., 2020 [98]; Z. Liu et al., 2020 [108]; Moore et al., 2020 [127]; Moscardino et al., 2021 [129]; Ozturk Eyimaya & Yalçin Irmak, 2020 [148]; Quenzer-Alfred et al., 2021 [163]; Sama et al., 2020 [174]; Scarpellini et al., 2021 [176]) **Access barriers to health services**^**b**^ (Ayas et al., 2020 [12]; Brisca et al., 2021 [17]; Cui et al., 2020 [42]; Quandt et al., 2020 [162])	
Healthcare and Childcare Services			**Impact of telehealth/ virtual healthcare**^**a**^ (Aksoy Derya et al., 2020 [5]; Farhadi et al., 2021 [62]; Naeem et al., 2020 [135]) **Health insurance**^**a**^ (Patrick et al., 2020 [151]) **Daycare services**^**a**^ (Ayas et al., 2020 [12]; Cui et al., 2020 [42]; Quenzer-Alfred et al., 2021 [163])

^a^Positive association

^b^Negative association

^c^Positive and Negative associations reported

#### Maternal mental health

This analysis is based on an understanding that mental disorders exist in a spectrum. As such, a dimensional approach is assumed as the basis to inform the individual lived experience of suffering and resilience in the targeted population. The approach considers the differing resources that individuals may have at their disposal based on their unique developmental trajectories, as well as mediating protective factors such as different levels of social support, access to health services, or income stability.

The principle of convergence of evidence from multiple fields [150] oriented our analysis of the risk factors at play, largely informed by the sociological, economic, environmental, and medical perspectives on the impact of poverty and inequality on mental health. Risk factors are understood as potential moderators to the outcome, affecting it differently according to their severity level. Risk factors will be categorized by domains, informed by the Lancet Commission on Global Mental Health and Sustainable Development [150].

Table 2 Summarizes the findings of included systematic reviews with meta-analysis (*n* = 5) on the impact of the pandemic on maternal mental health. Table 3 shows evidence gaps, categorized by domains, concerning the impact of the COVID-19 pandemic on maternal mental health.

**Table 2:** Systematic reviews with meta-analysis: Maternal Mental Health

Reviews	Main findings
**Mental Health of Pregnant and Postpartum Women During the Coronavirus Disease 2019 Pandemic: A Systematic Review and Meta-Analysis**	**Pregnant women:** **Pooled prevalence:** Anxiety: 37% (95% [CI] 25–49%) Depressive symptoms: 31% (95% CI 20–42%), Psychological distress: 70% (95% CI 60–79%) Insomnia: 49% (95% CI 46–52%) **Pooled relative risk:** Anxiety: 1.65 (95% CI: 1.25–2.19) Depressive symptoms: 1.08 (95% CI: 0.80–1.46) **Postpartum women:** **Pooled prevalence:** Depressive symptoms: 22% (95% CI 15–29%)
(Shorey et al., 2021) [181] **Anxiety and depressive symptoms of women in the perinatal period during the COVID-19 pandemic: A systematic review and meta-analysis**	**Antenatal:** **Pooled prevalence:** Anxiety: 40% (95% (CI): 0.27–0.52) Depressive symptoms: 27% (95% CI: 0.20–0.33) **Postnatal:** **Pooled prevalence:** Depressive symptoms: 17% (95% CI: 0.10–0.24) **On subgroup analysis based on geographical regions, only data from upper middle- and high-income countries in Asia, Europe and North America were available. Prevalence data from lower income countries and those in the Africa or South America continent were unavailable**
(Fan et al., 2021 [59]) **Psychological effects caused by COVID-19 pandemic on pregnant women: A systematic review with meta-analysis**	**Pregnant women:** **Pooled prevalence:** Anxiety: 42% (95%CI: 26%–57%) Depression: 25% (95%CI 20%–31%)
(Hessami et al., 2020) [81] **COVID-19 pandemic and maternal mental health: a systematic review and meta-analysis**	**Pregnant and postpartum women:** **Polled scale scores differences during/pre pandemic:** EPDS: 0.40 (95% CI: − 0.05 − 0.86, *p* = .083) STAI: 0.82 (95% CI: 0.49 − 1.16, *p* < .001)
(Chmielewska et al., 2021) [32] **Effects of the COVID-19 pandemic on maternal and perinatal outcomes: a systematic review and meta-analysis**	**Postpartum women:** **Polled scale scores difference during/pre pandemic:** EPDS: 0·42 (95% CI 0·02–0·81)

**Table 3:** Main evidence gaps, categorized by domains, concerning the impact of the COVID-19 pandemic on maternal mental health

Maternal Mental Health	
Health Care	1.Prevalence as well as magnitude and significance of change in obstetric violence and its impact on maternal mental health, stratified by socioeconomic status, country/sub-regions 2.Level of access and quality of ante and postnatal care services, stratified by socioeconomic status, country/sub-regions 3.Level of access and quality of mental health care services, especially substance abuse disorder services, stratified by socioeconomic status, country/sub-regions
Relational	1.Intimate partner violence- overall prevalence, as well as magnitude and significance of change, reported by subtype: psychological, physical, sexual. Stratified by socioeconomic status and country/sub-regions 2.Differential impact of multiple risk factors on the mental health of primiparous mothers 3.Added impact of bereavement, stratified by levels of support, access to mental health services, and presence of multiple risk factors contributing to elevated stress and depression risk factors
Demographic	1.Cross-cultural and culture-specific relationship between maternal age and: (1) prevalence of elevated stress, anxiety, and depression symptoms; (2) emotion regulation skills; (3) financial autonomy 2.Cross-cultural and culture-specific relationship between maternal ethnicity and: (1) prevalence of elevated stress, anxiety and depression symptoms; (2) access barriers to health services; (3) financial autonomy; (4) employment, financial and food insecurity; (5) neighborhood socioeconomic status as a compound measure of income, housing and public infrastructure, safety and security, deprivation, overcrowding, and access to cultural resources
Economic	1.Food insecurity: prevalence as well as magnitude and significance of change, stratified by country, sub-regions, and type concerning quantity and quality of household dietary patterns 2.Impact of financial strain on family health insurance: prevalence as well as magnitude and significance of change, stratified by country and sub-regions, as well as family socioeconomic strata, levels of access to health services, quality of health services 3.Change in quality of work productivity and performance during lockdown restrictions, in the presence of increased and unbalanced caregiving and housework responsibilities
Neighborhood	1.Association of maternal mental health and well-being and living conditions: (1) distal: city and neighborhood infrastructure or deprivation; (2) proximal: community safety and security, housing structure, overcrowding, WASH conditions, access to recreational activities, schools, daycare centers, urban parks
Environmental	1.Impact on maternal mental health and wellbeing of the interaction between the COVID-19 pandemic and environmental events, such as: (1) natural disasters; (2) industrial disasters; (3) war and conflict; (4) climate change; (5) forced migration; (6) trauma and violence
Social and Cultural	1.Cross-cultural and culture-specific relationship between maternal mental health and wellbeing and: (1) community social capital; (2) social stability; (3) individual social capital and participation

Protective factors identified in the selected studies were frequently related to individual emotional and psychological resources, namely latent constructs such as self-kindness, resilience, or perceived self-efficacy. The role of parental personality traits such as neuroticism or psychological flexibility were scrutinized as components of complex models correlating children and parental mental health, moderated, for instance, by family functioning impact or presence of other family members outside the maternal-infant dyad [44, 121].

Studies that analysed risk factors related to unbalanced caregiving and housework responsibilities were all conducted in high or upper-middle-income countries [11, 32, 39, 40, 47, 64, 70, 73, 79, 83, 110, 116, 122, 130, 157, 177, 202, 211, 212, 214, 215, 219] pointing to a large evidence gap concerning the exacerbation of gender inequities during lockdown measures and its impact on maternal well-being in low- and middle-income countries (LMICs).

Importantly, this gap extends to overall descriptive findings regarding the prevalence of elevated maternal stress, anxiety, and depressive symptoms, as well as associated risk factors across all areas as suggested by the low prevalence of included publications from low and lower-middle-income countries, 0.97% and 5.23%, respectively.

Disrupted sleeping and dietary patterns, as well as sleep deprivation, were as commonly reported as unbalanced and increased caregiving and housework responsibilities during the lockdown. This parallel suggests that the negative health impact on maternal wellbeing could be mitigated by a more balanced division of such responsibilities within households on lockdown, especially when considering children’s age as a relevant factor to maternal burnout. Equivalently, lack of support has often been directly associated with worsened maternal mental health status.

While access barriers to health services pose an obvious threat to maternal health, barriers to accessing clear, concise, and sufficient information regarding health risks related to COVID appeared as a major source of concern to mothers. Fear of infection and vertical transmission, as well as fear of adverse birth outcomes, were commonly registered risk factors. Conversely,  effective public health communication efforts would be directly beneficial to maternal mental health, in diverse contexts.

#### Early childhood development

Our review indicated that robust, consistent, and up-to-date global/regional estimates of the pandemic's impact on children`s morbidity and mortality burdens are not yet consolidated in the literature.

Concerning summarized findings on health outcomes of neonates born to COVID-19 positive mothers, we found 8 systematic reviews without meta-analysis [1, 8, 81, 85, 88, 132, 149, 218] and 5 with meta-analysis, presented in Table 4. Table 5 shows evidence gaps, categorized by domains, concerning the impact of the COVID-19 pandemic on early childhood development.

**Table 4:** Systematic reviews with meta-analysis: Neonatal Outcomes

Reviews	Main findings
(Chmielewska et al., 2021) [32] **Effects of the COVID-19 pandemic on maternal and perinatal outcomes: a systematic review and meta-analysis**	**Pooled odds ratios:** **Changes during x before pandemic:** **Stillbirth increase:** 1·28 (95% CI: 1·07–1·54) **Maternal death increase:** 1·37 (95% CI: 1·22–1·53) **Preterm birth < 37 weeks' gestation not significantly changed:** 0·94 (95% CI: 0·87–1·02) **Preterm births < 37 weeks' gestation decreased in high-income countries:** 0·91 (95% CI: 0·84–0·99) **Spontaneous preterm birth decreased in high-income countries:** 0·81 (95% CI: 0·67–0·97) **Surgically managed ectopic pregnancies increased:** 5·81 (95% CI: 2·16–15·6) **No significant changes in any other outcomes**
(Jafari et al., 2021) [86] **Clinical characteristics and outcomes of pregnant women with COVID-19 and comparison with control patients: A systematic review and meta-analysis**	**Pooled odds ratios:** **More common among COVID-19 positive mothers:** **Low birth weight**: 9 (95% CI: 2.4–30) **Preterm birth:** 2.5 (95% CI: 1.5–3.5) **Pooled prevalences:** **NICU admission**: 43% (95% CI: 2–96) **Fetal distress**: 30% (95% CI: 12–58) **Low birth weight**: 25% (95% CI:16–37) **Vertical transmission**: 5.3% (95% CI:1.3–16) **Positive SARS-CoV-2 test:** 8% (95% CI: 4–16)
(Capobianco et al., 2020) [23] **COVID-19 in pregnant women: A systematic review and meta-analysis**	**Pooled prevalences:** **Preterm births:** 23% (95% CI: 11.0 –39.0) **Infected neonates**: 6% (95% CI: 2.0 –12.0)
(Di Mascio et al., 2020) [51] **Outcome of coronavirus spectrum infections (SARS, MERS, COVID-19) during pregnancy: a systematic review and meta-analysis**	**Pooled prevalences:** **Preterm birth < 37 weeks**: 41.1% (95% CI: 25.6–57.6) **Perinatal death**: 7.0% (95% CI: 1.4–16.3) **No signs of vertical transmission**
(Diriba et al., 2020) [55] **The effect of coronavirus infection (SARS-CoV-2, MERS-CoV, and SARS-CoV) during pregnancy and the possibility of vertical maternal–fetal transmission: a systematic review and meta-analysis**	**Pooled prevalences:** **Preterm birth < 37 weeks of gestation**: 14.3%, **Fetal growth restriction:** 2.8% **Fetal distress:** 26.5% **Neonatal asphyxia:** 1.4% **Apgar score < 7 at 5 min:** 1.2% **Admitted to ICU:** 11.3% **Perinatal death:** 2.2% **No reports of vertical transmission in uterus**

**Table 5:** Main evidence gaps, categorized by domains, concerning the impact of the COVID-19 pandemic on early childhood development

Early Childhood Development	
Health and Development	1.Global and regional robust and current estimates of the added morbidity and mortality burden of COVID-19 and communicable and non-communicable diseases, especially in LMICs undergoing humanitarian crisis 2.Up to date global and regional robust estimates on the state of the Sustainable Development Goals achievement, and their direct impact on ECD 3.Up to date consistent global and regional estimates of immunization delays 4.Impact of tele/virtual healthcare on ECD- stratified by presence of neurodevelopmental disorders and/or chronic illnesses, socioeconomic status 5.Level of access and quality of mental health care services, stratified by socioeconomic status, country/sub-regions 6.Impact on education: quality and pedagogical milestones, stratified by gender, ethnicity, and family socioeconomic status 7.Cross-cultural and country-specific impact on socioemotional development, based on longitudinal data, stratified by family socioeconomic status, gender, age, ethnicity 8.Differential impact on health status and developmental milestones concerning children with neurodevelopmental disorders and/or chronic illnesses
Relational	1.Harsh parenting / abusive parental behavior- overall prevalence, as well as magnitude and significance of change, reported by subtype: psychological, physical, sexual. Stratified by socioeconomic status and country/sub-regions 2.Added impact of bereavement, stratified by levels of support, access to mental health services, family socioeconomic status, caregivers’ mental health status 3.Overall estimates of incidence and impact of orphanhood on the health and development of children, stratified by family socioeconomic status, presence of secondary or tertiary caregivers, institutional resources, children’s rights policies
Demographic	1.Cross-cultural and culture-specific differential impact concerning gender and ethnicity on developmental milestones and educational gaps, stratified by socioeconomic status
Economic	1.Impact of food insecurity: prevalence as well as magnitude and significance of change, stratified by country, sub-regions, and type concerning quantity and quality of household dietary patterns 2.Impact of financial strain on family health insurance: prevalence as well as magnitude and significance of change, stratified by country and sub-regions, as well as family socioeconomic strata, levels of access to health services, quality of health services
Neighborhood	1.Association of ECD and living conditions: (1) distal: city and neighborhood infrastructure or deprivation; (2) proximal: community safety and security, housing structure, overcrowding, WASH conditions, access to recreational activities, schools, daycare centers, urban parks
Environmental	1.Interaction between the pandemic and environmental events, such as: (1) natural disasters; (2) industrial disasters; (3) war and conflict; (4) climate change; (5) forced migration; (6) trauma and violence
Social and Cultural	1.Cross-cultural and culture-specific relationship between ECD and: (1) community social capital; (2) social stability

Chmielewska et al. [32] identified an unchanged global prevalence in preterm birth and neonatal death, pointing out, however, that data suggest increased rates of both outcomes in LMICs and decreased in high-income countries (HICs), apparently driven by a reduction in spontaneous preterm birth in HICs. This finding stresses the role of insufficient capacity of healthcare systems to cope with the pandemic. Furthermore, despite large evidence gaps, the review shows that stillbirth rates might be particularly increased in LMICs. These findings highlight the urgent need for more contributions from LMICs regarding the differential impact of the pandemic and its interplay with local socioeconomic conditions, as well as pre-existing and exacerbated disease burdens concerning maternal and neonatal health. While the strong association between parental and children's mental health was pointed out in various studies [20, 31, 49, 72, 99, 151, 169, 183, 184] some sought to identify mediating mechanistic effects, such as parental emotion regulation capacity during lockdown [159, 160] or parental perceived manageability of the dyadic relationship [183, 184].

Most studies have either assessed worsened mental health states, such as the significantly higher prevalence of anxiety and depression symptoms [43, 100, 100, 174, 189, 195, 217] or changes in children’s behavioural patterns or developmental markers. Despite scarce evidence and particularly large evidence gaps concerning LMICs, few studies highlighted fundamental aspects of the pandemic's impact on ECD.

While reported changes in sleeping patterns were mixed [11, 24, 25, 84, 108, 137, 225] sedentarism during social isolation restrictions appears to be exceptionally high in one Canadian study [127], based on WHO recommendations. Additionally, a U.S. study utilized pre-existing longitudinal data on expected reading ability gain among kindergarten children to predict changes associated with school closures [13], concluding that in the absence of formal in-person education such gains would decrease by 31%. However, the model also predicted that the loss could be ameliorated by 42% if children had books read to them daily.

The impact of disrupted early childhood education undertaken in school and daycare centers that remained closed during lockdown was well documented in the literature. Equivalently, many studies sought to identify disrupted daily habits, such as sleeping and dietary patterns, as well as changes in screen time, regular physical activities, and loss of playing time outdoors. The potential associations between school closures and behavioral changes could be better explored, ideally through a careful examination of how factors such as socioeconomic status, the presence of welfare policies, or caregiving dynamics might inform these associations.

Overall, the review identified scattered initiatives in documenting the impact of the pandemic on developmental milestones and educational gaps. A more synthetic analytical approach would better inform how such impacts could differ by sociocultural context, gender, ethnicity, and socioeconomic status. Importantly, the acute paucity of longitudinal data on the impact of the pandemic on early childhood development is a considerable barrier to a more comprehensive understanding of its ongoing effects on the health and wellbeing of children worldwide, during a key developmental period. This scarcity of consistent information on the subject also hinders the analysis of its relationship with social determinants at play, which in turn is needed for the design and implementation of effective public health policies aiming at fostering healthier developmental paths.

Finally, most studies examined the multiple pathways through which the advent of the pandemic might have impacted ECD under scenarios that preserved the normal functioning of institutions to a considerable extent, but not much is known about such impact under ongoing humanitarian crises. Certainly, there is a large gap regarding the potential interaction between the pandemic and concomitant events that have likely acted as catalysts, magnifying the impact on ECD, such as natural and industrial disasters, war and conflict, climate change, forced migration, as well as trauma and violence on diverse scales.

#### Parenting practices

From the 497 empirical studies and case reports included in this review only 69 (14%) directly or indirectly assessed parental practices during the COVID-19 pandemic, comprising not only descriptive studies on children's mental health and behaviour patterns but also assessments of potential predictors and mechanistic effects mediating parental mental health and children’s wellbeing.

Table 6 shows evidence gaps, categorized by domains, concerning the impact of the COVID-19 pandemic on parenting practices.

**Table 6:** Main evidence gaps, categorized by domains, concerning the impact of the COVID-19 pandemic on parenting practices

Parenting Practices	
Behavioral	1.Global/regional estimates of adherence to WHO guidelines on breastfeeding and newborn care, stratified by type of breastfeeding practice, preterm birth, weight at birth, newborn comorbidities, level, and type of support offered to the mother, and family socioeconomic status. (e.g., Scala, M. et al., 2020^452^) 2.Differential impact of multiple risk factors on parental practices delivered by primiparous mothers 3.Differential impact of elevated stress, anxiety and depressive symptoms reported by secondary caregivers on parental practices
Health and Development	1.Global/regional estimates of immunization schedule compliance, especially in LMICs undergoing humanitarian crisis 2.Global/regional up to date estimates of the differential impact of access barriers to health services, rehabilitation centers and school closures on the wellbeing of children with neurodevelopmental disorders and/ or chronic illnesses 3.Global/regional estimates of the impact of tele/virtual healthcare on ECD, stratified by presence of neurodevelopmental disorders and/or chronic illnesses and socioeconomic status 4.Global/regional estimates on the impact of homeschooling on education quality and pedagogical milestones, stratified by gender and family socioeconomic status
Relational	1.Harsh parenting / abusive parental behavior: overall prevalence, as well as magnitude and significance of change, reported by subtype: psychological, physical, sexual. Stratified by socioeconomic status and country/sub-regions 2.Cross-cultural and culture-specific estimates on maternal-newborn and infant attachment quality, stratified by socioeconomic status, level and type of support offered to the mother, and family financial stability
Economic	1.Impact of food insecurity on family feeding practices: prevalence as well as magnitude and significance of change, stratified by country, sub-regions, and type concerning quantity and quality of household dietary patterns 2.Impact of loss of family health insurance coverage on care practices, especially concerning neonates and children with neurodevelopmental disabilities and/or chronic illnesses, stratified by sub-regions, as well as family socioeconomic status, levels of access to health services, and quality of health services available
Neighborhood	1.Association of parental practices and living conditions, stratified by: (1) city and neighborhood infrastructure or deprivation; (2) community safety and security, housing structure, overcrowding, WASH conditions, access to recreational activities, schools, daycare centers, urban parks
Environmental	1.Impact on parental practices of the interaction between the COVID-19 pandemic and environmental events, such as: (1) natural disasters; (2) industrial disasters; (3) war and conflict; (4) climate change; (5) forced migration; (6) trauma and violence
Social and Cultural	1.Cross-cultural and culture-specific relationship between parental practices and: (1) community social capital; (2) social stability

A pressing matter concerning newborn care raised by a few studies [175, 195, 203] is whether COVID-19 positive mothers and their newborn babies should be separated to avoid vertical transmission. Recent guidelines by the WHO (World Health Organization, 2021) recommend maintaining close mother-baby contact as a fundamental measure to prevent newborn morbidity and mortality during the pandemic, stimulating practices such as kangaroo care and breastfeeding, especially among babies born preterm or at low birth weight. Accordingly, the number of mother-newborn separation days in one study [203] was found to be negatively associated with neurobehavioral development at 3 months old, after adjusting for preterm and NICU admission. Yet, evidence suggests that family visitations and receipt of developmental care activities were less frequent and shorter [175] among preterm infants admitted at a U.S. newborn intensive care unit (NICU).

Similarly, one review [199] found a stark misalignment between WHO recommendations and country-specific guidelines regarding breastfeeding and newborn care practices during the pandemic across 33 countries. The review points out that not only inconsistencies on guidelines were abundant (and none recommended all aspects of WHO guidance), but they also frequently included recommendations against practices supportive of breastfeeding, even in countries with high infant mortality rates. Despite a considerable evidence gap on the prevalence and change in breastfeeding practices by type—exclusive or complementary—few studies [18, 198] have found associations between breastfeeding cessation or non-initiation with lack of support and fear of vertical transmission.

Significant associations between specific parenting styles and children’s behaviours have been reported in the literature [127, 148, 152, 169, 183, 184] pointing to the detrimental effect of parental behavioural traits such as inconsistency and inflexibility [44, 148, 152]. Those studies describe a cycle where parental and infant mental suffering and burnout are bidirectionally associated with dysfunctional family dynamics. Correspondingly, they also suggest that greater use of constructive parenting strategies, such as supportive and consistent practices, are associated with greater family cohesion. Such strategies are predicted by parental emotion regulation capacity and psychological flexibility, which in turn was found to be highly associated with parental stress levels. Importantly, coercive practices were found to increase in situations of high parental stress [128, 152, 168, 171, 183, 184].

Finally, parental practices concerning children with disabilities or debilitating health conditions during the pandemic were reported in few studies [3, 12, 15, 17, 27, 30, 75, 123, 139, 143, 175] in which the main challenges identified by caregivers were routine disruptions accompanied by loss of access to stimulating activities, increased behavioural difficulties, health concerns regarding COVID-19 infection, loss of in-person rehabilitation support activities, and decreased access and quality of care services offered to children within overburdened health systems.

## Discussion

### Summary of evidence

Our findings suggest that there is a much larger body of evidence so far concerning the impact of the pandemic on maternal mental health than there is on early childhood development, and even less so on parental practices. We hypothesize that this imbalance is partially due to differing degrees of difficulty in accessing and enrolling participants in empirical studies. For instance, it might be overall easier to recruit women who attend health services based on convenience sampling, or to launch an online open survey through social media platforms, than it is to enroll children during periods of school and daycare center closures. Furthermore, in order to examine parental practices based on observed behavior it is often necessary to access households in person, which poses additional health risks of COVID contamination to both study participants and researchers.

Certainly, measuring the magnitude of the pandemic impact on each outcome category exceeds the scope of this review. However, some relevant findings synthesizing global burden estimates have been pointed out by specific studies. O'Driscoll et al. [144] estimated age-specific mortality and immunity patterns of SARS-CoV-2 across 45 countries, demonstrating that the age distribution of excess deaths due to COVID-19 in age groups younger than 65 years old was consistent across different settings. That study concluded that the infection fatality ratio (IFR) was lowest among children 5–9 years old (> 0.001), closely followed by children younger than five (> 0.01); male children’s IFR ranked slightly higher than females in both age groups [144]. 

Importantly, Hillis et al. [82] analysed global estimates of orphanhood associated with COVID-19, finding that between March 2020 and April 2021, approximately 1.134.000 children (95% CI: 884.000 – 1.185.000) experienced the death of primary caregivers, while 1.562.000 children (95% CI: 1.299.000 – 1.683.000) lost at least one primary or secondary caregiver. The study has identified countries with high rates of primary caregiver deaths per 1,000 children, including Peru (10.2), South Africa (5.1), Mexico (3.5), Brazil (2.4), Colombia (2.3), Iran (1.7), USA (1.5), Argentina (1.1), and Russia (1.0). It also estimated that between two to five more children experienced the death of fathers than mothers [82]. These alarming findings regarding orphanhood highlight the need for longitudinal studies that are capable of examining the compound impact of the pandemic on child development over time, across diverse sociocultural contexts.

This study addresses an evidence gap concerning the analysis of the impact of the COVID-19 pandemic on all three fields, namely, maternal mental health, early childhood development, and parental practices, globally. The breadth of our review lies not only in the diversity of themes explored by empirical research, case reports, and literature reviews within each field but also in the large period of publication determined by our inclusion criteria, from January 1st, 2020, until June 9th, 2021, capturing the development of relevant research efforts during diverse stages of the pandemic, across the world.

Regarding this global research effort, three points should be highlighted. Firstly, the deeply unequal contribution of evidence concerning countries’ income level groups, with as much as 63.19% of empirical research studies and case reports having been conducted in high-income countries, and as little as 0.97% originating from low-income countries. Secondly, the majority (67.4%) of primary research studies has employed a cross-sectional design, even when addressing etiologic questions concerning potential determinants of negatively impacted states of maternal mental health, childhood development, or parenting. Thirdly, convenience sampling methods have been utilized by almost half of all primary research studies (48.8%); specifically, 34% of studies recruited participants exclusively via open-access links disseminated through online social platforms.

Some considerations should be made based on these findings. Concerning the evidence gap based on country-level income group: there is relevant evidence [32] suggesting that stillbirth and maternal mortality rates have sharply increased in LMICs during the pandemic, and yet only 6% of the selected studies has been conducted within low and lower-middle income countries. The co-occurrence of inconsistent and misguided recommendations on key parental practices such as breastfeeding and neonatal care [199], and the general convergence of risk factors, sometimes within ongoing humanitarian crises, especially endangers the wellbeing of mothers and young children in LMICs. Without sufficient quality data it is impossible to estimate, and even more difficult to effectively act upon, this complex scenario which poses the threat of lifelong negative consequences on the health of young children currently experiencing toxic stress and deprivation during the first years of their life. The second consideration regards the overall quality and generalizability of evidence produced by most research efforts within all three fields, since the pandemic onset. The gap in longitudinal data is expected, considering the relative novelty of the phenomenon. However, it certainly hinders the possibility to grasp mechanistic effects better informing health outcomes, creating added difficulties to the timely monitoring of important changes on the magnitude of the pandemic impact on population health status. Finally, the massive employment of low-quality sampling methods, compromising internal and external validity of evidence across fields, clearly signals the need for more concerted research efforts to support the implementation of public policies that prioritize the most serious problems for maternal and child health. Importantly, there are considerable ethical and methodological difficulties in robustly assessing the pandemic impact on specific variables, such as intimate partner violence, abusive parental practices, or maternal-infant attachment quality. To minimize the risk of bias, the same population should be assessed before and during the pandemic, utilizing the same methods and instruments, which poses large limitations on research efforts. Additionally, there is the added barrier of assessing data on intrafamilial violence during periods where lockdowns have been imposed. In such times, not only do victims only do victims frequently havefrequently have no alternative except cohabitating with aggressors, but also pre-existing protective institutional resources may have been limited, for instance, by redirected investments to mitigate the burden of disease associated with COVID-19. Both conditions seem to stimulate participants’ hesitation of disclosing sensitive information regarding intrafamilial violence, even when anonymized. Unfortunately, even after considering these potential barriers to more accurate estimates, existing data on the subject show alarmingly increased rates of IPV and child abuse during the pandemic.

We would like to highlight four important findings from our study. First, because of the diverse and various pathways through which the mental health of caregivers might be directly correlated with the health and development of young children under their care, there are equivalently various measures that can be taken to foster a global improvement of outcomes within this relationship.

Second, even though certain social measures that are needed to mitigate the spread of COVID-19, such as physical isolation, might contribute as stressors to the wellbeing of caregivers and children [1759, 155, 319, 359, 639, 832, 879, 889, 1010, 1113, 1354, 1424, 1519, 171, 394, 1204, 1447, 1606, 1756, 1519, 234, 443, 324, 262, 361, 137, 343, 398, 1204, 1807, 2099, 639] this review found evidence that the added financial burden [831, 1515, 570, 517, 662, 692, 1073, 1140, 1377, 1519, 1462, 2107, 1351, 817, 928, 975, 186] and loss of access to health services [1354, 330, 879, 832, 176, 852, 1186, 398, 2174, 2192, 2123] seem to be component causes that are equally relevant in producing the reported negative outcomes in maternal mental health and ECD. More data is needed to estimate the differential impact of the pandemic on our outcome categories of interest in a scenario where physical distancing measures were adopted, within comparable populations, and that have experienced different levels of added financial hardship, employment, and food insecurity, and access barriers to health care throughout the pandemic.

Third, although the compound effect of various risk factors on the mental health and wellbeing of caregivers and children is highly dependent on local and dynamic circumstances during the pandemic, pre-existing socio-cultural conditions, such as gender inequality, seem to have been exacerbated in the absence of institutional and relational support resources, e.g., school and daycare centres closures [234, 443, 324, 262, 361, 145, 960, 1634, 1468, 1855, 1145]. The overburden created by unbalanced childcare and housework workloads performed by female caregivers appeared as one of the most influential risk factors for increased maternal stress, decreased maternal mental health, and parenting styles that are detrimental to ECD [466, 472, 775, 1743, 290, 384, 284, 774, 1409, 1920, 1143, 1473, 1059, 1056, 707, 582, 600, 662, 831, 186, 137, 1045, 1351, 1399].

Lastly, global estimates on orphanhood due to COVID-19 [82] identify the urgent need to map and timely address, both through international and local collaborations, this critical situation experienced by children in key developmental stages, especially regarding institutionalisation and immediate care needs.

### Limitations

The assumption of mothers as the most frequent primary caregiver has implications on our findings. We have not systematically retrieved data on the impact of the pandemic on the mental health of alternative and/or secondary caregivers, which appears as an important component of constructive parenting practices and healthy family dynamics in the literature. Furthermore, even though the physical health of caregivers during the pandemic, especially pregnant and postpartum women, is intimately connected to their mental health status, such investigation surpasses the scope of this review. Finally, our search strategy did not include controlled vocabulary terms referring to specific parenting practices, such as “breastfeeding” or “home schooling”.

Overall, data on maternal mental health was more voluminous, structured, and easily accessible than on ECD, and even more so for parental practices. Most data concerning ECD was indirectly reported by caregivers, which poses a larger liability to information bias. These limitations, along with the frequent use of low-quality sampling methods, signal the added barriers to recruitment and selection of participants to research studies during the pandemic, particularly so during periods of lockdowns.

### Conclusions

The central conclusion of this review is that the inherent interplay between the mental health of caregivers and the health and development of young children under their care, mediated by parenting practices, is deeply permeable to social determinants. While this finding is certainly echoed by a robust body of literature predating the pandemic, our review contributes to the understanding of the extent to which the pandemic has exacerbated previous conditions of vulnerability, frequently creating interactions between risk factors that deeply magnify the effects of inequality on the health and wellbeing of specific populations.

## Supplementary Information

**Additional file 1.**

## Data Availability

Not applicable.
