# Supplementary material for: Impact of the COVID-19 pandemic on maternal mental health, early childhood development, and parental practices: a global scoping review
Source: BMC Public Health. 2023 Feb 24;23:388. doi: 10.1186/s12889-023-15003-4 (PMC9950022; doi:10.1186/s12889-023-15003-4)
Supplement: Supplementary file 1 — Additional file 1. [file 12889_2023_15003_MOESM1_ESM.zip › 12889_2023_15003_MOESM1_ESM.docx]

**Database Search Strategy Code and Description**

**Impact of the COVID-19 pandemic on maternal mental health, early childhood development, and parental practices: a global scoping review**

*^a^Department of Global Health and Population, Harvard T. H. Chan School of Public Health, Boston, U.S.A; ^b^Department of Community Health, Federal University of Ceará, Fortaleza, Brazil; ^c^Pontifical Catholic University, Rio de Janeiro, Brazil; ^e^Harvard Countway Library, Boston, U.S.A*

*Corresponding author: Ana Luiza Penna

Address: Department of Global Health and Population, Harvard T. H. Chan School of Public Health, 677 Huntington Avenue, Boston, MA, United Stated, 02115

e-mail: anapenna@g.harvard.edu

Phone number: +1 617 8523983

PubMed (National Library of Medicine, NCBI)

((("Mothers"[Mesh] OR "Pregnancy"[Mesh] OR "Pregnant Women"[Mesh] OR "Maternal Health"[Mesh] OR "Pregnancy Complications"[Mesh:NoExp] OR "Pregnancy Complications, Infectious"[Mesh:NoExp] OR "Depression, Postpartum"[Mesh] OR "Prenatal Exposure Delayed Effects"[Mesh] OR pregnan*[tiab] OR mother*[tiab] OR maternal[tiab] OR prenatal[tiab] OR perinatal[tiab] OR antenatal [tiab] OR postnatal[tiab])

AND

("Mental Health"[Mesh] OR "Stress, Psychological"[Mesh] OR "Anxiety"[Mesh:NoExp] OR "Psychological Distress"[Mesh] OR "Depression"[Mesh] OR "Adaptation, Psychological"[Mesh] OR "Fear"[Mesh] OR "Loneliness"[Mesh] OR "Sadness"[Mesh] OR anxiety[tiab] OR cope[tiab] OR coping[tiab] OR depression[tiab] OR depressive[tiab] OR distress[tiab] OR depression[tiab] OR depressive[tiab] OR distress[tiab] OR fear[tiab] OR lonliness[tiab] OR "mental health"[tiab] OR "emotional stress" [tiab] OR "mental stress" [tiab] OR "psychological stress"[tiab] OR "psychological status"[tiab] OR sadness[tiab]))

OR

"Parenting"[Mesh] OR "Parent-Child Relations"[Mesh] OR "Child Care"[Mesh] OR "Child Rearing"[Mesh:NoExp] OR "Maternal Behavior"[Mesh] OR parenthood[tiab] OR parenting[tiab] OR "parent child"[tiab] OR "maternal behav*"[tiab] OR "maternal fetal"[tiab] OR "prenatal bond*"[tiab] OR "maternal infant"[tiab] OR "maternal child"[tiab] OR "mother child"[tiab] OR "mother infant"[tiab] OR "child care"[tiab] OR childcare[tiab] OR "child rearing"[tiab]

OR

"Child Development"[Mesh] OR "fetal development*"[tiab] OR "prenatal development*"[tiab] OR "neonatal development*"[tiab] OR "perinatal development*"[tiab] OR "postnatal development*"[tiab] OR "infant development*"[tiab] OR "child development*"[tiab] OR "childhood development*"[tiab]) AND

("Coronavirus"[Mesh:NoExp] OR "Betacoronavirus"[Mesh:NoExp] OR "Coronavirus Infections"[Mesh:NoExp] OR "COVID-19"[Supplementary Concept] OR "severe acute respiratory syndrome coronavirus 2"[Supplementary Concept] OR "Pandemics"[Mesh] OR ((wuhan[tiab] OR hubei[tiab] OR huanan[tiab]) AND ("severe acute respiratory"[tiab] OR pneumonia[tiab]) AND (outbreak[tiab])) OR coronavirus[tiab] OR "corona virus"[tiab] OR coronavirinae[tiab] OR coronaviridae[tiab] OR betacoronavirus[tiab] OR covid19[tiab] OR "covid 19"[tiab] OR nCoV[tiab] OR "CoV 2"[tiab] OR CoV2[tiab] OR sarscov2[tiab] OR "sars CoV 2"[tiab] OR 2019nCoV[tiab] OR "novel CoV"[tiab] OR "wuhan virus"[tiab] OR pandemic[tiab])

AND

2020/01/01:2030/12/31[dp]

-1474 results 06/09/2021

Embase (Elsevier, Embase.com)

settings:

source = Embase

date of publication = 2020-2021

1)

'mother'/de OR 'pregnancy'/de OR 'pregnant woman'/de OR 'maternal welfare'/exp OR 'pregnancy complication'/de OR 'postnatal depression'/de

OR

2)

(pregnan* OR mother* OR maternal OR prenatal OR perinatal OR antenatal OR postnatal):ab,ti,kw

AND

3)

'mental health'/exp OR 'mental stress'/de OR 'emotional stress'/exp OR 'anxiety'/de OR 'depression'/de OR 'minor depression'/de OR 'coping behavior'/de OR 'fear'/de OR 'loneliness'/de OR 'sadness'/de

OR

4)

(anxiety OR cope OR coping OR depression OR depressive OR distress OR depression OR depressive OR distress OR fear OR lonliness OR "mental health" OR "emotional stress" OR "mental stress" OR "psychological stress" OR "psychological status" OR sadness):ab,ti,kw

OR

5)

'parental behavior'/de OR 'parenthood'/de OR 'child parent relation'/exp OR 'mother fetus relationship'/de OR 'child rearing'/de OR 'child care'/de OR 'infant care'/exp

OR

6)

(parenthood OR parenting OR "parent child" OR "maternal behav*" OR "maternal fetal" OR "prenatal bond*" OR "maternal infant" OR "maternal child" OR "mother child" OR "mother infant" OR "child care" OR childcare OR "child rearing"):ab,ti,kw

OR

7)

'prenatal development'/exp OR 'perinatal development'/de OR 'postnatal development'/de OR 'child development'/de

OR

8)

("fetal development*" OR "prenatal development*" OR "neonatal development*" OR "perinatal development*" OR "postnatal development*" OR "infant development*" OR "child development*" OR "childhood development*"):ab,ti,kw

AND (all sets above combined with OR)

9)

('Coronaviridae'/de OR 'Coronavirinae'/de OR 'coronavirus disease 2019'/de OR 'Coronavirus infection'/de OR 'Betacoronavirus'/de OR 'Severe acute respiratory syndrome coronavirus 2'/de OR 'pandemic'/de) NOT 'conference abstract'/it

OR

10)

(coronavirus OR "corona virus" OR coronavirinae OR coronaviridae OR betacoronavirus OR covid19 OR "covid 19" OR nCoV OR "CoV 2" OR CoV2 OR sarscov2 OR "sars CoV 2" OR 2019nCoV OR "novel CoV" OR "wuhan virus" OR pandemic):ab,ti,kw NOT 'conference abstract'/it

(((1 OR 2) AND (3 OR 4)) OR 5 OR 6 OR 7 OR 8) AND (9 OR 10)

1065 results 06/09/2021

Psycinfo (American Psychological Association, Ebsco)

settings:

remove: apply equivalent subjects

document type = journal article

publication year = 2020 – 2021

1)

DE "Mothers" OR DE "Pregnancy" OR DE "Pregnancy Outcomes" OR DE "Postpartum Depression"

2)

[title OR abstract OR keywords]:

pregnan* OR mother* OR maternal OR prenatal OR perinatal OR antenatal OR postnatal

3)

DE "Mental Health" OR DE "Stress" OR DE "Psychological Stress" OR DE "Distress" OR DE "Anxiety" OR DE "Coping Behavior" OR DE "Depression (Emotion)" OR DE "Fear" OR DE "Loneliness" OR DE "Sadness"

4)

[title OR abstract OR keywords]:

anxiety OR cope OR coping OR depression OR depressive OR distress OR depression OR depressive OR distress OR fear OR lonliness OR "mental health" OR "emotional stress" OR "mental stress" OR "psychological stress" OR "psychological status" OR sadness

5)

DE "Parenting" OR DE "Parent Child Relations" OR DE "Mother Child Relations" OR DE "Childrearing Practices" OR DE "Child Care"

6)

[title OR abstract OR keywords]:

parenthood OR parenting OR "parent child" OR "maternal behav*" OR "maternal fetal" OR "prenatal bond*" OR "maternal infant" OR "maternal child" OR "mother child" OR "mother infant" OR "child care" OR childcare OR "child rearing"

7)

DE "Prenatal Development" OR DE "Infant Development" OR DE "Early Childhood Development"

8)

[title OR abstract OR keywords]:

"fetal development*" OR "prenatal development*" OR "neonatal development*" OR "perinatal development*" OR "postnatal development*" OR "infant development*" OR "child development*" OR "childhood development*"

9)

DE "Coronavirus" OR DE "Pandemics"

10)

[title OR abstract OR keywords]:

coronavirus OR "corona virus" OR coronavirinae OR coronaviridae OR betacoronavirus OR covid19 OR "covid 19" OR nCoV OR "CoV 2" OR CoV2 OR sarscov2 OR "sars CoV 2" OR 2019nCoV OR "novel CoV" OR "wuhan virus" OR pandemic

(((1 OR 2) AND (3 OR 4)) OR 5 OR 6 OR 7 OR 8) AND (9 OR 10)

169 results 06/09/2020

CINAHL (CINAHL Plus with Full Text, Ebsco)

settings

remove:

search within full text of articles

apply equivalent subjects

published date: 2020 – 2021

publication type: journal article

1)

MH "Mothers" OR MH "Pregnancy" OR MH "Pregnancy, High Risk" OR MH "Depression, Postpartum" OR MH "Expectant Mothers" OR MH "Maternal Exposure" OR MH "Prenatal Exposure Delayed Effects"

2)

[title OR abstract OR subject]:

pregnan* OR mother* OR maternal OR prenatal OR perinatal OR antenatal OR postnatal

3)

MH "Mental Health" OR MH "Stress, Psychological" OR MH "Psychological Distress" OR MH "Anxiety" OR MH "Depression" OR MH "Coping" OR MH "Fear" OR MH "Loneliness" OR MH "Sadness"

4)

[title OR abstract OR subject]:

anxiety OR cope OR coping OR depression OR depressive OR distress OR depression OR depressive OR distress OR fear OR lonliness OR "mental health" OR "emotional stress" OR "mental stress" OR "psychological stress" OR "psychological status" OR sadness

5)

MH "Parenting" OR MH "Parenthood" OR MH "Parent-Child Relations" OR MH "Mother-Child Relations" OR MH "Mother-Infant Relations" OR MH "Prenatal Bonding" OR MH "Child Rearing" OR MH "Maternal Behavior" OR MH "Infant Care" OR MH "Child Care"

6)

OR [title OR abstract OR subject]:

parenthood OR parenting OR "parent child" OR "maternal behav*" OR "maternal fetal" OR "prenatal bond*" OR "maternal infant" OR "maternal child" OR "mother child" OR "mother infant" OR "child care" OR childcare OR "child rearing"

7)

MH "Child Development" OR MH "Infant Development" OR MH "Fetal Development"

8)

[title OR abstract OR subject]:

"fetal development*" OR "prenatal development*" OR "neonatal development*" OR "perinatal development*" OR "postnatal development*" OR "infant development*" OR "child development*" OR "childhood development*"

9)

MH "Coronavirus" OR MH "COVID-19" OR MH "Coronavirus Infections" OR MH "Disease Outbreaks"

10)

[title OR abstract OR subject]:

coronavirus OR "corona virus" OR coronavirinae OR coronaviridae OR betacoronavirus OR covid19 OR "covid 19" OR nCoV OR "CoV 2" OR CoV2 OR sarscov2 OR "sars CoV 2" OR 2019nCoV OR "novel CoV" OR "wuhan virus" OR pandemic

(((1 OR 2) AND (3 OR 4)) OR 5 OR 6 OR 7 OR 8) AND (9 OR 10)

- 133 results 06/09/2020

- 2841 results retrieved across 4 databases

- 2158 unique results for screening in Covidence
