# Supplementary material for: Impact of the COVID-19 pandemic on maternal mental health, early childhood development, and parental practices: a global scoping review
Source: BMC Public Health. 2023 Feb 24;23:388. doi: 10.1186/s12889-023-15003-4 (PMC9950022; doi:10.1186/s12889-023-15003-4)
Supplement: Supplementary file 1 — Additional file 1. [file 12889_2023_15003_MOESM1_ESM.zip › 12889_2023_15003_MOESM2_ESM.docx]

**Empirical Research Studies: Invidivual Characteristics**

| **Study Reference** | **Country** | **Population** | **Sample size** | **Study Design** | **Sampling** | **Use of Validated Instruments** | **Primary Outcomes** |  |
| --- | --- | --- | --- | --- | --- | --- | --- | --- |
| (Brown & Shenker, 2021) | U.K. | Mothers / children <1 | 1219 | Prospective cohort (4 weeks follow-up) | Online open access link | No | Perceived readiness and impact of lockdown on breastfeeding, reasons for stopping |  |
| (Spinelli et al., 2020) | Italy | Parents / children 2-14 | 810 | Cross-sectional | Online open access link Snowball | Yes, PSI, CHAOS and ERC | Family socioeconomic risk |  |
| (Ozturk Eyimaya & Yalçin Irmak, 2020) | Turkey | Parents / children 6-13 | 1115 | Cross-sectional | schools | Yes, PPS | Amount of children's screen time |  |
| (Bıkmazer et al., 2020) | Turkey | Parents / children 6-18 | 3278 | Cross-sectional | Online open access link Snowball | Yes, PHQ and SDQ | Parental and children's distress |  |
| (Kotabagi, Fortune, et al., 2020) | U.K. | Pregnant women | 11 | Cross-sectional | Hospital | Yes, PHQ-9 and GAD-7 | Depression and anxiety |  |
| (Mahajan et al., 2020) | India | Pregnant women | 879 | Cross-sectional | Hospital (controled by precovid records) | No | Neonatal outcomes among COVID-positive mothers with multiple gestation pregnancies: proportion of preterm deliveries the same but admission to NICU and incidence of eclampsia and pre-eclampsia higher during pandemic |  |
| (Lauri Korajlija & Jokic-Begic, 2020) | Croatia | Mothers / children 0-18 | 827 | Cross-sectional | Snowball | Yes, CAS1/2 | COVID related anxiety |  |
| (Ahlers-Schmidt et al., 2020) | U.S.A | Pregnant and postpartum | 114 | Cross-sectional | Online open access link | No | COVID-knowledge, risk reduction behavior change, mental health, pregnancy-related and infant care behaviors, acceptability of covid vaccination |  |
| (Hiraoka & Tomoda, 2020) | Japan | Parents / children 0-18 years | 353 | Cross-sectional | Online open access link | Yes, PSI-SF | Parental Stress |  |
| (Dell'Utri et al., 2020) | Italy | Neonates | 5 | Reported cases | Clinical outcomes | No | Change in reported fetal deaths as compared to pre-covid records |  |
|  | U.K. | Mothers / children <1 year | 1329 | Cross-sectional | Online open access link to support groups in social media platforms | No | Mental health and coping |  |
| (Dib et al., 2020) |  |  |  |  |  |  |  |  |
| (Romero-Gonzalez et al., 2020) | Spain | Pregnant women | 131 | Cross-sectional | Undisclosed | Yes, SCL-90-R, PDQ, EEP-14, CD-RISC, and AIS | Anxiety and depression, prenatal distress, stress, resilience, insomnia |  |
| (Scala et al., 2020) | U.S. | Neonates | 25 REF/27 CO | Cross-sectional | Hospital (controlled with precovid records) | No | Care practices to preterm neonates pre/post-lockdown:family visitation frequency, developmental care practices |  |
| (R. Yang et al., 2020) | China | Neonates | 65 | Retrospective cohort | Municipal maternal health information database | No | Neonates born to covid-positive mothers' outcomes: preterm, abnormal chest image, COVID swab test, PROM, diarrhea, fever, asphyxia, death. |  |
| (Quandt et al., 2020) | U.S. | Mothers / children <8 | 57 REF/45 CO | Cross-sectional within ongoing prospective cohort | Ongoing cohort study | No | Rural immigrants families' wellbeing during lockdown: parental employment status and family financial security, childcare and education, healthcare, community attitudes towards discrimination |  |
| (Craig & Churchill, 2020) | Australia | Parents / children <18 | 1536 | Cross-sectional | National survey (controlled by precovid estimates) | No | Stratified by gender:change in employment status, time in paid and unpaid labor, subjective feelings about time allocation |  |
| (Kotabagi, Nauta, et al., 2020) | U.K. | Pregnant women | 14CA/14CO | Case-control | Antenatal clinics | Yes, GAD-7 and PHQ-9 | Anxiety and depression |  |
| (Kovler et al., 2020) | U.S.A | Children < 15 | 257 | Retrospective review | Medical records | No | Increase in physical child abuse injuries in pediatric trauma center |  |
| (Russell et al., 2020) | U.S.A | Caregivers / children 0-5/6-11/12-18 | 420 | Cross-sectional | Social media recruitment | Yes, BSFC-s, GAD-7, MDI and CPRS | Caregivers burden, anxiety, major depression, child-parent relationship scale, perceived child stress. |  |
| (Zanardo et al., 2020) | Italy | Postpartum women | 91 CA/101CO | Non-concurrent case-control | Hospital | Yes, EPDS | Depression |  |
| (Leeb et al., 2020) | U.S.A | Children 0-4/ 5-11 / 12-17 |  | Retrospective open cohort | Medical records | No | Change in national mental health-related emergency department visits pre/post covid |  |
| (Moyer, Sakyi, et al., 2020) | Ghana | Pregnant women | 71 | Cross-sectional | Online open access link via social media platform | No | Heightened anxiety, stigma of becoming infeceted by COVID, food and job insecurity, conflicts at home |  |
| (Bao et al., 2020) | U.S.A | Children 5-6 | 3657 | Prospective cohort (ECLS-K) | Multistage | No | Changes in reading ability |  |
| (Auðardóttir & Rúdólfsdóttir, 2020) | Iceland | Parents / children 0-18 | 97 | Cross-sectional, qualitative | Online open access link | No | Parenting styles during lockdown, discipline and acceptable ways of coping, societal pressures on good parenting |  |
| (Effati-Daryani et al., 2020) | Iran | Pregnant women | 205 | Cross-sectional | Cluster | Yes, DASS-21 | Anxiety and depression |  |
| (Zheng et al., 2020) | China | Pregnant women | 331 | Cross-sectional | Hospital | Yes, SQ, PSRS and PQEEPH | Sense of security, stress, Depression/neurastenia/fear/compulsion/hypocondria |  |
| (Freedman et al., 2020) | U.S.A | Pregnant women and their children <3months old | 43 | Prospective cohort | Hospital | , Yes, IBQ-R | Correlation between prenatal choline levels and impact of maternal COVID on fetus and ECD: clinical records + infant behavior at 3 months old |  |
| (Z. Liu et al., 2020) | China | Caregivers / children 4-10 | 1619 REF/ 436 CO | Cross-sectional | Schools (controlled by precovid records) | Yes, CSHQ | Children's sleep patterns/distruebances during lockdown |  |
| (Preis, Mahaffey, & Lobel, 2020) | U.S.A | Pregnant women | 4451 | Cross-sectional | Scale-validation study | Yes, PREPS | Psychometric properties of the Pandemic related pregnancy-stress scale |  |
| (Protudjer et al., 2020) | Canada | Mothers / children 0-8 | 580 | Mixed methods (cross-sectional + interviews) | Online open access link (controls sampled equally) | Yes, GAD-7 | Correlation of parental anxiety with children's food allergy during lockdown |  |
| (Tang et al., 2021) | China | Children 6-17 | 4391 | Cross-sectional | Schools | Yes, DASS-21 | Children's depression, anxiety and stress, life satisfaction,impact of home quarantine |  |
| (C. H. Liu, C. Erdei, et al., 2021) | U.S. | Perinatal women | 1123 | Cross-sectional | Snowballing and social media groups | Yes, CES-D, GAD-7 and PTSD | Maternal depression, anxiety |  |
| (Grumi et al., 2020) | Italy | Caregivers / children 1-15 | 86 | Cross-sectinal within ongoing prospective cohort study | Cohort subjects | Yes, PSI-SF, BDI-II, and STAI-Y | Mental health of caregivers of children with neurodevelopmental disabilities: stress,depression, anxiety, communication with therapists, concern for the child, COVID information, worry and management |  |
| (Limbers et al., 2020) | U.S. | Mothers / children <5 | 200 | Cross-sectional | Online open access link | Yes, PSS, and WHOQOL-BREF | Working mothers mental health - stress,quality of life, and physical activity. |  |
| (Tso et al., 2020) | China | Parents / children 2-12 | 12163(parents children 2-5)17029(parents children6-12) | Cross-sectional | Schools | Yes, CPCIS, SDQ, PEDS-QL, and PSS | Parent-child interaction, children's negative behavior, pediatric quality of life, parental stress |  |
| (Bender et al., 2020) | U.S. | Pregnant women | 318 | Qualitaive-cross-sectional | Hospital | No | Impact of COVID-positive test on hospitalized women: neglect, isolation, difficulty with neonatal separation |  |
| (Qi et al., 2020) | China | Pregnant women | 298 | Cross-sectional | Online open access link | Yes, PQEEPH, SSS and PSSS | Pregnant women's perception of threat and psychological impact: psy. status on crisis, somatic self-rating, perceived social support. |  |
| (Del Boca et al., 2020) | Italy | Mothers / children 0-15 | 350 | Qualitative- cross-sectional | undisclosed (controls similarly undisclosed) | No | Stratified by gender: dividion of childcare, housework and homeschooling |  |
| (Mizrak Sahin & Kabakci, 2020) | Turkey | Pregnant women | 15 | Cross-sectional-qualitative | Snowball | No | Pregnant women's outcomes: fear and knowledge of pandemic, disruption of prenatal care, routines and social lives, isolation, strategies for coping with anxiety |  |
| Miller, J. J., et al.^315^, 2020, U.S.A | U.S. | Parents / children 0-18 | 990 | Cross-sectional | State wide list of eligible subjects + Snowball | Yes, PSSF | Foster parents'stress, |  |
| (Sade et al., 2020) | Israel | Pregnant women | 90 REF/279 CO | Cross-sectional | Hospitals (contolled by precovid cohort) | Yes, EPDS | High-risk pregnancies and depression |  |
| (X. Liu et al., 2020) | China | Pregnant women | 1947 | Cross-sectional | Online open access link | Yes, SAS | Anxiety |  |
| (Salehi et al., 2020) | Iran | Pregnant women | 222 | Cross-sectional | Hospital | Yes, FCV-19S, CDAS, DASS-21 and PES-brief | Fear of COVID, COVID anxiety, depression anxiety and stress scale, pregnancy experience scale |  |
| (Mullins et al., 2021) | U.K./U.S | Neonates | 4005 | Reported cases | Medical records | No | Neonates born to covid-positive mothers' outcomes: gestational age at delivery, weight, intrauterine and neonatal death, delivery mode, covid test |  |
| (Kassaw & Pandey, 2020) | Ethiopia | Pregnant women | 178 | Cross-sectional | Hospital | Yes, GAD | Anxiety and predictors- rural area, social support, primigravida, education level |  |
| (de Sá et al., 2020) | Brazil | Parents / children 0-12 | 816 | Cross-sectional | Online open access link | No | Physical and intelectual activity, play, screen time |  |
| (Shinomiya et al., 2021) | Japan | Caregivers and children | 2312 | Cross-sectional study | "Research company" | No | Sleep behavior of infants and caregivers |  |
| (Metz et al., 2021) | U.S. | Pregnant women | 1291 | Retrospective cohort | Hospital | No | Neonates born to covid-positive mothers' outcomes: gestational age at delivery, weight, intrauterine and neonatal death, delivery mode, NICU admission |  |
| (Hailemariam et al., 2021) | Ethiopia | Pregnant women | 44 | Qualitative cross-sectional | Household public list | No | Anxiety about social isolation, perceived poor quality of care during pandemi, fear of infection in health facilities |  |
| (Lemon et al., 2021) | U.S. | Neonates | 5396 REF/17687 CON | Retrospective cohort | Medical records | No | Sought for reasons behind falling preterm birth rates by analysing stress during pregnancy, racially mediated impacts of the pandemic and impacts of provider care |  |
| (Ayas et al., 2020) | UAE | Parents / children with cochlear implants | 24 | Cross-sectional study | Convenience | No | Aims to understand the impact of COVID-19 on access to hearing healthcare services for children with cochlear implants |  |
| (Niela-Vilén et al., 2021) | Finland | Pregnant women | 38 | Cohort | Open access (social media advertisements) and clinics | Yes, EPDS and PRAQ-R2 | Weekly depression and pregnancy anxiety responses. Also analyzed stress levels through physiological data collected by smartwatch |  |
| (Kar et al., 2021) | Canada | Pregnant women | 7470 | Cross-sectional study | Online open access link | Yes, EPDS and PROMIS | Depression ; Anxiety . Participants were asked about their use of alcohol, cannabis, tobacco, and illicit drugs in the year before the current pregnancy/while pregnant. |  |
| (Mehdizadehkashi et al., 2021) | Iran | Pregnant women | 300 | Cross-sectional | Hospital | Yes, PSS and DASS | Perceived stress and anxiety |  |
| (Q. Wang, B. Song, et al., 2021) | China | Pregnant women | 6248 | Cross-sectional | Prenatal clinics | Yes, PHQ-9 and GAD-7 | Depression, anxiety, social support, isolation, intention to seek mental health care services |  |
| (Popofsky et al., 2020) | U.S. | Pregnant women | 85 | Cohort | Hospital | No | Less breastfeeding adherence in dyads who were separated in hospital due to COVID |  |
| (Neubauer et al., 2021) | Germany | Parents / children < 6-19 | 970 | Cohort | Online open access link | Yes, SDQ and SCS | Child behavior, familiy enviroment, parental stress, vitality, autonomy-supportive parenting, child well-being, parental need fulfillment. Parents of school children were asked repeatedly about their own experiences, their assessments of their child’s feelings and behaviors, and their perceptions of the family environment. |  |
| (Kallander et al., 2021) | Nigeria, Tanzania, Sierra Leone | Children 9-13y. | 51 | Qualitative cross-sectional | Household list | No | COVID impact on daily lives, domestic challenges, schooling, perception of social stability, religion and hope |  |
| (H. Jiang et al., 2021) | China | Pregnant women | 1873 | Cross-sectional | Online open access link | Yes, PSS, EPDS and SAS | Perceived stress, depression, anxiety |  |
| (Kachi et al., 2021) | England | Pregnant women | 359 | Cross-sectional | Online open access link | Yes, EPDS | Depression as a result of discrimination against pregnant women on the workplace |  |
| (Ademhan Tural et al., 2020) | Turkey | Children 4-18y. and parents | 113 REF/108 CO | Case-control | Hospital | Yes, GHQ-12, COPE and COX-PSY-CP | Impact of pandemic on parents/children with chronic lung disease. Parental mental health, parental coping strategies, children`s depression and anxiety |  |
| (Guo et al., 2021) | China, Italy, Netherlands | Mothers / children <10y. | 2463 | Cross-sectional | Online open access link | Yes, BSI-18 and COPE | Cross-cultural predictors of maternal mental health: depression and anxiety, family conflict and pandemic-related stress, father and gradparents support, resilience |  |
| (Yan et al., 2021) | China | Neonates | 15 REF/45 CO | Case-control | Health information system | No | Impact of COVID on positive neonates cognition/development: Hammersmith neonatal neurological exam, neuroimages, lung images and development |  |
| (Yirmiya et al., 2021) | Israel | Pregnant women | 1114REF/256 CO | Cross-sectional | Online open access link | Yes, PHQ-9 and PREPS | Depression, pandemic-related stress during pregnancy and predictors |  |
| (Lorentz et al., 2021) | Brazil | Postpartum women | 125 | Prospective cohort | Hospital | Yes, EPDS and FSFI | Postpartum depression and sexual disfunction |  |
| (Steinberg et al., 2021) | U.S. | Parents / children between 6 and 70 months old | 177 | Cross-sectional study | Online open access link | Yes, Music@ Home Scale, PAQ and Kessler6 | Parent-Child Musical Engagement Parent-Child Attachment Parental Distress |  |
| (Xie et al., 2021) | China | Pregnant women | 689 REF/2657 CON | Cohort | Online open access link and Hospital | Yes, SCL-90-R, PSQI and FES | Seeks to analyze how Pregnant woman were affected psychologically by the pandemic, w/ family well being as a mediator and sleep quality as an indirect indicator. Somatization, depression, anxiety, hostility, Pittsburgh Sleep Quality Index, and Family Environment Scale |  |
| (Dickerson et al., 2020) | U.K. | Parents / children 0-4yo OR 9-13yo | 2144 | Cross-sectional study | Convenience | Yes, PHQ-8 and GAD-7 | Depression Anxiety ; caregivers' impressions and mental health assessment over 1st lockdown |  |
| (Vasilevski et al., 2021) | Australia | Partners / pregnant women | 44 | Qualitative study | convenience | No | Experiences of the maternity service response, level of support provided, conflicting information and processes, approaches to partners and support persons, missing out, psychological impacts, benefits of COVID-19 restrictions |  |
| (Pearson et al., 2021) | 20 countries | Mothers | 2109 | Cross-sectional | Online open access link | No | Physician mothers`s concern about infecting their children, homeschooling, disrupted family life, financial dificulties |  |
| (Quenzer-Alfred et al., 2021) | Germany | Children 2-6 y. | 49 | Qualitative cross-sectional | Preschools | Yes, IDS-2 | Impact of nursery closure on ECD/school preparedness: Intelligence and development, math and language skills, |  |
| (Martínez Pérez et al., 2020) | Spain | Mothers / children 0-18y. | 185 | Cross-sectional | Specialized health care centers | No | As a result of IPV at home: children's emotional disregulation, emotional alteration, stress and trauma, somatization |  |
| (Al-Matary et al., 2021) | Saudi Arabia | Neonates | 288 | Case series | Medical records | No | Neonates born to covid-positive mothers' outcomes: gestational age at delivery, weight, intrauterine and neonatal death, Apgar 1m and 5m, COVID test |  |
| (Chen et al., 2021) | U.S. | Neonates | 92 | Case series | Medical records | No | NICU admission |  |
|  |  |  |  |  |  |  | IUGR 5-minute Apgar score < 7 Respiratory distress SARS-CoV-2 vertical transmission Fetal death from the studied population |  |
| (Mahmoud et al., 2021) | U.K. | Parents / children 3-17 y. | 384 | Cross-sectional | Online open access link | No | Complex model: parental stress/ anxiety and family healthy eating behaviors, moderated by gender |  |
| (C. H. Liu, L. Mittal, et al., 2021) | U.S. | Postpartum women | 63 REF/565 CO | Cross-sectional | Hospital | Yes, CES-D, GAD-7 and PCL-C | Mothers of neonates at NICU more at risk of developing depression, anxiety, PTSD |  |
| (TG et al., 2021) | Ethiopia | Pregnant women | 422 | Cross-sectional | Prenatal clinic | No | Association between fear of/knowledge about COVID and adherence to isolation measures |  |
| (Hcini et al., 2021) | French Guiana | Neonates | 108 | Prospective cohort | Hospital | No | Neonates born to covid-positive mothers' outcomes: COVID test, peripartum or neonatal death, NICU admission, respiratory distress, seizures, Apgar score and low birthweight |  |
| (Peng et al., 2020) | China | Postpartum women | 24 REF/21 CO | Prospective cohort | Household list | No | Lower rate of breastfeeding on covid-positive/suspected mothers |  |
| (Alhuzimi, 2021) | Saudi Arabia | Parents / children with ASD | 150 | Cross-sectional study | Clinics | Yes, PSI-SF and GHQ-12 | Studies changes in autistic behaviours and their impatcs on parents' psyche. Parental Distress and Emotional well being |  |
| (F. Wu et al., 2021) | China | Pregnant women | 3434 | Cross-sectional study | Hospital and clinic | Yes, GAD-7 and PHQ-9 | Anxiety and Depression . Seeks Prevalence and contributory factors by logistic regression analysis |  |
| (Ashini et al., 2021) | Libya | Parents / NICU newborns | 41 | Cross-sectional study | Hospitals | Yes, EPDS | Depression Evaluates how visitation restrictions affected parents of NB |  |
| (Wdowiak et al., 2021) | Poland | Pregnant women | 280 | Cross-sectional study | Clinics | Yes, BDI | Depression, Body Mass Index |  |
| (Mariño-Narvaez et al., 2021) | Spain | Mothers | 75 REF/82 CON | Cross-sectional study | Clinics | Yes, S-BSS-R and EPDS | Birth Satisfaction Depression |  |
| (Chrzan-Dętkoś et al., 2021) | Poland | Postpartum women | 139 | Cross-sectional | Prenatal services | Yes, EPDS | Depression |  |
| (Mangolian Shahrbabaki et al., 2021) | Iran | Children 7-11 y. | 340 | Cross-sectional | Schools | Yes, CDAS | Children`s pandemic-related anxiety and COVID fear, association with being a single child and maternal education level |  |
| (Fernandes et al., 2021) | Portugal | Postpartum women | 567 | Cross-sectional | Online open access link | Yes, HDAS, PSS, IM-P and PBQ | Maternal anxiety and depression, stress, mindful parenting, postpartum bonding |  |
| (Muldoon et al., 2021) | Canada | Postpartum women | 216 | Cross-sectional | Hospital | Yes, WHO questionnaire-25% | Perinatal IPV and risk factors |  |
| (Naghizadeh et al., 2021) | Iran | Pregnant women | 250 | Cross-sectional | Prenatal clinic | Yes, WHO questionnaire-25% | IPV correlation with quality of life |  |
| (Sbrilli et al., 2021) | U.S. | Perinatal women | 199 | Cross-sectional | Online open acess link | Yes, BSI-18, FFMQ-SF AND IUS-12 | Psychological symptoms - anxiety, stress and somatization, mindfulness, intolerance of uncertainty |  |
| (Puertas-Gonzalez et al., 2021) | Spain | Pregnant women | 100 REF/100 CON | Cross-sectional study | Hospital and open access survey | Yes, SCL-90-R, PSS, PDQ and AIS | Depression, Anxiety, Stress Pregnancy Stress, Athens Insomnia Scale |  |
| (Maharlouei et al., 2021) | Iran | Pregnant women | 540 | cross-sectional study | Clinics | Yes, DASS-21 | Depression, Anxiety, Stress |  |
| (Horiuchi et al., 2020) | Japan | Parents / 3–14yo | 1200 | cross-sectional study | Online open access link | Yes, K6 | Depression and anxiety, asked if children had sleep, appetite, physical and mental conditions, activity, or behavior problems |  |
| (J. Wang, Y. Li, et al., 2021) | China | Children 6-13yo | 123 535 | cross-sectional study | Convenience | No | Myopic shift was noted after home confinement due to coronavirus disease 2019 for children aged 6 to 8 years. |  |
| (Berard et al., 2021) | France | Parents / 2-21yo ASD children | 239 | Cross-sectional study | Hospital | Yes, CSS, BECS, PEP-3, WPPSI-IV, WISC-V, WAIS-IV and K-ABC | Children’s behavior during the pandemic regarding nutrition, sleep, challenging |  |
|  |  |  |  |  |  |  | behaviors, communicative abilities, and stereotyped behavior. Children's behavior severity child's intellectual functioning |  |
| (Milan & Dáu, 2021) | U.S. | Parents / 3-18yo | 240 | cross-sectional study | Online open access link | Yes, PCL-5 and PHQ-9 | Posttraumatic Symptoms, Depression, child vaccine intentions/COVID vaccine intentions |  |
| (Özkan Şat & Yaman Sözbir, 2021) | Turkey | Pregnant women | 376 | Cross-sectional study | Online open access link | Yes, TPDS | Pregnancy distress |  |
| (Ferrante et al., 2021) | U.S. | Parents / children 4-8yo | 1000 | Cross-sectional study | Online open access link | No | Examined parent-reported impacts of COVID-19 on lifestyle and current family food acquisition and eating behaviors |  |
| (Valero-Moreno et al., 2021) | Spain | Parents / adolescents | 94 | Cohort | Online open access link | Yes, DASS-21, MOOD, SCL and CD-RISC | Anxiety, depression and stress, moods, somatization and resilience. |  |
| (Lebel et al., 2020) | Canada | Pregnant women | 1987 | Cross-sectional | Snowball and online open access link | Yes, PROMIS, EPDS, SSEQ | Maternal anxiety, depression and social support |  |
| (Matsushima & Horiguchi, 2020) | Japan | Pregnant women | 1777 | Cross-sectional | Online platform for pregnant women | Yes, EPDS | Post-natal depression |  |
| (Kahyaoglu Sut & Kucukkaya, 2020) | Turkey | Pregnant women | 403 | Cross-sectional | Online open access link | Yes, HADS | Maternal anxiety and depression |  |
| (Wang et al., 2020) | China | Mothers | 72 | Longitudinal | Single-arm cohort | Yes, PCL-C, EPDS, ASQ-3, ASQ:SE-2 | Association between dyad separation and child neurobehavioral development at 3 months old |  |
| (Pariente et al., 2020) | Israel | Mothers | 223 REF/123 CON | Cross-sectional | Hospital | Yes, EPDS | Depression and delivery during pandemic |  |
| (Medina-Jimenez et al., 2020) | Mexico | 549 | Pregnant women | Cross-sectional | Hospital | Yes, EPDS and PSS | Depression and stress |  |
|  |  |  |  |  |  |  |  |  |
| (Mappa et al., 2020) | Italy | Pregnant women | 178 | Cross-sectional | Prenatal clinics | Yes, STAI | Anxiety, fear of infection's consequences |  |
| (Ayaz et al., 2020) | Turkey | Pregnant women | 63 | Prospective cohort | Prenatal clinics | Yes, IDAS-II, BAI | Depression and anxiety |  |
| (Cusinato et al., 2020) | Italy | Parents of children 5-17yo | 463 | Cross-sectional | Online open access link | Yes, PGWB, SDQ, PSS, CYRM-R | Parental Psychological Wellbeing |  |
| (Hui et al., 2020) | China | Mothers | 4357 | Retrospective cohort | Hospital | Yes, EPDS | Post-partum depression after pandemic alert |  |
| (Chung et al., 2020) | Singapore | Parents of children <1y.o. | 258 | Cross-sectional | Online open access link | Yes, CIQ, PSS | COVID financial, psychological impact, Parental stress |  |
| (Nodoushan et al., 2020) | Iran | Pregnant women | 560 | Cross-sectional | Hospital | Yes, DASS-21, Sheerer | Spiritual health; Stress, anxiety and depression; Self-efficacy |  |
| (Silverman et al., 2020) | U.S. | Pregnant women | 488 | Cross-sectional | Hospital | Yes, EPDS | Post-partum depression |  |
|  |  |  |  |  |  |  |  |  |
| (Evans et al., 2020) | Australia | Parents of children <18y.o. | 2130 | Qualitative | Online open access link | No | Overall impacts of COVID on family |  |
| (Hamadani et al., 2020) | Bangladesh | Mothers of children <1y.o. | 2424 | Interrupted time series | Ongoing RCT | Yes, GAD-7 | Anxiety |  |
| (Spinelli et al., 2020) | Italy | Parents of children 2-14y.o. | 854 | Cross-sectional | Online open access link | Yes, DASS, PSI, SDQ | Parents stress |  |
| (Ng et al., 2020) | Singapore | Pregnant women | 324 | Cross-sectional | Prenatal clinics | Yes, DASS | Depression, anxiety and stress |  |
| (Durankuş & Aksu, 2020) | Turkey | Pregnant women | 260 | Cross-sectional | Hospital | Yes, EPDS, BDI, BAI | Depression and anxiety |  |
| (Sinaci et al., 2020) | Turkey | Pregnant women | 446 | Cross-sectional | Hospital | Yes, STAI-T, BAI | Anxiety |  |
| (Zanardo et al., 2021) | Italy | Mothers | 163 REF 154 CON | Non-concurrent case-control | Prenatal clinics | Yes, EPDS | Breastfeeding, depression |  |
| (Mazza et al., 2020) | Italy | Parents of children<18yo | 1180 | Cross-sectional | Online open access link | Yes, BFI-10, GHQ | Parents personality-neuroticism |  |
| (Malkawi et al., 2020) | Jordan | Mothers | 2013 | Cross-sectional | Online open access link | Yes, DASS | Depression, Anxiety, Stress |  |
| (J. Yue et al., 2020) | China | Children (mean 10y.o.)+ parents | 1360 | Cross-sectional | Online open access link | Yes, SAS, CES-DC SDS | Anxiety depression and PTSD |  |
| (Yuan et al., 2020) | China | Parents | 50 REF/ 50 CON | Cross-sectional | Hospital | Yes, HAD, VADS | Anxiety and Depression |  |
| (Lin et al., 2020) | China | Pregnant women | 751 | Cross-sectional | Online open access link | Yes, SAS, PHQ-9 | Anxiety and depression |  |
| (C. Yue et al., 2020) | China | Pregnant women | 308 | Cross-sectional | Prenatal clinics | Yes,SAS, SSRS | Anxiety, Social support |  |
| (Dong et al., 2020) | China | Pregnant women | 156 i | Cross-sectional | Online open access link | Yes, SAS, SDS | Anxiety and Depression |  |
| (Overbeck et al., 2021) | Denmark | Pregnant women | 257 | Cross-sectional | Prenatal clinics | No | Fear of COVID and isolation |  |
| (Shayganfard et al., 2020) | Iran | Pregnant and post-partum women | 103 | Cross-sectional | Prenatal clinics | Yes, HAI, EPDS, PSS | Anxiety, depression and Stress |  |
| (Parra-Saavedra et al., 2020) | Colombia | Pregnant women | 946 | Cross-sectional | Online open access link | No | COVID-19 knowledge, attitudes and psychological symptoms |  |
| (Nanjundaswamy et al., 2020) | India | Obstetricians | 118 | Cross-sectional | Online open access link | No | COVID-19 related concerns and anxiety on pregnant patients |  |
| (Zhou et al., 2020) | China | Pregnant/non-pregnant women | 859 | Cross-sectional | Hospital | Yes, PHQ-9, GAD-7, SCL-90, ISI | Depression, Anxiety, Somatic symptoms, Insomnia |  |
| (Yerkes et al., 2020) | Netherlands | Parents-employed, child<18 yo | 868 | Cross-sectional | Online open access link | No | Paid work, division of childcare and household tasks by gender, quality of life |  |
| (Suzuki, 2020a) | Japan |  | 117 REF/134 CON | Cross-sectional | Hospital | No | Anxiety at 8-12 weeks of gestation |  |
| (Rhodes et al., 2020) | U.K. | Pregnant women and parents children < 2y.o. | 244 | Cross-sectional | Health system app users | No | Anxiety, Stress, Disruption, Support levels |  |
| (Corbett et al., 2020) | Ireland | Pregnant women | 71 | Cross-sectional | Prenatal clinics | No | Worry, behavioural changes |  |
| (Thayer & Gildner, 2020) | U.S. | Pregnant women | 2099 | Cross-sectional | online open access link | Yes, EPDS | Financial stress, Depression |  |
| (Taubman-Ben-Ari, Chasson, Abu Sharkia, et al., 2020) | Israel | Pregnant women | 403 | Cross-sectional | online open access link | Yes, SCF-SF, MPSS | Anxiety, Self-Compassion, Social Support |  |
| (Romero et al., 2020) | Spain | Parents and children 3-12 | 1049 parents provided data on 1049 children 3-12y.o. | Cross-sectional | online open access link | Yes, CD0RISC-10, PHQ-4, SDQ | Parental resilience |  |
| (Molgora & Accordini, 2020) | Italy | Pregnant women | 575 | Cross-sectional | online open access link | Yes, STAI-Y, EPDS, WDEQ-A/B, PPQ | Anxiety and Depression |  |
| (Chivers et al., 2020) | Australia | Postpartum women | Unknown | Qualitative | online open access link | No | Distress, dispair, grief due to altered support relationships |  |
| (H. Yang et al., 2020) | China | Postpartum women and neonates | 21 women/23 neonates | Retrospective cohort study | hospital | No | Clinical features of mothers, and obstetrical outcomes (Apgar, weight, COVID PCR) |  |
| (Taubman-Ben-Ari, Chasson, & Abu-Sharkia, 2020) | Israel | Pregnant women | 336 | Cross-sectional | online open access link | No | COVID related distress and anxiety |  |
| (Ceulemans et al., 2020) | Belgium | Pregnant and postpartum | 5866 | Cross-sectional | undisclosed | Yes, EPDS, GAD-7 | Depression and Anxiety |  |
| (Marchetti, Fontanesi, Mazza, et al., 2020) | Italy | Parents | 2173 | Cross-sectional | online open access link | Yes, PBI, PAPF | Emotional exhaustion, Resilience and connections |  |
| (Yu et al., 2020) | China | Pregnant, post-partum and neonates | Unknown | Retrospective cohort | hospital | No | Neonatal outcomes: weight, Apgar (1 and 5 minutes), complications |  |
| (Chasson et al., 2020) | Israel | Pregnant | 403 | Cross-sectional | online open access link | Yes, BRS, MPSS, MHI-5 | Resilience, Social support, Distress |  |
| (Wu et al., 2020) | China | Pregnant women | 4124 | Cross-sectional | hospital | Yes, EPDS | Depression |  |
| (Yildirim & Eslen-Ziya, 2020) | France, Germany, Italy, Norway, Sweden, Turkey, U.K. and the United States | Academics | 460 | Cross-sectional | qualitative | No | Impact of homeoffice on work, routines and contribution to housework and childcare |  |
| (Farrell et al., 2020) | Qatar | Postpartum | 288 | Cross-sectional | hospital | Yes, PHQ-9, GAD-7 | Anxiety and depression |  |
| (Yassa et al., 2020) | Turkey | Pregnant women | 203 REF/101 CON | Prospective case-control | hospital | Yes, STAI, MOCI | Anxiety and OCD |  |
| (Berthelot et al., 2020) | Canada | Pregnant women | 1258 REF/496 CO | Cross-sectional | online open access link | Yes, (K10, PANAS) | Distress, Positive and negative affect, PTSD |  |
| (Zreik et al., 2020) | Israel | Mothers to children 6-72 months | 264 | Cross-sectional | online open access link | Yes, (ISI) | Insomnia, Anxiety, Child Sleep |  |
| (Zhang & Ma, 2020) | China | Pregnant women | 560 | Cross-sectional | online open access link | Yes, IES, MHLSS | Impact of event, Family and social support |  |
| (Gur et al., 2020) | U.S. | Pregnant women | 216 REF/571CON | Cross-sectional | hospital | Yes, GAD-7, PHQ-2 | Anxiety and depression |  |
| (Hu et al., 2020) | China | Neonates | 6 | Retrospective cohort study | hospital | No | Preterm neonates- covid-19, sepsis, head ultrasound abnormality, patent ductus arteriosus, BPD, NEC, prolonged jaundice caused by cholestasis, and retinopathy of prematurity. |  |
| (Campagnaro et al., 2020) | Brazil | Parents of children <12 | 385 | Cross-sectional | online open access link | No | Changes in food intake- choices, portion size- and in care seeking behavior regarding parents and children oral health due to fear of COVID |  |
| (L. Zeng et al., 2020) | China | Neonates | 33 | Prospective cohort study | hospital | No | Neonates born to covid-positive mothers. Main outcomes: preterm, small-for-gestational age, Asphyxia, fever, pneumonia, respiratory syndrome distress, shortness of breath, cyanosis, feeding intolerance, white blood cell, llymphocyte count, creatine kinase isoenzimes, alanine aminotransferase, mechanical ventilation, antibiotic, duration of intensive care, death. |  |
| (Shockley et al., 2020) | U.S. | Parents to children < 6 | 334 | Qualitative | online open access link | No | Emergent themes:marital tension, sleep quantity, job performance, distress |  |
| (Naurin et al., 2020) | Sweden | Pregnant women | 6941 | Part of larger longitudinal (1 year- 3 time points) | prospective cohort | No | COVID and overrall health related worries (regarding self, child, and partner) |  |
| (Ahorsu et al., 2020) | Iran | Pregnant women and husbands | 290 | Cross-sectional | national health system database | Yes, HADS, SF-12, PHQ-9 | Fear of covid and preventive behavior, Hospital anxiety and depression |  |
| (Cameron et al., 2020) | Canada | Pregnant women and mothers of children < 8 | 641 | Cross-sectional | social media platforms | Yes, RSE, RDAS, MSPSS, PASS, GAD-7, EPDS, CESD-R | Maternal use of mental health services and distress |  |
| (Aksoy Derya et al., 2020) | Turkey | Pregnant women | 48 REF/48 CON | Prospective cohort study | attendees of public Prenatal education class | Yes, Nu-PDQ, PRAQ-R2 | Effect of tele-education intervention on prenatal distress, anxiety |  |
| (Garcia de Avila et al., 2020) | Brazil | Children and caregivers | 289 | Cross-sectional | online open access link | Yes, CAQ, NRS | Children's anxiety |  |
| (Peltz et al., 2020) | U.S. | Parents of childrend 5-11 | 1003 | Cross-sectional | online open access link | Yes, MPFI | Stress related to health, finances, work and parenting |  |
| (Brown et al., 2020) | U.S. | Parents of childrend <18 | 183 | Cross-sectional | educational settings and family agencies | Yes, PSS-10, CAP | Parental: perceived stress and child abuse |  |
| (Carroll et al., 2020) | Canada | Parents to children 18 months-5years | 254 | Cross-sectional within longitudinal | prospective cohort study | No | Change in health behaviors, for parents and children (diet, physical activity, screen time sleep) stress, food insecurity, |  |
| (Brandt et al., 2020) | U.S. | Neonates | 61 | Non-concurrent matched case-control | hospital | No | Neonates born to covid positive mothers' outcomes: lenght of stay, antepartum admission, cesarian delivery, festational age at birth, Chorioamnionitis, Venous thromboembolism, Persistent category 2 fetal heart rate tracing, Preeclampsia, Intrauterine fetal demise, birthweight, Apgar, NICU admission and lenght of stay, Respiratory distress syndrome, Intraventricular hemorrhage, Necrotizing enterocolitis, death. |  |
| (Milne et al., 2020) | Ireland | Pregnant women | 70 | Cross-sectional within ongoing propective cohort study | hospital | No | Mood, relationship tension and deterioriation |  |
| (Shafer et al., 2020) | Canada | Parent to children <18 | 1245 | Cross-sectional | online open access link | No | Housework and childcare task sharing, changes in fathers' housework and childcare, gender |  |
| (Sama et al., 2021) | India | Parents to children age undisclosed | 310 | Cross-sectional | online open access link | No | Children's mental health and behavior: depression, anxiety, anger, irritation, diet, weight, sleep, screen time, fights, socialdevelopment |  |
| (Farghaly et al., 2020) | U.S. | Neonates | 79 | Retrospective cohort study (at birth and 2 week follow-up) | hospital | No | Neonates born to covid positive mothers- outcomes: isolation, brestfeeding, NICU admission, respiratory distress, desaturation, seizures, poor feeding, feverabnormal chest x-ray, mechanical ventilation, CPAP, oxygen therapy |  |
| (Daks et al., 2020) | U.S. | Parents | 742 | Cross-sectional | online open access link | Yes, PHQ-9, MPFI, CHAOS, FAD, APQ, PPQ, CBCL | Psychological flexibility, depression, stress, family discord |  |
| (Ostacoli et al., 2020) | Italy | Postpartum women | 163 | Cross-sectional | sampling: hospital | Yes, EPDS, IES-R | Depression, Impact of event relationship and PTSD |  |
| (X. Zeng et al., 2020) | China | Postpartum women | 625 | Cross-sectional | hospital | Yes, | Anxiety, depression, insomnia, quality of life |  |
| (Oncel et al., 2020) | Turkey | Neonates | 125 | Multicenter prospective cohort study | hospital | No | Clinical outcomes of neonates born to covid positive mothers: gestational age, birthweight, mode of delivery, apgar at 1 and 5minutes, resuscitation, respiratory support, breastfeeding, NICO admission, maternal death, COVID swab, full blood count |  |
| (Moore et al., 2020) | Canada | Parents to children 5-17 | 1472 | Cross-sectional | national census | No | Movement and play behavior |  |
| (Morelli et al., 2020) | Italy | Parents to children 6-13 | 277 | Cross-sectional | online open access link | Yes, PSS, ERC | Distress, Children's emotion regulation |  |
| (Preis, Mahaffey, et al., 2020b) | U.S. | Pregnant women | 4450 | Cross-sectional | online open access link | Yes, PREPS | Pandemic-related pregnancy stress |  |
| (X. Li et al., 2020) | China | Pregnant women | 45 | Cross-sectional | online open access link | Yes, PHQ-9, GAD-7, ISI, IES-7 | Depression, anxiety, insomnia, distress |  |
| (Moyer, Compton, et al., 2020) | U.S. | Pregnant women | 2740 | Cross-sectional | online open access link | No | Pregnancy-related anxiety |  |
| (Preis, Mahaffey, et al., 2020a) | U.S. | Pregnant women | 788 | Cross-sectional | online open access link | Yes, PREPS | Pandemic-related pregnancy stress |  |
| (Gassman-Pines et al., 2020) | U.S. | Parents of children 2-7 | 561 | Prospective cohort | hospital | No | Parent and child psychological wellbeing, financial hardship |  |
| (Pierce-Williams et al., 2020) | U.S. | Neonates | 33 | Retrospective and prospective cohort | hospital | No | Neonatal outcomes: gestational age and weight at birth, NICU admission, Apgar at 5m, death |  |
| (Patabendige et al., 2020) | Sri Lanka | Pregnant women | 257 | Cross-sectional | hospital | Yes, | Anxiety and depression (HADS) |  |
| (M. Li et al., 2020) | China | Neonates | 3407 | Cross-sectional | hospital | No | Neonatal outcomes: gestational age and weight at birth, asphyxia |  |
| (Pınar Senkalfa et al., 2020) | Turkey | Parents + Children 9-18 | 45 REF/90 CON | Case-control | hospital | Yes, STAI | Anxiety |  |
| (Oskovi-Kaplan et al., 2020) | Turkey | Postpartum | 223 | Cross-sectional | hospital | Yes, EPDS, MAI | Depression and attachment |  |
| (López-Morales et al., 2020) | Argentina | Pregnant women | 102 REF/102 CON | Prospective cohort | hospital | Yes, BDI-II, STAI, PANAS | Depression, anxiety, positive and negative affect |  |
| (Liang et al., 2020) | China | Postpartum | 864 | Cross-sectional | online open access link | Yes, EPDS | Depression |  |
| (Ravaldi, Ricca, et al., 2020) | Italy | Pregnant women | 737 | Cross-sectional | snowball | Yes, NSESS, STAI-Y | PTSD, anxiety |  |
| (Dagklis et al., 2020) | Greece | Pregnant women | 269 | Cross-sectional | Prenatal clinics | Yes, STAI, EPDS | Anxiety, depression |  |
| (Vazquez-Vazquez et al., 2021) | U.K. | Postpartum | 1365 | Cross-sectional | snowball | No | Impact of COVID on household health, finances, work, birth experience, infant feeding and behavior, mothers activities, moods, access to support. |  |
| (Alsuhaibani & Alaqeel, 2020) | Saudi Arabia | Parents of children 0-2 | 749 | Cross-sectional | online open access link | No | Children's immunization history before/during COVID, barriers towards timely immunization, parents' attitude towards delayed immunization |  |
| (Günther-Bel et al., 2020) | Spain | Parents of children 0-18 | 167 | Mixed methods(cross-sectional + qualitative) | online open access link | Yes, CERFB | Conjugal, parental and coparental functioning |  |
| (Ravaldi, Wilson, et al., 2020) | Italy | Pregnant women | 200 | Mixed methods (cross-sectional+qualitative) | convenience | No | Covid related anxiety and stress in pregnancy |  |
| (H. Chen et al., 2020) | China | Neonates | 9 | Retrospective review | hospital | No | Outcomes of neonates born to covid positive mothers: age and weight at birth, Apgar at 1 and 5m, asphyxia, death |  |
| (Neece et al., 2020) | U.S. | Parents of children 3-5 | 77 | Qualitative study within ongoing RCT | ongoing RCT | No | Impact of lockdown on parental practices concerning children with developmental and intellectual disabilities |  |
| (Suzuki, 2020b) | Japan | Postpartum women | 148 REF/132CON | Cross-sectional | hospital | Yes, EPDS, MIBS-J | Depression, mother-infant bonding |  |
| (Shahid et al., 2020) | Pakistan | Pregnant women | 552 | Cross-sectional | hospital | Yes, EPDS, K-10 | Depression, pandemic-related psychological impact |  |
| (Lemieux et al., 2020) | Canada | Pregnant women | 1014 | Cross-sectional | online open access link | Yes, EPDS, PANAS | News media consumption and depression, PTSD |  |
| (Lawson et al., 2020) | U.S. | Parents of children 4-10 | 342 | Cross-sectional | online open access link | Yes, CTSPC, F-COPES, CES-D | Family financial stability, children's psychological maltreatment and child abuse |  |
| (Xu et al., 2020) | U.S. | Grandparents to children 0-19 | 362 | Cross-sectional | online open access link | No, CTS-PC, MIH-5 | Psychological aggression, corporal punisment, and neglect, grandparents' mental health |  |
| (Hjálmsdóttir & Bjarnadóttir, 2021) | Iceland | Mothers to children 0-18 | 37 | Qualitative (diary records- 2 weeks) | online open access link | No | Gendered division of household chores and childcare |  |
| (Shawna J Lee et al., 2021) | U.S. | Parents of children < 12 | 654 | Cross-sectional | online open access link | Yes, FFCWS, PHQ-8 | Child anxiety and behavior changes, parental depression |  |
| (Farewell et al., 2020) | U.S. | Pregnant and postpartum | 31 | Mixed methods (qualitative + cross-sectional) | online open access link | Yes, PHQ-2, BRS | Depression and resilience |  |
| (Ergenekon et al., 2020) | Turkey | Mothers of children 0-15 | 21 REF/32 CON | Cross-sectional | hospital | Yes, BDI, STAI-S/T | Maternal depression, anxiety |  |
| (Mayeur et al., 2020) | France | Pregnant women | 104 | Retrospective cohort | hospital | No | Anxiety and interruption of work |  |
| (Patrick et al., 2020) | U.S. | Parents of children < 18 | 1011 | Cross-sectional | online research panel based on random sampling of US households | No | Family changes in health status, insurance and access to health and child care, food security, parental mental health and children's behavior |  |
| (Odeh et al., 2020) | Jordan | Parents of children 0-18 | 340 | Cross-sectional | hospital | No | Parental worry over wellbeing of children with type-1 diabetes during lockdown |  |
| (Mirlashari et al., 2021) | Iran | Parents and children | 5 children/13 parents | Qualitative | hospital | No | Experience of children with cancer during lockdown- quality of care, adaptation and emotional impact |  |
| (Hocaoglu et al., 2020) | Turkey | Pregnant women | 283 | Cross-sectional | Prenatal clinics | Yes, STAI, IES-R | Anxiety |  |
| (Khamees et al., 2021) | Kuwait | Pregnant women | 120 | Cross-sectional | Prenatal clinics | Yes, EPDS, KUAS | Depression, Anxiety |  |
| (Mousavi, 2020) | Iran | Parents | 213 | Cross-sectional | Open online access link | Yes, KMMS, PBA, WHO-10 | Marital satisfaction, parental burnout, psychological well-being |  |
| (Aronu et al., 2020) | Nigeria | Mothers | 404 | Cross-sectional | Systematic random sampling | No | Maternal perception and preferences about send their children back to school |  |
| (Tchimtchoua Tamo, 2020) | China | Mothers | 274 | Cross-sectional | Snowball | Yes, PSI-SF | Parental Stress |  |
| (Farsi & Farsi, 2021) | U.S. | Parents | 206 | Cohort | Sampling convenience | Yes, CES-D, CRS, SDQ, PBI | Depression, Anxiety, Children behavior, Parenting |  |
| (Ferns et al., 2021) | U.K. | Parents | 242 | Cross-sectional | Sampling convenience | No | Gender disperities |  |
| (Kawamura et al., 2021) | Japan | Pregnant women and HCW | 584 | Cross-sectional | Hospital | No | Anxiety/ COVID fear pregnant women |  |
|  |  |  |  |  |  |  |  |  |
| (Martins-Filho et al., 2020) | Brazil | Children | 123 | Cohort | Criminal statistics data | No | Rates of child abuse pre to post outbreak |  |
| (Bradfield et al., 2021) | Australia | Parents | 3701 | Cross-sectional | Open online access link | No | Maternity care during the covid-19 |  |
| (Kinser et al., 2021) | U.S. | Pregnant and post-partum women | 524 | Cross-sectional | Open online access link | Yes, BSI, PCL-5, CD-RISC 2 | Depression, anxiety and resilience |  |
| (Çakmak & Öztürk, 2021) | Turkey | Parents | 162 | Cross-sectional | Hospital | Yes, PSI-SF, Coronavirus Anxiety Scale | Parental Stress and Anxiety |  |
| (Fumagalli et al., 2021) | Italy | Post-partum women | 34 | Qualitative study | Hospital | No | Childbearing experiences of covid-19 positive mothers. |  |
| (Fallon et al., 2021) | U.K. | Post-partum women | 614 | Cross-sectional | Open online access link | Yes, EPDS, STAI, PSAS-RSF-C, PSOC, MSPSS, MIBS | Prevalence rates and risk factors for clinically relevant depression and anxiety |  |
| (Peng et al., 2021) | China | Post-partum women | 71 | Cross-sectional | Hospital | Yes, MPAS, SAS, SDS | Mental health |  |
| (Marchetti, Fontanesi, Di Giandomenico, et al., 2020) | Italy | Parents | 878 | Cross-sectional | Snowball | Yes, GHQ-12, PSDQ | Effect of parents psychological distress and verbal aggression on behavioral and emotional symptoms of children during covid |  |
| (Akgor et al., 2021) | Turkey | Pregnant women | 297 | Cross-sectional | Hospital | Yes, HaDS-D, HADS-A | Depression and Anxiety |  |
| (Wolf et al., 2021) | U.S. | Parents | 329 | Cross-sectional | Snowball | Yes, DD, PSS | Parental behavior and stress |  |
| (Bentenuto et al., 2021) | Italy | Parents | 164 | Cross-sectional | Snowball | Yes, PSS, CRS, SDQ | Parental stress, coparenting and child adjustment |  |
| (Rice & Williams, 2021) | Canada | Post-partum women | 57 | Qualitative study | Snowball | No | Mental health, breatfeeding |  |
| (S. D. Wang et al., 2021) | U.S. | Mothers | 197 | Cross-sectional | Open online access link | Yes, FPSQ, TFEQ-R18 | Child feeding practices |  |
| (Roos et al., 2021) | Canada | Parents | 656 | Cross-sectional | Open online access link | Yes, GAD-7, PASS, CESD, CESD-R, EPDS, RDAS, PARYC, OS, PSI-SF | Parental practices and mental health |  |
| (Litmanovitz et al., 2021) | 22 countries | Parents | 96 | Cross-sectional | Open online access link | No | Parental practices during pandemic |  |
| (Cooper et al., 2021) | U.S. | Fathers | 466 | Cross-sectional | Open online access link | Yes, PROMIS, CEFIS | Fathers mental health |  |
| (Sahithya et al., 2020) | India | Parents | 196 | Cross-sectional | Open online access link | Yes, PSS | Parental mental health |  |
| (Buonsenso et al., 2020) | Sierra Leone | Children | 785 | Cohort | Medical records | No | Malaria diagnoses rates in children during COVID 19 |  |
| (An et al., 2021) | China | Post-partum women | 209 | Cross-sectional | Sampling convenience | Yes, EPDS, CPSS | Parental mental health |  |
| (Cohodes et al., 2021) | U.S. | Parents | 247 | Cross-sectional | Open online access link | Yes, CBCL, EPII, ERPSST, PRTQ, BAI, PSI-4, | Parents and children mental health |  |
| (Khamees et al., 2021) | Egypt | Pregnant women | 120 | Cross-sectional | Hospital | Yes, KUAS, EPDS | Anxiety and depression in pregnant women |  |
| (Dulfe et al., 2021) | Spain | Pregnant women | 204 | Cohort | Hospital | Yes, EPDS, STAI, MOS-SSS | Depression, anxiety and social support pregnant women |  |
| (Camerlink et al., 2021) | 28 countries | Caregivers | 117 | Cross-sectional | Open online access link | Yes, PSS, ISSB | Caregivers stress and social support |  |
| (Linos et al., 2021) | U.S. | Mothers | 1809 | Cross-sectional | Open online access link | Yes, GAD-7 | Anxiety |  |
| (Scarpellini et al., 2021) | Italy | Mothers | 2149 | Cross-sectional | Open online access link | No | Homeschooling, ECD domain and parental practices |  |
| (Halley et al., 2021) | U.S. | Mothers | 1806 | Cross-sectional + qualitative study | Open online access link | No | Maternal mental health |  |
| (Manja et al., 2020) | Malaysia | Parents | 158 | Cross-sectional | Open online access link | No | Parental mental health |  |
| (Faccioli et al., 2021) | Italy | Parents | 292 | Cross-sectional | Open online access link | No | Parental mental health |  |
| (C. H. Liu, C. Erdei, et al., 2021) | China | Mothers | 125 | Cohort | Prenatal clinics | Yes, SAS, SDS, PSI-SF, HHI | Maternal mental health |  |
| (Khoury et al., 2021) | Canada | Pregnant women | 303 | Cross-sectional | Open online access link | Yes, CWS, CES-D, ISI | Maternal mental health |  |
| (Kimura et al., 2021) | Japan | Mothers | 4700 | Cohort | Open online access link | Yes, K6 | Maternal mental health |  |
| (Preis, Mahaffey, & Lobel, 2021) | U.S. | Pregnant women | 3896 | Cohort | Open online access link | Yes, FBS, BBS, PREPS | Maternal mental health |  |
| (Mo et al., 2021) | Iran | Pregnant women | 19 | Cross-sectional | Qualitative Study | No | Maternal mental health |  |
|  |  |  |  |  |  |  |  |  |
| (Rogers et al., 2021) | U.S. | Caregivers | 8 | Cross-sectional | Open online access link | Yes, PREPS, GAD-7 | Maternal mental health |  |
|  |  |  |  |  |  |  |  |  |
|  |  |  |  | Qualitative Study |  |  |  |  |
| (Preis, Mahaffey, Pati, et al., 2021) | U.S. | Pregnant women | 1367 | Cohort | Open online access link | Yes, PREPS, GAD-7 | Maternal mental health and neonatal outcomes |  |
| (Jansen et al., 2021) | U.S. | Parents | 318 | Cross-sectional | Open online access link | Yes, CFPQ, PFSQ, FPSQ, P-SNAQ | Stress and feeding practices |  |
| (Handayani & Dina, 2021) | Indonesia | Pregnant women | 66 | Cross-sectional | Not clear | No | Maternal mental health |  |
|  |  |  |  |  |  |  |  |  |
|  |  |  |  | Mixed method |  |  |  |  |
|  |  |  |  | Qualitative study |  |  |  |  |
| (Fisher et al., 2021) | U.S. | Parents | 92 | Cross-sectional | Hospital | Yes, ECHO, CEFIS | Caregivers mental health |  |
| (Del Río et al., 2021) | Spain | Mothers | 490 | Cross-sectional | Hospital | No | Breastfeeding during pandemic |  |
| (Orsini et al., 2021) | Italy | Parents | 96 | Cross-sectional | Hospital | Yes, | Caregivers mental health |  |
|  |  |  |  |  |  | IES-R, GAD-7, PHQ-9 |  |  |
| (Cellini et al., 2021) | Italy | Mothers | 299 | Cross-sectional | Open online access link | Yes, PSQUI, STQ, SDQ, DERS, SDQ-P | Children’s mental health and ECD |  |
| (Dumbre et al., 2020) | India | Parents | 60 | Cross-sectional | Open online access link | No | Children mental health/ ECD |  |
| (Prikhidko et al., 2020) | U.S.. | Parents | 155 | Cross- sectional | Open online access link | Yes, CTS, PBA, DASS-21, ERQ | Parents mental health |  |
| (Green et al., 2021) | U.K. | Parents | 14376 | Cohort | Stratified clustered sampling | Yes, SDQ | Parental practices and ECD domain |  |
| (Brisca et al., 2021) | Italy | Children | 236 | Cohort | Hospital | No | Child health, ECD domain, health (pediatric) assess |  |
| (Effati-Daryani et al., 2020) | Iran | Pregnant women | 437 | Cross-sectional | Prenatal clinics | Yes, DASS-21, FSFI | Mental health and sexual function |  |
| (Basu et al., 2021) | 64 countries | Pregnant and post-partum women | 6894 | Cross-sectional | Open online access link | Yes, PHQ-4, IES-6, UCLA-3 | Mental health |  |
| (Talbot et al., 2021) | 13 countries | Pregnant women | 51 | Cohort | Tweets thematic analysis | No | Maternal mental health |  |
| (Kracht et al., 2021) | U.S. | Mothers | 1721 | Cross-sectional | Open online access link | Yes, CHAOS | Maternal mental health |  |
| (J. Wang, Y. Zhou, et al., 2021) | China | Pregnant and post-partum women | 2235 | Cross-sectional | Hospital | Yes, GAD, PHQ-9, ISI | Maternal mental health |  |
| (Sakalidis et al., 2021) | Australia, New Zealand | Post-partum women | 364 | Cross-sectional | Open online access link | Yes, IFPS II, PSS, BISQ, FAD, MHCSF | Maternal mental health, breastfeeding and parental practices |  |
| (Wilke et al., 2020) | 14 countries | NGO’s leader | 67 | Cross-sectional | Snowball | No | Parental practices and family dynamics |  |
| (Giurge et al., 2021) | 5 countries | Parents | 31141 | Cross-sectional | Open online access link | No | Maternal mental health |  |
| (Janevic et al., 2021) | U.S. | Perinatal women | 237 | Cross-sectional | Hospital | Yes, BSSR, DMS, GAD-7, PHQ-2, PSS, SCID, DSM-5 | Maternal mental health |  |
| (Perzow et al., 2021) | U.S. | Pregnant and post-partum women | 135 | Cross-sectional | Ongoing RCT | Yes, EPDS, STAI-SF, RULS-8, NIH coronavirus HIS, INR | Maternal mental health |  |
| (Wong et al., 2021) | China | Parents | 600 | Cross-sectional | Random | Yes, AAS, CTSPC | Child maltreatment, ECD domain, parental practices and caregiver mental health |  |
| (Ribeiro et al., 2021) | Brazil | Caregivers | 188 | Cross-sectional | Snowball | Yes, PSS, WHO-5, | Parental practices, caregiver mental health |  |
| (S. Q. Chen et al., 2020) | China | Parents | 1450 | Cross-sectional | Prenatal clinics | Yes, Selfdesigned, GHQ-12, PSS, PSI-SF-15 | Mental health |  |
| (Zhou et al., 2021) | China | Pregnant women | 1266 | Cross-sectional | Prenatal clinics | Yes, PHQ-9, SSRS | Maternal mental health |  |
| (Takaku & Yokoyama, 2021) | Japan | Mothers | 22553 | Cross-sectional | Random | Yes, QMI | Family dinamics |  |
| (Chaves et al., 2021) | Spain | Pregnant and post-partum women | 724 | Cross-sectional | Snowball | Yes, EPDS, PANAS, SWLS | Maternal mental health |  |
| (Rosen et al., 2021) | Israel | Neonates | 55 | Cohort | Hospital | No | ECD domain |  |
| (Di Riso et al., 2021) | Italy | Mothers | 45 | Cross-sectional | Snowball | Yes, GHQ-12, MAC-RF, SDQ, SCAS-SAD | Caregivers mental health |  |
| (Ceulemans et al., 2021) | 5 countries | Pregnant and post-partum women | 9041 | Cross-sectional | Open online access link | Yes, EPDS, GAD-7, PSS | Maternal mental health |  |
| (Cheng et al., 2021) | U.K. | Parents | 6795 | Cohort | Open online access link | Yes, GHQ | Parental practices, Caregivers mental health |  |
| (Masters et al., 2021) | U.S. | Pregnant women | 183 | Cross-sectional | Ongoing RCT | Yes, EPDS, GAD-7, PTSD, PCL-C, BACE | Maternal mental health |  |
| (Connell & Strambler, 2021) | U.S. | Parents | 2068 | Cross-sectional | Open online access link | Yes, PC-CTS, MNBS | Mental health and parental practices |  |
| (Korukcu et al., 2021) | Turkey | Pregnant women | 497 | Cross-sectional | Open online access link | Yes, EPDS | Maternal mental health |  |
| (Latorre et al., 2021) | Italy | Mother-baby | 204 | Cohort | Hospital | No | Parental practices, breastfeeding. |  |
| (C. J. P. Zhang et al., 2021) | China | Pregnant women | 1901 | Cross-sectional | Hospital and open online access link | Yes, EPDS, PTSD | Maternal mental health |  |
| (Cui et al., 2021) | China | Parent-child pair | 867 | Cohort | Open online access link | No | Homeschooling and ECD domain |  |
| (Poulain et al., 2021) | Germany | Parents (1-10yo) | 285 | Cohort (longitudinal) | Open online access link | No | Homechooling, parental practices, family dinamics |  |
| (Celik et al., 2021) | Turkey | Mothers | 116 | Cross-sectional | Hospital | Yes, BAI | Maternal mental health |  |
| (Nassar et al., 2021) | Egypt | Mothers (9-11yo) | 37 | Cross-sectional | Convenience | Yes, CSHQ, HRQoL, DASS-21 | Maternal mental health |  |
| (Stallard et al., 2021) | Portugal, U.K. | Caregivers (6-16yo) | 385 | Cross-sectional | Open online access link | Yes, GAD-7, WEMWBS | Caregiver mental health |  |
| (C. Li et al., 2021) | China | Prenatal and post-natal women | 2201 | Cross-sectional | Convenience | Yes, PHQ-9, ISI, GAD-7 | Maternal mental health - depression |  |
| (Liang et al., 2021) | Italy | Parents (3-18yo) | 838 | Cross-sectional | Snowball | Yes, RQ | Parental practices, caregivers mental health, child mental health, ECD domain |  |
| (Daulay, 2021) | Indonesia | Mothers of ASD (6-8yo) | 1462 | Cross-sectional | Convenience | No | Maternal mental health, homeschooling, child behavior, ECD domain |  |
|  |  |  |  | Qualitative study |  |  |  |  |
| (Sweet et al., 2021) | Australia | Mothers | 27 | Cross-sectional | Open online access link | No | Maternal mental health |  |
|  |  |  |  | Qualitative study |  |  |  |  |
| (Ares et al., 2021) | Uruguay | Parents | 1725 | Cross-sectional | Convenience | No | Parental practices |  |
|  |  |  |  | Mixed-methods (most qualitative) |  |  |  |  |
| (Ueda et al., 2021) | Japan | Caregivers (6-18yo) | 136 | Cross-sectional | Convenience | Yes, STAI, CBCL, CES-D, PSI, WHOQOL-BREF, KINDL | Caregivers and children mental health |  |
| (Ren et al., 2020) | China | Parents (pre-school aged) | 1451 | Cross-sectional | Open online access link | Yes, S-AI, PSI-SF-15, NEO-FFI, MSPSS, PMBP, CMBP | Parental stress and social support |  |
| (Waller et al., 2021) | U.S. | Parents (3-10yo) | 303 | Cross-sectional | Open online access link | Yes, CU, ICU, SDQ, APQ, Parent scale | Parental practices |  |
| (Moscardino et al., 2021) | Italy | Parents | 89 | Cross-sectional | Open online access link | Yes, LTE-Q, SEPTI, FAD, PSS | Parental stress |  |
|  |  |  |  |  |  |  |  |  |
| (Ehsan & Jahan, 2021) | Bangladesh | Mothers | 223 | Cross-sectional | Open online access link | No | Family impact, social support, mothers mental health |  |
|  |  |  |  | Qualitative study |  |  |  |  |
| (Wimberly et al., 2021) | U.S. | Caregivers | 321 | Cross-sectional | Hospital | No | Caregivers mental health |  |
|  |  |  |  |  |  |  |  |  |
| (Quílez-Robres et al., 2021) | Spain | Parents | 145 | Cross-sectional | Snowball | Yes, BDI-II, EQ, BAI | Caregivers mental health, anxiety and empathy |  |
| (Mayopoulos et al., 2021) | U.S. | Mothers | 1161 | Cohort | Open online access link | Yes, PDI, CB-PTSD, MIBS, MAI | Maternal mental health |  |
| (Vaterlaus et al., 2021) | U.S. | Parents (<18yo) | 365 | Cross-sectional | Snowball | No | Family dinamics |  |
|  |  |  |  | Qualitative study |  |  |  |  |
| (Rocha et al., 2021) | Brazil | Mother-child pairs | 577 | Cohort | “previous subjects” enrolled via phone | Yes, EBIA, MMD, SRQ | Maternal mental health and food insecurity |  |
| (Preuss et al., 2021) | Germany and Austria | Parents | 285 | Cross-sectional | Open online access link | No | Caregivers mental health and parental practices |  |
| (King et al., 2021) | U.S. | Pregnant women | 725 | Cohort | Convenience | Yes, EPDS | Maternal mental health |  |
| (Calvano et al., 2021) | Germany | Parents | 1024 | Cross-sectional | Dual-frame | Yes, pediMACE, PHQ-4, PHQ-D, Parental Stress Scale | Parents and children mental health, ECD domain |  |
| (Bin-Nun et al., 2021) | Israel | Mothers | 86 | Cross-sectional | Convenience | Yes, MHI, NSS-8, PSS-NICU | Maternal mental health |  |
| (McDonald et al., 2020) | U.K. | Children | 69568 | Cross-sectional | Eletronic records | No | Parental practices, vaccination rates |  |
| (Nurrizka et al., 2021) | N.Z. | Parents (4-5yo) | 362 | Cross-sectional | Open online access link | Yes, ASI, PSDQ, IMS, PCRQ | Maternal mental health |  |
| (Miller et al., 2020) | U.S. | Parents | 281 | Cross-sectional | Convenience | No | Gender disparities on childcare, Maternal mental health |  |
| (Gildner et al., 2020) | U.S. | Pregnant women | 1856 | Cross-sectional | Open online access link | Yes, EPDS | Maternal mental health, financial problems, depression |  |
| (Wilder et al., 2021) | U.S. | Parents | 12 | Cross-sectional | Convenience | No | Caregivers mental health |  |
|  |  |  |  | Qualitative analysis |  |  |  |  |
| (Silverman et al., 2020) | U.S. | Postpartum women | 516 | Cohort | Hospital | Yes, EPDS | Maternal mental health |  |
| (Q. Wang, P. K. H. Mo, et al., 2021) | China | Pregnant women | 15428 | Cross-sectional | Convenience | Yes, PHQ-9, GAD-7 | Maternal mental health |  |
|  |  |  |  |  |  |  |  |  |
| (Ogamba et al., 2021) | U.S. | Pregnant women | 40 | Cohort | Hospital | No | Neonatal outcomes |  |
| (Pope et al., 2021) | 3 countries | Pregnant women | 537 | Cross-sectional | Convenience | Yes, PSS-4, NuPQ, SF-12, PHBS, MSPSS | Maternal mental health |  |
|  |  |  |  |  |  |  |  |  |
| (Ollivier et al., 2021) | Canada | Parents | 68 | Cross-sectional | Convenience | No | Maternal mental health |  |
|  |  |  |  | Qualitative study |  |  |  |  |
| (Freisthler et al., 2021) | U.S. | Parents | 322 | Cross-sectional | Open online access link | No | Caregivers mental health |  |
| (Sheridan et al., 2021) | U.S. | Children |  | Cross-sectional | Prenatal clinics | No | Pediatric mental health, ECD domain |  |
| (Katayama et al., 2021) | Japan | Children 0-19y | 1240 | Cohort (retrospective) | Prefecture records |  | Epidemiological information |  |
| (Forbes et al., 2021) | U.S. | Parents and children 0-18y | 1048 | Cross-sectional | Random | Yes, IPAQ | Parental practices |  |
| (Harrison et al., 2021) | U.K. | Postpartum women | 251 | Cross-sectional | Online focus groups | Yes, EPDS, PASS, RTQ-10, MSPSS | Maternal depression, Anxiety, Negative thinking and Social support. |  |
| (Glynn et al., 2021) | U.S. | Parents | 48 | Cross-sectional, qualitative | Online focus groups |  | Child screen-time during covid-19 |  |
| (Barbosa-Leiker et al., 2021) | U.S. | Perinatal women | 162 | Cross-sectional, quantitative and qualitative | Online focus groups | Yes, ISEL | Social support and Maternal mental health |  |
| (Abedzadeh-Kalahroudi et al., 2021) | Iran | Pregnant women | 56 (REF)/ 96(CON) | Cohort | Hospital |  | Neonatal outcomes: fetal distress, prematurity, Apgar |  |
| (Siegle et al., 2020) | Brazil | Caregivers | 816 | Cross-sectional | Online open access link |  | ECD domain |  |
| (Nguyen et al., 2021) | India | Mothers | 587 | Cohort | Random |  | Food insecurity and Parental practices |  |
| (Chapman et al., 2021) | U.K. | Perinatal women |  | Cross-sectional |  |  | Google trends analysis on perinatal mental health diagnoses, risk factors, red flags and suggested admission criteria during |  |
| (Cacioppo et al., 2021) | France | Parents | 1000 | Cross-sectional | Online open access link |  | Parental mental health, child wellbeing ECD domain |  |
| (Dellagiulia et al., 2020) | Italy | Mothers | 37 | Cohort | Preschools |  | Sleep, ECD domain |  |
| (Nicholson et al., 2020) | Ireland | Parents and children 0-16m | 1044 | Cross-sectional | Convenience |  | Pediatric health care access, ECD domain |  |
| (Aronu et al., 2020) | Nigeria | Mothers | 387 | Cross-sectioal | Convenience |  | Parental practices, mask use. |  |
| (Boekhorst et al., 2021) | Netherlands | Perinatal women | 669 | Cohort | Hospital | Yes, EPDS, TPDS-NA | Depression, Stress, Mental health |  |
|  |  |  |  |  |  |  |  |  |
| (Stojanov et al., 2020) | Serbia | Mothers and children 0-2y | 108 (REF)/ 87 (CON) | Cross-sectional | Online open access link | Yes, EPDS | Depression |  |
| (Colizzi et al., 2020) | Italy | Parents | 527 | Cross-sectional | Online open access link |  | Children's mental health. Autism spectrum disorders and Parental practices |  |
| (Upendra et al., 2020) | India | Mothers | 4 | Cross-sectional, qualitative |  |  | Maternal mental health |  |
|  |  |  |  |  |  |  |  |  |
| (Adadms et al., 2020) | 2021 | Parents and children 5-18y | 433 | Cross-sectional | Snowball | Yes, PSS | Stress and Parental mental health |  |
| (Fosco et al., 2021) | U.S. | Parents | 204 | Cross-sectional |  |  | Internalizing and externalizing behaviors, family ECD, Parenting quality and Parent emotional distress |  |
| (DeYoung & Mangum, 2021) | U.S. | Perinatal women | 116 | Cross-sectional, quantitative and qualitative | Online focus groups | Yes, PCL, SWLS | Traumatic experience and Well being |  |
| (Suárez-Rico et al., 2021) | México | Postpartum women | 293 | Cross-sectional | Online open access link | Yes, EPDS, T-STAI, PSS-10 | Maternal mental health: Anxiety, Depression, Stress |  |
| (Adadms et al., 2020) | Canada | Postpartum women | 433 | Cross-sectional | Online open access link | Yes, PSS | Stress |  |
| (Choi et al., 2021) | South Korea | Parents and children 9-10y | 166 | Cohort | Convenience |  | Children's mental health, Parental practices |  |
| (Koyucu & Karaca, 2021) | Turkey | Pregnant woman | 729 | Cross-sectional | Online open access link | Yes, DASS, MSPSS | Depression, Social support and Maternal mental health |  |
| (Guruge et al., 2021) | Canada | Parents | 50 | Cross-sectional, qualitative | Convenience |  | Parents mental health, Parental practices |  |
| (Costoya et al., 2021) | Argentina | Parents | 961 | Cross-sectional | Online open access link |  | Family dynamics |  |
|  |  |  |  |  |  |  |  |  |
| (Syed Anwar Aly et al., 2021) | Malaysia | Pregnant women | 415 | Cross-sectional | Online open access link |  | Maternity care experience, Maternal anxiety |  |
| (W. Li et al., 2021) | China | Parents | 21526 | Cross-sectional | Online open access link | Yes, SDQ | Mental health, Education level, Parental practices, ECD domain |  |
| (Ding et al., 2021) | China | Pregnant women | 817 | Cross-sectional | Online open access link | Yes, SAS | Anxiety |  |
| (Tambling et al., 2021) | U.S. | Parents | 210 | Cross-sectional, qualitative | Convenience |  | Parental practices |  |
| (Asai et al., 2021) | Japan | Pregnant women | 292 | Cross-sectional | Convenience | Yes, FCV-19S | Fear of COVID |  |
| (Rodriguez et al., 2021) | U.S. | Parents | 405 | Cross-sectional | Online open access link | Yes, CTSPC | Parental practices, ECD domain |  |
| (Salmi et al., 2021) | Finland | Caregivers and children 0-15y | 20 | Cohort | Hospital |  | ECD domain, pediatric access to healthcare |  |
| (Sahin & Ozturk, 2021) | Turkey | Pregnant women | 240 | Cross-sectional | Online open access link | Yes, STAI | Anxiety |  |
| (Y. Zhang et al., 2021) | China | Pregnant women | 1794 | Cross-sectional | Hospital | Yes, SAS | Anxiety |  |
| (Saadati et al., 2021) | Iran | Pregnant women | 300 | Cross-sectional | Online open access link | Yes, HAQ | Anxiety |  |
| (Ge et al., 2021) | China | Pregnant women | 446 | Cross-sectional | Online open access link | Yes, SRAS | Anxiety |  |
| (Ronchi et al., 2021) | Italy | Neonates | 62 | Cohort | Hospital |  | Risk of transmission of SARS-CoV-2 from infected mothers to their neonates |  |
| (Pearson et al., 2021) | U.S. | Mothers | 2709 | Cross-sectional | Snowball |  | Personal and professional experiences of physician mothers during pandemic and the impact in their lives |  |
| (Zilver et al., 2021) | 2021 | Pregnant women | 1466 | Cohort | Online open access link | Yes, HADS, PSS-10 | Depression, Anxiety and Stress |  |
| (Achterberg et al., 2021) | Netherlands | Parents and children 10-13y | 257 | Cohort | Medical records | Yes, BSI | Depression, Anxiety, Hostility and Interpersonal sensitivity and Stress |  |
|  |  |  |  |  |  |  |  |  |
| (Aimen et al., 2020) | Pakistan | Children 1-16y | 74 | Cross-sectional | Hospital |  | Epidemiological, clinical, severity and early outcomes of COVID-19 in children |  |
| (Alaya et al., 2021) | Ireland | Perinatal women | 38 | Cohort | Hospital | Yes, SF-12 | Quality of life and hospital quality of care |  |
| (Aydin & Aktaş, 2021) | Turkey | Pregnant women | 14 | Cross-sectional, qualitative | Hospital |  | Pregnancy experiences in pandemic of COVID-19 |  |
| (Bo et al., 2021) | China | Pregnant women | 1309 | Cross-sectional | Hospital | Yes, PHQ | Depression |  |
| (Cao et al., 2021) | China | Children 12-18y | 11681 | Cross-sectional | School | Yes, 9-PHQ, GAD7, CTQ CDRS | Depression, Anxiety, Psychologic traumas, Resilience |  |
| (Chan & Fung, 2021) | China | Parents | 129 | Cross-sectional | Online open access link | Yes, 9-PHQ, GAD-7 | Depression, Anxiety and Stress |  |
| (Cho & Ilari, 2021) | U.S. | Parents | 19 | Cross-sectional, qualitative | Convenience |  | How parents used recorded music in their everyday lives during the pandemic |  |
| (Çolak et al., 2021) | Turkey | Pregnant women | 149 | Cross-sectional | Hospital | Yes, BDI, BAI, PSQI | Depression, Anxiety, Sleep quality |  |
| (Conti et al., 2021) | Italy | Pregnant women | 37 | Cross-sectional | Hospital |  | Effects of the early separation of the maternal-infantil dyad, in case of maternal SARS-CoV-2 infection |  |
| (Cui et al., 2020) | China | Children | 33 hospitals | Cross-sectional | Hospital |  | Editorial discussing mental health services for children in China during pandemic |  |
| (Dhiman et al., 2020) | India | Caregivers | 264 | Cross-sectional | Online open access link |  | Mental health status and changes in perceived strain among caregivers during pandemic outbreak |  |
| (Dule et al., 2021) | Ethiopia | Pregnant women | 384 | Cross-sectional | Convenience | Yes, WHO MSPSSFCoV-19S | Quality of life, Social support, Fear of Covid-19 scale |  |
| (El-Osta et al., 2021) | U.K. | Parents | 1214 | Cross-sectional | Online open access link | Yes, UCLATILS and DMOL | Impact of Covid-19 lockdown on feelings of loneliness and social isolation |  |
| (J. Liu et al., 2021) | U.S. | Pregnant women | 715 | Cross-sectional | Online open access link | Yes, EPDS, GADS | Depression, Anxiety |  |
| (Maggs et al., 2021) | U.S. | Parents and adolescents | 1367 | Cohort |  |  | Levels and predictors of US parents who newly allowed adolescents to drink alcohol at home during the shutdown |  |
| (Farsi & Farsi, 2021) | Saudi Arabia | Mothers | 833 | Cross-sectional | Snowball |  | Knowledge of COVID-19 and attitudes and fears about dental visits during the pandemic |  |
| (Gadermann et al., 2021) | Canada | Parents | 3000 | Cross-sectional | Online open access link |  | Mental health impacts of the COVID-19 pandemic on families with children |  |
| (Mangiavacchi et al., 2021) | Italy | Mothers | 3352 | Cross-sectional | Online open access link |  | Effect of lockdown on children's use of time, their emotional status and home learning |  |
| (Hamzehgardeshi et al., 2021) | Iran | Pregnant women | 318 | Cross-sectional | Online open access link | Yes, EPDS, PRAQ, CDAQ | Depression, Anxiety |  |
| (Hiiragi et al., 2021) | Japan | Postpartum women | 279 (REF)/ 339 (CON) | Cross-sectional | Hospital | Yes, EPDS | Depression |  |
| (Huebener et al., 2021) | Germany | Parents | 14781 | Cross-sectional | Random |  | General life satisfaction |  |
| (Hussong et al., 2021) | U.S. | Parents and children 6-16y | 105 | Cohort | Snowball | Yes, PSC, CBCL, CGAS | Children psychological problems, Behavior, Global Assessment |  |
| (Jani et al., 2021) | U.S. | Pregnant women | 34 | Cross-sectional | Hospital |  | Covid-19 infected mothers and infant's health outcomes and d placental pathology |  |
| (Jelly et al., 2021) | India | Pregnant women | 333 | Cross-sectional | Snowball | Yes, IES-R, GAD-7 | Impact of Event-Revised and Anxiety |  |
| (Y. Jiang et al., 2021) | China | Parents and grandparents | 171 | Cross-sectional | Convenience | Yes, CES-D | Depression |  |
| (Kumari et al., 2021) | India | Pregnant women | 25 | Cross-sectional, qualitative | Convenience |  | Impact of COVID-19 on mental health |  |
| (Markovic et al., 2021) | Germany | Children 0-71m | 781 | Cohort | Online open access link | Yes, BISQ, CSHQ | Sleep Quality and Sleep Habits |  |
| (S. J. Lee et al., 2021) | U.S. | Parents and children 0-12y | 555 | Cross-sectional | Convenience | Yes, CTS-PC, PHQ-8 | Parental practices and Parental depression |  |
| (Mayopoulos et al., 2020) | U.S. | Mothers and neonates | 68 (REF)/ 68 (CON) | Cross-sectional | Convenience | Yes, PDI | Peritraumatic Distress |  |
| (Mazza et al., 2021) | Italy | Parents and children 3-13y | 917 | Cross-sectional | Convenience | Yes, BFI-10, SDQ-P, GHQ-12 | Personality characteristics, Hyperactivity, Non-psychotic psychiatric conditions |  |
| (McFarland et al., 2021) | U.S. | Postpartum women | 4526 (REF)/ 18569 (CON) | Cross-sectional | Vital Statistics records | Yes, EPDS | Depression |  |
| (Meaney et al., 2021) | Ireland | Pregnant women | 573 | Cross-sectional | Online open access link | Yes, QPFQ, MSPSS | Prenatal care satisfaction, Social support |  |
| (Mirzaei et al., 2021) | Iran | Pregnant women | 403 (REF)/ 201 (CON) | Cross-sectional | Convenience | Yes, HADS, FSFI, SF-12 | Anxiety and depression, Female sexual function Quality of life (SF-12) |  |
| (Zamarro & Prados, 2021) | Italy | Parents | 89 | Cross-sectional | Convenience | Yes, LTE-Q, SEPTI, FAD, PSS | Stressful event, Self-Efficacy for Parenting, Family functioning, Stress |  |
| (Mortazavi et al., 2021) | Iran | Pregnant women | 484 | Cross-sectional | Convenience | Yes, CWS, WHO-5 | Worry, well-being |  |
| (Mumbardó-Adam et al., 2021) | Spain | Parents | 47 | Cross-sectional | Convenience |  | Quarantine routine |  |
| (Muniraman et al., 2020) | U.S. | Parents | 231 | Cross-sectional | Snowball |  | Parental perceptions of the impact of restricted visiting policies to neonatal intensive care units during the COVID-19 pandemic |  |
| (Myers & Emmott, 2021) | England | Mothers | 162 | Cross-sectional, qualitative | Online open access link | Yes, EPDS | Depression |  |
| (Nastro et al., 2020) | Italy | Children 1-18y | 71 | Cross-sectional | Convenience | Yes, QL 4.0, Promis anxiety questionnaires | Quality of life and Anxiety |  |
| (Yavaş Çelik, 2021) | Turkey | Parents | 26 | Cross-sectional, qualitative | Snowball |  | Experience of parents with coronavirus disease which demanded they separate from their children |  |
| (Nomura et al., 2021) | U.S. | Parents | 7 | Cross-sectional, qualitative | Convenience |  | Practices concerning COVID-19 |  |
| (Norman et al., 2021) | Sweden | Pregnant women | 2323 | Cohort | Hospital |  | Gestational age, APGAR, Survival in days, COVID positive |  |
| (Overbeck et al., 2021) | Denmark | Pregnant woman | 330 (REF)/ 1428 (CON) | Cross-sectional | Ongoing cohort | Yes, MDI and ASS | Mental well-being and Anxiety |  |
| (Pasca et al., 2021) | Italy | Children | 23 | Prospective cohort | Hospital | Yes, CBCL and PSI-SF | Behaviour and Stress |  |
| (Paschke et al., 2021) | Germany | Parents and children 10-17y | 824 | Cohort | Random | Yes, Procrastination Questionnaire for Students, PSS, Parental Self-efficacy Questionnaire | Procrastination, Children’s Stress, Parents Stress, Parents Self-efficacy |  |
| (Perez et al., 2021) | England | Parents | 590 | Cross-sectional, qualitative | Online open access link |  | Mood and parenting confidence |  |
| (Petrocchi et al., 2020) | Italy | Mothers | 144 | Cross-sectional | Online open access link |  | Psychological impact |  |
|  |  |  |  |  |  |  |  |  |
| (Philippe et al., 2021) | France | Children 3-18y | 498 | Cross-sectional | Online open access link | Yes, CEBQ, HomeSTEAD, BMI | Eating behavior, Physical activity and screen time, Nutritional status |  |
| (Yang et al., 2021) | China | Pregnant women | 19515 | Cross-sectional | Online open access link | Yes, PHQ-9, GAD-7, CERQ-short | Depression, Anxiety, Maladaptive cognition related to COVID-19 |  |
| (Racine et al., 2021) | Canada | Pregnant women | 2445 | Cohort | Random | Yes, CESD-10, SSTAI-short | Depression, Anxiety |  |
| (Ravens-Sieberer et al., 2020) | Germany | Children 11-17y | 1040 | Cohort | Online open access link | Yes, KIDSCREEN-10 index, SDQ, SCARED, CES-D | Health-related quality of life, Mental health problems, Generalized anxiety, Depression |  |
| (Raviv et al., 2021) | 2021 | Caregivers | 32217 | Cross-sectional | Online open access link | Yes, CEFIS | COVID-19 Exposure and Family Impact, Psychological Well-being |  |
|  |  |  |  |  |  |  |  |  |
| (Recto & Lesser, 2020) | U.S. | Fathers | 17 | Cross-sectional, qualitative | Convenience |  | COVID-19 pandemic impact on their lives |  |
| (Yaman et al., 2021) | Turkey | Mothers and neonates | 28 | Cohort | Hospital |  | Clinical characteristics and clinical evolution of newborns to mothers diagnosed with COVID-19 |  |
| (Rudrum, 2021) | Canada | Pregnant women | 56 | Cross-sectional, qualitative | Convenience |  | Experiences of pregnant people during the early stages of the pandemic |  |
| (Shah et al., 2021) | India | Children | 41 | Cross-sectional | Convenience |  | Problematic behaviors of child during the lockdown |  |
| (Shangguan et al., 2021) | China | Pregnant women | 2120 | Cross-sectional | Online open access link | Yes, GAD-7, PSS | Anxiety, Stress |  |
| (Shreffler et al., 2021) | U.S. | Pregnant women | 101 | Cohort | Hospital | Yes, ACEq | Adverse Childhood Experiences |  |
| (Shrestha et al., 2021) | Nepal | Pregnant women | 273 | Cross-sectional | Hospital | Yes, HAM-A | Anxiety |  |
| (Smith et al., 2021) | U.S. | Pregnant women | 83 | Cross-sectional | Online open access link | Yes, EPDS | Depression |  |
| (Solís-García et al., 2021) | Spain | Pregnant women | 73 | Cohort | Hospital |  | Epidemiological and clinical data |  |
| (Spinola et al., 2020) | Italy | Mothers | 243 | Cross-sectional | Online open access link | Yes, MSSS, Brief COPE, PSS, EPDS | Social Support, Coping, Stress, Depression |  |
| (Stepowicz et al., 2020) | Poland | Pregnant and postpartum women | 210 | Cross-sectional | Hospital | Yes, STAI, PSS-10 | Anxiety, Stress |  |
| (Suffren et al., 2021) | Canada | Parents and children 9-12y | 144 | Cross-sectional | Online open access link |  | COVID-19 Fears and Concerns and COVID-19 Changes and Health Issues |  |
| (Taubman-Ben-Ari & Ben-Yaakov, 2020) | Israel | Parents | 606 | Cross-sectional | Online open access link | Yes, PSI-SF, ECRS-SF, Self-Mastery Scale | Parental Stress, Attachment style, COVID-19-related anxiety |  |
|  |  |  |  |  |  |  |  |  |
| (Taubman-Ben-Ari et al., 2021) | Israel | Parents | 606 (REF)/ 985 (CON) | Cross-sectional | Online open access link | Yes, PSI-SF, Relationship Assessment Scale | Stress, Marital satisfaction |  |
| (Thompson & Bardone-Cone, 2021) | U.S. | Postpartum women | 232 (REF)/ 137 (CON) | Cross-sectional | Online open access link | Yes, CES-D, DASS-21, EAT-26, PSS | Depression, Anxiety, Eating disorders, Stress |  |
| (Vigod et al., 2021) | Canada | Postpartum women | 137609 | Cross-sectional | Medical records |  | Outpatient physician visit for mental illness to a primary care provider or to a psychiatrist |  |
| (Wheeler et al., 2021) | U.S. | Pregnant women | 33 | Cross-sectional | Convenience | Yes, PSS, PCI | Stress, Prenatal Coping |  |
| (Q. Wu et al., 2021) | U.S. | Grandparents and caregivers | 234 | Cross-sectional | Online focus groups | Yes, BRS | Resilience, Stress |  |
| (Xu et al., 2021) | China | Pregnant women | 274 | Cross-sectional | Online open access link | Yes, EPDS, SAS, PSS, PSQI | Depression, Anxiety, Stress, Sleep Quality |  |
| (Xue et al., 2021) | Switzerland | Caregivers | 53 | Cohort | Hospital | Yes, TOPSE, EPDS, CSI, SF12 | Parenting self-efficacy, Depression, Couple satisfaction, Health-related quality of life |  |
| (Xue & McMunn, 2021) | U.K. | Parents | 25690 | Cross-sectional | Ongoing cohort | Yes, GHQ | Gender differences in unpaid care work and psychological distresses |  |
| (Yamamura & Tsustsui, 2021) | Japan | Parents | 11867 | Cross-sectional | Random |  | Presence of children influences on parents’ work at home |  |

**References**

Abedzadeh-Kalahroudi, M., Sehat, M., Vahedpour, Z., & Talebian, P. (2021). Maternal and neonatal outcomes of pregnant patients with COVID-19: A prospective cohort study. *International Journal of Gynaecology and Obstetrics*, *153*(3), 449-456. <https://doi.org/10.1002/ijgo.13661>

Achterberg, M., Dobbelaar, S., Boer, O. D., & Crone, E. A. (2021). Perceived stress as mediator for longitudinal effects of the COVID-19 lockdown on wellbeing of parents and children. *Scientific Reports*, *11*(1), 2971. <https://doi.org/10.1038/s41598-021-81720-8>

Adadms, E. L., Smith, D., Caccavale, L. J., & Bean, M. K. (2020). Parents are stressed! Patterns of parent stress across COVID-19. *Res Sq*. <https://doi.org/10.21203/rs.3.rs-66730/v2>

Ademhan Tural, D., Emiralioglu, N., Tural Hesapcioglu, S., Karahan, S., Ozsezen, B., Sunman, B., . . . Kiper, N. (2020). Psychiatric and general health effects of COVID-19 pandemic on children with chronic lung disease and parents' coping styles. *Pediatric Pulmonology*, *55*(12), 3579-3586. <https://doi.org/10.1002/ppul.25082>

Ahlers-Schmidt, C. R., Hervey, A. M., Neil, T., Kuhlmann, S., & Kuhlmann, Z. (2020). Concerns of women regarding pregnancy and childbirth during the COVID-19 pandemic. *Patient Education and Counseling*, *103*(12), 2578-2582. <https://doi.org/10.1016/j.pec.2020.09.031>

Ahorsu, D. K., Imani, V., Lin, C. Y., Timpka, T., Broström, A., Updegraff, J. A., . . . Pakpour, A. H. (2020). Associations Between Fear of COVID-19, Mental Health, and Preventive Behaviours Across Pregnant Women and Husbands: An Actor-Partner Interdependence Modelling. *Int J Ment Health Addict*, 1-15. <https://doi.org/10.1007/s11469-020-00340-x>

Aimen, C., Bari, A., Rashid, J., Alvi, Y., Naz, F., Rana, N., . . . Sadiq, M. (2020). Comorbidity and covid-19 in children-a single center experience. *Pakistan Paediatric Journal*, *44*(4), 306-313.

Akgor, U., Fadıloglu, E., Soyak, B., Unal, C., Cagan, M., Temiz, B. E., . . . Ozyuncu, O. (2021). Anxiety, depression and concerns of pregnant women during the COVID-19 pandemic. *Archives of Gynecology and Obstetrics*, *304*(1), 125-130. <https://doi.org/10.1007/s00404-020-05944-1>

Aksoy Derya, Y., Altiparmak, S., AkÇa, E., GÖkbulut, N., & Yilmaz, A. N. (2020). Pregnancy and birth planning during COVID-19: The effects of tele-education offered to pregnant women on prenatal distress and pregnancy-related anxiety. *Midwifery*, *92*, 102877. <https://doi.org/10.1016/j.midw.2020.102877>

Al-Matary, A., Almatari, F., Al-Matary, M., AlDhaefi, A., Alqahtani, M. H. S., Alhulaimi, E. A., . . . Aldandan, F. K. (2021). Clinical outcomes of maternal and neonate with COVID-19 infection - Multicenter study in Saudi Arabia. *J Infect Public Health*, *14*(6), 702-708. <https://doi.org/10.1016/j.jiph.2021.03.013>

Alaya, F., Worrall, A. P., O'Toole, F., Doyle, J., Duffy, R. M., & Geary, M. P. (2021). Health-related quality of life and quality of care in pregnant and postnatal women during the coronavirus disease 2019 pandemic: A cohort study. *International Journal of Gynaecology and Obstetrics*. <https://doi.org/10.1002/ijgo.13711>

Alhuzimi, T. (2021). Stress and emotional wellbeing of parents due to change in routine for children with Autism Spectrum Disorder (ASD) at home during COVID-19 pandemic in Saudi Arabia. *Research in Developmental Disabilities*, *108*, 103822. <https://doi.org/10.1016/j.ridd.2020.103822>

Alsuhaibani, M., & Alaqeel, A. (2020). Impact of the COVID-19 Pandemic on Routine Childhood Immunization in Saudi Arabia. *Vaccines (Basel)*, *8*(4). <https://doi.org/10.3390/vaccines8040581>

An, R., Chen, X., Wu, Y., Liu, J., Deng, C., Liu, Y., & Guo, H. (2021). A survey of postpartum depression and health care needs among Chinese postpartum women during the pandemic of COVID-19. *Archives of Psychiatric Nursing*, *35*(2), 172-177. <https://doi.org/10.1016/j.apnu.2021.02.001>

Ares, G., Bove, I., Vidal, L., Brunet, G., Fuletti, D., Arroyo, Á., & Blanc, M. V. (2021). The experience of social distancing for families with children and adolescents during the coronavirus (COVID-19) pandemic in Uruguay: Difficulties and opportunities. *Child Youth Serv Rev*, *121*, 105906. <https://doi.org/10.1016/j.childyouth.2020.105906>

Aronu, A. E., Chinawa, J. M., Nduagubam, O. C., Ossai, E. N., Chinawa, A. T., & Igwe, W. C. (2020). Maternal perception of masking in children as a preventive strategy for COVID-19 in Nigeria: A multicentre study. *PloS One*, *15*(11 November). <https://doi.org/10.1371/journal.pone.0242650>

Asai, K., Wakashima, K., Toda, S., & Koiwa, K. (2021). Fear of novel coronavirus disease (COVID-19) among pregnant and infertile women in Japan. *J Affect Disord Rep*, *4*, 100104. <https://doi.org/10.1016/j.jadr.2021.100104>

Ashini, A., Alsoufi, A., & Elhadi, M. (2021). Parental perception of neonatal ICU visitation during the COVID-19 pandemic. *International Journal of Gynaecology and Obstetrics*, *153*(3), 554-555. <https://doi.org/10.1002/ijgo.13650>

Auðardóttir, A. M., & Rúdólfsdóttir, A. G. (2020). Chaos ruined the children's sleep, diet and behaviour: Gendered discourses on family life in pandemic times. *Gend Work Organ*. <https://doi.org/10.1111/gwao.12519>

Ayas, M., Ali Al Amadi, A. M. H., Khaled, D., & Alwaa, A. M. (2020). Impact of COVID-19 on the access to hearing health care services for children with cochlear implants: A survey of parents. *F1000Research*, *9*. <https://doi.org/10.12688/f1000research.24915.1>

Ayaz, R., Hocaoğlu, M., Günay, T., Yardımcı, O. D., Turgut, A., & Karateke, A. (2020). Anxiety and depression symptoms in the same pregnant women before and during the COVID-19 pandemic. *Journal of Perinatal Medicine*, *48*(9), 965-970. <https://doi.org/10.1515/jpm-2020-0380>

Aydin, R., & Aktaş, S. (2021). An investigation of women's pregnancy experiences during the covid-19 pandemic: A qualitative study. *International Journal of Clinical Practice*, e14418. <https://doi.org/10.1111/ijcp.14418>

Bao, X., Qu, H., Zhang, R., & Hogan, T. P. (2020). Modeling reading ability gain in kindergarten children during COVID-19 school closures. *International Journal of Environmental Research and Public Health*, *17*(17), 6371.

Barbosa-Leiker, C., Smith, C. L., Crespi, E. J., Brooks, O., Burduli, E., Ranjo, S., . . . Gartstein, M. A. (2021). Stressors, coping, and resources needed during the COVID-19 pandemic in a sample of perinatal women. *BMC Pregnancy and Childbirth*, *21*(1), 171. <https://doi.org/10.1186/s12884-021-03665-0>

Basu, A., Kim, H. H., Basaldua, R., Choi, K. W., Charron, L., Kelsall, N., . . . Koenen, K. C. (2021). A cross-national study of factors associated with women's perinatal mental health and wellbeing during the COVID-19 pandemic. *PloS One*, *16*(4), e0249780. <https://doi.org/10.1371/journal.pone.0249780>

Bender, W. R., Srinivas, S., Coutifaris, P., Acker, A., & Hirshberg, A. (2020). The Psychological Experience of Obstetric Patients and Health Care Workers after Implementation of Universal SARS-CoV-2 Testing. *American Journal of Perinatology*, *37*(12), 1271-1279. <https://doi.org/10.1055/s-0040-1715505>

Bentenuto, A., Mazzoni, N., Giannotti, M., Venuti, P., & de Falco, S. (2021). Psychological impact of Covid-19 pandemic in Italian families of children with neurodevelopmental disorders. *Research in Developmental Disabilities*, *109*, 103840.

Berard, M., Rattaz, C., Peries, M., Loubersac, J., Munir, K., & Baghdadli, A. (2021). Impact of containment and mitigation measures on children and youth with ASD during the COVID-19 pandemic: Report from the ELENA cohort. *Journal of Psychiatric Research*, *137*, 73-80. <https://doi.org/10.1016/j.jpsychires.2021.02.041>

Berthelot, N., Lemieux, R., Garon-Bissonnette, J., Drouin-Maziade, C., Martel, É., & Maziade, M. (2020). Uptrend in distress and psychiatric symptomatology in pregnant women during the coronavirus disease 2019 pandemic. *Acta Obstetricia et Gynecologica Scandinavica*, *99*(7), 848-855. <https://doi.org/10.1111/aogs.13925>

Bıkmazer, A., Kadak, M. T., Görmez, V., Doğan, U., Aslankaya, Z. D., Bakır, F., . . . Öztürk, M. (2020). Parental psychological distress associated with COVID-19 outbreak: A large-scale multicenter survey from Turkey. *International Journal of Social Psychiatry*, 20764020970240. <https://doi.org/10.1177/0020764020970240>

Bin-Nun, A., Palmor-Haspal, S., Mimouni, F. B., Kasirer, Y., Hammerman, C., & Tuval-Moshiach, R. (2021). Infant delivery and maternal stress during the COVID-19 pandemic: a comparison of the well-baby versus neonatal intensive care environments. *Journal of Perinatology*, 1-7. <https://doi.org/10.1038/s41372-021-01016-7>

Bo, H. X., Yang, Y., Chen, J., Zhang, M., Li, Y., Zhang, D. Y., . . . Xiang, Y. T. (2021). Prevalence of Depressive Symptoms Among Pregnant and Postpartum Women in China During the COVID-19 Pandemic. *Psychosomatic Medicine*, *83*(4), 345-350. <https://doi.org/10.1097/psy.0000000000000904>

Boekhorst, M., Muskens, L., Hulsbosch, L. P., Van Deun, K., Bergink, V., Pop, V. J. M., & van den Heuvel, M. I. (2021). The COVID-19 outbreak increases maternal stress during pregnancy, but not the risk for postpartum depression. *Arch Womens Ment Health*, 1-7. <https://doi.org/10.1007/s00737-021-01104-9>

Bradfield, Z., Wynter, K., Hauck, Y., Vasilevski, V., Kuliukas, L., Wilson, A. N., . . . Sweet, L. (2021). Experiences of receiving and providing maternity care during the COVID-19 pandemic in Australia: A five-cohort cross-sectional comparison. *PloS One*, *16*(3), e0248488. <https://doi.org/10.1371/journal.pone.0248488>

Brandt, J. S., Hill, J., Reddy, A., Schuster, M., Patrick, H. S., Rosen, T., . . . Ananth, C. V. (2020). Epidemiology of coronavirus disease 2019 in pregnancy: risk factors and associations with adverse maternal and neonatal outcomes. *American Journal of Obstetrics and Gynecology*. <https://doi.org/10.1016/j.ajog.2020.09.043>

Brisca, G., Vagelli, G., Tagliarini, G., Rotulo, A., Pirlo, D., Romanengo, M., & Piccotti, E. (2021). The impact of COVID-19 lockdown on children with medical complexity in pediatric emergency department. *American Journal of Emergency Medicine*, *42*, 225-227. <https://doi.org/10.1016/j.ajem.2020.11.066>

Brown, A., & Shenker, N. (2021). Experiences of breastfeeding during COVID‐19: Lessons for future practical and emotional support. *Maternal & child nutrition*, *17*(1), e13088.

Brown, S. M., Doom, J. R., Lechuga-Peña, S., Watamura, S. E., & Koppels, T. (2020). Stress and parenting during the global COVID-19 pandemic. *Child Abuse and Neglect*, 104699. <https://doi.org/10.1016/j.chiabu.2020.104699>

Buonsenso, D., Iodice, F., Cinicola, B., Raffaelli, F., Sowa, S., & Ricciardi, W. (2020). Management of Malaria in Children Younger Than 5 Years Old During Coronavirus Disease 2019 Pandemic in Sierra Leone: A Lesson Learned? *Frontiers in Pediatrics*, *8*. <https://doi.org/10.3389/fped.2020.587638>

Cacioppo, M., Bouvier, S., Bailly, R., Houx, L., Lempereur, M., Mensah-Gourmel, J., . . . Pons, C. (2021). Emerging health challenges for children with physical disabilities and their parents during the COVID-19 pandemic: The ECHO French survey. *64*(3). <https://doi.org/10.1016/j.rehab.2020.08.001>

Calvano, C., Engelke, L., Di Bella, J., Kindermann, J., Renneberg, B., & Winter, S. M. (2021). Families in the COVID-19 pandemic: parental stress, parent mental health and the occurrence of adverse childhood experiences-results of a representative survey in Germany. *European Child and Adolescent Psychiatry*, 1-13. <https://doi.org/10.1007/s00787-021-01739-0>

Camerlink, I., Nielsen, B. L., Windschnurer, I., & Vigors, B. (2021). Impacts of the COVID-19 pandemic on animal behaviour and welfare researchers. *Applied Animal Behaviour Science*, *236*, 105255. <https://doi.org/10.1016/j.applanim.2021.105255>

Cameron, E. E., Joyce, K. M., Delaquis, C. P., Reynolds, K., Protudjer, J. L. P., & Roos, L. E. (2020). Maternal psychological distress & mental health service use during the COVID-19 pandemic. *Journal of Affective Disorders*, *276*, 765-774. <https://doi.org/10.1016/j.jad.2020.07.081>

Campagnaro, R., Collet, G. O., Andrade, M. P., Salles, J., Calvo Fracasso, M. L., Scheffel, D. L. S., . . . Santin, G. C. (2020). COVID-19 pandemic and pediatric dentistry: Fear, eating habits and parent's oral health perceptions. *Child Youth Serv Rev*, *118*, 105469. <https://doi.org/10.1016/j.childyouth.2020.105469>

Cao, Y., Huang, L., Si, T., Wang, N. Q., Qu, M., & Zhang, X. Y. (2021). The role of only-child status in the psychological impact of COVID-19 on mental health of Chinese adolescents. *Journal of Affective Disorders*, *282*, 316-321. <https://doi.org/10.1016/j.jad.2020.12.113>

Carroll, N., Sadowski, A., Laila, A., Hruska, V., Nixon, M., Ma, D. W., & Haines, J. (2020). The impact of COVID-19 on health behavior, stress, financial and food security among middle to high income Canadian families with young children. *Nutrients*, *12*(8), 2352.

Celik, H., Acikel, S. B., Ozdemir, F. M. A., Aksoy, E., Oztoprak, U., Cucu, E., . . . Yuksel, D. (2021). Evaluation of the Anxiety Level of Mothers of Children with Epilepsy during the COVID-19 Pandemic Period. *European Neurology*, *84*(3), 192-199. <https://doi.org/10.1159/000514826>

Cellini, N., Di Giorgio, E., Mioni, G., & Di Riso, D. (2021). Sleep and Psychological Difficulties in Italian School-Age Children During COVID-19 Lockdown. *Journal of Pediatric Psychology*, *46*(2), 153-167. <https://doi.org/10.1093/jpepsy/jsab003>

Ceulemans, M., Foulon, V., Ngo, E., Panchaud, A., Winterfeld, U., Pomar, L., . . . Nordeng, H. (2021). Mental health status of pregnant and breastfeeding women during the COVID-19 pandemic-A multinational cross-sectional study. *Acta Obstetricia et Gynecologica Scandinavica*. <https://doi.org/10.1111/aogs.14092>

Ceulemans, M., Hompes, T., & Foulon, V. (2020). Mental health status of pregnant and breastfeeding women during the COVID-19 pandemic: A call for action. *International Journal of Gynaecology and Obstetrics*, *151*(1), 146-147. <https://doi.org/10.1002/ijgo.13295>

Chan, R. C. H., & Fung, S. C. (2021). Elevated Levels of COVID-19-Related Stress and Mental Health Problems Among Parents of Children with Developmental Disorders During the Pandemic. *Journal of Autism and Developmental Disorders*, 1-12. <https://doi.org/10.1007/s10803-021-05004-w>

Chapman, G. E., Ishlek, I., & Spoors, J. (2021). Google search behaviour relating to perinatal mental wellbeing during the United Kingdom's first COVID-19 lockdown period: a warning for future restrictions. *Arch Womens Ment Health*, *24*(4), 681-686. <https://doi.org/10.1007/s00737-021-01110-x>

Chasson, M., Taubman-Ben-Ari, O., & Abu-Sharkia, S. (2020). Jewish and Arab pregnant women's psychological distress during the COVID-19 pandemic: the contribution of personal resources. *Ethnicity and Health*, 1-13. <https://doi.org/10.1080/13557858.2020.1815000>

Chaves, C., Marchena, C., Palacios, B., Salgado, A., & Duque, A. (2021). Effects of the COVID-19 pandemic on perinatal mental health in Spain: Positive and negative outcomes. *Women Birth*. <https://doi.org/10.1016/j.wombi.2021.01.007>

Chen, H., Guo, J., Wang, C., Luo, F., Yu, X., Zhang, W., . . . Zhang, Y. (2020). Clinical characteristics and intrauterine vertical transmission potential of COVID-19 infection in nine pregnant women: a retrospective review of medical records. *Lancet*, *395*(10226), 809-815. <https://doi.org/10.1016/s0140-6736(20)30360-3>

Chen, S., Bernstein, P., Nair, S., Romanelli, E., Khoury, R., Labins, J., . . . Reddy, S. (2021). A review of 92 obstetric patients with COVID-19 in the Bronx, New York and their peripartum anaesthetic management. *Anaesthesiol Intensive Ther*, 1-11. <https://doi.org/10.5114/ait.2021.105120>

Chen, S. Q., Chen, S. D., Li, X. K., & Ren, J. (2020). Mental Health of Parents of Special Needs Children in China during the COVID-19 Pandemic. *International Journal of Environmental Research and Public Health*, *17*(24). <https://doi.org/10.3390/ijerph17249519>

Cheng, Z., Mendolia, S., Paloyo, A. R., Savage, D. A., & Tani, M. (2021). Working parents, financial insecurity, and childcare: mental health in the time of COVID-19 in the UK. *Rev Econ Househ*, 1-22. <https://doi.org/10.1007/s11150-020-09538-3>

Chivers, B. R., Garad, R. M., Boyle, J. A., Skouteris, H., Teede, H. J., & Harrison, C. L. (2020). Perinatal Distress During COVID-19: Thematic Analysis of an Online Parenting Forum. *Journal of Medical Internet Research*, *22*(9), e22002. <https://doi.org/10.2196/22002>

Cho, E., & Ilari, B. S. (2021). Mothers as Home DJs: Recorded Music and Young Children's Well-Being During the COVID-19 Pandemic. *Frontiers in Psychology*, *12*, 637569. <https://doi.org/10.3389/fpsyg.2021.637569>

Choi, J., Park, Y., Kim, H. E., Song, J., Lee, D., Lee, E., . . . Lee, Y. (2021). Daily Life Changes and Life Satisfaction among Korean School-Aged Children in the COVID-19 Pandemic. *International Journal of Environmental Research and Public Health*, *18*(6). <https://doi.org/10.3390/ijerph18063324>

Chrzan-Dętkoś, M., Walczak-Kozłowska, T., & Lipowska, M. (2021). The need for additional mental health support for women in the postpartum period in the times of epidemic crisis. *BMC Pregnancy and Childbirth*, *21*(1), 114. <https://doi.org/10.1186/s12884-021-03544-8>

Chung, G., Lanier, P., & Wong, P. Y. J. (2020). Mediating Effects of Parental Stress on Harsh Parenting and Parent-Child Relationship during Coronavirus (COVID-19) Pandemic in Singapore. *J Fam Violence*, 1-12. <https://doi.org/10.1007/s10896-020-00200-1>

Cohodes, E. M., McCauley, S., & Gee, D. G. (2021). Parental Buffering of Stress in the Time of COVID-19: Family-Level Factors May Moderate the Association Between Pandemic-Related Stress and Youth Symptomatology. *Res Child Adolesc Psychopathol*, *49*(7), 935-948. <https://doi.org/10.1007/s10802-020-00732-6>

Colizzi, M., Sironi, E., Antonini, F., Ciceri, M. L., Bovo, C., & Zoccante, L. (2020). Psychosocial and behavioral impact of COVID-19 in autism spectrum disorder: An online parent survey. *Brain Sciences*, *10*(6). <https://doi.org/10.3390/brainsci10060341>

Connell, C. M., & Strambler, M. J. (2021). Experiences With COVID-19 Stressors and Parents' Use of Neglectful, Harsh, and Positive Parenting Practices in the Northeastern United States. *Child Maltreat*, 10775595211006465. <https://doi.org/10.1177/10775595211006465>

Conti, M. G., Natale, F., Stolfi, I., Pedicino, R., Boscarino, G., Ajassa, C., . . . Terrin, G. (2021). Consequences of Early Separation of Maternal-Newborn Dyad in Neonates Born to SARS-CoV-2 Positive Mothers: An Observational Study. *International Journal of Environmental Research and Public Health*, *18*(11). <https://doi.org/10.3390/ijerph18115899>

Cooper, S. M., Thomas, A., & Bamishigbin, O. (2021). Black American Fathers Employed in Higher-Risk Contexts for Contracting COVID-19: Implications for Individual Wellbeing and Work-Family Spillover. *Am J Mens Health*, *15*(2), 15579883211005617. <https://doi.org/10.1177/15579883211005617>

Corbett, G. A., Milne, S. J., Hehir, M. P., Lindow, S. W., & O'Connell M, P. (2020). Health anxiety and behavioural changes of pregnant women during the COVID-19 pandemic. *European Journal of Obstetrics, Gynecology, and Reproductive Biology*, *249*, 96-97. <https://doi.org/10.1016/j.ejogrb.2020.04.022>

Costoya, V., Echeverría, L., Edo, M., Rocha, A., & Thailinger, A. (2021). Gender Gaps within Couples: Evidence of Time Re-allocations during COVID-19 in Argentina. *J Fam Econ Issues*, 1-14. <https://doi.org/10.1007/s10834-021-09770-8>

Craig, L., & Churchill, B. (2020). Dual-earner Parent Couples' Work and Care during COVID-19. *Gend Work Organ*. <https://doi.org/10.1111/gwao.12497>

Cui, S., Zhang, C., Wang, S., Zhang, X., Wang, L., Zhang, L., . . . Zhou, X. (2021). Experiences and Attitudes of Elementary School Students and Their Parents Toward Online Learning in China During the COVID-19 Pandemic: Questionnaire Study. *Journal of Medical Internet Research*, *23*(5), e24496. <https://doi.org/10.2196/24496>

Cui, Y., Li, Y., & Zheng, Y. (2020). Mental health services for children in China during the COVID-19 pandemic: results of an expert-based national survey among child and adolescent psychiatric hospitals. *European Child and Adolescent Psychiatry*, *29*(6), 743-748. <https://doi.org/10.1007/s00787-020-01548-x>

Cusinato, M., Iannattone, S., Spoto, A., Poli, M., Moretti, C., Gatta, M., & Miscioscia, M. (2020). Stress, Resilience, and Well-Being in Italian Children and Their Parents during the COVID-19 Pandemic. *International Journal of Environmental Research and Public Health*, *17*(22). <https://doi.org/10.3390/ijerph17228297>

Dagklis, T., Tsakiridis, I., Mamopoulos, A., Athanasiadis, A., Pearson, R., & Papazisis, G. (2020). Impact of the COVID-19 lockdown on antenatal mental health in Greece. *Psychiatry and Clinical Neurosciences*, *74*(11), 616-617. <https://doi.org/10.1111/pcn.13135>

Daks, J. S., Peltz, J. S., & Rogge, R. D. (2020). Psychological flexibility and inflexibility as sources of resiliency and risk during a pandemic: Modeling the cascade of COVID-19 stress on family systems with a contextual behavioral science lens. *J Contextual Behav Sci*, *18*, 16-27. <https://doi.org/10.1016/j.jcbs.2020.08.003>

Daulay, N. (2021). Home education for children with autism spectrum disorder during the COVID-19 pandemic: Indonesian mothers experience. *Research in Developmental Disabilities*, *114*, 103954. <https://doi.org/10.1016/j.ridd.2021.103954>

de Sá, C. D. S. C., Pombo, A., Luz, C., Rodrigues, L. P., & Cordovil, R. (2020). Covid-19 social isolation in Brazil: Effects on the physical activity routine of families with children. *Revista Paulista de Pediatria*, *39*. <https://doi.org/10.1590/1984-0462/2021/39/2020159>

Del Boca, D., Oggero, N., Profeta, P., & Rossi, M. (2020). Women's and men's work, housework and childcare, before and during COVID-19. *Rev Econ Househ*, 1-17. <https://doi.org/10.1007/s11150-020-09502-1>

Del Río, R., Dip Pérez, E., & Marín Gabriel, M. (2021). Multi-centre study showed reduced compliance with the World Health Organization recommendations on exclusive breastfeeding during COVID-19. *Acta Paediatrica*, *110*(3), 935-936. <https://doi.org/10.1111/apa.15642>

Dell'Utri, C., Manzoni, E., Cipriani, S., Spizzico, C., Dell'Acqua, A., Barbara, G., . . . Kustermann, A. (2020). Effects of SARS Cov-2 epidemic on the obstetrical and gynecological emergency service accesses. What happened and what shall we expect now? *European Journal of Obstetrics, Gynecology, and Reproductive Biology*, *254*, 64-68. <https://doi.org/10.1016/j.ejogrb.2020.09.006>

Dellagiulia, A., Lionetti, F., Fasolo, M., Verderame, C., Sperati, A., & Alessandri, G. (2020). Early impact of COVID-19 lockdown on children's sleep: A 4-week longitudinal study. *Journal of Clinical Sleep Medicine*, *16*(9), 1639-1640. <https://doi.org/10.5664/jcsm.8648>

DeYoung, S. E., & Mangum, M. (2021). Pregnancy, Birthing, and Postpartum Experiences During COVID-19 in the United States. *Front Sociol*, *6*, 611212. <https://doi.org/10.3389/fsoc.2021.611212>

Dhiman, S., Sahu, P. K., Reed, W. R., Ganesh, G. S., Goyal, R. K., & Jain, S. (2020). Impact of COVID-19 outbreak on mental health and perceived strain among caregivers tending children with special needs. *Research in Developmental Disabilities*, *107*. <https://doi.org/10.1016/j.ridd.2020.103790>

Di Riso, D., Spaggiari, S., Cambrisi, E., Ferraro, V., Carraro, S., & Zanconato, S. (2021). Psychosocial impact of Covid-19 outbreak on Italian asthmatic children and their mothers in a post lockdown scenario. *Scientific Reports*, *11*(1), 1-8.

Dib, S., Rougeaux, E., Vázquez-Vázquez, A., Wells, J. C. K., & Fewtrell, M. (2020). Maternal mental health and coping during the COVID-19 lockdown in the UK: Data from the COVID-19 New Mum Study. *International Journal of Gynaecology and Obstetrics*, *151*(3), 407-414. <https://doi.org/10.1002/ijgo.13397>

Dickerson, J., Kelly, B., Lockyer, B., Bridges, S., Cartwright, C., Willan, K., . . . Pickett, K. E. (2020). Experiences of lockdown during the Covid-19 pandemic: descriptive findings from a survey of families in the Born in Bradford study. *Wellcome Open Res*, *5*, 228. <https://doi.org/10.12688/wellcomeopenres.16317.2>

Ding, W., Lu, J., Zhou, Y., Wei, W., Zhou, Z., & Chen, M. (2021). Knowledge, attitudes, practices, and influencing factors of anxiety among pregnant women in Wuhan during the outbreak of COVID-19: a cross-sectional study. *BMC Pregnancy and Childbirth*, *21*(1), 80. <https://doi.org/10.1186/s12884-021-03561-7>

Dong, H., Hu, R., Lu, C., Huang, D., Cui, D., Huang, G., & Zhang, M. (2020). Investigation on the mental health status of pregnant women in China during the Pandemic of COVID-19. *Archives of Gynecology and Obstetrics*, 1-7. <https://doi.org/10.1007/s00404-020-05805-x>

Dule, A., Hajure, M., Mohammedhussein, M., & Abdu, Z. (2021). Health-related quality of life among Ethiopian pregnant women during COVID-19 pandemic. *Brain Behav*, *11*(4), e02045. <https://doi.org/10.1002/brb3.2045>

Dulfe, P. A. M., Alves, V. H., Pereira, A. V., Vieira, B. D. G., Rodrigues, D. P., Marchiori, G. R. S., & Branco, M. (2021). Nurse-midwives reconfiguring care in the scope of labor and births in COVID-19 times. *Rev Bras Enferm*, *74Suppl 1*(Suppl 1), e20200863. <https://doi.org/10.1590/0034-7167-2020-0863>

Dumbre, D. U., Ramesh, S., Chavan, R., & Jabade, M. (2020). A descriptive study to assess the stress and coping mechanism due to lockdown among school going children. *Indian Journal of Forensic Medicine and Toxicology*, *14*(4), 3590-3597. <https://doi.org/10.37506/ijfmt.v14i4.12186>

Durankuş, F., & Aksu, E. (2020). Effects of the COVID-19 pandemic on anxiety and depressive symptoms in pregnant women: a preliminary study. *Journal of Maternal-Fetal & Neonatal Medicine*, 1-7. <https://doi.org/10.1080/14767058.2020.1763946>

Effati-Daryani, F., Zarei, S., Mohammadi, A., Hemmati, E., Ghasemi Yngyknd, S., & Mirghafourvand, M. (2020). Depression, stress, anxiety and their predictors in Iranian pregnant women during the outbreak of COVID-19. *BMC Psychol*, *8*(1), 99. <https://doi.org/10.1186/s40359-020-00464-8>

Ehsan, S. M. A., & Jahan, F. (2021). Analysing the impact of COVID-19 on the mothers of Bangladesh: hearing the unheard. *Z Gesundh Wiss*, 1-14. <https://doi.org/10.1007/s10389-021-01501-5>

El-Osta, A., Alaa, A., Webber, I., Riboli Sasco, E., Bagkeris, E., Millar, H., . . . Majeed, A. (2021). How is the COVID-19 lockdown impacting the mental health of parents of school-age children in the UK? A cross-sectional online survey. *BMJ Open*, *11*(5), e043397. <https://doi.org/10.1136/bmjopen-2020-043397>

Ergenekon, A. P., Yilmaz Yegit, C., Cenk, M., Bas Ikizoglu, N., Atag, E., Gokdemir, Y., . . . Karadag, B. (2020). Depression and anxiety in mothers of home ventilated children before and during COVID-19 pandemic. *Pediatric Pulmonology*. <https://doi.org/10.1002/ppul.25107>

Evans, S., Mikocka-Walus, A., Klas, A., Olive, L., Sciberras, E., Karantzas, G., & Westrupp, E. M. (2020). From "It Has Stopped Our Lives" to "Spending More Time Together Has Strengthened Bonds": The Varied Experiences of Australian Families During COVID-19. *Frontiers in Psychology*, *11*, 588667. <https://doi.org/10.3389/fpsyg.2020.588667>

Faccioli, S., Lombardi, F., Bellini, P., Costi, S., Sassi, S., & Pesci, M. C. (2021). How did italian adolescents with disability and parents deal with the covid-19 emergency? *International Journal of Environmental Research and Public Health*, *18*(4), 1-13. <https://doi.org/10.3390/ijerph18041687>

Fallon, V., Davies, S. M., Silverio, S. A., Jackson, L., De Pascalis, L., & Harrold, J. A. (2021). Psychosocial experiences of postnatal women during the COVID-19 pandemic. A UK-wide study of prevalence rates and risk factors for clinically relevant depression and anxiety. *Journal of Psychiatric Research*, *136*, 157-166. <https://doi.org/10.1016/j.jpsychires.2021.01.048>

Farewell, C. V., Jewell, J., Walls, J., & Leiferman, J. A. (2020). A Mixed-Methods Pilot Study of Perinatal Risk and Resilience During COVID-19. *Journal of Primary Care & Community Health*, *11*, 2150132720944074. <https://doi.org/10.1177/2150132720944074>

Farghaly, M. A. A., Kupferman, F., Castillo, F., & Kim, R. M. (2020). Characteristics of Newborns Born to SARS-CoV-2-Positive Mothers: A Retrospective Cohort Study. *American Journal of Perinatology*, *37*(13), 1310-1316. <https://doi.org/10.1055/s-0040-1715862>

Farrell, T., Reagu, S., Mohan, S., Elmidany, R., Qaddoura, F., Ahmed, E. E., . . . Alabdulla, M. A. (2020). The impact of the COVID-19 pandemic on the perinatal mental health of women. *Journal of Perinatal Medicine*, *48*(9), 971-976. <https://doi.org/10.1515/jpm-2020-0415>

Farsi, D., & Farsi, N. (2021). Mothers' Knowledge, Attitudes, and Fears About Dental Visits During the COVID-19 Pandemic: A Cross-sectional Study. *J Int Soc Prev Community Dent*, *11*(1), 83-91. <https://doi.org/10.4103/jispcd.JISPCD_395_20>

Fernandes, D. V., Canavarro, M. C., & Moreira, H. (2021). Postpartum during COVID-19 pandemic: Portuguese mothers' mental health, mindful parenting, and mother-infant bonding. *Journal of Clinical Psychology*. <https://doi.org/10.1002/jclp.23130>

Ferns, S. J., Gautam, S., & Hudak, M. L. (2021). COVID-19 and Gender Disparities in Pediatric Cardiologists with Dependent Care Responsibilities. *American Journal of Cardiology*, *147*, 137-142. <https://doi.org/10.1016/j.amjcard.2021.02.017>

Ferrante, M. J., Goldsmith, J., Tauriello, S., Epstein, L. H., Leone, L. A., & Anzman-Frasca, S. (2021). Food acquisition and daily life for U.S. families with 4-to 8-year-old children during COVID-19: Findings from a nationally representative survey [Article]. *International Journal of Environmental Research and Public Health*, *18*(4), 1-15. <https://doi.org/10.3390/ijerph18041734>

Fisher, A. P., Patronick, J., Gerhardt, C. A., Radonovich, K., Salloum, R., & Wade, S. L. (2021). Impact of COVID-19 on adolescent and emerging adult brain tumor survivors and their parents. *Pediatric Blood & Cancer*, e29116. <https://doi.org/10.1002/pbc.29116>

Forbes, L. K., Lamar, M. R., Speciale, M., & Donovan, C. (2021). Mothers' and fathers' parenting attitudes during COVID-19. *Current Psychology (New Brunswick, N.J.)*, 1-10. <https://doi.org/10.1007/s12144-021-01605-x>

Fosco, G. M., Sloan, C. J., Fang, S., & Feinberg, M. E. (2021). Family vulnerability and disruption during the COVID-19 pandemic: prospective pathways to child maladjustment. *Journal of Child Psychology and Psychiatry and Allied Disciplines*. <https://doi.org/10.1111/jcpp.13458>

Freedman, R., Hunter, S. K., Law, A. J., D'Alessandro, A., Noonan, K., Wyrwa, A., & Camille Hoffman, M. (2020). Maternal choline and respiratory coronavirus effects on fetal brain development. *Journal of Psychiatric Research*, *128*, 1-4. <https://doi.org/10.1016/j.jpsychires.2020.05.019>

Freisthler, B., Gruenewald, P. J., Tebben, E., Shockley McCarthy, K., & Price Wolf, J. (2021). Understanding at-the-moment stress for parents during COVID-19 stay-at-home restrictions. *Social Science and Medicine*, *279*, 114025. <https://doi.org/10.1016/j.socscimed.2021.114025>

Fumagalli, S., Ornaghi, S., Borrelli, S., Vergani, P., & Nespoli, A. (2021). The experiences of childbearing women who tested positive to COVID-19 during the pandemic in northern Italy. *Women Birth*. <https://doi.org/10.1016/j.wombi.2021.01.001>

Gadermann, A. C., Thomson, K. C., Richardson, C. G., Gagné, M., McAuliffe, C., Hirani, S., & Jenkins, E. (2021). Examining the impacts of the COVID-19 pandemic on family mental health in Canada: findings from a national cross-sectional study. *BMJ Open*, *11*(1), e042871. <https://doi.org/10.1136/bmjopen-2020-042871>

Garcia de Avila, M. A., Hamamoto Filho, P. T., Jacob, F., Alcantara, L. R. S., Berghammer, M., Jenholt Nolbris, M., . . . Nilsson, S. (2020). Children's Anxiety and Factors Related to the COVID-19 Pandemic: An Exploratory Study Using the Children's Anxiety Questionnaire and the Numerical Rating Scale. *International Journal of Environmental Research and Public Health*, *17*(16). <https://doi.org/10.3390/ijerph17165757>

Gassman-Pines, A., Ananat, E. O., & Fitz-Henley, J., 2nd. (2020). COVID-19 and Parent-Child Psychological Well-being. *Pediatrics*, *146*(4). <https://doi.org/10.1542/peds.2020-007294>

Ge, Y., Shi, C., Wu, B., Liu, Y., Chen, L., & Deng, Y. (2021). Anxiety and Adaptation of Behavior in Pregnant Zhuang Women During the COVID-19 Pandemic: A Mixed-Mode Survey. *Risk Management and Healthcare Policy*, *14*, 1563-1573. <https://doi.org/10.2147/rmhp.S303835>

Gildner, T. E., Laugier, E. J., & Thayer, Z. M. (2020). Exercise routine change is associated with prenatal depression scores during the COVID-19 pandemic among pregnant women across the United States. *PloS One*, *15*(12), e0243188. <https://doi.org/10.1371/journal.pone.0243188>

Giurge, L. M., Whillans, A. V., & Yemiscigil, A. (2021). A multicountry perspective on gender differences in time use during COVID-19. *Proceedings of the National Academy of Sciences of the United States of America*, *118*(12). <https://doi.org/10.1073/pnas.2018494118>

Glynn, L. M., Davis, E. P., Luby, J. L., Baram, T. Z., & Sandman, C. A. (2021). A predictable home environment may protect child mental health during the COVID-19 pandemic. *Neurobiol Stress*, *14*, 100291. <https://doi.org/10.1016/j.ynstr.2020.100291>

Green, M. J., Pearce, A., Parkes, A., Robertson, E., & Katikireddi, S. V. (2021). Pre-school childcare and inequalities in child development. *SSM Popul Health*, *14*, 100776. <https://doi.org/10.1016/j.ssmph.2021.100776>

Grumi, S., Provenzi, L., Gardani, A., Aramini, V., Dargenio, E., Naboni, C., . . . Borgatti, R. (2020). Rehabilitation services lockdown during the COVID-19 emergency: the mental health response of caregivers of children with neurodevelopmental disabilities. *Disability and Rehabilitation*, 1-6. <https://doi.org/10.1080/09638288.2020.1842520>

Guo, J., De Carli, P., Lodder, P., Bakermans-Kranenburg, M. J., & Riem, M. M. E. (2021). Maternal mental health during the COVID-19 lockdown in China, Italy, and the Netherlands: a cross-validation study. *Psychological Medicine*, 1-11. <https://doi.org/10.1017/s0033291720005504>

Gur, R. E., White, L. K., Waller, R., Barzilay, R., Moore, T. M., Kornfield, S., . . . Elovitz, M. A. (2020). The Disproportionate Burden of the COVID-19 Pandemic Among Pregnant Black Women. *Psychiatry Research*, *293*, 113475. <https://doi.org/10.1016/j.psychres.2020.113475>

Guruge, S., Lamaj, P., Lee, C., Ronquillo, C. E., Sidani, S., Leung, E., . . . Morrison, L. (2021). COVID-19 restrictions: experiences of immigrant parents in Toronto. *AIMS Public Health*, *8*(1), 172-185. <https://doi.org/10.3934/publichealth.2021013>

Günther-Bel, C., Vilaregut, A., Carratala, E., Torras-Garat, S., & Pérez-Testor, C. (2020). A Mixed-method Study of Individual, Couple, and Parental Functioning During the State-regulated COVID-19 Lockdown in Spain. *Family Process*, *59*(3), 1060-1079. <https://doi.org/10.1111/famp.12585>

Hailemariam, S., Agegnehu, W., & Derese, M. (2021). Exploring COVID-19 Related Factors Influencing Antenatal Care Services Uptake: A Qualitative Study among Women in a Rural Community in Southwest Ethiopia. *Journal of Primary Care & Community Health*, *12*, 2150132721996892. <https://doi.org/10.1177/2150132721996892>

Halley, M. C., Mathews, K. S., Diamond, L. C., Linos, E., Sarkar, U., Mangurian, C., . . . Jagsi, R. (2021). The Intersection of Work and Home Challenges Faced by Physician Mothers During the Coronavirus Disease 2019 Pandemic: A Mixed-Methods Analysis. *J Womens Health (Larchmt)*, *30*(4), 514-524. <https://doi.org/10.1089/jwh.2020.8964>

Hamadani, J. D., Hasan, M. I., Baldi, A. J., Hossain, S. J., Shiraji, S., Bhuiyan, M. S. A., . . . Pasricha, S. R. (2020). Immediate impact of stay-at-home orders to control COVID-19 transmission on socioeconomic conditions, food insecurity, mental health, and intimate partner violence in Bangladeshi women and their families: an interrupted time series. *Lancet Glob Health*, *8*(11), e1380-e1389. <https://doi.org/10.1016/s2214-109x(20)30366-1>

Hamzehgardeshi, Z., Omidvar, S., Amoli, A. A., & Firouzbakht, M. (2021). Pregnancy-related anxiety and its associated factors during COVID-19 pandemic in Iranian pregnant women: a web-based cross-sectional study. *BMC Pregnancy and Childbirth*, *21*(1), 208. <https://doi.org/10.1186/s12884-021-03694-9>

Handayani, & Dina, H. (2021). Analysis of self-empowerment of pregnant women during Covid-19 pandemic. *Pakistan Journal of Medical and Health Sciences*, *15*(1), 333-336.

Harrison, V., Moulds, M. L., & Jones, K. (2021). Support from friends moderates the relationship between repetitive negative thinking and postnatal wellbeing during COVID-19. *Journal of Reproductive and Infant Psychology*, 1-16. <https://doi.org/10.1080/02646838.2021.1886260>

Hcini, N., Maamri, F., Picone, O., Carod, J. F., Lambert, V., Mathieu, M., . . . Pomar, L. (2021). Maternal, fetal and neonatal outcomes of large series of SARS-CoV-2 positive pregnancies in peripartum period: A single-center prospective comparative study. *European Journal of Obstetrics, Gynecology, and Reproductive Biology*, *257*, 11-18. <https://doi.org/10.1016/j.ejogrb.2020.11.068>

Hiiragi, K., Obata, S., Misumi, T., Miyagi, E., & Aoki, S. (2021). Psychological stress associated with the COVID-19 pandemic in postpartum women in Yokohama, Japan. *Journal of Obstetrics and Gynaecology Research*. <https://doi.org/10.1111/jog.14776>

Hiraoka, D., & Tomoda, A. (2020). Relationship between parenting stress and school closures due to the COVID-19 pandemic. *Psychiatry and Clinical Neurosciences*, *74*(9), 497-498. <https://doi.org/10.1111/pcn.13088>

Hjálmsdóttir, A., & Bjarnadóttir, V. S. (2021). “I have turned into a foreman here at home”: Families and work–life balance in times of COVID‐19 in a gender equality paradise. *Gender, Work & Organization*, *28*(1), 268-283.

Hocaoglu, M., Ayaz, R., Gunay, T., Akin, E., Turgut, A., & Karateke, A. (2020). Anxiety and Post-Traumatic Stress Disorder Symptoms in Pregnant Women during the COVID-19 Pandemic's Delay Phase. *Psychiatr Danub*, *32*(3-4), 521-526. <https://doi.org/10.24869/psyd.2020.521>

Horiuchi, S., Shinohara, R., Otawa, S., Akiyama, Y., Ooka, T., Kojima, R., . . . Yamagata, Z. (2020). Caregivers' mental distress and child health during the COVID-19 outbreak in Japan. *PloS One*, *15*(12), e0243702. <https://doi.org/10.1371/journal.pone.0243702>

Hu, X., Gao, J., Wei, Y., Chen, H., Sun, X., Chen, J., . . . Chen, L. (2020). Managing Preterm Infants Born to COVID-19 Mothers: Evidence from a Retrospective Cohort Study in Wuhan, China. *Neonatology*, 1-7. <https://doi.org/10.1159/000509141>

Huebener, M., Waights, S., Spiess, C. K., Siegel, N. A., & Wagner, G. G. (2021). Parental well-being in times of Covid-19 in Germany. *Rev Econ Househ*, 1-32. <https://doi.org/10.1007/s11150-020-09529-4>

Hui, P. W., Ma, G., Seto, M. T. Y., & Cheung, K. W. (2020). Effect of COVID-19 on delivery plans and postnatal depression scores of pregnant women. *Hong Kong Medical Journal. Xianggang Yi Xue Za Zhi*. <https://doi.org/10.12809/hkmj208774>

Hussong, A. M., Midgette, A. J., Thomas, T. E., Coffman, J. L., & Cho, S. (2021). Coping and Mental Health in Early Adolescence during COVID-19. *Res Child Adolesc Psychopathol*, 1-11. <https://doi.org/10.1007/s10802-021-00821-0>

Janevic, T., Maru, S., Nowlin, S., McCarthy, K., Bergink, V., Stone, J., . . . Howell, E. A. (2021). Pandemic Birthing: Childbirth Satisfaction, Perceived Health Care Bias, and Postpartum Health During the COVID-19 Pandemic. *Matern Child Health J*, *25*(6), 860-869. <https://doi.org/10.1007/s10995-021-03158-8>

Jani, S., Jacques, S. M., Qureshi, F., Natarajan, G., Bajaj, S., Velumula, P., . . . Bajaj, M. (2021). Clinical Characteristics of Mother-Infant Dyad and Placental Pathology in COVID-19 Cases in Predominantly African American Population. *AJP Rep*, *11*(1), e15-e20. <https://doi.org/10.1055/s-0040-1721673>

Jansen, E., Thapaliya, G., Aghababian, A., Sadler, J., Smith, K., & Carnell, S. (2021). Parental stress, food parenting practices and child snack intake during the COVID-19 pandemic. *Appetite*, *161*, 105119. <https://doi.org/10.1016/j.appet.2021.105119>

Jelly, P., Chadha, L., Kaur, N., Sharma, S., Sharma, R., Stephen, S., & Rohilla, J. (2021). Impact of COVID-19 Pandemic on the Psychological Status of Pregnant Women. *Cureus*, *13*(1), e12875. <https://doi.org/10.7759/cureus.12875>

Jiang, H., Jin, L., Qian, X., Xiong, X., La, X., Chen, W., . . . Li, M. (2021). Maternal Mental Health Status and Approaches for Accessing Antenatal Care Information During the COVID-19 Epidemic in China: Cross-Sectional Study. *Journal of Medical Internet Research*, *23*(1), e18722. <https://doi.org/10.2196/18722>

Jiang, Y., He, T., Lin, X., Zhou, Q., & Wu, Q. (2021). Caregivers' joint depressive symptoms and preschoolers' daily routines in Chinese three-generation families: Does household chaos matter? *Current Psychology (New Brunswick, N.J.)*, 1-9. <https://doi.org/10.1007/s12144-021-01595-w>

Kachi, Y., Fujiwara, T., Eguchi, H., Inoue, A., Baba, S., Ohta, H., & Tsutsumi, A. (2021). Association between maternity harassment and depression during pregnancy amid the COVID-19 state of emergency. *J Occup Health*, *63*(1), e12196. <https://doi.org/10.1002/1348-9585.12196>

Kahyaoglu Sut, H., & Kucukkaya, B. (2020). Anxiety, depression, and related factors in pregnant women during the COVID-19 pandemic in Turkey: A web-based cross-sectional study. *Perspectives in Psychiatric Care*. <https://doi.org/10.1111/ppc.12627>

Kallander, S. W., Gordon, R., & Borzekowski, D. L. G. (2021). "People Will Continue to Suffer If the Virus Is Around": A Qualitative Analysis of Sub-Saharan African Children's Experiences during the COVID-19 Pandemic. *International Journal of Environmental Research and Public Health*, *18*(11). <https://doi.org/10.3390/ijerph18115618>

Kar, P., Tomfohr-Madsen, L., Giesbrecht, G., Bagshawe, M., & Lebel, C. (2021). Alcohol and substance use in pregnancy during the COVID-19 pandemic. *Drug and Alcohol Dependence*, *225*, 108760. <https://doi.org/10.1016/j.drugalcdep.2021.108760>

Kassaw, C., & Pandey, D. (2020). The prevalence of general anxiety disorder and its associated factors among women's attending at the perinatal service of Dilla University referral hospital, Dilla town, Ethiopia, April, 2020 in Covid pandemic. *Heliyon*, *6*(11), e05593. <https://doi.org/10.1016/j.heliyon.2020.e05593>

Katayama, Y., Zha, L., Kitamura, T., Hirayama, A., Takeuchi, T., Tanaka, K., . . . On Behalf Of The Covid-Epidemiology Research Group Of Osaka, U. (2021). Characteristics and Outcomes of Pediatric COVID-19 Patients in Osaka, Japan. *International Journal of Environmental Research and Public Health*, *18*(11). <https://doi.org/10.3390/ijerph18115911>

Kawamura, H., Orisaka, M., & Yoshida, Y. (2021). Mentality of pregnant women and obstetric healthcare workers about prenatal SARS-CoV-2 testing: A regional survey over the first wave of the COVID-19 pandemic in Japan. *Journal of Obstetrics and Gynaecology Research*, *47*(5), 1763-1771. <https://doi.org/10.1111/jog.14740>

Khamees, R. E., Taha, O. T., & Ali, T. Y. M. (2021). Anxiety and depression during pregnancy in the era of COVID-19. *Journal of Perinatal Medicine*. <https://doi.org/10.1515/jpm-2021-0181>

Khoury, J. E., Atkinson, L., Bennett, T., Jack, S. M., & Gonzalez, A. (2021). COVID-19 and mental health during pregnancy: The importance of cognitive appraisal and social support. *Journal of Affective Disorders*, *282*, 1161-1169. <https://doi.org/10.1016/j.jad.2021.01.027>

Kimura, M., Kimura, K., & Ojima, T. (2021). Relationships between changes due to COVID-19 pandemic and the depressive and anxiety symptoms among mothers of infants and/or preschoolers: a prospective follow-up study from pre-COVID-19 Japan. *BMJ Open*, *11*(2), e044826. <https://doi.org/10.1136/bmjopen-2020-044826>

King, L. S., Feddoes, D. E., Kirshenbaum, J. S., Humphreys, K. L., & Gotlib, I. H. (2021). Pregnancy during the pandemic: the impact of COVID-19-related stress on risk for prenatal depression. *Psychological Medicine*, 1-11. <https://doi.org/10.1017/s003329172100132x>

Kinser, P. A., Jallo, N., Amstadter, A. B., Thacker, L. R., Jones, E., Moyer, S., . . . Salisbury, A. L. (2021). Depression, Anxiety, Resilience, and Coping: The Experience of Pregnant and New Mothers During the First Few Months of the COVID-19 Pandemic. *J Womens Health (Larchmt)*, *30*(5), 654-664. <https://doi.org/10.1089/jwh.2020.8866>

Korukcu, O., Ozkaya, M., Boran, O. F., & Bakacak, M. (2021). Factors associated with antenatal depression during the COVID-19 (SARS-CoV2) pandemic: A cross-sectional study in a cohort of Turkish pregnant women. *Perspectives in Psychiatric Care*. <https://doi.org/10.1111/ppc.12778>

Kotabagi, P., Fortune, L., Essien, S., Nauta, M., & Yoong, W. (2020). Anxiety and depression levels among pregnant women with COVID-19. *Acta Obstetricia et Gynecologica Scandinavica*, *99*(7), 953-954. <https://doi.org/10.1111/aogs.13928>

Kotabagi, P., Nauta, M., Fortune, L., & Yoong, W. (2020). COVID-19 positive mothers are not more anxious or depressed than non COVID pregnant women during the pandemic: A pilot case-control comparison. *European Journal of Obstetrics, Gynecology, and Reproductive Biology*, *252*, 615-616. <https://doi.org/10.1016/j.ejogrb.2020.07.037>

Kovler, M. L., Ziegfeld, S., Ryan, L. M., Goldstein, M. A., Gardner, R., Garcia, A. V., & Nasr, I. W. (2020). Increased proportion of physical child abuse injuries at a level I pediatric trauma center during the Covid-19 pandemic. *Child Abuse and Neglect*, 104756. <https://doi.org/10.1016/j.chiabu.2020.104756>

Koyucu, R. G., & Karaca, P. P. (2021). The Covid 19 outbreak: Maternal Mental Health and Associated Factors. *Midwifery*, *99*, 103013. <https://doi.org/10.1016/j.midw.2021.103013>

Kracht, C. L., Katzmarzyk, P. T., & Staiano, A. E. (2021). Household chaos, maternal stress, and maternal health behaviors in the United States during the COVID-19 outbreak. *Womens Health (Lond)*, *17*, 17455065211010655. <https://doi.org/10.1177/17455065211010655>

Kumari, A., Ranjan, P., Sharma, K. A., Sahu, A., Bharti, J., Zangmo, R., & Bhatla, N. (2021). Impact of COVID-19 on psychosocial functioning of peripartum women: A qualitative study comprising focus group discussions and in-depth interviews. *International Journal of Gynaecology and Obstetrics*, *152*(3), 321-327. <https://doi.org/10.1002/ijgo.13524>

Latorre, G., Martinelli, D., Guida, P., Masi, E., De Benedictis, R., & Maggio, L. (2021). Impact of COVID-19 pandemic lockdown on exclusive breastfeeding in non-infected mothers. *Int Breastfeed J*, *16*(1), 36. <https://doi.org/10.1186/s13006-021-00382-4>

Lauri Korajlija, A., & Jokic-Begic, N. (2020). COVID-19: Concerns and behaviours in Croatia. *British Journal of Health Psychology*. <https://doi.org/10.1111/bjhp.12425>

Lawson, M., Piel, M. H., & Simon, M. (2020). Child Maltreatment during the COVID-19 Pandemic: Consequences of Parental Job Loss on Psychological and Physical Abuse Towards Children. *Child Abuse and Neglect*, 104709. <https://doi.org/10.1016/j.chiabu.2020.104709>

Lebel, C., MacKinnon, A., Bagshawe, M., Tomfohr-Madsen, L., & Giesbrecht, G. (2020). Elevated depression and anxiety symptoms among pregnant individuals during the COVID-19 pandemic. *Journal of Affective Disorders*, *277*, 5-13. <https://doi.org/10.1016/j.jad.2020.07.126>

Lee, S. J., Ward, K. P., Chang, O. D., & Downing, K. M. (2021). Parenting activities and the transition to home-based education during the COVID-19 pandemic. *Children and Youth Services Review*, *122*, 105585.

Lee, S. J., Ward, K. P., Lee, J. Y., & Rodriguez, C. M. (2021). Parental Social Isolation and Child Maltreatment Risk during the COVID-19 Pandemic. *J Fam Violence*, 1-12. <https://doi.org/10.1007/s10896-020-00244-3>

Leeb, R. T., Bitsko, R. H., Radhakrishnan, L., Martinez, P., Njai, R., & Holland, K. M. (2020). Mental Health-Related Emergency Department Visits Among Children Aged <18 Years During the COVID-19 Pandemic - United States, January 1-October 17, 2020. *MMWR: Morbidity and Mortality Weekly Report*, *69*(45), 1675-1680. <https://doi.org/10.15585/mmwr.mm6945a3>

Lemieux, R., Garon-Bissonnette, J., Loiselle, M., Martel, É., Drouin-Maziade, C., & Berthelot, N. (2020). [Not Available]. *Canadian Journal of Psychiatry. Revue Canadienne de Psychiatrie*, 706743720963917. <https://doi.org/10.1177/0706743720963917>

Lemon, L., Edwards, R. P., & Simhan, H. N. (2021). What is driving the decreased incidence of preterm birth during the coronavirus disease 2019 pandemic? *Am J Obstet Gynecol MFM*, *3*(3), 100330. <https://doi.org/10.1016/j.ajogmf.2021.100330>

Li, C., Huo, L., Wang, R., Qi, L., Wang, W., Zhou, X., . . . Zhang, X. (2021). The prevalence and risk factors of depression in prenatal and postnatal women in China with the outbreak of Corona Virus Disease 2019. *Journal of Affective Disorders*, *282*, 1203-1209. <https://doi.org/10.1016/j.jad.2021.01.019>

Li, M., Yin, H., Jin, Z., Zhang, H., Leng, B., Luo, Y., & Zhao, Y. (2020). Impact of Wuhan lockdown on the indications of cesarean delivery and newborn weights during the epidemic period of COVID-19. *PloS One*, *15*(8), e0237420. <https://doi.org/10.1371/journal.pone.0237420>

Li, W., Wang, Z., Wang, G., Ip, P., Sun, X., Jiang, Y., & Jiang, F. (2021). Socioeconomic inequality in child mental health during the COVID-19 pandemic: First evidence from China. *Journal of Affective Disorders*, *287*, 8-14. <https://doi.org/10.1016/j.jad.2021.03.009>

Li, X., Lu, P., Hu, L., Huang, T., & Lu, L. (2020). Factors Associated with Mental Health Results among Workers with Income Losses Exposed to COVID-19 in China. *International Journal of Environmental Research and Public Health*, *17*(15). <https://doi.org/10.3390/ijerph17155627>

Liang, P., Wang, Y., Shi, S., Liu, Y., & Xiong, R. (2020). Prevalence and factors associated with postpartum depression during the COVID-19 pandemic among women in Guangzhou, China: a cross-sectional study. *BMC Psychiatry*, *20*(1), 557. <https://doi.org/10.1186/s12888-020-02969-3>

Liang, Z., Delvecchio, E., Cheng, Y., & Mazzeschi, C. (2021). Parent and Child's Negative Emotions During COVID-19: The Moderating Role of Parental Attachment Style. *Frontiers in Psychology*, *12*, 567483. <https://doi.org/10.3389/fpsyg.2021.567483>

Limbers, C. A., McCollum, C., & Greenwood, E. (2020). Physical activity moderates the association between parenting stress and quality of life in working mothers during the COVID-19 pandemic. *Ment Health Phys Act*, *19*, 100358. <https://doi.org/10.1016/j.mhpa.2020.100358>

Lin, W., Wu, B., Chen, B., Lai, G., Huang, S., Li, S., . . . Wang, Y. (2020). Sleep Conditions Associate with Anxiety and Depression Symptoms among Pregnant Women during the Epidemic of COVID-19 in Shenzhen. *Journal of Affective Disorders*. <https://doi.org/10.1016/j.jad.2020.11.114>

Linos, E., Halley, M. C., Sarkar, U., Mangurian, C., Sabry, H., Olazo, K., . . . Jagsi, R. (2021). Anxiety Levels Among Physician Mothers During the COVID-19 Pandemic. *American Journal of Psychiatry*, *178*(2), 203-204. <https://doi.org/10.1176/appi.ajp.2020.20071014>

Litmanovitz, I., Silberstein, D., Butler, S., & Vittner, D. (2021). Care of hospitalized infants and their families during the COVID-19 pandemic: an international survey. *Journal of Perinatology*, *41*(5), 981-987. <https://doi.org/10.1038/s41372-021-00960-8>

Liu, C. H., Erdei, C., & Mittal, L. (2021). Risk factors for depression, anxiety, and PTSD symptoms in perinatal women during the COVID-19 Pandemic. *Psychiatry Research*, *295*, 113552. <https://doi.org/10.1016/j.psychres.2020.113552>

Liu, C. H., Mittal, L., & Erdei, C. (2021). COVID-19-related health worries compound the psychiatric distress experienced by families of high-risk infants. *Journal of Perinatology*, *41*(5), 1191-1195. <https://doi.org/10.1038/s41372-021-01000-1>

Liu, J., Hung, P., Alberg, A. J., Hair, N. L., Whitaker, K. M., Simon, J., & Taylor, S. K. (2021). Mental health among pregnant women with COVID-19-related stressors and worries in the United States. *Birth*. <https://doi.org/10.1111/birt.12554>

Liu, X., Chen, M., Wang, Y., Sun, L., Zhang, J., Shi, Y., . . . Qi, H. (2020). Prenatal anxiety and obstetric decisions among pregnant women in Wuhan and Chongqing during the COVID-19 outbreak: a cross-sectional study. *BJOG: An International Journal of Obstetrics and Gynaecology*, *127*(10), 1229-1240. <https://doi.org/10.1111/1471-0528.16381>

Liu, Z., Tang, H., Jin, Q., Wang, G., Yang, Z., Chen, H., . . . Owens, J. (2020). Sleep of preschoolers during the coronavirus disease 2019 (COVID-19) outbreak. *Journal of Sleep Research*, e13142. <https://doi.org/10.1111/jsr.13142>

Lorentz, M. S., Chagas, L. B., Perez, A. V., da Silva Cassol, P. A., Vettorazzi, J., & Lubianca, J. N. (2021). Correlation between depressive symptoms and sexual dysfunction in postpartum women during the COVID-19 pandemic. *European Journal of Obstetrics, Gynecology, and Reproductive Biology*, *258*, 162-167. <https://doi.org/10.1016/j.ejogrb.2020.12.039>

López-Morales, H., Del Valle, M. V., Canet-Juric, L., Andrés, M. L., Galli, J. I., Poó, F., & Urquijo, S. (2020). Mental health of pregnant women during the COVID-19 pandemic: A longitudinal study. *Psychiatry Research*, 113567. <https://doi.org/10.1016/j.psychres.2020.113567>

Maggs, J. L., Cassinat, J. R., Kelly, B. C., Mustillo, S. A., & Whiteman, S. D. (2021). Parents Who First Allowed Adolescents to Drink Alcohol in a Family Context During Spring 2020 COVID-19 Emergency Shutdowns. *Journal of Adolescent Health*, *68*(4), 816-818. <https://doi.org/10.1016/j.jadohealth.2021.01.010>

Mahajan, N. N., Ansari, M., Gaikwad, C., Jadhav, P., Tirkey, D., Pophalkar, M. P., . . . Gajbhiye, R. K. (2020). Impact of SARS-CoV-2 on multiple gestation pregnancy. *International Journal of Gynaecology and Obstetrics*. <https://doi.org/10.1002/ijgo.13508>

Maharlouei, N., Keshavarz, P., Salemi, N., & Lankarani, K. B. (2021). Depression and anxiety among pregnant mothers in the initial stage of the Coronavirus Disease (COVID-19) pandemic in the southwest of Iran. *Reprod Health*, *18*(1), 111. <https://doi.org/10.1186/s12978-021-01167-y>

Mahmoud, A. B., Hack-Polay, D., Fuxman, L., & Nicoletti, M. (2021). The Janus-faced effects of COVID-19 perceptions on family healthy eating behavior: Parent's negative experience as a mediator and gender as a moderator. *Scandinavian Journal of Psychology*. <https://doi.org/10.1111/sjop.12742>

Malkawi, S. H., Almhdawi, K., Jaber, A. F., & Alqatarneh, N. S. (2020). COVID-19 Quarantine-Related Mental Health Symptoms and their Correlates among Mothers: A Cross Sectional Study. *Matern Child Health J*, 1-11. <https://doi.org/10.1007/s10995-020-03034-x>

Mangiavacchi, L., Piccoli, L., & Pieroni, L. (2021). Fathers matter: Intrahousehold responsibilities and children's wellbeing during the COVID-19 lockdown in Italy. *Economics and Human Biology*, *42*, 101016. <https://doi.org/10.1016/j.ehb.2021.101016>

Mangolian Shahrbabaki, P., Dehghan, M., Maazallahi, M., & Asadi, N. (2021). Fear and anxiety in girls aged 7 to 11 years old and related factors during the coronavirus pandemic. *Clinical Child Psychology and Psychiatry*, 13591045211013873. <https://doi.org/10.1177/13591045211013873>

Manja, S. A., Mohamad, I., Ismail, H., & Yusof, N. I. (2020). COVID-19: The investigation on the emotional parental burnout during movement control order in Malaysia. *European Journal of Molecular and Clinical Medicine*, *7*(2), 4912-4929.

Mappa, I., Distefano, F. A., & Rizzo, G. (2020). Effects of coronavirus 19 pandemic on maternal anxiety during pregnancy: a prospectic observational study. *Journal of Perinatal Medicine*, *48*(6), 545-550. <https://doi.org/10.1515/jpm-2020-0182>

Marchetti, D., Fontanesi, L., Di Giandomenico, S., Mazza, C., Roma, P., & Verrocchio, M. C. (2020). The Effect of Parent Psychological Distress on Child Hyperactivity/Inattention During the COVID-19 Lockdown: Testing the Mediation of Parent Verbal Hostility and Child Emotional Symptoms. *Frontiers in Psychology*, *11*, 567052. <https://doi.org/10.3389/fpsyg.2020.567052>

Marchetti, D., Fontanesi, L., Mazza, C., Di Giandomenico, S., Roma, P., & Verrocchio, M. C. (2020). Parenting-Related Exhaustion During the Italian COVID-19 Lockdown. *Journal of Pediatric Psychology*, *45*(10), 1114-1123. <https://doi.org/10.1093/jpepsy/jsaa093>

Mariño-Narvaez, C., Puertas-Gonzalez, J. A., Romero-Gonzalez, B., & Peralta-Ramirez, M. I. (2021). Giving birth during the COVID-19 pandemic: The impact on birth satisfaction and postpartum depression. *International Journal of Gynaecology and Obstetrics*, *153*(1), 83-88. <https://doi.org/10.1002/ijgo.13565>

Markovic, A., Mühlematter, C., Beaugrand, M., Camos, V., & Kurth, S. (2021). Severe effects of the COVID-19 confinement on young children's sleep: A longitudinal study identifying risk and protective factors. *Journal of Sleep Research*, e13314. <https://doi.org/10.1111/jsr.13314>

Martins-Filho, P. R., Damascena, N. P., Lage, R. C. M., & Sposato, K. B. (2020). Decrease in child abuse notifications during COVID-19 outbreak: A reason for worry or celebration? *Journal of Paediatrics and Child Health*, *56*(12), 1980-1981. <https://doi.org/10.1111/jpc.15213>

Martínez Pérez, A., López-Soler, C., Fernández-Fernández, V., Alcántara-López, M., & Castro Sáez, M. (2020). Preliminary results of the impact of COVID-19 on children and adolescents exposed to intrafamily abuse. *Terapia Psicologica*, *38*(3), 427-445. <https://doi.org/10.4067/S0718-48082020000300427>

Masters, G. A., Asipenko, E., Bergman, A. L., Person, S. D., Brenckle, L., Moore Simas, T. A., . . . Byatt, N. (2021). Impact of the COVID-19 pandemic on mental health, access to care, and health disparities in the perinatal period. *Journal of Psychiatric Research*, *137*, 126-130. <https://doi.org/10.1016/j.jpsychires.2021.02.056>

Matsushima, M., & Horiguchi, H. (2020). The COVID-19 Pandemic and Mental Well-Being of Pregnant Women in Japan: Need for Economic and Social Policy Interventions. *Disaster Medicine and Public Health Preparedness*, 1-6. <https://doi.org/10.1017/dmp.2020.334>

Mayeur, A., Binois, O., Gallot, V., Hesters, L., Benoit, A., Oppenheimer, A., . . . Sonigo, C. (2020). First follow-up of art pregnancies in the context of the COVID-19 outbreak. *European Journal of Obstetrics, Gynecology, and Reproductive Biology*, *253*, 71-75. <https://doi.org/10.1016/j.ejogrb.2020.07.050>

Mayopoulos, G., Ein-Dor, T., Li, K., Chan, S., & Dekel, S. (2020). Giving birth under hospital visitor restrictions: Heightened acute stress in childbirth in COVID-19 positive women. *Res Sq*. <https://doi.org/10.21203/rs.3.rs-112882/v1>

Mayopoulos, G. A., Ein-Dor, T., Dishy, G. A., Nandru, R., Chan, S. J., Hanley, L. E., . . . Dekel, S. (2021). COVID-19 is associated with traumatic childbirth and subsequent mother-infant bonding problems. *Journal of Affective Disorders*, *282*, 122-125. <https://doi.org/10.1016/j.jad.2020.12.101>

Mazza, C., Marchetti, D., Ricci, E., Fontanesi, L., Di Giandomenico, S., Verrocchio, M. C., & Roma, P. (2021). The COVID-19 lockdown and psychological distress among Italian parents: Influence of parental role, parent personality, and child difficulties. *International Journal of Psychology. Journal International de Psychologie*. <https://doi.org/10.1002/ijop.12755>

Mazza, C., Ricci, E., Marchetti, D., Fontanesi, L., Di Giandomenico, S., Verrocchio, M. C., & Roma, P. (2020). How Personality Relates to Distress in Parents during the Covid-19 Lockdown: The Mediating Role of Child's Emotional and Behavioral Difficulties and the Moderating Effect of Living with Other People. *International Journal of Environmental Research and Public Health*, *17*(17). <https://doi.org/10.3390/ijerph17176236>

McDonald, H. I., Tessier, E., White, J. M., Woodruff, M., Knowles, C., Bates, C., . . . Edelstein, M. (2020). Early impact of the coronavirus disease (COVID-19) pandemic and physical distancing measures on routine childhood vaccinations in England, January to April 2020. *Eurosurveillance*, *25*(19). <https://doi.org/10.2807/1560-7917.ES.2020.25.19.2000848>

McFarland, M. J., McFarland, C. A. S., Hill, T. D., & D'Oria, R. (2021). Postpartum Depressive Symptoms during the Beginning of the COVID-19 Pandemic: An Examination of Population Birth Data from Central New Jersey. *Matern Child Health J*, *25*(3), 353-359. <https://doi.org/10.1007/s10995-020-03116-w>

Meaney, S., Leitao, S., Olander, E. K., Pope, J., & Matvienko-Sikar, K. (2021). The impact of COVID-19 on pregnant womens' experiences and perceptions of antenatal maternity care, social support, and stress-reduction strategies. *Women Birth*. <https://doi.org/10.1016/j.wombi.2021.04.013>

Medina-Jimenez, V., Bermudez-Rojas, M. L., Murillo-Bargas, H., Rivera-Camarillo, A. C., Muñoz-Acosta, J., Ramirez-Abarca, T. G., . . . Martinez-Portilla, R. J. (2020). The impact of the COVID-19 pandemic on depression and stress levels in pregnant women: a national survey during the COVID-19 pandemic in Mexico. *Journal of Maternal-Fetal & Neonatal Medicine*, 1-3. <https://doi.org/10.1080/14767058.2020.1851675>

Mehdizadehkashi, A., Chaichian, S., Haghighi, L., Eshraghi, N., Bordbar, A., Hashemi, N., . . . Tahermanesh, K. (2021). The Impact of COVID-19 Pandemic on Stress and Anxiety of Non-infected Pregnant Mothers. *J Reprod Infertil*, *22*(2), 125-132. <https://doi.org/10.18502/jri.v22i2.5801>

Metz, T. D., Clifton, R. G., Hughes, B. L., Sandoval, G., Saade, G. R., Grobman, W. A., . . . Macones, G. A. (2021). Disease Severity and Perinatal Outcomes of Pregnant Patients With Coronavirus Disease 2019 (COVID-19). *Obstetrics and Gynecology*, *137*(4), 571-580. <https://doi.org/10.1097/aog.0000000000004339>

Milan, S., & Dáu, A. (2021). The Role of Trauma in Mothers' COVID-19 Vaccine Beliefs and Intentions. *Journal of Pediatric Psychology*. <https://doi.org/10.1093/jpepsy/jsab043>

Miller, K. A., Mannix, R., Schmitz, G., Monuteaux, M. C., & Lee, L. K. (2020). Impact of COVID-19 on professional and personal responsibilities of Massachusetts physicians. *American Journal of Emergency Medicine*, *38*(11), 2365-2367. <https://doi.org/10.1016/j.ajem.2020.08.051>

Milne, S. J., Corbett, G. A., Hehir, M. P., Lindow, S. W., Mohan, S., Reagu, S., . . . O'Connell, M. P. (2020). Effects of isolation on mood and relationships in pregnant women during the covid-19 pandemic. *European Journal of Obstetrics, Gynecology, and Reproductive Biology*, *252*, 610-611. <https://doi.org/10.1016/j.ejogrb.2020.06.009>

Mirlashari, J., Ebrahimpour, F., & Salisu, W. J. (2021). War on two fronts: Experience of children with cancer and their family during COVID-19 pandemic in Iran. *Journal of pediatric nursing*, *57*, 25-31.

Mirzaei, N., Jahanian Sadatmahalleh, S., Bahri Khomami, M., Moini, A., & Kazemnejad, A. (2021). Sexual function, mental health, and quality of life under strain of COVID-19 pandemic in Iranian pregnant and lactating women: a comparative cross-sectional study. *Health Qual Life Outcomes*, *19*(1), 66. <https://doi.org/10.1186/s12955-021-01720-0>

Mizrak Sahin, B., & Kabakci, E. N. (2020). The experiences of pregnant women during the COVID-19 pandemic in Turkey: A qualitative study. *Women Birth*. <https://doi.org/10.1016/j.wombi.2020.09.022>

Mo, P. K. H., Fong, V. W. I., Song, B., Di, J., Wang, Q., & Wang, L. (2021). Association of Perceived Threat, Negative Emotions, and Self-Efficacy With Mental Health and Personal Protective Behavior Among Chinese Pregnant Women During the COVID-19 Pandemic: Cross-sectional Survey Study. *Journal of Medical Internet Research*, *23*(4), e24053. <https://doi.org/10.2196/24053>

Molgora, S., & Accordini, M. (2020). Motherhood in the Time of Coronavirus: The Impact of the Pandemic Emergency on Expectant and Postpartum Women's Psychological Well-Being. *Frontiers in Psychology*, *11*, 567155. <https://doi.org/10.3389/fpsyg.2020.567155>

Moore, S. A., Faulkner, G., Rhodes, R. E., Brussoni, M., Chulak-Bozzer, T., Ferguson, L. J., . . . Vanderloo, L. M. (2020). Impact of the COVID-19 virus outbreak on movement and play behaviours of Canadian children and youth: a national survey. *International Journal of Behavioral Nutrition and Physical Activity*, *17*(1), 1-11.

Morelli, M., Cattelino, E., Baiocco, R., Trumello, C., Babore, A., Candelori, C., & Chirumbolo, A. (2020). Parents and Children During the COVID-19 Lockdown: The Influence of Parenting Distress and Parenting Self-Efficacy on Children's Emotional Well-Being. *Frontiers in Psychology*, *11*, 584645. <https://doi.org/10.3389/fpsyg.2020.584645>

Mortazavi, F., Mehrabadi, M., & KiaeeTabar, R. (2021). Pregnant women's well-being and worry during the COVID-19 pandemic: a cross-sectional study. *BMC Pregnancy and Childbirth*, *21*(1), 59. <https://doi.org/10.1186/s12884-021-03548-4>

Moscardino, U., Dicataldo, R., Roch, M., Carbone, M., & Mammarella, I. C. (2021). Parental stress during COVID-19: A brief report on the role of distance education and family resources in an Italian sample. *Current Psychology (New Brunswick, N.J.)*, 1-4. <https://doi.org/10.1007/s12144-021-01454-8>

Mousavi, S. F. (2020). Psychological Well-Being, Marital Satisfaction, and Parental Burnout in Iranian Parents: The Effect of Home Quarantine During COVID-19 Outbreaks. *Frontiers in Psychology*, *11*, 553880. <https://doi.org/10.3389/fpsyg.2020.553880>

Moyer, C. A., Compton, S. D., Kaselitz, E., & Muzik, M. (2020). Pregnancy-related anxiety during COVID-19: a nationwide survey of 2740 pregnant women. *Arch Womens Ment Health*, 1-9. <https://doi.org/10.1007/s00737-020-01073-5>

Moyer, C. A., Sakyi, K. S., Sacks, E., Compton, S. D., Lori, J. R., & Williams, J. E. O. (2020). COVID-19 is increasing Ghanaian pregnant women's anxiety and reducing healthcare seeking. *International Journal of Gynaecology and Obstetrics*. <https://doi.org/10.1002/ijgo.13487>

Muldoon, K. A., Denize, K. M., Talarico, R., Boisvert, C., Frank, O., Harvey, A. L. J., . . . El-Chaar, D. (2021). COVID-19 and perinatal intimate partner violence: a cross-sectional survey of pregnant and postpartum individuals in the early stages of the COVID-19 pandemic. *BMJ Open*, *11*(5), e049295. <https://doi.org/10.1136/bmjopen-2021-049295>

Mullins, E., Hudak, M. L., Banerjee, J., Getzlaff, T., Townson, J., Barnette, K., . . . Lees, C. C. (2021). Pregnancy and neonatal outcomes of COVID-19: coreporting of common outcomes from PAN-COVID and AAP-SONPM registries. *Ultrasound in Obstetrics and Gynecology*, *57*(4), 573-581. <https://doi.org/10.1002/uog.23619>

Mumbardó-Adam, C., Barnet-López, S., & Balboni, G. (2021). How have youth with Autism Spectrum Disorder managed quarantine derived from COVID-19 pandemic? An approach to families perspectives. *Research in Developmental Disabilities*, *110*, 103860. <https://doi.org/10.1016/j.ridd.2021.103860>

Muniraman, H., Ali, M., Cawley, P., Hillyer, J., Heathcote, A., Ponnusamy, V., . . . Clarke, P. (2020). Parental perceptions of the impact of neonatal unit visitation policies during COVID-19 pandemic. *BMJ Paediatrics Open*, *4*(1). <https://doi.org/10.1136/bmjpo-2020-000899>

Myers, S., & Emmott, E. H. (2021). Communication Across Maternal Social Networks During England's First National Lockdown and Its Association With Postnatal Depressive Symptoms. *Frontiers in Psychology*, *12*, 648002. <https://doi.org/10.3389/fpsyg.2021.648002>

Naghizadeh, S., Mirghafourvand, M., & Mohammadirad, R. (2021). Domestic violence and its relationship with quality of life in pregnant women during the outbreak of COVID-19 disease. *BMC Pregnancy and Childbirth*, *21*(1), 88. <https://doi.org/10.1186/s12884-021-03579-x>

Nanjundaswamy, M. H., Shiva, L., Desai, G., Ganjekar, S., Kishore, T., Ram, U., . . . Chandra, P. S. (2020). COVID-19-related anxiety and concerns expressed by pregnant and postpartum women-a survey among obstetricians. *Arch Womens Ment Health*, 1-4. <https://doi.org/10.1007/s00737-020-01060-w>

Nassar, M. F., Allam, M. F., & Shata, M. O. (2021). Effect of COVID-19 Lockdown on Young Egyptian Soccer Players. *Glob Pediatr Health*, *8*, 2333794x211012980. <https://doi.org/10.1177/2333794x211012980>

Nastro, F. F., Tolone, C., Serra, M. R., Pacella, D., Campanozzi, A., & Strisciuglio, C. (2020). Prevalence of functional gastrointestinal disorders in children with celiac disease during the COVID-19 lockdown. *Digestive and Liver Disease*, *52*(10), 1082-1084. <https://doi.org/10.1016/j.dld.2020.06.030>

Naurin, E., Markstedt, E., Stolle, D., Enström, D., Wallin, A., Andreasson, I., . . . Sengpiel, V. (2020). Pregnant under the pressure of a pandemic: a large-scale longitudinal survey before and during the COVID-19 outbreak. *European Journal of Public Health*. <https://doi.org/10.1093/eurpub/ckaa223>

Neece, C., McIntyre, L. L., & Fenning, R. (2020). Examining the impact of COVID-19 in ethnically diverse families with young children with intellectual and developmental disabilities. *Journal of Intellectual Disability Research*, *64*(10), 739-749. <https://doi.org/10.1111/jir.12769>

Neubauer, A. B., Schmidt, A., Kramer, A. C., & Schmiedek, F. (2021). A Little Autonomy Support Goes a Long Way: Daily Autonomy-Supportive Parenting, Child Well-Being, Parental Need Fulfillment, and Change in Child, Family, and Parent Adjustment Across the Adaptation to the COVID-19 Pandemic. *Child Development*. <https://doi.org/10.1111/cdev.13515>

Ng, Q. J., Koh, K. M., Tagore, S., & Mathur, M. (2020). Perception and Feelings of Antenatal Women during COVID-19 Pandemic: A Cross-Sectional Survey. *Ann Acad Med Singap*, *49*(8), 543-552.

Nguyen, P. H., Kachwaha, S., Pant, A., Tran, L. M., Ghosh, S., Sharma, P. K., . . . Menon, P. (2021). Impact of COVID-19 on household food insecurity and interlinkages with child feeding practices and coping strategies in Uttar Pradesh, India: a longitudinal community-based study. *BMJ Open*, *11*(4), e048738. <https://doi.org/10.1136/bmjopen-2021-048738>

Nicholson, E., McDonnell, T., Conlon, C., Barrett, M., Cummins, F., Hensey, C., & McAuliffe, E. (2020). Parental hesitancy and concerns around accessing paediatric unscheduled healthcare during covid-19: A cross-sectional survey. *International Journal of Environmental Research and Public Health*, *17*(24), 1-19. <https://doi.org/10.3390/ijerph17249264>

Niela-Vilén, H., Auxier, J., Ekholm, E., Sarhaddi, F., Asgari Mehrabadi, M., Mahmoudzadeh, A., . . . Axelin, A. (2021). Pregnant women's daily patterns of well-being before and during the COVID-19 pandemic in Finland: Longitudinal monitoring through smartwatch technology. *PloS One*, *16*(2), e0246494. <https://doi.org/10.1371/journal.pone.0246494>

Nodoushan, R. J., Alimoradi, H., & Nazari, M. (2020). Spiritual Health and Stress in Pregnant Women During the Covid-19 Pandemic. *SN Compr Clin Med*, 1-7. <https://doi.org/10.1007/s42399-020-00582-9>

Nomura, R., Tavares, I., Ubinha, A. C., Costa, M. L., Opperman, M. L., Brock, M., . . . Br, A. P. S. C. B. A. D. P. S. G. I. C. (2021). Impact of the COVID-19 Pandemic on Maternal Anxiety in Brazil. *J Clin Med*, *10*(4). <https://doi.org/10.3390/jcm10040620>

Norman, M., Navér, L., Söderling, J., Ahlberg, M., Hervius Askling, H., Aronsson, B., . . . Stephansson, O. (2021). Association of Maternal SARS-CoV-2 Infection in Pregnancy With Neonatal Outcomes. *JAMA*, *325*(20), 2076-2086. <https://doi.org/10.1001/jama.2021.5775>

Nurrizka, R. H., Nurdiantami, Y., & Makkiyah, F. A. (2021). Psychological outcomes of the COVID-19 pandemic among pregnant women in Indonesia: a cross-sectional study. *Osong Public Health Res Perspect*, *12*(2), 80-87. <https://doi.org/10.24171/j.phrp.2021.12.2.05>

Odeh, R., Gharaibeh, L., Daher, A., Kussad, S., & Alassaf, A. (2020). Caring for a child with type 1 diabetes during COVID-19 lockdown in a developing country: Challenges and parents' perspectives on the use of telemedicine. *Diabetes Research and Clinical Practice*, *168*, 108393. <https://doi.org/10.1016/j.diabres.2020.108393>

Ogamba, I., Kliss, A., Rainville, N., Chuang, L., Panarelli, E., Petrini, J., & Zilberman, D. (2021). Initial review of pregnancy and neonatal outcomes of pregnant women with COVID-19 infection. *Journal of Perinatal Medicine*, *49*(3), 263-268. <https://doi.org/10.1515/jpm-2020-0446>

Ollivier, R., Aston, D. M., Price, D. S., Sim, D. M., Benoit, D. B., Joy, D. P., . . . Nassaji, N. A. (2021). Mental Health & Parental Concerns during COVID-19: The Experiences of New Mothers Amidst Social Isolation. *Midwifery*, *94*, 102902. <https://doi.org/10.1016/j.midw.2020.102902>

Oncel, M. Y., Akın, I. M., Kanburoglu, M. K., Tayman, C., Coskun, S., Narter, F., . . . Koc, E. (2020). A multicenter study on epidemiological and clinical characteristics of 125 newborns born to women infected with COVID-19 by Turkish Neonatal Society. *European Journal of Pediatrics*, 1-10. <https://doi.org/10.1007/s00431-020-03767-5>

Orsini, A., Corsi, M., Pedrinelli, V., Santangelo, A., Bertelloni, C., Dell'Oste, V., . . . Carmassi, C. (2021). Post-traumatic stress, anxiety, and depressive symptoms in caregivers of children tested for COVID-19 in the acute phase of the Italian outbreak. *Journal of Psychiatric Research*, *135*, 256-263. <https://doi.org/10.1016/j.jpsychires.2021.01.024>

Oskovi-Kaplan, Z. A., Buyuk, G. N., Ozgu-Erdinc, A. S., Keskin, H. L., Ozbas, A., & Moraloglu Tekin, O. (2020). The Effect of COVID-19 Pandemic and Social Restrictions on Depression Rates and Maternal Attachment in Immediate Postpartum Women: a Preliminary Study. *Psychiatric Quarterly*, 1-8. <https://doi.org/10.1007/s11126-020-09843-1>

Ostacoli, L., Cosma, S., Bevilacqua, F., Berchialla, P., Bovetti, M., Carosso, A. R., . . . Benedetto, C. (2020). Psychosocial factors associated with postpartum psychological distress during the Covid-19 pandemic: a cross-sectional study. *BMC Pregnancy and Childbirth*, *20*(1), 703. <https://doi.org/10.1186/s12884-020-03399-5>

Overbeck, G., Rasmussen, I. S., Siersma, V., Andersen, J. H., Kragstrup, J., Wilson, P., . . . Ertmann, R. K. (2021). Depression and anxiety symptoms in pregnant women in Denmark during COVID-19. *Scand J Public Health*, 14034948211013271. <https://doi.org/10.1177/14034948211013271>

Ozturk Eyimaya, A., & Yalçin Irmak, A. (2020). Relationship between parenting practices and children's screen time during the COVID-19 Pandemic in Turkey. *Journal of Pediatric Nursing*, *56*, 24-29. <https://doi.org/10.1016/j.pedn.2020.10.002>

Pariente, G., Wissotzky Broder, O., Sheiner, E., Lanxner Battat, T., Mazor, E., Yaniv Salem, S., . . . Wainstock, T. (2020). Risk for probable post-partum depression among women during the COVID-19 pandemic. *Arch Womens Ment Health*, 1-7. <https://doi.org/10.1007/s00737-020-01075-3>

Parra-Saavedra, M., Villa-Villa, I., Pérez-Olivo, J., Guzman-Polania, L., Galvis-Centurion, P., Cumplido-Romero, Á., . . . Miranda, J. (2020). Attitudes and collateral psychological effects of COVID-19 in pregnant women in Colombia. *International Journal of Gynaecology and Obstetrics*, *151*(2), 203-208. <https://doi.org/10.1002/ijgo.13348>

Pasca, L., Zanaboni, M. P., Grumi, S., Totaro, M., Ballante, E., Varesio, C., & De Giorgis, V. (2021). Impact of COVID-19 pandemic in pediatric patients with epilepsy with neuropsychiatric comorbidities: A telemedicine evaluation. *Epilepsy & Behavior*, *115*, 107519. <https://doi.org/10.1016/j.yebeh.2020.107519>

Paschke, K., Arnaud, N., Austermann, M. I., & Thomasius, R. (2021). Risk factors for prospective increase in psychological stress during COVID-19 lockdown in a representative sample of adolescents and their parents. *BJPsych Open*, *7*(3), e94. <https://doi.org/10.1192/bjo.2021.49>

Patabendige, M., Gamage, M. M., Weerasinghe, M., & Jayawardane, A. (2020). Psychological impact of the COVID-19 pandemic among pregnant women in Sri Lanka. *International Journal of Gynaecology and Obstetrics*, *151*(1), 150-153. <https://doi.org/10.1002/ijgo.13335>

Patrick, S. W., Henkhaus, L. E., Zickafoose, J. S., Lovell, K., Halvorson, A., Loch, S., . . . Davis, M. M. (2020). Well-being of Parents and Children During the COVID-19 Pandemic: A National Survey. *Pediatrics*, *146*(4). <https://doi.org/10.1542/peds.2020-016824>

Pearson, C., Levine, M., Messman, A., Chopra, T., Awali, R., Robb, L., . . . Levine, D. L. (2021). Understanding the Impact of COVID-19 on Physician Moms. *Disaster Medicine and Public Health Preparedness*, 1-17. <https://doi.org/10.1017/dmp.2021.49>

Peltz, J. S., Daks, J. S., & Rogge, R. D. (2020). Mediators of the association between COVID-19-related stressors and parents' psychological flexibility and inflexibility: The roles of perceived sleep quality and energy. *J Contextual Behav Sci*, *17*, 168-176. <https://doi.org/10.1016/j.jcbs.2020.07.001>

Peng, S., Zhang, Y., Liu, H., Huang, X., Noble, D. J., Yang, L., . . . Narayan, A. (2021). A multi-center survey on the postpartum mental health of mothers and attachment to their neonates during COVID-19 in Hubei Province of China. *Ann Transl Med*, *9*(5), 382. <https://doi.org/10.21037/atm-20-6115>

Peng, S., Zhu, H., Yang, L., Cao, L., Huang, X., Dynes, M., . . . Xia, S. (2020). A study of breastfeeding practices, SARS-CoV-2 and its antibodies in the breast milk of mothers confirmed with COVID-19. *Lancet Reg Health West Pac*, *4*, 100045. <https://doi.org/10.1016/j.lanwpc.2020.100045>

Perez, A., Panagiotopoulou, E., Curtis, P., & Roberts, R. (2021). Barriers and facilitators to mood and confidence in pregnancy and early parenthood during COVID-19 in the UK: mixed-methods synthesis survey. *BJPsych Open*, *7*(4), e107. <https://doi.org/10.1192/bjo.2021.925>

Perzow, S. E. D., Hennessey, E. P., Hoffman, M. C., Grote, N. K., Davis, E. P., & Hankin, B. L. (2021). Mental health of pregnant and postpartum women in response to the COVID-19 pandemic. *J Affect Disord Rep*, *4*, 100123. <https://doi.org/10.1016/j.jadr.2021.100123>

Petrocchi, S., Levante, A., Bianco, F., Castelli, I., & Lecciso, F. (2020). Maternal Distress/Coping and Children's Adaptive Behaviors During the COVID-19 Lockdown: Mediation Through Children's Emotional Experience. *Front Public Health*, *8*, 587833. <https://doi.org/10.3389/fpubh.2020.587833>

Philippe, K., Chabanet, C., Issanchou, S., & Monnery-Patris, S. (2021). Child eating behaviors, parental feeding practices and food shopping motivations during the COVID-19 lockdown in France: (How) did they change? *Appetite*, *161*, 105132. <https://doi.org/10.1016/j.appet.2021.105132>

Pierce-Williams, R. A. M., Burd, J., Felder, L., Khoury, R., Bernstein, P. S., Avila, K., . . . Berghella, V. (2020). Clinical course of severe and critical coronavirus disease 2019 in hospitalized pregnancies: a United States cohort study. *Am J Obstet Gynecol MFM*, *2*(3), 100134. <https://doi.org/10.1016/j.ajogmf.2020.100134>

Pınar Senkalfa, B., Sismanlar Eyuboglu, T., Aslan, A. T., Ramaslı Gursoy, T., Soysal, A. S., Yapar, D., & İlhan, M. N. (2020). Effect of the COVID-19 pandemic on anxiety among children with cystic fibrosis and their mothers. *Pediatric Pulmonology*, *55*(8), 2128-2134. <https://doi.org/10.1002/ppul.24900>

Pope, J., Olander, E. K., Leitao, S., Meaney, S., & Matvienko-Sikar, K. (2021). Prenatal stress, health, and health behaviours during the COVID-19 pandemic: An international survey. *Women Birth*. <https://doi.org/10.1016/j.wombi.2021.03.007>

Popofsky, S., Noor, A., Leavens-Maurer, J., Quintos-Alagheband, M. L., Mock, A., Vinci, A., . . . Krilov, L. (2020). Impact of Maternal Severe Acute Respiratory Syndrome Coronavirus 2 Detection on Breastfeeding Due to Infant Separation at Birth. *Journal of Pediatrics*, *226*, 64-70. <https://doi.org/10.1016/j.jpeds.2020.08.004>

Poulain, T., Meigen, C., Sobek, C., Ober, P., Igel, U., Körner, A., . . . Vogel, M. (2021). Loss of childcare and classroom teaching during the Covid-19-related lockdown in spring 2020: A longitudinal study on consequences on leisure behavior and schoolwork at home. *PloS One*, *16*(3), e0247949. <https://doi.org/10.1371/journal.pone.0247949>

Preis, H., Mahaffey, B., Heiselman, C., & Lobel, M. (2020a). Pandemic-related pregnancy stress and anxiety among women pregnant during the coronavirus disease 2019 pandemic. *Am J Obstet Gynecol MFM*, *2*(3), 100155. <https://doi.org/10.1016/j.ajogmf.2020.100155>

Preis, H., Mahaffey, B., Heiselman, C., & Lobel, M. (2020b). Vulnerability and resilience to pandemic-related stress among U.S. women pregnant at the start of the COVID-19 pandemic. *Social Science and Medicine*, *266*, 113348. <https://doi.org/10.1016/j.socscimed.2020.113348>

Preis, H., Mahaffey, B., & Lobel, M. (2020). Psychometric properties of the Pandemic-Related Pregnancy Stress Scale (PREPS). *Journal of Psychosomatic Obstetrics and Gynaecology*, *41*(3), 191-197. <https://doi.org/10.1080/0167482x.2020.1801625>

Preis, H., Mahaffey, B., & Lobel, M. (2021). The role of pandemic-related pregnancy stress in preference for community birth during the beginning of the COVID-19 pandemic in the United States. *Birth*, *48*(2), 242-250. <https://doi.org/10.1111/birt.12533>

Preis, H., Mahaffey, B., Pati, S., Heiselman, C., & Lobel, M. (2021). Adverse Perinatal Outcomes Predicted by Prenatal Maternal Stress Among U.S. Women at the COVID-19 Pandemic Onset. *Annals of Behavioral Medicine*, *55*(3), 179-191. <https://doi.org/10.1093/abm/kaab005>

Preuss, H., Capito, K., van Eickels, R. L., Zemp, M., & Kolar, D. R. (2021). Cognitive reappraisal and self-compassion as emotion regulation strategies for parents during COVID-19: An online randomized controlled trial. *Internet Interv*, *24*, 100388. <https://doi.org/10.1016/j.invent.2021.100388>

Prikhidko, A., Long, H., & Wheaton, M. G. (2020). The Effect of Concerns About COVID-19 on Anxiety, Stress, Parental Burnout, and Emotion Regulation: The Role of Susceptibility to Digital Emotion Contagion. *Front Public Health*, *8*, 567250. <https://doi.org/10.3389/fpubh.2020.567250>

Protudjer, J. L. P., Golding, M., Salisbury, M. R., Abrams, E. M., & Roos, L. E. (2020). High anxiety and health-related quality of life in families with children with food allergy during coronavirus disease 2019. *Annals of Allergy, Asthma, and Immunology*, *126*(1), 83-88.e81. <https://doi.org/10.1016/j.anai.2020.09.010>

Puertas-Gonzalez, J. A., Mariño-Narvaez, C., Peralta-Ramirez, M. I., & Romero-Gonzalez, B. (2021). The psychological impact of the COVID-19 pandemic on pregnant women. *Psychiatry Research*, *301*, 113978. <https://doi.org/10.1016/j.psychres.2021.113978>

Qi, M., Li, X., Liu, S., Li, Y., & Huang, W. (2020). Impact of the COVID-19 epidemic on patterns of pregnant women's perception of threat and its relationship to mental state: A latent class analysis. *PloS One*, *15*(10), e0239697. <https://doi.org/10.1371/journal.pone.0239697>

Quandt, S. A., LaMonto, N. J., Mora, D. C., Talton, J. W., Laurienti, P. J., & Arcury, T. A. (2020). COVID-19 Pandemic Among Immigrant Latinx Farmworker and Non-farmworker Families: A Rural-Urban Comparison of Economic, Educational, Healthcare, and Immigration Concerns. *medRxiv*. <https://doi.org/10.1101/2020.10.30.20223156>

Quenzer-Alfred, C., Schneider, L., Soyka, V., Harbrecht, M., Blume, V., & Mays, D. (2021). No nursery ‘til school–the transition to primary school without institutional transition support due to the COVID-19 shutdown in Germany. *European Journal of Special Needs Education*, *36*(1), 127-141. <https://doi.org/10.1080/08856257.2021.1872850>

Quílez-Robres, A., Lozano-Blasco, R., Íñiguez-Berrozpe, T., & Cortés-Pascual, A. (2021). Social, Family, and Educational Impacts on Anxiety and Cognitive Empathy Derived From the COVID-19: Study on Families With Children. *Frontiers in Psychology*, *12*, 562800. <https://doi.org/10.3389/fpsyg.2021.562800>

Racine, N., Hetherington, E., McArthur, B. A., McDonald, S., Edwards, S., Tough, S., & Madigan, S. (2021). Maternal depressive and anxiety symptoms before and during the COVID-19 pandemic in Canada: a longitudinal analysis. *Lancet Psychiatry*, *8*(5), 405-415. <https://doi.org/10.1016/s2215-0366(21)00074-2>

Ravaldi, C., Ricca, V., Wilson, A., Homer, C., & Vannacci, A. (2020). Previous psychopathology predicted severe COVID-19 concern, anxiety, and PTSD symptoms in pregnant women during "lockdown" in Italy. *Arch Womens Ment Health*, 1-4. <https://doi.org/10.1007/s00737-020-01086-0>

Ravaldi, C., Wilson, A., Ricca, V., Homer, C., & Vannacci, A. (2020). Pregnant women voice their concerns and birth expectations during the COVID-19 pandemic in Italy. *Women Birth*. <https://doi.org/10.1016/j.wombi.2020.07.002>

Ravens-Sieberer, U., Kaman, A., Otto, C., Adedeji, A., Devine, J., Erhart, M., . . . Hurrelmann, K. (2020). Mental health and quality of life in children and adolescents during the COVID-19 pandemic—results of the copsy study. *Deutsches Arzteblatt International*, *117*, 828-829. <https://doi.org/10.3238/arztebl.2020.0828>

Raviv, T., Warren, C. M., Washburn, J. J., Kanaley, M. K., Eihentale, L., Goldenthal, H. J., . . . Gupta, R. (2021). Caregiver Perceptions of Children's Psychological Well-being During the COVID-19 Pandemic. *JAMA Netw Open*, *4*(4), e2111103. <https://doi.org/10.1001/jamanetworkopen.2021.11103>

Recto, P., & Lesser, J. (2020). Young Hispanic fathers during COVID-19: Balancing parenthood, finding strength, and maintaining hope. *Public Health Nursing*. <https://doi.org/10.1111/phn.12857>

Ren, J., Li, X., Chen, S., & Nie, Y. (2020). The Influence of Factors Such as Parenting Stress and Social Support on the State Anxiety in Parents of Special Needs Children During the COVID-19 Epidemic. *Frontiers in Psychology*, *11*, 565393. <https://doi.org/10.3389/fpsyg.2020.565393>

Rhodes, A., Kheireddine, S., & Smith, A. D. (2020). A mixed methods investigation into the experiences, attitudes and needs of Baby Buddy pregnancy and parenting app users during the COVID-19 pandemic. *JMIR Mhealth Uhealth*. <https://doi.org/10.2196/23157>

Ribeiro, F. S., Braun Janzen, T., Passarini, L., & Vanzella, P. (2021). Exploring Changes in Musical Behaviors of Caregivers and Children in Social Distancing During the COVID-19 Outbreak. *Frontiers in Psychology*, *12*, 633499. <https://doi.org/10.3389/fpsyg.2021.633499>

Rice, K., & Williams, S. (2021). Women's postpartum experiences in Canada during the COVID-19 pandemic: a qualitative study. *CMAJ Open*, *9*(2), E556-e562. <https://doi.org/10.9778/cmajo.20210008>

Rocha, H. A., Sudfeld, C. R., Leite Á, J., Rocha, S. G., Machado, M. M., Campos, J. S., . . . Correia, L. L. (2021). Coronavirus disease 2019, food security and maternal mental health in Ceará, Brazil: a repeated cross-sectional survey. *Public Health Nutrition*, *24*(7), 1836-1840. <https://doi.org/10.1017/s1368980021000628>

Rodriguez, C. M., Lee, S. J., Ward, K. P., & Pu, D. F. (2021). The Perfect Storm: Hidden Risk of Child Maltreatment During the Covid-19 Pandemic. *Child Maltreat*, *26*(2), 139-151. <https://doi.org/10.1177/1077559520982066>

Rogers, G., Perez-Olivas, G., Stenfert Kroese, B., Patel, V., Murphy, G., Rose, J., . . . Willner, P. (2021). The experiences of mothers of children and young people with intellectual disabilities during the first COVID-19 lockdown period. *J Appl Res Intellect Disabil*. <https://doi.org/10.1111/jar.12884>

Romero, E., López-Romero, L., Domínguez-Álvarez, B., Villar, P., & Gómez-Fraguela, J. A. (2020). Testing the effects of COVID-19 confinement in Spanish children: The role of parents’ distress, emotional problems and specific parenting. *International journal of environmental research and public health*, *17*(19), 6975.

Romero-Gonzalez, B., Puertas-Gonzalez, J. A., Mariño-Narvaez, C., & Peralta-Ramirez, M. I. (2020). Confinement variables by COVID-19 predictors of anxious and depressive symptoms in pregnant women. *Medicina Clínica*. <https://doi.org/10.1016/j.medcli.2020.10.002>

Ronchi, A., Pietrasanta, C., Zavattoni, M., Saruggia, M., Schena, F., Sinelli, M. T., . . . Pugni, L. (2021). Evaluation of Rooming-in Practice for Neonates Born to Mothers With Severe Acute Respiratory Syndrome Coronavirus 2 Infection in Italy. *JAMA Pediatr*, *175*(3), 260-266. <https://doi.org/10.1001/jamapediatrics.2020.5086>

Roos, L. E., Salisbury, M., Penner-Goeke, L., Cameron, E. E., Protudjer, J. L. P., Giuliano, R., . . . Reynolds, K. (2021). Supporting families to protect child health: Parenting quality and household needs during the COVID-19 pandemic. *PloS One*, *16*(5), e0251720. <https://doi.org/10.1371/journal.pone.0251720>

Rosen, H., Bart, Y., Zlatkin, R., Ben-Sira, L., Bashat, D. B., Amit, S., . . . Yinon, Y. (2021). Fetal and perinatal outcome following first and second trimester covid-19 infection: Evidence from a prospective cohort study. *Journal of Clinical Medicine*, *10*(10). <https://doi.org/10.3390/jcm10102152>

Rudrum, S. (2021). Pregnancy During the Global COVID-19 Pandemic: Canadian Experiences of Care. *Front Sociol*, *6*, 611324. <https://doi.org/10.3389/fsoc.2021.611324>

Russell, B. S., Hutchison, M., Tambling, R., Tomkunas, A. J., & Horton, A. L. (2020). Initial Challenges of Caregiving During COVID-19: Caregiver Burden, Mental Health, and the Parent-Child Relationship. *Child Psychiatry and Human Development*, *51*(5), 671-682. <https://doi.org/10.1007/s10578-020-01037-x>

Saadati, N., Afshari, P., Boostani, H., Beheshtinasab, M., Abedi, P., & Maraghi, E. (2021). Health anxiety and related factors among pregnant women during the COVID-19 pandemic: a cross-sectional study from Iran. *BMC Psychiatry*, *21*(1), 95. <https://doi.org/10.1186/s12888-021-03092-7>

Sade, S., Sheiner, E., Wainstock, T., Hermon, N., Yaniv Salem, S., Kosef, T., . . . Pariente, G. (2020). Risk for Depressive Symptoms among Hospitalized Women in High-Risk Pregnancy Units during the COVID-19 Pandemic. *J Clin Med*, *9*(8). <https://doi.org/10.3390/jcm9082449>

Sahin, B., & Ozturk, D. M. (2021). Evaluation of the level of anxiety among pregnant women during the outbreak of Covid-19. *Journal of Experimental and Clinical Medicine (Turkey)*, *38*(2), 143-149. <https://doi.org/10.52142/omujecm.38.2.16>

Sahithya, B. R., Kashyap, R. S., & Roopesh, B. N. (2020). Perceived stress, parental stress, and parenting during covid-19 lockdown: A preliminary study. *Journal of Indian Association for Child and Adolescent Mental Health*, *16*(4), 44-63.

Sakalidis, V. S., Rea, A., Perrella, S. L., McEachran, J., Collis, G., Miraudo, J., . . . Geddes, D. T. (2021). Wellbeing of Breastfeeding Women in Australia and New Zealand during the COVID-19 Pandemic: A Cross-Sectional Study. *Nutrients*, *13*(6). <https://doi.org/10.3390/nu13061831>

Salehi, L., Rahimzadeh, M., Molaei, E., Zaheri, H., & Esmaelzadeh-Saeieh, S. (2020). The relationship among fear and anxiety of COVID-19, pregnancy experience, and mental health disorder in pregnant women: A structural equation model. *Brain Behav*, *10*(11), e01835. <https://doi.org/10.1002/brb3.1835>

Salmi, H., Heinonen, S., Hästbacka, J., Lääperi, M., Rautiainen, P., Miettinen, P. J., . . . Knip, M. (2021). New-onset type 1 diabetes in Finnish children during the COVID-19 pandemic. *Archives of Disease in Childhood*. <https://doi.org/10.1136/archdischild-2020-321220>

Sama, B. K., Kaur, P., Thind, P. S., Verma, M. K., Kaur, M., & Singh, D. D. (2021). Implications of COVID‐19‐induced nationwide lockdown on children's behaviour in Punjab, India. *Child: care, health and development*, *47*(1), 128-135.

Sbrilli, M. D., Haigler, K., & Laurent, H. K. (2021). The Indirect Effect of Parental Intolerance of Uncertainty on Perinatal Mental Health via Mindfulness During COVID-19. *Mindfulness (N Y)*, 1-10. <https://doi.org/10.1007/s12671-021-01657-x>

Scala, M., Marchman, V. A., Brignoni-Pérez, E., Morales, M. C., & Travis, K. E. (2020). Impact of the COVID-19 pandemic on developmental care practices for infants born preterm. *medRxiv*. <https://doi.org/10.1101/2020.11.25.20238956>

Scarpellini, F., Segre, G., Cartabia, M., Zanetti, M., Campi, R., Clavenna, A., & Bonati, M. (2021). Distance learning in Italian primary and middle school children during the COVID-19 pandemic: a national survey. *BMC Public Health*, *21*(1), 1035. <https://doi.org/10.1186/s12889-021-11026-x>

Shafer, K., Scheibling, C., & Milkie, M. A. (2020). The Division of Domestic Labor before and during the COVID-19 Pandemic in Canada: Stagnation versus Shifts in Fathers' Contributions. *Can Rev Sociol*, *57*(4), 523-549. <https://doi.org/10.1111/cars.12315>

Shah, R., Raju, V. V., Sharma, A., & Grover, S. (2021). Impact of COVID-19 and Lockdown on Children with ADHD and Their Families-An Online Survey and a Continuity Care Model. *Journal of Neurosciences in Rural Practice*, *12*(1), 71-79. <https://doi.org/10.1055/s-0040-1718645>

Shahid, A., Javed, A., Rehman, S., Tariq, R., Ikram, M., & Suhail, M. (2020). Evaluation of psychological impact, depression, and anxiety among pregnant women during the COVID-19 pandemic in Lahore, Pakistan. *International Journal of Gynaecology and Obstetrics*, *151*(3), 462-465. <https://doi.org/10.1002/ijgo.13398>

Shangguan, F., Wang, R., Quan, X., Zhou, C., Zhang, C., Qian, W., . . . Zhang, X. Y. (2021). Association of Stress-Related Factors With Anxiety Among Chinese Pregnant Participants in an Online Crisis Intervention During COVID-19 Epidemic. *Frontiers in Psychology*, *12*, 633765. <https://doi.org/10.3389/fpsyg.2021.633765>

Shayganfard, M., Mahdavi, F., Haghighi, M., Sadeghi Bahmani, D., & Brand, S. (2020). Health Anxiety Predicts Postponing or Cancelling Routine Medical Health Care Appointments among Women in Perinatal Stage during the Covid-19 Lockdown. *International Journal of Environmental Research and Public Health*, *17*(21). <https://doi.org/10.3390/ijerph17218272>

Sheridan, D. C., Cloutier, R., Johnson, K., & Marshall, R. (2021). Where have all the emergency paediatric mental health patients gone during COVID-19? *Acta Paediatrica, International Journal of Paediatrics*, *110*(2), 598-599. <https://doi.org/10.1111/apa.15537>

Shinomiya, Y., Yoshizaki, A., Murata, E., Fujisawa, T. X., Taniike, M., & Mohri, I. (2021). Sleep and the General Behavior of Infants and Parents during the Closure of Schools as a Result of the COVID-19 Pandemic: Comparison with 2019 Data. *Children (Basel)*, *8*(2). <https://doi.org/10.3390/children8020168>

Shockley, K. M., Clark, M. A., Dodd, H., & King, E. B. (2020). Work-family strategies during COVID-19: Examining gender dynamics among dual-earner couples with young children. *Journal of Applied Psychology*. <https://doi.org/10.1037/apl0000857>

Shreffler, K. M., Joachims, C. N., Tiemeyer, S., Simmons, W. K., Teague, T. K., & Hays-Grudo, J. (2021). Childhood Adversity and Perceived Distress from the COVID-19 Pandemic. *Advers Resil Sci*, 1-4. <https://doi.org/10.1007/s42844-021-00030-0>

Shrestha, D., Saha, R., Manandhar, N., Adhikari, A., & Dahal, J. (2021). Anxiety among pregnant women about corona virus infections during covid-19 pandemic at a tertiary care center in nepal: A descriptive cross-sectional study. *Journal of the Nepal Medical Association*, *59*(234), 152-155. <https://doi.org/10.31729/jnma.5377>

Siegle, C. B. H., Pombo, A., Luz, C., Rodrigues, L. P., Cordovil, R., & dos Santos Cardoso de Sá, C. (2020). Influences of family and household characteristics on children's level of physical activity during social distancing due to covid-19 in Brazil. *Revista Paulista de Pediatria*, *39*. <https://doi.org/10.1590/1984-0462/2021/39/2020297>

Silverman, M. E., Burgos, L., Rodriguez, Z. I., Afzal, O., Kalishman, A., Callipari, F., . . . Loudon, H. (2020). Postpartum mood among universally screened high and low socioeconomic status patients during COVID-19 social restrictions in New York City. *Scientific Reports*, *10*(1), 22380. <https://doi.org/10.1038/s41598-020-79564-9>

Sinaci, S., Ozden Tokalioglu, E., Ocal, D., Atalay, A., Yilmaz, G., Keskin, H. L., . . . Moraloglu Tekin, O. (2020). Does having a high-risk pregnancy influence anxiety level during the COVID-19 pandemic? *European Journal of Obstetrics, Gynecology, and Reproductive Biology*, *255*, 190-196. <https://doi.org/10.1016/j.ejogrb.2020.10.055>

Smith, C. L., Waters, S. F., Spellacy, D., Burduli, E., Brooks, O., Carty, C. L., . . . Barbosa-Leiker, C. (2021). Substance use and mental health in pregnant women during the COVID-19 pandemic. *Journal of Reproductive and Infant Psychology*, 1-14. <https://doi.org/10.1080/02646838.2021.1916815>

Solís-García, G., Gutiérrez-Vélez, A., Pescador Chamorro, I., Zamora-Flores, E., Vigil-Vázquez, S., Rodríguez-Corrales, E., & Sánchez-Luna, M. (2021). [Epidemiology, management and risk of SARS-CoV-2 transmission in a cohort of newborns born to mothers diagnosed with COVID-19 infection]. *An Pediatr (Engl Ed)*, *94*(3), 173-178. <https://doi.org/10.1016/j.anpedi.2020.12.004>

Spinelli, M., Lionetti, F., Pastore, M., & Fasolo, M. (2020). Parents' Stress and Children's Psychological Problems in Families Facing the COVID-19 Outbreak in Italy. *Frontiers in Psychology*, *11*, 1713. <https://doi.org/10.3389/fpsyg.2020.01713>

Spinola, O., Liotti, M., Speranza, A. M., & Tambelli, R. (2020). Effects of COVID-19 Epidemic Lockdown on Postpartum Depressive Symptoms in a Sample of Italian Mothers. *Front Psychiatry*, *11*, 589916. <https://doi.org/10.3389/fpsyt.2020.589916>

Stallard, P., Pereira, A. I., & Barros, L. (2021). Post-traumatic growth during the COVID-19 pandemic in carers of children in Portugal and the UK: cross-sectional online survey. *BJPsych Open*, *7*(1), e37. <https://doi.org/10.1192/bjo.2021.1>

Steinberg, S., Liu, T., & Lense, M. D. (2021). Musical Engagement and Parent-Child Attachment in Families With Young Children During the Covid-19 Pandemic. *Frontiers in Psychology*, *12*, 641733. <https://doi.org/10.3389/fpsyg.2021.641733>

Stepowicz, A., Wencka, B., Bieńkiewicz, J., Horzelski, W., & Grzesiak, M. (2020). Stress and Anxiety Levels in Pregnant and Post-Partum Women during the COVID-19 Pandemic. *International Journal of Environmental Research and Public Health*, *17*(24). <https://doi.org/10.3390/ijerph17249450>

Stojanov, J., Stankovic, M., Zikic, O., & Stojanov, A. (2020). The risk for nonpsychotic postpartum mood and anxiety disorders during the COVID-19 pandemic. *International Journal of Psychiatry in Medicine*, 91217420981533. <https://doi.org/10.1177/0091217420981533>

Suffren, S., Dubois-Comtois, K., Lemelin, J. P., St-Laurent, D., & Milot, T. (2021). Relations between Child and Parent Fears and Changes in Family Functioning Related to COVID-19. *International Journal of Environmental Research and Public Health*, *18*(4). <https://doi.org/10.3390/ijerph18041786>

Suzuki, S. (2020a). Psychological status during the first trimester of pregnancy under the COVID-19 epidemic in Japan. *Journal of Maternal-Fetal & Neonatal Medicine*, 1-2. <https://doi.org/10.1080/14767058.2020.1793319>

Suzuki, S. (2020b). Psychological status of postpartum women under the COVID-19 pandemic in Japan. *Journal of Maternal-Fetal & Neonatal Medicine*, 1-3. <https://doi.org/10.1080/14767058.2020.1763949>

Suárez-Rico, B. V., Estrada-Gutierrez, G., Sánchez-Martínez, M., Perichart-Perera, O., Rodríguez-Hernández, C., González-Leyva, C., . . . Reyes-Muñoz, E. (2021). Prevalence of Depression, Anxiety, and Perceived Stress in Postpartum Mexican Women during the COVID-19 Lockdown. *International Journal of Environmental Research and Public Health*, *18*(9). <https://doi.org/10.3390/ijerph18094627>

Sweet, L., Bradfield, Z., Vasilevski, V., Wynter, K., Hauck, Y., Kuliukas, L., . . . Wilson, A. N. (2021). Becoming a mother in the 'new' social world in Australia during the first wave of the COVID-19 pandemic. *Midwifery*, *98*, 102996. <https://doi.org/10.1016/j.midw.2021.102996>

Syed Anwar Aly, S. A., Abdul Rahman, R., Sharip, S., Shah, S. A., Abdullah Mahdy, Z., & Kalok, A. (2021). Pregnancy and COVID-19 Pandemic Perception in Malaysia: A Cross-Sectional Study. *International Journal of Environmental Research and Public Health*, *18*(11). <https://doi.org/10.3390/ijerph18115762>

Takaku, R., & Yokoyama, I. (2021). What the COVID-19 school closure left in its wake: Evidence from a regression discontinuity analysis in Japan. *J Public Econ*, *195*, 104364. <https://doi.org/10.1016/j.jpubeco.2020.104364>

Talbot, J., Charron, V., & Konkle, A. T. (2021). Feeling the Void: Lack of Support for Isolation and Sleep Difficulties in Pregnant Women during the COVID-19 Pandemic Revealed by Twitter Data Analysis. *International Journal of Environmental Research and Public Health*, *18*(2). <https://doi.org/10.3390/ijerph18020393>

Tambling, R. R., Tomkunas, A. J., Russell, B. S., Horton, A. L., & Hutchison, M. (2021). Thematic Analysis of Parent-Child Conversations About COVID-19: "Playing It Safe". *J Child Fam Stud*, 1-13. <https://doi.org/10.1007/s10826-020-01889-w>

Tang, S., Xiang, M., Cheung, T., & Xiang, Y.-T. (2021). Mental health and its correlates among children and adolescents during COVID-19 school closure: The importance of parent-child discussion. *Journal of affective disorders*, *279*, 353-360.

Taubman-Ben-Ari, O., & Ben-Yaakov, O. (2020). Distress and apprehension among new parents during the COVID-19 pandemic: The contribution of personal resources. *American Journal of Orthopsychiatry*, *90*(6), 810-816. <https://doi.org/10.1037/ort0000497>

Taubman-Ben-Ari, O., Ben-Yaakov, O., & Chasson, M. (2021). Parenting stress among new parents before and during the COVID-19 pandemic. *Child Abuse and Neglect*, *117*, 105080. <https://doi.org/10.1016/j.chiabu.2021.105080>

Taubman-Ben-Ari, O., Chasson, M., Abu Sharkia, S., & Weiss, E. (2020). Distress and anxiety associated with COVID-19 among Jewish and Arab pregnant women in Israel. *Journal of Reproductive and Infant Psychology*, *38*(3), 340-348. <https://doi.org/10.1080/02646838.2020.1786037>

Taubman-Ben-Ari, O., Chasson, M., & Abu-Sharkia, S. (2020). Childbirth anxieties in the shadow of COVID-19: Self-compassion and social support among Jewish and Arab pregnant women in Israel. *Health Soc Care Community*. <https://doi.org/10.1111/hsc.13196>

Tchimtchoua Tamo, A. R. (2020). An analysis of mother stress before and during COVID-19 pandemic: The case of China. *Health Care for Women International*, *41*(11-12), 1349-1362. <https://doi.org/10.1080/07399332.2020.1841194>

TG, W. M., Kassie, B. A., Asratie, M. H., & Abate, A. T. (2021). The Effects of Fear and Knowledge of COVID-19 on Preventive Practice Among Pregnant Women Who Attend Antenatal Care in Northwest Ethiopia, 2020: Institution-Based Cross-Sectional Study. *Int J Womens Health*, *13*, 95-100. <https://doi.org/10.2147/ijwh.S286088>

Thayer, Z. M., & Gildner, T. E. (2020). COVID-19-related financial stress associated with higher likelihood of depression among pregnant women living in the United States. *American Journal of Human Biology*, e23508. <https://doi.org/10.1002/ajhb.23508>

Thompson, K. A., & Bardone-Cone, A. M. (2021). 2019-nCOV distress and depressive, anxiety and OCD-type, and eating disorder symptoms among postpartum and control women. *Arch Womens Ment Health*, 1-10. <https://doi.org/10.1007/s00737-021-01120-9>

Tso, W. W. Y., Wong, R. S., Tung, K. T. S., Rao, N., Fu, K. W., Yam, J. C. S., . . . Lp, P. (2020). Vulnerability and resilience in children during the COVID-19 pandemic. *European Child and Adolescent Psychiatry*, 1-16. <https://doi.org/10.1007/s00787-020-01680-8>

Ueda, R., Okada, T., Kita, Y., Ozawa, Y., Inoue, H., Shioda, M., . . . Ozawa, H. (2021). The quality of life of children with neurodevelopmental disorders and their parents during the Coronavirus disease 19 emergency in Japan. *Scientific Reports*, *11*(1), 3042. <https://doi.org/10.1038/s41598-021-82743-x>

Upendra, S., Devi, S., Kaur, J., Waghmare, S., & Barde, S. (2020). A phenomenological study of pregnant women’s experience: COVID-19 lockdown period. *Indian Journal of Forensic Medicine and Toxicology*, *14*(4), 3939-3942. <https://doi.org/10.37506/ijfmt.v14i4.12253>

Valero-Moreno, S., Lacomba-Trejo, L., Tamarit, A., Pérez-Marín, M., & Montoya-Castilla, I. (2021). Psycho-emotional adjustment in parents of adolescents: A cross-sectional and longitudinal analysis of the impact of the COVID pandemic. *Journal of Pediatric Nursing*. <https://doi.org/10.1016/j.pedn.2021.01.028>

Vasilevski, V., Sweet, L., Bradfield, Z., Wilson, A. N., Hauck, Y., Kuliukas, L., . . . Wynter, K. (2021). Receiving maternity care during the COVID-19 pandemic: Experiences of women's partners and support persons. *Women Birth*. <https://doi.org/10.1016/j.wombi.2021.04.012>

Vaterlaus, J. M., Shaffer, T., Patten, E. V., & Spruance, L. A. (2021). Parent-Child Relationships and the COVID-19 Pandemic: An Exploratory Qualitative Study with Parents in Early, Middle, and Late Adulthood. *J Adult Dev*, 1-13. <https://doi.org/10.1007/s10804-021-09381-5>

Vazquez-Vazquez, A., Dib, S., Rougeaux, E., Wells, J. C., & Fewtrell, M. S. (2021). The impact of the Covid-19 lockdown on the experiences and feeding practices of new mothers in the UK: Preliminary data from the COVID-19 New Mum Study. *Appetite*, *156*, 104985. <https://doi.org/10.1016/j.appet.2020.104985>

Vigod, S. N., Brown, H. K., Huang, A., Fung, K., Barker, L. C., Hussain-Shamsy, N., . . . Moineddin, R. (2021). Postpartum mental illness during the COVID-19 pandemic: a population-based, repeated cross-sectional study. *CMAJ: Canadian Medical Association Journal*, *193*(23), E835-e843. <https://doi.org/10.1503/cmaj.210151>

Waller, R., Powell, T., Rodriguez, Y., Corbett, N., Perlstein, S., White, L. K., . . . Wagner, N. J. (2021). The Impact of the COVID-19 Pandemic on Children's Conduct Problems and Callous-Unemotional Traits. *Child Psychiatry and Human Development*, 1-12. <https://doi.org/10.1007/s10578-020-01109-y>

Wang, J., Li, Y., Musch, D. C., Wei, N., Qi, X., Ding, G., . . . Qian, X. (2021). Progression of Myopia in School-Aged Children After COVID-19 Home Confinement. *JAMA Ophthalmol*, *139*(3), 293-300. <https://doi.org/10.1001/jamaophthalmol.2020.6239>

Wang, J., Zhou, Y., Qian, W., Han, R., & Liu, Z. (2021). Maternal insomnia during the COVID-19 pandemic: associations with depression and anxiety. *Social Psychiatry and Psychiatric Epidemiology*, 1-9. <https://doi.org/10.1007/s00127-021-02072-2>

Wang, Q., Mo, P. K. H., Song, B., Di, J. L., Zhou, F. R., Zhao, J., . . . Wang, L. H. (2021). Mental health and preventive behaviour of pregnant women in China during the early phase of the COVID-19 period. *Infect Dis Poverty*, *10*(1), 37. <https://doi.org/10.1186/s40249-021-00825-4>

Wang, Q., Song, B., Di, J., Yang, X., Wu, A., Lau, J., . . . Mo, P. K. (2021). Intentions to Seek Mental Health Services During the COVID-19 Pandemic Among Chinese Pregnant Women With Probable Depression or Anxiety: Cross-sectional, Web-Based Survey Study. *JMIR Ment Health*, *8*(2), e24162. <https://doi.org/10.2196/24162>

Wang, S. D., Devjani, S., Chillakanti, M., Dunton, G. F., & Mason, T. B. (2021). The COMET study: Examining the effects of COVID-19-related perceived stress on Los Angeles Mothers’ dysregulated eating behaviors, child feeding practices, and body mass index. *Appetite*, *163*, 105209.

Wang, Y., Chen, L., Wu, T., Shi, H., Li, Q., Jiang, H., . . . Qiao, J. (2020). Impact of Covid-19 in pregnancy on mother's psychological status and infant's neurobehavioral development: a longitudinal cohort study in China. *BMC Medicine*, *18*(1), 347. <https://doi.org/10.1186/s12916-020-01825-1>

Wdowiak, A., Makara-Studzińska, M., Raczkiewicz, D., Janczyk, P., Słabuszewska-Jóźwiak, A., Wdowiak-Filip, A., & Studzińska, N. (2021). Effect of Excessive Body Weight and Emotional Disorders on the Course of Pregnancy and Well-Being of a Newborn before and during COVID-19 Pandemic. *J Clin Med*, *10*(4). <https://doi.org/10.3390/jcm10040656>

Wheeler, J. M., Misra, D. P., & Giurgescu, C. (2021). Stress and coping among pregnant black women during the COVID-19 pandemic. *Public Health Nursing*. <https://doi.org/10.1111/phn.12909>

Wilder, J. L., Hark, C. M., Marcus, C. H., Rabinowitz, E. C., Michelson, C. D., Winn, A. S., & Pingree, E. W. (2021). Pediatric Trainees as Parents: Perspectives From a Pandemic. *Academic Pediatrics*. <https://doi.org/10.1016/j.acap.2021.04.006>

Wilke, N. G., Howard, A. H., & Goldman, P. (2020). Rapid return of children in residential care to family as a result of COVID-19: Scope, challenges, and recommendations. *Child Abuse and Neglect*, *110*. <https://doi.org/10.1016/j.chiabu.2020.104712>

Wimberly, C. E., Towry, L., Caudill, C., Johnston, E. E., & Walsh, K. M. (2021). Impacts of COVID-19 on caregivers of childhood cancer survivors. *Pediatric Blood & Cancer*, *68*(4), e28943. <https://doi.org/10.1002/pbc.28943>

Wolf, J. P., Freisthler, B., & Chadwick, C. (2021). Stress, alcohol use, and punitive parenting during the COVID-19 pandemic. *Child Abuse and Neglect*, *117*, 105090. <https://doi.org/10.1016/j.chiabu.2021.105090>

Wong, J. Y., Wai, A. K., Wang, M. P., Lee, J. J., Li, M., Kwok, J. Y., . . . Choi, A. W. (2021). Impact of COVID-19 on Child Maltreatment: Income Instability and Parenting Issues. *International Journal of Environmental Research and Public Health*, *18*(4). <https://doi.org/10.3390/ijerph18041501>

Wu, F., Lin, W., Liu, P., Zhang, M., Huang, S., Chen, C., . . . Chen, Q. (2021). Prevalence and contributory factors of anxiety and depression among pregnant women in the post-pandemic era of COVID-19 in Shenzhen, China. *Journal of Affective Disorders*, *291*, 243-251. <https://doi.org/10.1016/j.jad.2021.05.014>

Wu, Q., Xu, Y., & Jedwab, M. (2021). Custodial Grandparent's Job Loss During the COVID-19 Pandemic and Its Relationship With Parenting Stress and Mental Health. *Journal of Applied Gerontology*, 7334648211006222. <https://doi.org/10.1177/07334648211006222>

Wu, Y., Zhang, C., Liu, H., Duan, C., Li, C., Fan, J., . . . Huang, H. F. (2020). Perinatal depressive and anxiety symptoms of pregnant women during the coronavirus disease 2019 outbreak in China. *American Journal of Obstetrics and Gynecology*, *223*(2), 240.e241-240.e249. <https://doi.org/10.1016/j.ajog.2020.05.009>

Xie, M., Wang, X., Zhang, J., & Wang, Y. (2021). Alteration in the psychologic status and family environment of pregnant women before and during the COVID-19 pandemic. *International Journal of Gynaecology and Obstetrics*, *153*(1), 71-75. <https://doi.org/10.1002/ijgo.13575>

Xu, K., Zhang, Y., Xu, Q., Lv, L., & Zhang, J. (2021). Mental health among pregnant women under public health interventions during COVID-19 outbreak in Wuhan, China. *Psychiatry Research*, *301*, 113977. <https://doi.org/10.1016/j.psychres.2021.113977>

Xu, Y., Wu, Q., Levkoff, S. E., & Jedwab, M. (2020). Material hardship and parenting stress among grandparent kinship providers during the COVID-19 pandemic: The mediating role of grandparents' mental health. *Child Abuse and Neglect*, 104700. <https://doi.org/10.1016/j.chiabu.2020.104700>

Xue, A., Oros, V., Marca-Ghaemmaghami, P., Scholkmann, F., Righini-Grunder, F., Natalucci, G., . . . Restin, T. (2021). New Parents Experienced Lower Parenting Self-Efficacy during the COVID-19 Pandemic Lockdown. *Children (Basel)*, *8*(2). <https://doi.org/10.3390/children8020079>

Xue, B., & McMunn, A. (2021). Gender differences in unpaid care work and psychological distress in the UK Covid-19 lockdown. *PloS One*, *16*(3), e0247959. <https://doi.org/10.1371/journal.pone.0247959>

Yamamura, E., & Tsustsui, Y. (2021). The impact of closing schools on working from home during the COVID-19 pandemic: evidence using panel data from Japan. *Rev Econ Househ*, 1-20. <https://doi.org/10.1007/s11150-020-09536-5>

Yaman, A., Kandemir, I., & Varkal, M. A. (2021). Infants infected with SARS-CoV-2 and newborns born to mother diagnosed with COVID-19: clinical experience. *Irish Journal of Medical Science*, 1-6. <https://doi.org/10.1007/s11845-021-02662-8>

Yan, K., Xiao, F. F., Jiang, Y. W., Xiao, T. T., Zhang, D. J., Yuan, W. H., . . . Zeng, L. K. (2021). Effects of SARS-CoV-2 infection on neuroimaging and neurobehavior in neonates. *World Journal of Pediatrics*, *17*(2), 171-179. <https://doi.org/10.1007/s12519-021-00423-2>

Yang, H., Hu, B., Zhan, S., Yang, L. Y., & Xiong, G. (2020). Effects of Severe Acute Respiratory Syndrome Coronavirus 2 Infection on Pregnant Women and Their Infants. *Archives of Pathology and Laboratory Medicine*, *144*(10), 1217-1222. <https://doi.org/10.5858/arpa.2020-0232-SA>

Yang, R., Mei, H., Zheng, T., Fu, Q., Zhang, Y., Buka, S., . . . Zhou, A. (2020). Pregnant women with COVID-19 and risk of adverse birth outcomes and maternal-fetal vertical transmission: a population-based cohort study in Wuhan, China. *BMC Medicine*, *18*(1), 330. <https://doi.org/10.1186/s12916-020-01798-1>

Yang, X., Song, B., Wu, A., Mo, P. K. H., Di, J., Wang, Q., . . . Wang, L. (2021). Social, Cognitive, and eHealth Mechanisms of COVID-19-Related Lockdown and Mandatory Quarantine That Potentially Affect the Mental Health of Pregnant Women in China: Cross-Sectional Survey Study. *Journal of Medical Internet Research*, *23*(1), e24495. <https://doi.org/10.2196/24495>

Yassa, M., Yassa, A., Yirmibeş, C., Birol, P., Ünlü, U. G., Tekin, A. B., . . . Tug, N. (2020). Anxiety levels and obsessive compulsion symptoms of pregnant women during the COVID-19 pandemic. *Turk J Obstet Gynecol*, *17*(3), 155-160. <https://doi.org/10.4274/tjod.galenos.2020.91455>

Yavaş Çelik, M. (2021). The obligation of parents with COVID-19 positivity to stay separated from their children. *Journal of Child and Adolescent Psychiatric Nursing*, *34*(2), 105-111. <https://doi.org/10.1111/jcap.12303>

Yerkes, M. A., André, S. C. H., Besamusca, J. W., Kruyen, P. M., Remery, C., van der Zwan, R., . . . Geurts, S. A. E. (2020). 'Intelligent' lockdown, intelligent effects? Results from a survey on gender (in)equality in paid work, the division of childcare and household work, and quality of life among parents in the Netherlands during the Covid-19 lockdown. *PloS One*, *15*(11), e0242249. <https://doi.org/10.1371/journal.pone.0242249>

Yildirim, T. M., & Eslen-Ziya, H. (2020). The Differential Impact of COVID-19 on the Work Conditions of Women and Men Academics during the Lockdown. *Gend Work Organ*. <https://doi.org/10.1111/gwao.12529>

Yirmiya, K., Yakirevich-Amir, N., Preis, H., Lotan, A., Atzil, S., & Reuveni, I. (2021). Women's Depressive Symptoms during the COVID-19 Pandemic: The Role of Pregnancy. *International Journal of Environmental Research and Public Health*, *18*(8). <https://doi.org/10.3390/ijerph18084298>

Yu, N., Li, W., Kang, Q., Xiong, Z., Wang, S., Lin, X., . . . Wu, J. (2020). Clinical features and obstetric and neonatal outcomes of pregnant patients with COVID-19 in Wuhan, China: a retrospective, single-centre, descriptive study. *Lancet Infectious Diseases*, *20*(5), 559-564. <https://doi.org/10.1016/s1473-3099(20)30176-6>

Yuan, R., Xu, Q. H., Xia, C. C., Lou, C. Y., Xie, Z., Ge, Q. M., & Shao, Y. (2020). Psychological status of parents of hospitalized children during the COVID-19 epidemic in China. *Psychiatry Research*, *288*, 112953. <https://doi.org/10.1016/j.psychres.2020.112953>

Yue, C., Liu, C., Wang, J., Zhang, M., Wu, H., Li, C., & Yang, X. (2020). Association between social support and anxiety among pregnant women in the third trimester during the coronavirus disease 2019 (COVID-19) epidemic in Qingdao, China: The mediating effect of risk perception. *International Journal of Social Psychiatry*, 20764020941567. <https://doi.org/10.1177/0020764020941567>

Yue, J., Zang, X., Le, Y., & An, Y. (2020). Anxiety, depression and PTSD among children and their parent during 2019 novel coronavirus disease (COVID-19) outbreak in China. *Current Psychology (New Brunswick, N.J.)*, 1-8. <https://doi.org/10.1007/s12144-020-01191-4>

Zamarro, G., & Prados, M. J. (2021). Gender differences in couples' division of childcare, work and mental health during COVID-19. *Rev Econ Househ*, 1-30. <https://doi.org/10.1007/s11150-020-09534-7>

Zanardo, V., Manghina, V., Giliberti, L., Vettore, M., Severino, L., & Straface, G. (2020). Psychological impact of COVID-19 quarantine measures in northeastern Italy on mothers in the immediate postpartum period. *International Journal of Gynaecology and Obstetrics*, *150*(2), 184-188. <https://doi.org/10.1002/ijgo.13249>

Zanardo, V., Tortora, D., Guerrini, P., Garani, G., Severino, L., Soldera, G., & Straface, G. (2021). Infant feeding initiation practices in the context of COVID-19 lockdown. *Early Human Development*, *152*, 105286.

Zeng, L., Xia, S., Yuan, W., Yan, K., Xiao, F., Shao, J., & Zhou, W. (2020). Neonatal Early-Onset Infection With SARS-CoV-2 in 33 Neonates Born to Mothers With COVID-19 in Wuhan, China. *JAMA Pediatr*, *174*(7), 722-725. <https://doi.org/10.1001/jamapediatrics.2020.0878>

Zeng, X., Li, W., Sun, H., Luo, X., Garg, S., Liu, T., . . . Zhang, Y. (2020). Mental Health Outcomes in Perinatal Women During the Remission Phase of COVID-19 in China. *Front Psychiatry*, *11*, 571876. <https://doi.org/10.3389/fpsyt.2020.571876>

Zhang, C. J. P., Wu, H., He, Z., Chan, N. K., Huang, J., Wang, H., . . . Ming, W. K. (2021). Psychobehavioral Responses, Post-Traumatic Stress and Depression in Pregnancy During the Early Phase of COVID-19 Outbreak. *Psychiatric Research and Clinical Practice*, *3*(1), 46-54. <https://doi.org/10.1176/appi.prcp.20200019>

Zhang, Y., Deng, R., Chen, M., Cao, R., Chen, S., Chen, K., . . . Tian, K. (2021). Association of Sleep Duration and Screen Time With Anxiety of Pregnant Women During the COVID-19 Pandemic. *Frontiers in Psychology*, *12*, 646368. <https://doi.org/10.3389/fpsyg.2021.646368>

Zhang, Y., & Ma, Z. F. (2020). Psychological responses and lifestyle changes among pregnant women with respect to the early stages of COVID-19 pandemic. *International Journal of Social Psychiatry*, 20764020952116. <https://doi.org/10.1177/0020764020952116>

Zheng, Q. X., Jiang, X. M., Lin, Y., Liu, G. H., Lin, Y. P., Kang, Y. L., & Liu, X. W. (2020). The influence of psychological response and security sense on pregnancy stress during the outbreak of coronavirus disease 2019: A mediating model. *Journal of Clinical Nursing*, *29*(21-22), 4248-4257. <https://doi.org/10.1111/jocn.15460>

Zhou, Y., Shi, H., Liu, Z., Peng, S., Wang, R., Qi, L., . . . Zhang, X. (2020). The prevalence of psychiatric symptoms of pregnant and non-pregnant women during the COVID-19 epidemic. *Transl Psychiatry*, *10*(1), 319. <https://doi.org/10.1038/s41398-020-01006-x>

Zhou, Y., Wang, R., Liu, L., Ding, T., Huo, L., Qi, L., . . . Dai, G. (2021). The impact of lockdown policy on depressive symptoms among pregnant women in China: mediating effects of internet use and family support. *Glob Health Res Policy*, *6*(1), 11. <https://doi.org/10.1186/s41256-021-00193-4>

Zilver, S. J. M., Broekman, B. F. P., Hendrix, Y., de Leeuw, R. A., Mentzel, S. V., van Pampus, M. G., & de Groot, C. J. M. (2021). Stress, anxiety and depression in 1466 pregnant women during and before the COVID-19 pandemic: a Dutch cohort study. *Journal of Psychosomatic Obstetrics and Gynaecology*, *42*(2), 108-114. <https://doi.org/10.1080/0167482x.2021.1907338>

Zreik, G., Asraf, K., Haimov, I., & Tikotzky, L. (2020). Maternal perceptions of sleep problems among children and mothers during the coronavirus disease 2019 (COVID-19) pandemic in Israel. *Journal of Sleep Research*, e13201. <https://doi.org/10.1111/jsr.13201>

Çakmak, G., & Öztürk, Z. A. (2021). Being Both a Parent and a Healthcare Worker in the Pandemic: Who Could Be Exhausted More? *Healthcare (Basel)*, *9*(5). <https://doi.org/10.3390/healthcare9050564>

Çolak, S., Gürlek, B., Önal, Ö., Yılmaz, B., & Hocaoglu, C. (2021). The level of depression, anxiety, and sleep quality in pregnancy during coronavirus disease 2019 pandemic. *Journal of Obstetrics and Gynaecology Research*. <https://doi.org/10.1111/jog.14872>

Özkan Şat, S., & Yaman Sözbir, Ş. (2021). Use of Mobile Applications by Pregnant Women and Levels of Pregnancy Distress During the COVID-19 (Coronavirus) Pandemic. *Matern Child Health J*, *25*(7), 1057-1068. <https://doi.org/10.1007/s10995-021-03162-y>
