# Supplementary material for: Impact of the COVID-19 pandemic on maternal mental health, early childhood development, and parental practices: a global scoping review
Source: BMC Public Health. 2023 Feb 24;23:388. doi: 10.1186/s12889-023-15003-4 (PMC9950022; doi:10.1186/s12889-023-15003-4)
Supplement: Supplementary file 1 — Additional file 1. [file 12889_2023_15003_MOESM1_ESM.zip › 12889_2023_15003_MOESM3_ESM.docx]

**Case Report Studies: Invidivual Characteristics**

| **Study Reference** | **Country** | **Subjects (n)** | **Primary Outcomes** |
| --- | --- | --- | --- |
| (Zhu et al., 2020) | China | 10 | Neonates born to covid-positive mothers' outcomes: full blood count, chest x-ray, pneumotórax. |
| (Hsu et al., 2020) | U.S.A | 1 | Neonate born to covid-positive mother' outcomes: resuscitation and Apgar at 1 and 5 minutes, weight, lenght, head circunference, age at birth. |
| (Sola et al., 2020) | America Latina | 80 | Neonates born to covid-positive mother' outcomes: breathing difficulties, pulmonary malformation (CCAM), preterm, death |
| (Salvatori et al., 2020) | Italy | 2 | Neonates born to covid-positive mothers' outcomes: age and weight at birth, gender, fever, saturation and oxygen support, NICU admission, COVID test. |
| (Soumana et al., 2020) | Niger | 1 | Child outcome: death after severe acute malnutrition (SAM) combined with COVID |
| (P. Liu et al., 2020) | China | 51 | Neonates born to covid-positive mothers' outcomes: full blood count, age and weight at birth, Apgar at 1 and 5m, chest image, COVID swab test. |
| (Fan et al., 2020) | China | 12 | Neonates born to covid-positive mothers' outcomes: age and weight at birht, sex, COVID swab test, Apgar at 1 and 5m, asphyxia, death. |
| (Xu et al., 2020) | U.S.A | 362 | Correlation of parental stress, family material hardship moderated by caregivers as grandparents and their mental health (MHI-5) |
| (Alay et al., 2020) | Turkey | 27 | Neonates born to covid-positive mothers' outcomes: fetal distress, age and weight at birth, NICU admission, Apgar 1 and 5m, COVID swab test. |
| (Mehta et al., 2020) | U.S.A | 2 | Neonates born to covid-positive mothers' outcomes: preterm, sex, weight, Apgar at 1 and 5m, NICU admission, COVID swab test, alveolar hemorrhage, mechanical ventilation. |
| (Kamali Aghdam et al., 2020) | Iran | 1 | COVID-positve neonatal outcomes: weight at birrth, NICU admission, respiratory distress, patent foramen ovale (PFO). |
| (Silverstein et al., 2020) | U.S.A | 2 | Neonates born to covid-positive mothers' outcomes: preterm, sex, weight, Apgar at 1 and 5m, COVID test, |
| (Ferrazzi et al., 2020) | Italy | 24 | Neonates born to covid-positive mothers' outcomes: weight, Apgar at 1 and 5m, COVID test, breastfeeding, vaginal delivery. |
| (Yang et al., 2020) | China | 7 | Neonates born to covid-positive mothers' outcomes: age and weight at bith, Apgar at 1 and 5m, NICU admission, respiratory support, COVID test(throat sawb, umbilical cord ans amniotic fluid. |
| (W. Liu, J. Wang, et al., 2020) | China | 19 | Neonates born to covid-positive mothers' outcomes: full blood count, chest x ray, immediate separation from mothers for 14 days, COVID test |
| (Lizama et al., 2021) | Peru | 206 | Gestational age, birth weigh, sex, COVID PCR test result, prematurity, low birth weight, breastfeeding type during hospital stay and after discharge, duration of hospital stay. Neonatal COVID symptoms were also analysed |
| (Biasucci et al., 2020) | Italy | 15 | Sex, Birth weight, 5’Apgar score, Swab at birth D3 D7 D14, Rooming-in and breast feeding, Symptoms in hospital, Symptoms after discharge |
| (Kamity et al., 2021) | U.S.A | 2 | Neonates born to covid-positive mothers' outcomes: collapsed lungs |
| (Franchi et al., 2020) | Italy | 1 | COVID positive mother- Postpartum psychosis, dyad separation |
| (Mendoza-Hernández et al., 2021) | Mexico | 1 | BW, Apgar 5’ and 7’, SIlverman, NICU admission, RT-PCR test |
| (Sánchez-Luna et al., 2021) | Spain | 503 | Birth weight, Prematurity, Apgar 1' and 5', Immediate skin-to-skin contact, Type of feeding, PCR diagnostic test, Admission to the NICU |
| (Paramanathan et al., 2021) | Denmark | 1 | Birth weight, Apgar 1' 5' 10', COVID test, prematurity |
| (Z. He et al., 2021) | China | 22 | Birth weight, Apgar 1' 5', Fetal death, covid test, |
| (Jak et al., 2021) | Brazil | 1 | Hypotonic, birthweight, Apgar scores, NICU admittance and perinatal death |
| (Mejía Jiménez et al., 2021) | Spain | 403 | COVID test, Type of delivery, Skin-to-skin, Breastfeeding, Apgar 1'and 5', NICU admission, |
| (Antoun et al., 2020) | England | 20 | Neonatal outcomes: Apgar (1 and 5 minutes), resuscitation, COVID swabs |
| (Abasse et al., 2020) | France | 1 | Clinical outcomes: covid pneumonia, preterm, 6 week NICU stay |
| (Schwartz, 2020) | U.S. | 39 | Neonates born to covid-positive mothers' outcomes: age and weight at birth, Apgar at 1 and 5m, small for gestational age, COVID swab test |
| (Khoury et al., 2020) | U.S. | 247 | Neonatal outcomes: gestational age and weight at birth, sex, COVID test, NICU admission, respiratory distress, sepsis, prematurity, anomaly |
| (Salik & Mehta, 2020) | U.S. | 1 | Neonate born to COVID-positive mother: occurrence of congenital heart defect (tetralogy of fallot), preterm, age and weight at birth, reduced lung volume, apneic episodes, covid positive, sustained hypoxia, shunt placement, NICU admission. |
| (Congdon et al., 2021) | U.S. | 70 | Newborn SARS-COV-2 status |
| (Kolkova et al., 2020) | Sweden | 1 | Neonatal outcomes |
| (J. R. He et al., 2021) | China | 1 | Neonatal outcomes and mother with covid |
| (Mulale et al., 2021) | Botswana | 1 | TB and COVID coinfection in low income country |
| (W. Liu, H. Cheng, et al., 2020) | China | 48 | Neonatal outcomes |
| (Schoenmakers et al., 2021) | Netherlands | 1 | Neonatal outcomes |
| Moeindarbary (Moeindarbary et al., 2021) | Iran | 2 | Neonatal outcomes |
| (Shaiba et al., 2021) | Saudi Arabia | 2 | Neonatal outcomes |
| (McCarty et al., 2021) | U.S. | 1 | Neonatal outcomes |
| (Ghema et al., 2021) | Morrocco | 32 | Neonatal outcomes |
| (Chen et al., 2020) | China | 3 | Neonatal outcomes |
| (Schwartz et al., 2021) | International (not specified) | 6 | Neonatal outcomes |
| Loret de Mola(Loret de Mola et al., 2021) | China | 1 | Neonatal outcomes |
| (Sabharwal et al., 2021) | U.S. | 75 | Neonatal outcomes, newborn care practices, delivery outcomes |
| (Harahap et al., 2021) | Indonesia | Neonates | Weight, Apgar, NICU admission, prematurity |
| (Kuhrt et al., 2020) | U.K. | Neonates | Mother covid-19 with placental abruption and neonatal outcomes |
| (Cooke et al., 2020) | U.K. | Neonates | Apgar scores, birthweight, intubation |
|  |  |  |  |
| (Rivera-Hernandez et al., 2020) | U.S. | Neonates | Laboratory results |
| (Perlman et al., 2020) | U.S. | Neonates | Apgar, prematurity, CPAP use, NICU admission |
| (Piersigilli et al., 2020) | Belgium | Neonates | Laboratory results |
| (Birindwa et al., 2021) | Congo | Pregnant women and neonates | Vascular outcomes in placenta and umbilical cord vessels |
| (du Fossé et al., 2021) | Netherlands | Pregnant women | Primigravid with Covid-19 related respiratory insufficiency and decreased naive T-cell and B-cell compartments |
| (Farhadi et al., 2021) | Iran | Mothers and newborns | Experience relating to three mother-infant pairs who were offered virtual bonding during the COVID-19 pandemic |
| (Fontanella et al., 2020) | Netherlands | Pregnant women | COVID-19 symptoms in woman with gestational diabetes |
| (Nakstad et al., 2021) | Botswana | Preterm neonates | Weight, laboratory results |

**References**

Abasse, S., Essabar, L., Costin, T., Mahisatra, V., Kaci, M., Braconnier, A., . . . Fayssoil, A. (2020). Neonatal COVID-19 Pneumonia: Report of the First Case in a Preterm Neonate in Mayotte, an Overseas Department of France. *Children (Basel)*, *7*(8). <https://doi.org/10.3390/children7080087>

Alay, I., Yildiz, S., Kaya, C., Yasar, K. K., Aydin, O. A., Karaosmanoglu, H. K., . . . Ekin, M. (2020). The clinical findings and outcomes of symptomatic pregnant women diagnosed with or suspected of having coronavirus disease 2019 in a tertiary pandemic hospital in Istanbul, Turkey. *Journal of Obstetrics and Gynaecology Research*. <https://doi.org/10.1111/jog.14493>

Antoun, L., Taweel, N. E., Ahmed, I., Patni, S., & Honest, H. (2020). Maternal COVID-19 infection, clinical characteristics, pregnancy, and neonatal outcome: A prospective cohort study. *European Journal of Obstetrics, Gynecology, and Reproductive Biology*, *252*, 559-562. <https://doi.org/10.1016/j.ejogrb.2020.07.008>

Biasucci, G., Cannalire, G., Raymond, A., Capra, M. E., Benenati, B., Vadacca, G., . . . Bonini, R. (2020). Safe Perinatal Management of Neonates Born to SARS-CoV-2 Positive Mothers at the Epicenter of the Italian Epidemic. *Frontiers in Pediatrics*, *8*. <https://doi.org/10.3389/fped.2020.565522>

Birindwa, E. K., Mulumeoderhwa, G. M., Nyakio, O., Mbale, G. Q. M., Mushamuka, S. Z., Materanya, J. M., . . . Balaluka, G. B. (2021). A case study of the first pregnant woman with COVID-19 in Bukavu, eastern Democratic Republic of the Congo. *Maternal Health, Neonatology and Perinatology*, *7*(1). <https://doi.org/10.1186/s40748-021-00127-5>

Chen, H., Guo, J., Wang, C., Luo, F., Yu, X., Zhang, W., . . . Zhang, Y. (2020). Clinical characteristics and intrauterine vertical transmission potential of COVID-19 infection in nine pregnant women: a retrospective review of medical records. *Lancet*, *395*(10226), 809-815. <https://doi.org/10.1016/s0140-6736(20)30360-3>

Congdon, J. L., Kair, L. R., Flaherman, V. J., Wood, K. E., LoFrumento, M. A., Nwaobasi-Iwuh, E., & Phillipi, C. A. (2021). Management and Early Outcomes of Neonates Born to Women with SARS-CoV-2 in 16 U.S. Hospitals. *American Journal of Perinatology*, *38*(6), 622-631. <https://doi.org/10.1055/s-0041-1726036>

Cooke, W. R., Billett, A., Gleeson, S., Jacques, A., Place, K., Siddall, J., . . . Soulsby, K. (2020). SARS-CoV-2 infection in very preterm pregnancy: Experiences from two cases. *European Journal of Obstetrics and Gynecology and Reproductive Biology*, *250*, 259-260. <https://doi.org/10.1016/j.ejogrb.2020.05.025>

du Fossé, N. A., Bronsgeest, K., Arbous, M. S., Zlei, M., Myeni, S. K., Kikkert, M., . . . van den Akker, T. (2021). Detailed immune monitoring of a pregnant woman with critical Covid-19. *Journal of Reproductive Immunology*, *143*. <https://doi.org/10.1016/j.jri.2020.103243>

Fan, C., Guo, Y., Qu, P., Wang, S., Wang, M., Yuan, J., . . . Wang, B. (2020). No Obviously Adverse Pregnancy Complications and Outcomes of the Recovered Pregnant Women from COVID-19. *Reproductive Toxicology*. <https://doi.org/10.1016/j.reprotox.2020.11.008>

Farhadi, R., Mehrpisheh, S., & Philip, R. K. (2021). Mobile-Assisted Virtual Bonding Enables Breast Milk Supply in Critically Ill Mothers With COVID-19: A Reflection on the Feasibility of Telelactation. *Cureus*, *13*(3), e13699. <https://doi.org/10.7759/cureus.13699>

Ferrazzi, E., Frigerio, L., Savasi, V., Vergani, P., Prefumo, F., Barresi, S., . . . Villa, A. (2020). Vaginal delivery in SARS-CoV-2-infected pregnant women in Northern Italy: a retrospective analysis. *BJOG: An International Journal of Obstetrics and Gynaecology*, *127*(9), 1116-1121. <https://doi.org/10.1111/1471-0528.16278>

Fontanella, F., Hannes, S., Keating, N., Martyn, F., Browne, I., Briet, J., . . . Baalman, J. H. (2020). COVID-19 infection during the third trimester of pregnancy: Current clinical dilemmas. *European Journal of Obstetrics and Gynecology and Reproductive Biology*, *251*, 268-271. <https://doi.org/10.1016/j.ejogrb.2020.05.053>

Franchi, M., Del Piccolo, L., Bosco, M., Tosadori, C., Casarin, J., Laganà, A. S., & Garzon, S. (2020). COVID-19 and mental health in the obstetric population: a lesson from a case of puerperal psychosis. *Minerva Ginecologica*, *72*(5), 355-357. <https://doi.org/10.23736/s0026-4784.20.04606-7>

Ghema, K., Lehlimi, M., Toumi, H., Badre, A., Chemsi, M., Habzi, A., & Benomar, S. (2021). Outcomes of newborns to mothers with COVID-19. *Infect Dis Now*. <https://doi.org/10.1016/j.idnow.2021.03.003>

Harahap, A., Harianto, A., Etika, R., Utomo, M. T., Angelika, D., Handayani, K. D., & Arif Sampurna, M. T. (2021). Spontaneous Ileum Perforation in a premature twin with Coronavirus-19 positive mother. *Journal of Pediatric Surgery Case Reports*, *67*. <https://doi.org/10.1016/j.epsc.2021.101807>

He, J. R., Xiao, Y. H., Ding, W., Shi, Y. L., He, X., Liu, X. D., . . . Qiu, X. (2021). Maternal, placental and neonatal outcomes after asymptomatic SARS-CoV-2 infection in the first trimester of pregnancy: A case report. *Case Reports in Women's Health*, *31*. <https://doi.org/10.1016/j.crwh.2021.e00321>

He, Z., Fang, Y., Zuo, Q., Huang, X., Lei, Y., Ren, X., & Liu, D. (2021). Vertical transmission and kidney damage in newborns whose mothers had coronavirus disease 2019 during pregnancy. *International Journal of Antimicrobial Agents*, *57*(2), 106260. <https://doi.org/10.1016/j.ijantimicag.2020.106260>

Hsu, A. L., Guan, M., Johannesen, E., Stephens, A. J., Khaleel, N., Kagan, N., . . . Wan, X. F. (2020). Placental SARS-CoV-2 in a pregnant woman with mild COVID-19 disease. *Journal of Medical Virology*. <https://doi.org/10.1002/jmv.26386>

Jak, B., Zanirati, G., Rodrigues, F. V. F., Grahl, M., Krimberg, F., Pinzetta, G., . . . Marinowic, D. R. (2021). Case Report: Placental Maternal Vascular Malperfusion Affecting Late Fetal Development and Multiorgan Infection Caused by SARS-CoV-2 in Patient With PAI-1 4G/5G Polymorphism. *Front Med (Lausanne)*, *8*, 624166. <https://doi.org/10.3389/fmed.2021.624166>

Kamali Aghdam, M., Jafari, N., & Eftekhari, K. (2020). Novel coronavirus in a 15-day-old neonate with clinical signs of sepsis, a case report. *Infect Dis (Lond)*, *52*(6), 427-429. <https://doi.org/10.1080/23744235.2020.1747634>

Kamity, R., Nayak, A., & Dumpa, V. (2021). Pneumothorax in Neonates Born to COVID-19-Positive Mothers: Fact or Fortuity? *AJP Rep*, *11*(1), e49-e53. <https://doi.org/10.1055/s-0041-1726020>

Khoury, R., Bernstein, P. S., Debolt, C., Stone, J., Sutton, D. M., Simpson, L. L., . . . Avila, K. (2020). Characteristics and Outcomes of 241 Births to Women With Severe Acute Respiratory Syndrome Coronavirus 2 (SARS-CoV-2) Infection at Five New York City Medical Centers. *Obstetrics and Gynecology*, *136*(2), 273-282. <https://doi.org/10.1097/AOG.0000000000004025>

Kolkova, Z., Bjurström, M. F., Länsberg, J. K., Svedas, E., Hamer, M. A., Hansson, S. R., . . . Zaigham, M. (2020). Obstetric and intensive-care strategies in a high-risk pregnancy with critical respiratory failure due to COVID-19: A case report. *Case Reports in Women's Health*, *27*. <https://doi.org/10.1016/j.crwh.2020.e00240>

Kuhrt, K., McMicking, J., Nanda, S., Nelson-Piercy, C., & Shennan, A. (2020). Placental abruption in a twin pregnancy at 32 weeks’ gestation complicated by coronavirus disease 2019 without vertical transmission to the babies. *American Journal of Obstetrics and Gynecology MFM*, *2*(3). <https://doi.org/10.1016/j.ajogmf.2020.100135>

Liu, P., Zheng, J., Yang, P., Wang, X., Wei, C., Zhang, S., . . . Zhang, Y. (2020). The immunologic status of newborns born to SARS-CoV-2-infected mothers in Wuhan, China. *Journal of Allergy and Clinical Immunology*, *146*(1), 101-109.e101. <https://doi.org/10.1016/j.jaci.2020.04.038>

Liu, W., Cheng, H., Wang, J., Ding, L., Zhou, Z., Liu, S., . . . Rong, Z. (2020). Clinical Analysis of Neonates Born to Mothers with or without COVID-19: A Retrospective Analysis of 48 Cases from Two Neonatal Intensive Care Units in Hubei Province. *American Journal of Perinatology*, *37*(13), 1317-1323. <https://doi.org/10.1055/s-0040-1716505>

Liu, W., Wang, J., Li, W., Zhou, Z., Liu, S., & Rong, Z. (2020). Clinical characteristics of 19 neonates born to mothers with COVID-19. *Frontiers of Medicine*, *14*(2), 193-198. <https://doi.org/10.1007/s11684-020-0772-y>

Lizama, O., Mucha, J., del Carmen Chincaro, M., Giraldo, G., Salazar, J., Agüero, K., . . . Espinoza, D. (2021). Pre and post-natal epidemiological and clinical features of neonates born from mothers infected with COVID-19 and 14-day follow-up post discharge in Lima, Peru. *Revista Medica Herediana*, *32*(1), 5-11. <https://doi.org/10.20453/RMH.V32I1.3942>

Loret de Mola, C., Blumenberg, C., Martins, R. C., Martins-Silva, T., Carpena, M. X., Del-Ponte, B., . . . Cesar, J. A. (2021). Increased depression and anxiety during the COVID-19 pandemic in Brazilian mothers: a longitudinal study. *Braz J Psychiatry*, *43*(3), 337-338. <https://doi.org/10.1590/1516-4446-2020-1628>

McCarty, K. L., Tucker, M., Lee, G., & Pandey, V. (2021). Fetal inflammatory response syndrome associated with maternal SARS-CoV-2 infection. *Pediatrics*, *147*(4). <https://doi.org/10.1542/peds.2020-010132>

Mehta, H., Ivanovic, S., Cronin, A., VanBrunt, L., Mistry, N., Miller, R., . . . Rezai, F. (2020). Novel coronavirus-related acute respiratory distress syndrome in a patient with twin pregnancy: A case report. *Case Rep Womens Health*, *27*, e00220. <https://doi.org/10.1016/j.crwh.2020.e00220>

Mejía Jiménez, I., Salvador López, R., García Rosas, E., Rodriguez de la Torre, I., Montes García, J., de la Cruz Conty, M. L., & Martínez Pérez, O. (2021). Umbilical cord clamping and skin-to-skin contact in deliveries from women positive for SARS-CoV-2: a prospective observational study. *BJOG: An International Journal of Obstetrics and Gynaecology*, *128*(5), 908-915. <https://doi.org/10.1111/1471-0528.16597>

Mendoza-Hernández, M., Huerta-Niño de Rivera, I., Yoldi-Negrete, M., Saviñon-Tejeda, P., Franco-Cendejas, R., López-Jácome, L. E., & Navarro-Castellanos, I. (2021). Probable Case of Vertical Transmission of SARS-CoV-2 in a Newborn in Mexico. *Neonatology*, 1-4. <https://doi.org/10.1159/000514710>

Moeindarbary, S., Pourhoseini, A., Layegh, P., Shahriari, Z., Fayyaz, F., Bahrami, M., & Rafiee, M. (2021). Neonates with coronavirus disease 2019 acquired from infected mothers: the incompatibility of maternal intensity and infant lung involvement: two case reports. *J Med Case Rep*, *15*(1), 310. <https://doi.org/10.1186/s13256-021-02698-5>

Mulale, U. K., Kashamba, T., Strysko, J., & Kyokunda, L. T. (2021). Fatal SARS-CoV-2 and Mycobacterium tuberculosis coinfection in an infant: insights from Botswana. *BMJ Case Reports*, *14*(4). <https://doi.org/10.1136/bcr-2020-239701>

Nakstad, B., Kaang, T., Gezmu, A. M., & Strysko, J. (2021). Nosocomial SARS-CoV-2 transmission in a neonatal unit in Botswana: chronic overcrowding meets a novel pathogen. *BMJ Case Reports*, *14*(6). <https://doi.org/10.1136/bcr-2021-242421>

Paramanathan, S., Kyng, K. J., Laursen, A. L., Jensen, L. D., Grejs, A. M., & Jain, D. (2021). COVID-19 with severe acute respiratory distress in a pregnant woman leading to preterm caesarean section: A case report. *Case Rep Womens Health*, *30*, e00304. <https://doi.org/10.1016/j.crwh.2021.e00304>

Perlman, J., Oxford, C., Chang, C., Salvatore, C., & Pace, J. D. (2020). Delivery Room Preparedness and Early Neonatal Outcomes during COVID-19 Pandemic in New York City. *Pediatrics*, *146*(2). <https://doi.org/10.1542/peds.2020-1567>

Piersigilli, F., Carkeek, K., Hocq, C., van Grambezen, B., Hubinont, C., Chatzis, O., . . . Danhaive, O. (2020). COVID-19 in a 26-week preterm neonate. *The Lancet Child and Adolescent Health*, *4*(6), 476-478. <https://doi.org/10.1016/S2352-4642(20)30140-1>

Rivera-Hernandez, P., Nair, J., Islam, S., Davidson, L., Chang, A., & Elberson, V. (2020). Coronavirus Disease 2019 in a Premature Infant: Vertical Transmission and Antibody Response or Lack Thereof. *AJP Reports*, *10*(3), E224-E227. <https://doi.org/10.1055/s-0040-1715176>

Sabharwal, V., Bartolome, R., Hassan, S. A., Levesque, B. M., Camelo, I. Y., Wachman, E. M., . . . Parker, M. G. (2021). Mother-Infant Dyads with COVID-19 at an Urban, Safety-Net Hospital: Clinical Manifestations and Birth Outcomes. *American Journal of Perinatology*, *38*(7), 741-746. <https://doi.org/10.1055/s-0041-1726429>

Salik, I., & Mehta, B. (2020). Tetralogy of Fallot palliation in a COVID-19 positive neonate. *Journal of Clinical Anesthesia*, *66*, 109914. <https://doi.org/10.1016/j.jclinane.2020.109914>

Salvatori, G., De Rose, D. U., Concato, C., Alario, D., Olivini, N., Dotta, A., & Campana, A. (2020). Managing COVID-19-Positive Maternal-Infant Dyads: An Italian Experience. *Breastfeeding Medicine*, *15*(5), 347-348. <https://doi.org/10.1089/bfm.2020.0095>

Schoenmakers, S., Snijder, P., Verdijk, R. M., Kuiken, T., Kamphuis, S. S. M., Koopman, L. P., . . . Reiss, I. K. M. (2021). Severe Acute Respiratory Syndrome Coronavirus 2 Placental Infection and Inflammation Leading to Fetal Distress and Neonatal Multi-Organ Failure in an Asymptomatic Woman. *J Pediatric Infect Dis Soc*, *10*(5), 556-561. <https://doi.org/10.1093/jpids/piaa153>

Schwartz, D. A. (2020). An Analysis of 38 Pregnant Women with COVID-19, Their Newborn Infants, and Maternal-Fetal Transmission of SARS-CoV-2: Maternal Coronavirus Infections and Pregnancy Outcomes. *Archives of Pathology and Laboratory Medicine*. <https://doi.org/10.5858/arpa.2020-0901-SA>

Schwartz, D. A., Baldewijns, M., Benachi, A., Bugatti, M., Collins, R. R. J., De Luca, D., . . . Vivanti, A. J. (2021). Chronic Histiocytic Intervillositis With Trophoblast Necrosis Is a Risk Factor Associated With Placental Infection From Coronavirus Disease 2019 (COVID-19) and Intrauterine Maternal-Fetal Severe Acute Respiratory Syndrome Coronavirus 2 (SARS-CoV-2) Transmission in Live-Born and Stillborn Infants. *Archives of Pathology and Laboratory Medicine*, *145*(5), 517-528. <https://doi.org/10.5858/arpa.2020-0771-SA>

Shaiba, L. A., Hadid, A., Altirkawi, K. A., Bakheet, H. M., Alherz, A. M., Hussain, S. A., . . . Alzamil, F. A. (2021). Case Report: Neonatal Multi-System Inflammatory Syndrome Associated With SARS-CoV-2 Exposure in Two Cases From Saudi Arabia. *Front Pediatr*, *9*, 652857. <https://doi.org/10.3389/fped.2021.652857>

Silverstein, J. S., Limaye, M. A., Brubaker, S. G., Roman, A. S., Bautista, J., Chervenak, J., . . . Penfield, C. A. (2020). Acute Respiratory Decompensation Requiring Intubation in Pregnant Women with SARS-CoV-2 (COVID-19). *AJP Rep*, *10*(2), e169-e175. <https://doi.org/10.1055/s-0040-1712925>

Sola, A., Rodríguez, S., Cardetti, M., & Dávila, C. (2020). [Perinatal COVID-19 in Latin America]. *Revista Panamericana de Salud Publica*, *44*, e47. <https://doi.org/10.26633/rpsp.2020.47>

Soumana, A., Samaila, A., Moustapha, L. M., Kamaye, M., Daouda, B., Salifou, I. A., . . . Ibrahim, M. L. (2020). A Fatal Case of COVID-19 in an Infant with Severe Acute Malnutrition Admitted to a Paediatric Ward in Niger. *Case Rep Pediatr*, *2020*, 8847415. <https://doi.org/10.1155/2020/8847415>

Sánchez-Luna, M., Fernández Colomer, B., de Alba Romero, C., Alarcón Allen, A., Baña Souto, A., Camba Longueira, F., . . . Zamora Flores, E. (2021). Neonates Born to Mothers With COVID-19: Data From the Spanish Society of Neonatology Registry. *Pediatrics*, *147*(2). <https://doi.org/10.1542/peds.2020-015065>

Xu, Y., Wu, Q., Levkoff, S. E., & Jedwab, M. (2020). Material hardship and parenting stress among grandparent kinship providers during the COVID-19 pandemic: The mediating role of grandparents' mental health. *Child Abuse and Neglect*, 104700. <https://doi.org/10.1016/j.chiabu.2020.104700>

Yang, P., Wang, X., Liu, P., Wei, C., He, B., Zheng, J., & Zhao, D. (2020). Clinical characteristics and risk assessment of newborns born to mothers with COVID-19. *Journal of Clinical Virology*, *127*, 104356. <https://doi.org/10.1016/j.jcv.2020.104356>

Zhu, H., Wang, L., Fang, C., Peng, S., Zhang, L., Chang, G., . . . Zhou, W. (2020). Clinical analysis of 10 neonates born to mothers with 2019-nCoV pneumonia. *Transl Pediatr*, *9*(1), 51-60. <https://doi.org/10.21037/tp.2020.02.06>
