# Supplementary material for: Impact of the COVID-19 pandemic on maternal mental health, early childhood development, and parental practices: a global scoping review
Source: BMC Public Health. 2023 Feb 24;23:388. doi: 10.1186/s12889-023-15003-4 (PMC9950022; doi:10.1186/s12889-023-15003-4)
Supplement: Supplementary file 1 — Additional file 1. [file 12889_2023_15003_MOESM1_ESM.zip › 12889_2023_15003_MOESM4_ESM.docx]

**Systematic Reviews Classification**

| **Study Reference** | **PRISMA grade** | **Primary Outcomes** |
| --- | --- | --- |
| (Hessami, Romanelli, et al., 2020) | 29 | Maternal mental health |
| (Juan et al., 2020) | 24 | COVID impact on neonatal and maternal health |
| (Muhidin et al., 2020) | 26 | Neonatal and maternal health |
| (Capobianco et al., 2020) | 27 | Neonatal and maternal health |
| (Huntley et al., 2020) | 22 | Neonatal and maternal health |
| (Hessami, Homayoon, et al., 2020) | 15 | Neonatal and maternal health |
| (Teles Abrao Trad et al., 2020) | 17 | Neonatal and maternal health |
| (Smith et al., 2020) | 24 | Neonatal and maternal health |
| (Zaigham & Andersson, 2020) | 18 | Neonatal and maternal health |
| (Chi et al., 2020) | 16 | Neonatal and maternal health |
| (Ashraf et al., 2020) | 21 | Neonatal and maternal health |
| (Yoon et al., 2020) | 18 | Neonatal health |
| (Abou Ghayda et al., 2020) | 19 | Neonatal and maternal health |
| (Guo et al., 2020) | 19 | Child health |
| (Di Mascio et al., 2020) | 28 | Neonatal and maternal health |
| (Akhtar et al., 2020) | 16 | Neonatal and maternal health |
| (Araújo et al., 2020) | 18 | COVID impact on child development and parental mental health |
| (Walker et al., 2020) | 24 | COVID impact on neonatal health through breastfeeding |
| (V & Iarocci, 2020) | 29 | Child mental health |
| (Amaral et al., 2020) | 22 | Neonatal and maternal health |
| (Diriba et al., 2020) | 31 | Neonatal and maternal health |
| (Papapanou et al., 2021) | 36 | Neonatal and maternal health, child development |
| (Fan et al., 2021) | 31 | Maternal mental health |
| (Jones et al., 2021) | 34 | Child development |
| (Mark et al., 2021) | 25 | Child development |
| (Shorey et al., 2021) | 34 | Maternal mental health |
| (Yan et al., 2020) | 38 | Maternal mental health |
| (Chmielewska et al., 2021) | 35 | Maternal and perinatal mortality and morbidity, pregnancy complications and intrapartum/neonatal outcomes |
| Islam (Islam et al., 2020) | 19 | Neonatal outcomes |
| (Jafari et al., 2021) | 25 | Maternal and neonate outcomes in COVID-19 positive women |
| (Stavridou et al., 2020) | 23 | Child mental health and development |
| (Sun et al., 2020) | 25 | Maternal mental health |
| (Tomfohr-Madsen et al., 2021) | 21 | Antenatal depression and anxiety |

**References**

Abou Ghayda, R., Li, H., Lee, K. H., Lee, H. W., Hong, S. H., Kwak, M., . . . Shin, J. I. (2020). COVID-19 and Adverse Pregnancy Outcome: A Systematic Review of 104 Cases. *J Clin Med*, *9*(11). <https://doi.org/10.3390/jcm9113441>

Akhtar, H., Patel, C., Abuelgasim, E., & Harky, A. (2020). COVID-19 (SARS-CoV-2) Infection in Pregnancy: A Systematic Review. *Gynecologic and Obstetric Investigation*, *85*(4), 295-306. <https://doi.org/10.1159/000509290>

Amaral, W. N. D., Moraes, C. L., Rodrigues, A., Noll, M., Arruda, J. T., & Mendonça, C. R. (2020). Maternal Coronavirus Infections and Neonates Born to Mothers with SARS-CoV-2: A Systematic Review. *Healthcare (Basel)*, *8*(4). <https://doi.org/10.3390/healthcare8040511>

Araújo, L. A., Veloso, C. F., Souza, M. C., Azevedo, J. M. C., & Tarro, G. (2020). The potential impact of the COVID-19 pandemic on child growth and development: a systematic review. *Jornal de Pediatría*. <https://doi.org/10.1016/j.jped.2020.08.008>

Ashraf, M. A., Keshavarz, P., Hosseinpour, P., Erfani, A., Roshanshad, A., Pourdast, A., . . . Poordast, T. (2020). Coronavirus Disease 2019 (COVID-19): A Systematic Review of Pregnancy and the Possibility of Vertical Transmission. *J Reprod Infertil*, *21*(3), 157-168.

Capobianco, G., Saderi, L., Aliberti, S., Mondoni, M., Piana, A., Dessole, F., . . . Sotgiu, G. (2020). COVID-19 in pregnant women: A systematic review and meta-analysis. *European Journal of Obstetrics, Gynecology, and Reproductive Biology*, *252*, 543-558. <https://doi.org/10.1016/j.ejogrb.2020.07.006>

Chi, J., Gong, W., & Gao, Q. (2020). Clinical characteristics and outcomes of pregnant women with COVID-19 and the risk of vertical transmission: a systematic review. *Archives of Gynecology and Obstetrics*, 1-9. <https://doi.org/10.1007/s00404-020-05889-5>

Chmielewska, B., Barratt, I., Townsend, R., Kalafat, E., van der Meulen, J., Gurol-Urganci, I., . . . Khalil, A. (2021). Effects of the COVID-19 pandemic on maternal and perinatal outcomes: a systematic review and meta-analysis. *Lancet Glob Health*, *9*(6), e759-e772. <https://doi.org/10.1016/s2214-109x(21)00079-6>

Di Mascio, D., Khalil, A., Saccone, G., Rizzo, G., Buca, D., Liberati, M., . . . D'Antonio, F. (2020). Outcome of coronavirus spectrum infections (SARS, MERS, COVID-19) during pregnancy: a systematic review and meta-analysis. *Am J Obstet Gynecol MFM*, *2*(2), 100107. <https://doi.org/10.1016/j.ajogmf.2020.100107>

Diriba, K., Awulachew, E., & Getu, E. (2020). The effect of coronavirus infection (SARS-CoV-2, MERS-CoV, and SARS-CoV) during pregnancy and the possibility of vertical maternal-fetal transmission: a systematic review and meta-analysis. *European Journal of Medical Research*, *25*(1), 39. <https://doi.org/10.1186/s40001-020-00439-w>

Fan, S., Guan, J., Cao, L., Wang, M., Zhao, H., Chen, L., & Yan, L. (2021). Psychological effects caused by COVID-19 pandemic on pregnant women: A systematic review with meta-analysis. *Asian Journal of Psychiatry*, *56*, 102533. <https://doi.org/10.1016/j.ajp.2020.102533>

Guo, C.-X., He, L., Yin, J.-Y., Meng, X.-G., Tan, W., Yang, G.-P., . . . Chen, X. (2020). Epidemiological and clinical features of pediatric COVID-19. *BMC Medicine*, *18*(1), 1-7. <https://doi.org/10.1186/s12916-020-01719-2>

Hessami, K., Homayoon, N., Hashemi, A., Vafaei, H., Kasraeian, M., & Asadi, N. (2020). COVID-19 and maternal, fetal and neonatal mortality: a systematic review. *Journal of Maternal-Fetal & Neonatal Medicine*, 1-6. <https://doi.org/10.1080/14767058.2020.1806817>

Hessami, K., Romanelli, C., Chiurazzi, M., & Cozzolino, M. (2020). COVID-19 pandemic and maternal mental health: a systematic review and meta-analysis. *Journal of Maternal-Fetal & Neonatal Medicine*, 1-8. <https://doi.org/10.1080/14767058.2020.1843155>

Huntley, B. J. F., Huntley, E. S., Di Mascio, D., Chen, T., Berghella, V., & Chauhan, S. P. (2020). Rates of Maternal and Perinatal Mortality and Vertical Transmission in Pregnancies Complicated by Severe Acute Respiratory Syndrome Coronavirus 2 (SARS-Co-V-2) Infection: A Systematic Review. *Obstetrics and Gynecology*, *136*(2), 303-312. <https://doi.org/10.1097/AOG.0000000000004010>

Islam, M. M., Poly, T. N., Walther, B. A., Yang, H. C., Wang, C. W., Hsieh, W. S., . . . Jack Li, Y. C. (2020). Clinical Characteristics and Neonatal Outcomes of Pregnant Patients With COVID-19: A Systematic Review. *Front Med (Lausanne)*, *7*, 573468. <https://doi.org/10.3389/fmed.2020.573468>

Jafari, M., Pormohammad, A., Sheikh Neshin, S. A., Ghorbani, S., Bose, D., Alimohammadi, S., . . . Zarei, M. (2021). Clinical characteristics and outcomes of pregnant women with COVID-19 and comparison with control patients: A systematic review and meta-analysis. *Reviews in Medical Virology*, e2208. <https://doi.org/10.1002/rmv.2208>

Jones, E. A. K., Mitra, A. K., & Bhuiyan, A. R. (2021). Impact of COVID-19 on Mental Health in Adolescents: A Systematic Review. *International Journal of Environmental Research and Public Health*, *18*(5). <https://doi.org/10.3390/ijerph18052470>

Juan, J., Gil, M. M., Rong, Z., Zhang, Y., Yang, H., & Poon, L. C. (2020). Effect of coronavirus disease 2019 (COVID-19) on maternal, perinatal and neonatal outcome: systematic review. *Ultrasound in Obstetrics and Gynecology*, *56*(1), 15-27. <https://doi.org/10.1002/uog.22088>

Mark, E. G., Golden, W. C., Gilmore, M. M., Sick-Samuels, A., Curless, M. S., Nogee, L. M., . . . Johnson, J. (2021). Community-Onset Severe Acute Respiratory Syndrome Coronavirus 2 Infection in Young Infants: A Systematic Review. *Journal of Pediatrics*, *228*, 94-100.e103. <https://doi.org/10.1016/j.jpeds.2020.09.008>

Muhidin, S., Behboodi Moghadam, Z., & Vizheh, M. (2020). Analysis of Maternal Coronavirus Infections and Neonates Born to Mothers with 2019-nCoV; a Systematic Review. *Arch Acad Emerg Med*, *8*(1), e49.

Papapanou, M., Papaioannou, M., Petta, A., Routsi, E., Farmaki, M., Vlahos, N., & Siristatidis, C. (2021). Maternal and Neonatal Characteristics and Outcomes of COVID-19 in Pregnancy: An Overview of Systematic Reviews. *International Journal of Environmental Research and Public Health*, *18*(2). <https://doi.org/10.3390/ijerph18020596>

Shorey, S. Y., Ng, E. D., & Chee, C. Y. I. (2021). Anxiety and depressive symptoms of women in the perinatal period during the COVID-19 pandemic: A systematic review and meta-analysis. *Scand J Public Health*, 14034948211011793. <https://doi.org/10.1177/14034948211011793>

Smith, V., Seo, D., Warty, R., Payne, O., Salih, M., Chin, K. L., . . . Wallace, E. (2020). Maternal and neonatal outcomes associated with COVID-19 infection: A systematic review. *PloS One*, *15*(6), e0234187. <https://doi.org/10.1371/journal.pone.0234187>

Stavridou, A., Stergiopoulou, A. A., Panagouli, E., Mesiris, G., Thirios, A., Mougiakos, T., . . . Tsitsika, A. (2020). Psychosocial consequences of COVID-19 in children, adolescents and young adults: A systematic review. *Psychiatry and Clinical Neurosciences*, *74*(11), 615-616. <https://doi.org/10.1111/pcn.13134>

Sun, F., Zhu, J., Tao, H., Ma, Y., & Jin, W. (2020). A systematic review involving 11,187 participants evaluating the impact of COVID-19 on anxiety and depression in pregnant women. *Journal of Psychosomatic Obstetrics and Gynecology*. <https://doi.org/10.1080/0167482X.2020.1857360>

Teles Abrao Trad, A., Ibirogba, E. R., Elrefaei, A., Narang, K., Tonni, G., Picone, O., . . . Ruano, R. (2020). Complications and outcomes of SARS-CoV-2 in pregnancy: where and what is the evidence? *Hypertension in Pregnancy*, *39*(3), 361-369. <https://doi.org/10.1080/10641955.2020.1769645>

Tomfohr-Madsen, L. M., Racine, N., Giesbrecht, G. F., Lebel, C., & Madigan, S. (2021). Depression and anxiety in pregnancy during COVID-19: A rapid review and meta-analysis. *Psychiatry Research*, *300*, 113912. <https://doi.org/10.1016/j.psychres.2021.113912>

V, C. F., & Iarocci, G. (2020). Child and Family Outcomes Following Pandemics: A Systematic Review and Recommendations on COVID-19 Policies. *Journal of Pediatric Psychology*, *45*(10), 1124-1143. <https://doi.org/10.1093/jpepsy/jsaa092>

Walker, K. F., O'Donoghue, K., Grace, N., Dorling, J., Comeau, J. L., Li, W., & Thornton, J. G. (2020). Maternal transmission of SARS-COV-2 to the neonate, and possible routes for such transmission: a systematic review and critical analysis. *BJOG: An International Journal of Obstetrics and Gynaecology*, *127*(11), 1324-1336. <https://doi.org/10.1111/1471-0528.16362>

Yan, H., Ding, Y., & Guo, W. (2020). Mental Health of Pregnant and Postpartum Women During the Coronavirus Disease 2019 Pandemic: A Systematic Review and Meta-Analysis. *Frontiers in Psychology*, *11*, 617001. <https://doi.org/10.3389/fpsyg.2020.617001>

Yoon, S., Kang, J., & Ahn, J. (2020). Clinical outcomes of 201 neonates born to mothers with COVID-19: a systematic review. *Eur Rev Med Pharmacol Sci*, *24*(14), 7804-7815.

Zaigham, M., & Andersson, O. (2020). Maternal and perinatal outcomes with COVID-19: A systematic review of 108 pregnancies. *Acta Obstetricia et Gynecologica Scandinavica*, *99*(7), 823-829. <https://doi.org/10.1111/aogs.13867>
