# Supplementary material for: Impact of the COVID-19 pandemic on maternal mental health, early childhood development, and parental practices: a global scoping review
Source: BMC Public Health. 2023 Feb 24;23:388. doi: 10.1186/s12889-023-15003-4 (PMC9950022; doi:10.1186/s12889-023-15003-4)
Supplement: Supplementary file 1 — Additional file 1. [file 12889_2023_15003_MOESM1_ESM.zip › 12889_2023_15003_MOESM6_ESM.docx]

**References**

Abasse, S., Essabar, L., Costin, T., Mahisatra, V., Kaci, M., Braconnier, A., . . . Fayssoil, A. (2020). Neonatal COVID-19 Pneumonia: Report of the First Case in a Preterm Neonate in Mayotte, an Overseas Department of France. *Children (Basel)*, *7*(8). https://doi.org/10.3390/children7080087

Abedzadeh-Kalahroudi, M., Sehat, M., Vahedpour, Z., & Talebian, P. (2021). Maternal and neonatal outcomes of pregnant patients with COVID-19: A prospective cohort study. *International Journal of Gynaecology and Obstetrics*, *153*(3), 449-456. https://doi.org/10.1002/ijgo.13661

Abou Ghayda, R., Li, H., Lee, K. H., Lee, H. W., Hong, S. H., Kwak, M., . . . Shin, J. I. (2020). COVID-19 and Adverse Pregnancy Outcome: A Systematic Review of 104 Cases. *J Clin Med*, *9*(11). https://doi.org/10.3390/jcm9113441

Achterberg, M., Dobbelaar, S., Boer, O. D., & Crone, E. A. (2021). Perceived stress as mediator for longitudinal effects of the COVID-19 lockdown on wellbeing of parents and children. *Scientific Reports*, *11*(1), 2971. https://doi.org/10.1038/s41598-021-81720-8

Adadms, E. L., Smith, D., Caccavale, L. J., & Bean, M. K. (2020). Parents are stressed! Patterns of parent stress across COVID-19. *Res Sq*. https://doi.org/10.21203/rs.3.rs-66730/v2

Ademhan Tural, D., Emiralioglu, N., Tural Hesapcioglu, S., Karahan, S., Ozsezen, B., Sunman, B., . . . Kiper, N. (2020). Psychiatric and general health effects of COVID-19 pandemic on children with chronic lung disease and parents' coping styles. *Pediatric Pulmonology*, *55*(12), 3579-3586. https://doi.org/10.1002/ppul.25082

Ahlers-Schmidt, C. R., Hervey, A. M., Neil, T., Kuhlmann, S., & Kuhlmann, Z. (2020). Concerns of women regarding pregnancy and childbirth during the COVID-19 pandemic. *Patient Education and Counseling*, *103*(12), 2578-2582. https://doi.org/10.1016/j.pec.2020.09.031

Ahorsu, D. K., Imani, V., Lin, C. Y., Timpka, T., Broström, A., Updegraff, J. A., . . . Pakpour, A. H. (2020). Associations Between Fear of COVID-19, Mental Health, and Preventive Behaviours Across Pregnant Women and Husbands: An Actor-Partner Interdependence Modelling. *Int J Ment Health Addict*, 1-15. https://doi.org/10.1007/s11469-020-00340-x

Aimen, C., Bari, A., Rashid, J., Alvi, Y., Naz, F., Rana, N., . . . Sadiq, M. (2020). Comorbidity and covid-19 in children-a single center experience. *Pakistan Paediatric Journal*, *44*(4), 306-313.

Akgor, U., Fadıloglu, E., Soyak, B., Unal, C., Cagan, M., Temiz, B. E., . . . Ozyuncu, O. (2021). Anxiety, depression and concerns of pregnant women during the COVID-19 pandemic. *Archives of Gynecology and Obstetrics*, *304*(1), 125-130. https://doi.org/10.1007/s00404-020-05944-1

Akhtar, H., Patel, C., Abuelgasim, E., & Harky, A. (2020). COVID-19 (SARS-CoV-2) Infection in Pregnancy: A Systematic Review. *Gynecologic and Obstetric Investigation*, *85*(4), 295-306. https://doi.org/10.1159/000509290

Aksoy Derya, Y., Altiparmak, S., AkÇa, E., GÖkbulut, N., & Yilmaz, A. N. (2020). Pregnancy and birth planning during COVID-19: The effects of tele-education offered to pregnant women on prenatal distress and pregnancy-related anxiety. *Midwifery*, *92*, 102877. https://doi.org/10.1016/j.midw.2020.102877

Al-Matary, A., Almatari, F., Al-Matary, M., AlDhaefi, A., Alqahtani, M. H. S., Alhulaimi, E. A., . . . Aldandan, F. K. (2021). Clinical outcomes of maternal and neonate with COVID-19 infection - Multicenter study in Saudi Arabia. *J Infect Public Health*, *14*(6), 702-708. https://doi.org/10.1016/j.jiph.2021.03.013

Alay, I., Yildiz, S., Kaya, C., Yasar, K. K., Aydin, O. A., Karaosmanoglu, H. K., . . . Ekin, M. (2020). The clinical findings and outcomes of symptomatic pregnant women diagnosed with or suspected of having coronavirus disease 2019 in a tertiary pandemic hospital in Istanbul, Turkey. *Journal of Obstetrics and Gynaecology Research*. https://doi.org/10.1111/jog.14493

Alaya, F., Worrall, A. P., O'Toole, F., Doyle, J., Duffy, R. M., & Geary, M. P. (2021). Health-related quality of life and quality of care in pregnant and postnatal women during the coronavirus disease 2019 pandemic: A cohort study. *International Journal of Gynaecology and Obstetrics*. https://doi.org/10.1002/ijgo.13711

Alhuzimi, T. (2021). Stress and emotional wellbeing of parents due to change in routine for children with Autism Spectrum Disorder (ASD) at home during COVID-19 pandemic in Saudi Arabia. *Research in Developmental Disabilities*, *108*, 103822. https://doi.org/10.1016/j.ridd.2020.103822

Amaral, W. N. D., Moraes, C. L., Rodrigues, A., Noll, M., Arruda, J. T., & Mendonça, C. R. (2020). Maternal Coronavirus Infections and Neonates Born to Mothers with SARS-CoV-2: A Systematic Review. *Healthcare (Basel)*, *8*(4). https://doi.org/10.3390/healthcare8040511

An, R., Chen, X., Wu, Y., Liu, J., Deng, C., Liu, Y., & Guo, H. (2021). A survey of postpartum depression and health care needs among Chinese postpartum women during the pandemic of COVID-19. *Archives of Psychiatric Nursing*, *35*(2), 172-177. https://doi.org/10.1016/j.apnu.2021.02.001

Antoun, L., Taweel, N. E., Ahmed, I., Patni, S., & Honest, H. (2020). Maternal COVID-19 infection, clinical characteristics, pregnancy, and neonatal outcome: A prospective cohort study. *European Journal of Obstetrics, Gynecology, and Reproductive Biology*, *252*, 559-562. https://doi.org/10.1016/j.ejogrb.2020.07.008

Araújo, L. A., Veloso, C. F., Souza, M. C., Azevedo, J. M. C., & Tarro, G. (2020). The potential impact of the COVID-19 pandemic on child growth and development: a systematic review. *Jornal de Pediatría*. https://doi.org/10.1016/j.jped.2020.08.008

Ares, G., Bove, I., Vidal, L., Brunet, G., Fuletti, D., Arroyo, Á., & Blanc, M. V. (2021). The experience of social distancing for families with children and adolescents during the coronavirus (COVID-19) pandemic in Uruguay: Difficulties and opportunities. *Child Youth Serv Rev*, *121*, 105906. https://doi.org/10.1016/j.childyouth.2020.105906

Aronu, A., Awoere, C., Obinna, N., Ndudi, O. E., & Josephat, C. (2020). Readiness to send children back to school in the COVID-19 pandemic: Maternal perception and preferences. *Journal of Clinical and Diagnostic Research*, *14*(12), SC06-SC10. https://doi.org/10.7860/JCDR/2020/46257.14364

Aronu, A. E., Chinawa, J. M., Nduagubam, O. C., Ossai, E. N., Chinawa, A. T., & Igwe, W. C. (2020). Maternal perception of masking in children as a preventive strategy for COVID-19 in Nigeria: A multicentre study. *PloS One*, *15*(11 November). https://doi.org/10.1371/journal.pone.0242650

Asai, K., Wakashima, K., Toda, S., & Koiwa, K. (2021). Fear of novel coronavirus disease (COVID-19) among pregnant and infertile women in Japan. *J Affect Disord Rep*, *4*, 100104. https://doi.org/10.1016/j.jadr.2021.100104

Ashini, A., Alsoufi, A., & Elhadi, M. (2021). Parental perception of neonatal ICU visitation during the COVID-19 pandemic. *International Journal of Gynaecology and Obstetrics*, *153*(3), 554-555. https://doi.org/10.1002/ijgo.13650

Ashraf, M. A., Keshavarz, P., Hosseinpour, P., Erfani, A., Roshanshad, A., Pourdast, A., . . . Poordast, T. (2020). Coronavirus Disease 2019 (COVID-19): A Systematic Review of Pregnancy and the Possibility of Vertical Transmission. *J Reprod Infertil*, *21*(3), 157-168.

Auðardóttir, A. M., & Rúdólfsdóttir, A. G. (2020). Chaos ruined the children's sleep, diet and behaviour: Gendered discourses on family life in pandemic times. *Gend Work Organ*. https://doi.org/10.1111/gwao.12519

Ayas, M., Ali Al Amadi, A. M. H., Khaled, D., & Alwaa, A. M. (2020). Impact of COVID-19 on the access to hearing health care services for children with cochlear implants: A survey of parents. *F1000Research*, *9*. https://doi.org/10.12688/f1000research.24915.1

Ayaz, R., Hocaoğlu, M., Günay, T., Yardımcı, O. D., Turgut, A., & Karateke, A. (2020). Anxiety and depression symptoms in the same pregnant women before and during the COVID-19 pandemic. *Journal of Perinatal Medicine*, *48*(9), 965-970. https://doi.org/10.1515/jpm-2020-0380

Aydin, R., & Aktaş, S. (2021). An investigation of women's pregnancy experiences during the covid-19 pandemic: A qualitative study. *International Journal of Clinical Practice*, e14418. https://doi.org/10.1111/ijcp.14418

Bao, X., Qu, H., Zhang, R., & Hogan, T. P. (2020). Modeling Reading Ability Gain in Kindergarten Children during COVID-19 School Closures. *International Journal of Environmental Research and Public Health*, *17*(17). https://doi.org/10.3390/ijerph17176371

Barbosa-Leiker, C., Smith, C. L., Crespi, E. J., Brooks, O., Burduli, E., Ranjo, S., . . . Gartstein, M. A. (2021). Stressors, coping, and resources needed during the COVID-19 pandemic in a sample of perinatal women. *BMC Pregnancy and Childbirth*, *21*(1), 171. https://doi.org/10.1186/s12884-021-03665-0

Basu, A., Kim, H. H., Basaldua, R., Choi, K. W., Charron, L., Kelsall, N., . . . Koenen, K. C. (2021). A cross-national study of factors associated with women's perinatal mental health and wellbeing during the COVID-19 pandemic. *PloS One*, *16*(4), e0249780. https://doi.org/10.1371/journal.pone.0249780

Bender, W. R., Srinivas, S., Coutifaris, P., Acker, A., & Hirshberg, A. (2020). The Psychological Experience of Obstetric Patients and Health Care Workers after Implementation of Universal SARS-CoV-2 Testing. *American Journal of Perinatology*, *37*(12), 1271-1279. https://doi.org/10.1055/s-0040-1715505

Bentenuto, A., Mazzoni, N., Giannotti, M., Venuti, P., & de Falco, S. (2021). Psychological impact of Covid-19 pandemic in Italian families of children with neurodevelopmental disorders. *Research in Developmental Disabilities*, *109*, 103840. https://doi.org/10.1016/j.ridd.2020.103840

Berard, M., Rattaz, C., Peries, M., Loubersac, J., Munir, K., & Baghdadli, A. (2021). Impact of containment and mitigation measures on children and youth with ASD during the COVID-19 pandemic: Report from the ELENA cohort. *Journal of Psychiatric Research*, *137*, 73-80. https://doi.org/10.1016/j.jpsychires.2021.02.041

Berthelot, N., Lemieux, R., Garon-Bissonnette, J., Drouin-Maziade, C., Martel, É., & Maziade, M. (2020). Uptrend in distress and psychiatric symptomatology in pregnant women during the coronavirus disease 2019 pandemic. *Acta Obstetricia et Gynecologica Scandinavica*, *99*(7), 848-855. https://doi.org/10.1111/aogs.13925

Biasucci, G., Cannalire, G., Raymond, A., Capra, M. E., Benenati, B., Vadacca, G., . . . Bonini, R. (2020). Safe Perinatal Management of Neonates Born to SARS-CoV-2 Positive Mothers at the Epicenter of the Italian Epidemic. *Frontiers in Pediatrics*, *8*. https://doi.org/10.3389/fped.2020.565522

Bıkmazer, A., Kadak, M. T., Görmez, V., Doğan, U., Aslankaya, Z. D., Bakır, F., . . . Öztürk, M. (2020). Parental psychological distress associated with COVID-19 outbreak: A large-scale multicenter survey from Turkey. *International Journal of Social Psychiatry*, 20764020970240. https://doi.org/10.1177/0020764020970240

Bin-Nun, A., Palmor-Haspal, S., Mimouni, F. B., Kasirer, Y., Hammerman, C., & Tuval-Moshiach, R. (2021). Infant delivery and maternal stress during the COVID-19 pandemic: a comparison of the well-baby versus neonatal intensive care environments. *Journal of Perinatology*, 1-7. https://doi.org/10.1038/s41372-021-01016-7

Birindwa, E. K., Mulumeoderhwa, G. M., Nyakio, O., Mbale, G. Q. M., Mushamuka, S. Z., Materanya, J. M., . . . Balaluka, G. B. (2021). A case study of the first pregnant woman with COVID-19 in Bukavu, eastern Democratic Republic of the Congo. *Maternal Health, Neonatology and Perinatology*, *7*(1). https://doi.org/10.1186/s40748-021-00127-5

Bo, H. X., Yang, Y., Chen, J., Zhang, M., Li, Y., Zhang, D. Y., . . . Xiang, Y. T. (2021). Prevalence of Depressive Symptoms Among Pregnant and Postpartum Women in China During the COVID-19 Pandemic. *Psychosomatic Medicine*, *83*(4), 345-350. https://doi.org/10.1097/psy.0000000000000904

Boekhorst, M., Muskens, L., Hulsbosch, L. P., Van Deun, K., Bergink, V., Pop, V. J. M., & van den Heuvel, M. I. (2021). The COVID-19 outbreak increases maternal stress during pregnancy, but not the risk for postpartum depression. *Arch Womens Ment Health*, 1-7. https://doi.org/10.1007/s00737-021-01104-9

Bradfield, Z., Wynter, K., Hauck, Y., Vasilevski, V., Kuliukas, L., Wilson, A. N., . . . Sweet, L. (2021). Experiences of receiving and providing maternity care during the COVID-19 pandemic in Australia: A five-cohort cross-sectional comparison. *PloS One*, *16*(3), e0248488. https://doi.org/10.1371/journal.pone.0248488

Brandt, J. S., Hill, J., Reddy, A., Schuster, M., Patrick, H. S., Rosen, T., . . . Ananth, C. V. (2020). Epidemiology of coronavirus disease 2019 in pregnancy: risk factors and associations with adverse maternal and neonatal outcomes. *American Journal of Obstetrics and Gynecology*. https://doi.org/10.1016/j.ajog.2020.09.043

Brik, M., Sandonis, M. A., Fernández, S., Suy, A., Parramon-Puig, G., Maiz, N., . . . Carreras, E. (2021). Psychological impact and social support in pregnant women during lockdown due to SARS-CoV2 pandemic: A cohort study. *Acta Obstetricia et Gynecologica Scandinavica*, *100*(6), 1026-1033. https://doi.org/10.1111/aogs.14073

Brisca, G., Vagelli, G., Tagliarini, G., Rotulo, A., Pirlo, D., Romanengo, M., & Piccotti, E. (2021). The impact of COVID-19 lockdown on children with medical complexity in pediatric emergency department. *American Journal of Emergency Medicine*, *42*, 225-227. https://doi.org/10.1016/j.ajem.2020.11.066

Brown, A., & Shenker, N. (2020). Experiences of breastfeeding during COVID-19: Lessons for future practical and emotional support. *Maternal & Child Nutrition*, e13088. https://doi.org/10.1111/mcn.13088

Brown, S. M., Doom, J. R., Lechuga-Peña, S., Watamura, S. E., & Koppels, T. (2020). Stress and parenting during the global COVID-19 pandemic. *Child Abuse and Neglect*, 104699. https://doi.org/10.1016/j.chiabu.2020.104699

Buonsenso, D., Iodice, F., Cinicola, B., Raffaelli, F., Sowa, S., & Ricciardi, W. (2020). Management of Malaria in Children Younger Than 5 Years Old During Coronavirus Disease 2019 Pandemic in Sierra Leone: A Lesson Learned? *Frontiers in Pediatrics*, *8*. https://doi.org/10.3389/fped.2020.587638

Cacioppo, M., Bouvier, S., Bailly, R., Houx, L., Lempereur, M., Mensah-Gourmel, J., . . . Pons, C. (2021). Emerging health challenges for children with physical disabilities and their parents during the COVID-19 pandemic: The ECHO French survey. *64*(3). https://doi.org/10.1016/j.rehab.2020.08.001

Calvano, C., Engelke, L., Di Bella, J., Kindermann, J., Renneberg, B., & Winter, S. M. (2021). Families in the COVID-19 pandemic: parental stress, parent mental health and the occurrence of adverse childhood experiences-results of a representative survey in Germany. *European Child and Adolescent Psychiatry*, 1-13. https://doi.org/10.1007/s00787-021-01739-0

Camerlink, I., Nielsen, B. L., Windschnurer, I., & Vigors, B. (2021). Impacts of the COVID-19 pandemic on animal behaviour and welfare researchers. *Applied Animal Behaviour Science*, *236*, 105255. https://doi.org/10.1016/j.applanim.2021.105255

Cameron, E. E., Joyce, K. M., Delaquis, C. P., Reynolds, K., Protudjer, J. L. P., & Roos, L. E. (2020). Maternal psychological distress & mental health service use during the COVID-19 pandemic. *Journal of Affective Disorders*, *276*, 765-774. https://doi.org/10.1016/j.jad.2020.07.081

Campagnaro, R., Collet, G. O., Andrade, M. P., Salles, J., Calvo Fracasso, M. L., Scheffel, D. L. S., . . . Santin, G. C. (2020). COVID-19 pandemic and pediatric dentistry: Fear, eating habits and parent's oral health perceptions. *Child Youth Serv Rev*, *118*, 105469. https://doi.org/10.1016/j.childyouth.2020.105469

Cao, Y., Huang, L., Si, T., Wang, N. Q., Qu, M., & Zhang, X. Y. (2021). The role of only-child status in the psychological impact of COVID-19 on mental health of Chinese adolescents. *Journal of Affective Disorders*, *282*, 316-321. https://doi.org/10.1016/j.jad.2020.12.113

Capobianco, G., Saderi, L., Aliberti, S., Mondoni, M., Piana, A., Dessole, F., . . . Sotgiu, G. (2020). COVID-19 in pregnant women: A systematic review and meta-analysis. *European Journal of Obstetrics, Gynecology, and Reproductive Biology*, *252*, 543-558. https://doi.org/10.1016/j.ejogrb.2020.07.006

Carroll, N., Sadowski, A., Laila, A., Hruska, V., Nixon, M., Ma, D. W. L., . . . On Behalf Of The Guelph Family Health, S. (2020). The Impact of COVID-19 on Health Behavior, Stress, Financial and Food Security among Middle to High Income Canadian Families with Young Children. *Nutrients*, *12*(8). https://doi.org/10.3390/nu12082352

Celik, H., Acikel, S. B., Ozdemir, F. M. A., Aksoy, E., Oztoprak, U., Cucu, E., . . . Yuksel, D. (2021). Evaluation of the Anxiety Level of Mothers of Children with Epilepsy during the COVID-19 Pandemic Period. *European Neurology*, *84*(3), 192-199. https://doi.org/10.1159/000514826

Cellini, N., Di Giorgio, E., Mioni, G., & Di Riso, D. (2021). Sleep and Psychological Difficulties in Italian School-Age Children During COVID-19 Lockdown. *Journal of Pediatric Psychology*, *46*(2), 153-167. https://doi.org/10.1093/jpepsy/jsab003

Ceulemans, M., Foulon, V., Ngo, E., Panchaud, A., Winterfeld, U., Pomar, L., . . . Nordeng, H. (2021). Mental health status of pregnant and breastfeeding women during the COVID-19 pandemic-A multinational cross-sectional study. *Acta Obstetricia et Gynecologica Scandinavica*. https://doi.org/10.1111/aogs.14092

Ceulemans, M., Hompes, T., & Foulon, V. (2020). Mental health status of pregnant and breastfeeding women during the COVID-19 pandemic: A call for action. *International Journal of Gynaecology and Obstetrics*, *151*(1), 146-147. https://doi.org/10.1002/ijgo.13295

Chan, R. C. H., & Fung, S. C. (2021). Elevated Levels of COVID-19-Related Stress and Mental Health Problems Among Parents of Children with Developmental Disorders During the Pandemic. *Journal of Autism and Developmental Disorders*, 1-12. https://doi.org/10.1007/s10803-021-05004-w

Chasson, M., Taubman-Ben-Ari, O., & Abu-Sharkia, S. (2020). Jewish and Arab pregnant women's psychological distress during the COVID-19 pandemic: the contribution of personal resources. *Ethnicity and Health*, 1-13. https://doi.org/10.1080/13557858.2020.1815000

Chaves, C., Marchena, C., Palacios, B., Salgado, A., & Duque, A. (2021). Effects of the COVID-19 pandemic on perinatal mental health in Spain: Positive and negative outcomes. *Women Birth*. https://doi.org/10.1016/j.wombi.2021.01.007

Chen, H., Guo, J., Wang, C., Luo, F., Yu, X., Zhang, W., . . . Zhang, Y. (2020). Clinical characteristics and intrauterine vertical transmission potential of COVID-19 infection in nine pregnant women: a retrospective review of medical records. *Lancet*, *395*(10226), 809-815. https://doi.org/10.1016/s0140-6736(20)30360-3

Chen, H., Guo, J., Wang, C., Luo, F., Yu, X., Zhang, W., . . . Zhang, Y. (2020). Clinical characteristics and intrauterine vertical transmission potential of COVID-19 infection in nine pregnant women: a retrospective review of medical records. *Lancet*, *395 North American Edition*(10226), 809-815. https://doi.org/10.1016/S0140-6736(20)30360-3

Chen, S., Bernstein, P., Nair, S., Romanelli, E., Khoury, R., Labins, J., . . . Reddy, S. (2021). A review of 92 obstetric patients with COVID-19 in the Bronx, New York and their peripartum anaesthetic management. *Anaesthesiol Intensive Ther*, 1-11. https://doi.org/10.5114/ait.2021.105120

Chen, S. Q., Chen, S. D., Li, X. K., & Ren, J. (2020). Mental Health of Parents of Special Needs Children in China during the COVID-19 Pandemic. *International Journal of Environmental Research and Public Health*, *17*(24). https://doi.org/10.3390/ijerph17249519

Cheng, Z., Mendolia, S., Paloyo, A. R., Savage, D. A., & Tani, M. (2021). Working parents, financial insecurity, and childcare: mental health in the time of COVID-19 in the UK. *Rev Econ Househ*, 1-22. https://doi.org/10.1007/s11150-020-09538-3

Chi, J., Gong, W., & Gao, Q. (2020). Clinical characteristics and outcomes of pregnant women with COVID-19 and the risk of vertical transmission: a systematic review. *Archives of Gynecology and Obstetrics*, 1-9. https://doi.org/10.1007/s00404-020-05889-5

Chivers, B. R., Garad, R. M., Boyle, J. A., Skouteris, H., Teede, H. J., & Harrison, C. L. (2020). Perinatal Distress During COVID-19: Thematic Analysis of an Online Parenting Forum. *Journal of Medical Internet Research*, *22*(9), e22002. https://doi.org/10.2196/22002

Chmielewska, B., Barratt, I., Townsend, R., Kalafat, E., van der Meulen, J., Gurol-Urganci, I., . . . Khalil, A. (2021). Effects of the COVID-19 pandemic on maternal and perinatal outcomes: a systematic review and meta-analysis. *Lancet Glob Health*, *9*(6), e759-e772. https://doi.org/10.1016/s2214-109x(21)00079-6

Cho, E., & Ilari, B. S. (2021). Mothers as Home DJs: Recorded Music and Young Children's Well-Being During the COVID-19 Pandemic. *Frontiers in Psychology*, *12*, 637569. https://doi.org/10.3389/fpsyg.2021.637569

Choi, J., Park, Y., Kim, H. E., Song, J., Lee, D., Lee, E., . . . Lee, Y. (2021). Daily Life Changes and Life Satisfaction among Korean School-Aged Children in the COVID-19 Pandemic. *International Journal of Environmental Research and Public Health*, *18*(6). https://doi.org/10.3390/ijerph18063324

Chrzan-Dętkoś, M., Walczak-Kozłowska, T., & Lipowska, M. (2021). The need for additional mental health support for women in the postpartum period in the times of epidemic crisis. *BMC Pregnancy and Childbirth*, *21*(1), 114. https://doi.org/10.1186/s12884-021-03544-8

Chung, G., Lanier, P., & Wong, P. Y. J. (2020). Mediating Effects of Parental Stress on Harsh Parenting and Parent-Child Relationship during Coronavirus (COVID-19) Pandemic in Singapore. *J Fam Violence*, 1-12. https://doi.org/10.1007/s10896-020-00200-1

Cohodes, E. M., McCauley, S., & Gee, D. G. (2021). Parental Buffering of Stress in the Time of COVID-19: Family-Level Factors May Moderate the Association Between Pandemic-Related Stress and Youth Symptomatology. *Res Child Adolesc Psychopathol*, *49*(7), 935-948. https://doi.org/10.1007/s10802-020-00732-6

Colizzi, M., Sironi, E., Antonini, F., Ciceri, M. L., Bovo, C., & Zoccante, L. (2020). Psychosocial and behavioral impact of COVID-19 in autism spectrum disorder: An online parent survey. *Brain Sciences*, *10*(6). https://doi.org/10.3390/brainsci10060341

Congdon, J. L., Kair, L. R., Flaherman, V. J., Wood, K. E., LoFrumento, M. A., Nwaobasi-Iwuh, E., & Phillipi, C. A. (2021). Management and Early Outcomes of Neonates Born to Women with SARS-CoV-2 in 16 U.S. Hospitals. *American Journal of Perinatology*, *38*(6), 622-631. https://doi.org/10.1055/s-0041-1726036

Connell, C. M., & Strambler, M. J. (2021). Experiences With COVID-19 Stressors and Parents' Use of Neglectful, Harsh, and Positive Parenting Practices in the Northeastern United States. *Child Maltreat*, 10775595211006465. https://doi.org/10.1177/10775595211006465

Conti, M. G., Natale, F., Stolfi, I., Pedicino, R., Boscarino, G., Ajassa, C., . . . Terrin, G. (2021). Consequences of Early Separation of Maternal-Newborn Dyad in Neonates Born to SARS-CoV-2 Positive Mothers: An Observational Study. *International Journal of Environmental Research and Public Health*, *18*(11). https://doi.org/10.3390/ijerph18115899

Cooke, W. R., Billett, A., Gleeson, S., Jacques, A., Place, K., Siddall, J., . . . Soulsby, K. (2020). SARS-CoV-2 infection in very preterm pregnancy: Experiences from two cases. *European Journal of Obstetrics and Gynecology and Reproductive Biology*, *250*, 259-260. https://doi.org/10.1016/j.ejogrb.2020.05.025

Cooper, S. M., Thomas, A., & Bamishigbin, O. (2021). Black American Fathers Employed in Higher-Risk Contexts for Contracting COVID-19: Implications for Individual Wellbeing and Work-Family Spillover. *Am J Mens Health*, *15*(2), 15579883211005617. https://doi.org/10.1177/15579883211005617

Corbett, G. A., Milne, S. J., Hehir, M. P., Lindow, S. W., & O'Connell M, P. (2020). Health anxiety and behavioural changes of pregnant women during the COVID-19 pandemic. *European Journal of Obstetrics, Gynecology, and Reproductive Biology*, *249*, 96-97. https://doi.org/10.1016/j.ejogrb.2020.04.022

Costoya, V., Echeverría, L., Edo, M., Rocha, A., & Thailinger, A. (2021). Gender Gaps within Couples: Evidence of Time Re-allocations during COVID-19 in Argentina. *J Fam Econ Issues*, 1-14. https://doi.org/10.1007/s10834-021-09770-8

Covidence systematic review software, Veritas Health Innovation, Melbourne, Australia. Available at [www.covidence.org](http://www.covidence.org)

Craig, L., & Churchill, B. (2020). Dual-earner Parent Couples' Work and Care during COVID-19. *Gend Work Organ*. https://doi.org/10.1111/gwao.12497

Cui, S., Zhang, C., Wang, S., Zhang, X., Wang, L., Zhang, L., . . . Zhou, X. (2021). Experiences and Attitudes of Elementary School Students and Their Parents Toward Online Learning in China During the COVID-19 Pandemic: Questionnaire Study. *Journal of Medical Internet Research*, *23*(5), e24496. https://doi.org/10.2196/24496

Cui, Y., Li, Y., & Zheng, Y. (2020). Mental health services for children in China during the COVID-19 pandemic: results of an expert-based national survey among child and adolescent psychiatric hospitals. *European Child and Adolescent Psychiatry*, *29*(6), 743-748. https://doi.org/10.1007/s00787-020-01548-x

Cusinato, M., Iannattone, S., Spoto, A., Poli, M., Moretti, C., Gatta, M., & Miscioscia, M. (2020). Stress, Resilience, and Well-Being in Italian Children and Their Parents during the COVID-19 Pandemic. *International Journal of Environmental Research and Public Health*, *17*(22). https://doi.org/10.3390/ijerph17228297

Dagklis, T., Tsakiridis, I., Mamopoulos, A., Athanasiadis, A., Pearson, R., & Papazisis, G. (2020). Impact of the COVID-19 lockdown on antenatal mental health in Greece. *Psychiatry and Clinical Neurosciences*, *74*(11), 616-617. https://doi.org/10.1111/pcn.13135

Daks, J. S., Peltz, J. S., & Rogge, R. D. (2020). Psychological flexibility and inflexibility as sources of resiliency and risk during a pandemic: Modeling the cascade of COVID-19 stress on family systems with a contextual behavioral science lens. *J Contextual Behav Sci*, *18*, 16-27. https://doi.org/10.1016/j.jcbs.2020.08.003

Daulay, N. (2021). Home education for children with autism spectrum disorder during the COVID-19 pandemic: Indonesian mothers experience. *Research in Developmental Disabilities*, *114*, 103954. https://doi.org/10.1016/j.ridd.2021.103954

de Sá, C. D. S. C., Pombo, A., Luz, C., Rodrigues, L. P., & Cordovil, R. (2020). Covid-19 social isolation in Brazil: Effects on the physical activity routine of families with children. *Revista Paulista de Pediatria*, *39*. https://doi.org/10.1590/1984-0462/2021/39/2020159

Del Boca, D., Oggero, N., Profeta, P., & Rossi, M. (2020). Women's and men's work, housework and childcare, before and during COVID-19. *Rev Econ Househ*, 1-17. https://doi.org/10.1007/s11150-020-09502-1

Del Río, R., Dip Pérez, E., & Marín Gabriel, M. (2021). Multi-centre study showed reduced compliance with the World Health Organization recommendations on exclusive breastfeeding during COVID-19. *Acta Paediatrica*, *110*(3), 935-936. https://doi.org/10.1111/apa.15642

Dell'Utri, C., Manzoni, E., Cipriani, S., Spizzico, C., Dell'Acqua, A., Barbara, G., . . . Kustermann, A. (2020). Effects of SARS Cov-2 epidemic on the obstetrical and gynecological emergency service accesses. What happened and what shall we expect now? *European Journal of Obstetrics, Gynecology, and Reproductive Biology*, *254*, 64-68. https://doi.org/10.1016/j.ejogrb.2020.09.006

Dellagiulia, A., Lionetti, F., Fasolo, M., Verderame, C., Sperati, A., & Alessandri, G. (2020). Early impact of COVID-19 lockdown on children's sleep: A 4-week longitudinal study. *Journal of Clinical Sleep Medicine*, *16*(9), 1639-1640. https://doi.org/10.5664/jcsm.8648

DeYoung, S. E., & Mangum, M. (2021). Pregnancy, Birthing, and Postpartum Experiences During COVID-19 in the United States. *Front Sociol*, *6*, 611212. https://doi.org/10.3389/fsoc.2021.611212

Dhiman, S., Sahu, P. K., Reed, W. R., Ganesh, G. S., Goyal, R. K., & Jain, S. (2020). Impact of COVID-19 outbreak on mental health and perceived strain among caregivers tending children with special needs. *Research in Developmental Disabilities*, *107*. https://doi.org/10.1016/j.ridd.2020.103790

Di Mascio, D., Khalil, A., Saccone, G., Rizzo, G., Buca, D., Liberati, M., . . . D'Antonio, F. (2020). Outcome of coronavirus spectrum infections (SARS, MERS, COVID-19) during pregnancy: a systematic review and meta-analysis. *Am J Obstet Gynecol MFM*, *2*(2), 100107. https://doi.org/10.1016/j.ajogmf.2020.100107

Di Riso, D., Spaggiari, S., Cambrisi, E., Ferraro, V., Carraro, S., & Zanconato, S. (2021). Psychosocial impact of Covid-19 outbreak on Italian asthmatic children and their mothers in a post lockdown scenario. *Scientific Reports*, *11*(1), 9152. https://doi.org/10.1038/s41598-021-88152-4

Diaz, A., Baweja, R., & Bonatakis, J. K. (2021). Global health disparities in vulnerable populations of psychiatric patients during the COVID-19 pandemic. *World J Psychiatry*, *11*(4), 94-108. https://doi.org/10.5498/wjp.v11.i4.94

Dib, S., Rougeaux, E., Vázquez-Vázquez, A., Wells, J. C. K., & Fewtrell, M. (2020). Maternal mental health and coping during the COVID-19 lockdown in the UK: Data from the COVID-19 New Mum Study. *International Journal of Gynaecology and Obstetrics*, *151*(3), 407-414. https://doi.org/10.1002/ijgo.13397

Dickerson, J., Kelly, B., Lockyer, B., Bridges, S., Cartwright, C., Willan, K., . . . Pickett, K. E. (2020). Experiences of lockdown during the Covid-19 pandemic: descriptive findings from a survey of families in the Born in Bradford study. *Wellcome Open Res*, *5*, 228. https://doi.org/10.12688/wellcomeopenres.16317.2

Ding, W., Lu, J., Zhou, Y., Wei, W., Zhou, Z., & Chen, M. (2021). Knowledge, attitudes, practices, and influencing factors of anxiety among pregnant women in Wuhan during the outbreak of COVID-19: a cross-sectional study. *BMC Pregnancy and Childbirth*, *21*(1), 80. https://doi.org/10.1186/s12884-021-03561-7

Diriba, K., Awulachew, E., & Getu, E. (2020). The effect of coronavirus infection (SARS-CoV-2, MERS-CoV, and SARS-CoV) during pregnancy and the possibility of vertical maternal-fetal transmission: a systematic review and meta-analysis. *European Journal of Medical Research*, *25*(1), 39. https://doi.org/10.1186/s40001-020-00439-w

Dong, H., Hu, R., Lu, C., Huang, D., Cui, D., Huang, G., & Zhang, M. (2020). Investigation on the mental health status of pregnant women in China during the Pandemic of COVID-19. *Archives of Gynecology and Obstetrics*, 1-7. https://doi.org/10.1007/s00404-020-05805-x

du Fossé, N. A., Bronsgeest, K., Arbous, M. S., Zlei, M., Myeni, S. K., Kikkert, M., . . . van den Akker, T. (2021). Detailed immune monitoring of a pregnant woman with critical Covid-19. *Journal of Reproductive Immunology*, *143*. https://doi.org/10.1016/j.jri.2020.103243

Dule, A., Hajure, M., Mohammedhussein, M., & Abdu, Z. (2021). Health-related quality of life among Ethiopian pregnant women during COVID-19 pandemic. *Brain Behav*, *11*(4), e02045. https://doi.org/10.1002/brb3.2045

Dumbre, D. U., Ramesh, S., Chavan, R., & Jabade, M. (2020). A descriptive study to assess the stress and coping mechanism due to lockdown among school going children. *Indian Journal of Forensic Medicine and Toxicology*, *14*(4), 3590-3597. https://doi.org/10.37506/ijfmt.v14i4.12186

Durankuş, F., & Aksu, E. (2020). Effects of the COVID-19 pandemic on anxiety and depressive symptoms in pregnant women: a preliminary study. *Journal of Maternal-Fetal & Neonatal Medicine*, 1-7. https://doi.org/10.1080/14767058.2020.1763946

Effati-Daryani, F., Jahanfar, S., Mohammadi, A., Zarei, S., & Mirghafourvand, M. (2021). The relationship between sexual function and mental health in Iranian pregnant women during the COVID-19 pandemic. *BMC Pregnancy and Childbirth*, *21*(1), 327. https://doi.org/10.1186/s12884-021-03812-7

Effati-Daryani, F., Zarei, S., Mohammadi, A., Hemmati, E., Ghasemi Yngyknd, S., & Mirghafourvand, M. (2020). Depression, stress, anxiety and their predictors in Iranian pregnant women during the outbreak of COVID-19. *BMC Psychol*, *8*(1), 99. https://doi.org/10.1186/s40359-020-00464-8

Ehsan, S. M. A., & Jahan, F. (2021). Analysing the impact of COVID-19 on the mothers of Bangladesh: hearing the unheard. *Z Gesundh Wiss*, 1-14. https://doi.org/10.1007/s10389-021-01501-5

El-Osta, A., Alaa, A., Webber, I., Riboli Sasco, E., Bagkeris, E., Millar, H., . . . Majeed, A. (2021). How is the COVID-19 lockdown impacting the mental health of parents of school-age children in the UK? A cross-sectional online survey. *BMJ Open*, *11*(5), e043397. https://doi.org/10.1136/bmjopen-2020-043397

Ergenekon, A. P., Yilmaz Yegit, C., Cenk, M., Bas Ikizoglu, N., Atag, E., Gokdemir, Y., . . . Karadag, B. (2020). Depression and anxiety in mothers of home ventilated children before and during COVID-19 pandemic. *Pediatric Pulmonology*. https://doi.org/10.1002/ppul.25107

Evans, S., Mikocka-Walus, A., Klas, A., Olive, L., Sciberras, E., Karantzas, G., & Westrupp, E. M. (2020). From "It Has Stopped Our Lives" to "Spending More Time Together Has Strengthened Bonds": The Varied Experiences of Australian Families During COVID-19. *Frontiers in Psychology*, *11*, 588667. https://doi.org/10.3389/fpsyg.2020.588667

Faccioli, S., Lombardi, F., Bellini, P., Costi, S., Sassi, S., & Pesci, M. C. (2021). How did italian adolescents with disability and parents deal with the covid-19 emergency? *International Journal of Environmental Research and Public Health*, *18*(4), 1-13. https://doi.org/10.3390/ijerph18041687

Fallon, V., Davies, S. M., Silverio, S. A., Jackson, L., De Pascalis, L., & Harrold, J. A. (2021). Psychosocial experiences of postnatal women during the COVID-19 pandemic. A UK-wide study of prevalence rates and risk factors for clinically relevant depression and anxiety. *Journal of Psychiatric Research*, *136*, 157-166. https://doi.org/10.1016/j.jpsychires.2021.01.048

Fan, C., Guo, Y., Qu, P., Wang, S., Wang, M., Yuan, J., . . . Wang, B. (2020). No Obviously Adverse Pregnancy Complications and Outcomes of the Recovered Pregnant Women from COVID-19. *Reproductive Toxicology*. https://doi.org/10.1016/j.reprotox.2020.11.008

Fan, S., Guan, J., Cao, L., Wang, M., Zhao, H., Chen, L., & Yan, L. (2021). Psychological effects caused by COVID-19 pandemic on pregnant women: A systematic review with meta-analysis. *Asian Journal of Psychiatry*, *56*, 102533. https://doi.org/10.1016/j.ajp.2020.102533

Farewell, C. V., Jewell, J., Walls, J., & Leiferman, J. A. (2020). A Mixed-Methods Pilot Study of Perinatal Risk and Resilience During COVID-19. *Journal of Primary Care & Community Health*, *11*, 2150132720944074. https://doi.org/10.1177/2150132720944074

Farghaly, M. A. A., Kupferman, F., Castillo, F., & Kim, R. M. (2020). Characteristics of Newborns Born to SARS-CoV-2-Positive Mothers: A Retrospective Cohort Study. *American Journal of Perinatology*, *37*(13), 1310-1316. https://doi.org/10.1055/s-0040-1715862

Farhadi, R., Mehrpisheh, S., & Philip, R. K. (2021). Mobile-Assisted Virtual Bonding Enables Breast Milk Supply in Critically Ill Mothers With COVID-19: A Reflection on the Feasibility of Telelactation. *Cureus*, *13*(3), e13699. https://doi.org/10.7759/cureus.13699

Farrell, T., Reagu, S., Mohan, S., Elmidany, R., Qaddoura, F., Ahmed, E. E., . . . Alabdulla, M. A. (2020). The impact of the COVID-19 pandemic on the perinatal mental health of women. *Journal of Perinatal Medicine*, *48*(9), 971-976. https://doi.org/10.1515/jpm-2020-0415

Farsi, D., & Farsi, N. (2021). Mothers' Knowledge, Attitudes, and Fears About Dental Visits During the COVID-19 Pandemic: A Cross-sectional Study. *J Int Soc Prev Community Dent*, *11*(1), 83-91. https://doi.org/10.4103/jispcd.JISPCD_395_20

Farsi, Z., Taheriderakhsh, N., Bassirnia, M., Ahmadi, L., Shiva, M., & Yousefzadegan, S. (2020). Coronavirus disease-2019 infection in neonates of an infected pregnant mother with triplets. *Iranian Journal of Neonatology*, *11*(3), 120-122. https://doi.org/10.22038/ijn.2020.49218.1856

Feinberg, M. E., J, A. M., Lee, J. K., Tornello, S. L., Hostetler, M. L., Cifelli, J. A., . . . Hotez, E. (2021). Impact of the COVID-19 Pandemic on Parent, Child, and Family Functioning. *Family Process*. https://doi.org/10.1111/famp.12649

Fernandes, D. V., Canavarro, M. C., & Moreira, H. (2021). Postpartum during COVID-19 pandemic: Portuguese mothers' mental health, mindful parenting, and mother-infant bonding. *Journal of Clinical Psychology*. https://doi.org/10.1002/jclp.23130

Ferns, S. J., Gautam, S., & Hudak, M. L. (2021). COVID-19 and Gender Disparities in Pediatric Cardiologists with Dependent Care Responsibilities. *American Journal of Cardiology*, *147*, 137-142. https://doi.org/10.1016/j.amjcard.2021.02.017

Ferrante, M. J., Goldsmith, J., Tauriello, S., Epstein, L. H., Leone, L. A., & Anzman-Frasca, S. (2021). Food Acquisition and Daily Life for U.S. Families with 4-to 8-Year-Old Children during COVID-19: Findings from a Nationally Representative Survey. *International Journal of Environmental Research and Public Health*, *18*(4). https://doi.org/10.3390/ijerph18041734

Ferrazzi, E., Frigerio, L., Savasi, V., Vergani, P., Prefumo, F., Barresi, S., . . . Villa, A. (2020). Vaginal delivery in SARS-CoV-2-infected pregnant women in Northern Italy: a retrospective analysis. *BJOG: An International Journal of Obstetrics and Gynaecology*, *127*(9), 1116-1121. https://doi.org/10.1111/1471-0528.16278

Fisher, A. P., Patronick, J., Gerhardt, C. A., Radonovich, K., Salloum, R., & Wade, S. L. (2021). Impact of COVID-19 on adolescent and emerging adult brain tumor survivors and their parents. *Pediatric Blood & Cancer*, e29116. https://doi.org/10.1002/pbc.29116

Fontanella, F., Hannes, S., Keating, N., Martyn, F., Browne, I., Briet, J., . . . Baalman, J. H. (2020). COVID-19 infection during the third trimester of pregnancy: Current clinical dilemmas. *European Journal of Obstetrics and Gynecology and Reproductive Biology*, *251*, 268-271. https://doi.org/10.1016/j.ejogrb.2020.05.053

Forbes, L. K., Lamar, M. R., Speciale, M., & Donovan, C. (2021). Mothers' and fathers' parenting attitudes during COVID-19. *Current Psychology (New Brunswick, N.J.)*, 1-10. https://doi.org/10.1007/s12144-021-01605-x

Fosco, G. M., Sloan, C. J., Fang, S., & Feinberg, M. E. (2021). Family vulnerability and disruption during the COVID-19 pandemic: prospective pathways to child maladjustment. *Journal of Child Psychology and Psychiatry and Allied Disciplines*. https://doi.org/10.1111/jcpp.13458

Franchi, M., Del Piccolo, L., Bosco, M., Tosadori, C., Casarin, J., Laganà, A. S., & Garzon, S. (2020). COVID-19 and mental health in the obstetric population: a lesson from a case of puerperal psychosis. *Minerva Ginecologica*, *72*(5), 355-357. https://doi.org/10.23736/s0026-4784.20.04606-7

Freedman, R., Hunter, S. K., Law, A. J., D'Alessandro, A., Noonan, K., Wyrwa, A., & Camille Hoffman, M. (2020). Maternal choline and respiratory coronavirus effects on fetal brain development. *Journal of Psychiatric Research*, *128*, 1-4. https://doi.org/10.1016/j.jpsychires.2020.05.019

Freisthler, B., Gruenewald, P. J., Tebben, E., Shockley McCarthy, K., & Price Wolf, J. (2021). Understanding at-the-moment stress for parents during COVID-19 stay-at-home restrictions. *Social Science and Medicine*, *279*, 114025. https://doi.org/10.1016/j.socscimed.2021.114025

Fumagalli, S., Ornaghi, S., Borrelli, S., Vergani, P., & Nespoli, A. (2021). The experiences of childbearing women who tested positive to COVID-19 during the pandemic in northern Italy. *Women Birth*. https://doi.org/10.1016/j.wombi.2021.01.001

Gadermann, A. C., Thomson, K. C., Richardson, C. G., Gagné, M., McAuliffe, C., Hirani, S., & Jenkins, E. (2021). Examining the impacts of the COVID-19 pandemic on family mental health in Canada: findings from a national cross-sectional study. *BMJ Open*, *11*(1), e042871. https://doi.org/10.1136/bmjopen-2020-042871

Garcia de Avila, M. A., Hamamoto Filho, P. T., Jacob, F., Alcantara, L. R. S., Berghammer, M., Jenholt Nolbris, M., . . . Nilsson, S. (2020). Children's Anxiety and Factors Related to the COVID-19 Pandemic: An Exploratory Study Using the Children's Anxiety Questionnaire and the Numerical Rating Scale. *International Journal of Environmental Research and Public Health*, *17*(16). https://doi.org/10.3390/ijerph17165757

Gassman-Pines, A., Ananat, E. O., & Fitz-Henley, J., 2nd. (2020). COVID-19 and Parent-Child Psychological Well-being. *Pediatrics*, *146*(4). https://doi.org/10.1542/peds.2020-007294

Ge, Y., Shi, C., Wu, B., Liu, Y., Chen, L., & Deng, Y. (2021). Anxiety and Adaptation of Behavior in Pregnant Zhuang Women During the COVID-19 Pandemic: A Mixed-Mode Survey. *Risk Management and Healthcare Policy*, *14*, 1563-1573. https://doi.org/10.2147/rmhp.S303835

Ghema, K., Lehlimi, M., Toumi, H., Badre, A., Chemsi, M., Habzi, A., & Benomar, S. (2021). Outcomes of newborns to mothers with COVID-19. *Infect Dis Now*. https://doi.org/10.1016/j.idnow.2021.03.003

Gildner, T. E., Laugier, E. J., & Thayer, Z. M. (2020). Exercise routine change is associated with prenatal depression scores during the COVID-19 pandemic among pregnant women across the United States. *PloS One*, *15*(12), e0243188. https://doi.org/10.1371/journal.pone.0243188

Giurge, L. M., Whillans, A. V., & Yemiscigil, A. (2021). A multicountry perspective on gender differences in time use during COVID-19. *Proceedings of the National Academy of Sciences of the United States of America*, *118*(12). https://doi.org/10.1073/pnas.2018494118

Glynn, L. M., Davis, E. P., Luby, J. L., Baram, T. Z., & Sandman, C. A. (2021). A predictable home environment may protect child mental health during the COVID-19 pandemic. *Neurobiol Stress*, *14*, 100291. https://doi.org/10.1016/j.ynstr.2020.100291

Green, M. J., Pearce, A., Parkes, A., Robertson, E., & Katikireddi, S. V. (2021). Pre-school childcare and inequalities in child development. *SSM Popul Health*, *14*, 100776. https://doi.org/10.1016/j.ssmph.2021.100776

Grumi, S., Provenzi, L., Gardani, A., Aramini, V., Dargenio, E., Naboni, C., . . . Borgatti, R. (2020). Rehabilitation services lockdown during the COVID-19 emergency: the mental health response of caregivers of children with neurodevelopmental disabilities. *Disability and Rehabilitation*, 1-6. https://doi.org/10.1080/09638288.2020.1842520

Guo, C.-X., He, L., Yin, J.-Y., Meng, X.-G., Tan, W., Yang, G.-P., . . . Chen, X. (2020). Epidemiological and clinical features of pediatric COVID-19. *BMC Medicine*, *18*(1), 1-7. https://doi.org/10.1186/s12916-020-01719-2

Guo, J., De Carli, P., Lodder, P., Bakermans-Kranenburg, M. J., & Riem, M. M. E. (2021). Maternal mental health during the COVID-19 lockdown in China, Italy, and the Netherlands: a cross-validation study. *Psychological Medicine*, 1-11. https://doi.org/10.1017/s0033291720005504

Gur, R. E., White, L. K., Waller, R., Barzilay, R., Moore, T. M., Kornfield, S., . . . Elovitz, M. A. (2020). The Disproportionate Burden of the COVID-19 Pandemic Among Pregnant Black Women. *Psychiatry Research*, *293*, 113475. https://doi.org/10.1016/j.psychres.2020.113475

Guruge, S., Lamaj, P., Lee, C., Ronquillo, C. E., Sidani, S., Leung, E., . . . Morrison, L. (2021). COVID-19 restrictions: experiences of immigrant parents in Toronto. *AIMS Public Health*, *8*(1), 172-185. https://doi.org/10.3934/publichealth.2021013

Günther-Bel, C., Vilaregut, A., Carratala, E., Torras-Garat, S., & Pérez-Testor, C. (2020). A Mixed-method Study of Individual, Couple, and Parental Functioning During the State-regulated COVID-19 Lockdown in Spain. *Family Process*, *59*(3), 1060-1079. https://doi.org/10.1111/famp.12585

Hailemariam, S., Agegnehu, W., & Derese, M. (2021). Exploring COVID-19 Related Factors Influencing Antenatal Care Services Uptake: A Qualitative Study among Women in a Rural Community in Southwest Ethiopia. *Journal of Primary Care & Community Health*, *12*, 2150132721996892. https://doi.org/10.1177/2150132721996892

Halley, M. C., Mathews, K. S., Diamond, L. C., Linos, E., Sarkar, U., Mangurian, C., . . . Jagsi, R. (2021). The Intersection of Work and Home Challenges Faced by Physician Mothers During the Coronavirus Disease 2019 Pandemic: A Mixed-Methods Analysis. *J Womens Health (Larchmt)*, *30*(4), 514-524. https://doi.org/10.1089/jwh.2020.8964

Hamadani, J. D., Hasan, M. I., Baldi, A. J., Hossain, S. J., Shiraji, S., Bhuiyan, M. S. A., . . . Pasricha, S. R. (2020). Immediate impact of stay-at-home orders to control COVID-19 transmission on socioeconomic conditions, food insecurity, mental health, and intimate partner violence in Bangladeshi women and their families: an interrupted time series. *Lancet Glob Health*, *8*(11), e1380-e1389. https://doi.org/10.1016/s2214-109x(20)30366-1

Hammons, A. J., Villegas, E., & Robart, R. (2021). "It's Been Negative for Us Just All the Way Across the Board": Focus Group Study Exploring Parent Perceptions of Child Screen Time During the COVID-19 Pandemic. *JMIR Pediatr Parent*, *4*(2), e29411. https://doi.org/10.2196/29411

Hamzehgardeshi, Z., Omidvar, S., Amoli, A. A., & Firouzbakht, M. (2021). Pregnancy-related anxiety and its associated factors during COVID-19 pandemic in Iranian pregnant women: a web-based cross-sectional study. *BMC Pregnancy and Childbirth*, *21*(1), 208. https://doi.org/10.1186/s12884-021-03694-9

Handayani, & Dina, H. (2021). Analysis of self-empowerment of pregnant women during Covid-19 pandemic. *Pakistan Journal of Medical and Health Sciences*, *15*(1), 333-336.

Harahap, A., Harianto, A., Etika, R., Utomo, M. T., Angelika, D., Handayani, K. D., & Arif Sampurna, M. T. (2021). Spontaneous Ileum Perforation in a premature twin with Coronavirus-19 positive mother. *Journal of Pediatric Surgery Case Reports*, *67*. https://doi.org/10.1016/j.epsc.2021.101807

Harrison, V., Moulds, M. L., & Jones, K. (2021). Support from friends moderates the relationship between repetitive negative thinking and postnatal wellbeing during COVID-19. *Journal of Reproductive and Infant Psychology*, 1-16. https://doi.org/10.1080/02646838.2021.1886260

Hcini, N., Maamri, F., Picone, O., Carod, J. F., Lambert, V., Mathieu, M., . . . Pomar, L. (2021). Maternal, fetal and neonatal outcomes of large series of SARS-CoV-2 positive pregnancies in peripartum period: A single-center prospective comparative study. *European Journal of Obstetrics, Gynecology, and Reproductive Biology*, *257*, 11-18. https://doi.org/10.1016/j.ejogrb.2020.11.068

He, J. R., Xiao, Y. H., Ding, W., Shi, Y. L., He, X., Liu, X. D., . . . Qiu, X. (2021). Maternal, placental and neonatal outcomes after asymptomatic SARS-CoV-2 infection in the first trimester of pregnancy: A case report. *Case Reports in Women's Health*, *31*. https://doi.org/10.1016/j.crwh.2021.e00321

He, Z., Fang, Y., Zuo, Q., Huang, X., Lei, Y., Ren, X., & Liu, D. (2021). Vertical transmission and kidney damage in newborns whose mothers had coronavirus disease 2019 during pregnancy. *International Journal of Antimicrobial Agents*, *57*(2), 106260. https://doi.org/10.1016/j.ijantimicag.2020.106260

Herbert, J. S., Mitchell, A., Brentnall, S. J., & Bird, A. L. (2020). Identifying Rewards Over Difficulties Buffers the Impact of Time in COVID-19 Lockdown for Parents in Australia. *Frontiers in Psychology*, *11*, 606507. https://doi.org/10.3389/fpsyg.2020.606507

Hessami, K., Homayoon, N., Hashemi, A., Vafaei, H., Kasraeian, M., & Asadi, N. (2020). COVID-19 and maternal, fetal and neonatal mortality: a systematic review. *Journal of Maternal-Fetal & Neonatal Medicine*, 1-6. https://doi.org/10.1080/14767058.2020.1806817

Hessami, K., Romanelli, C., Chiurazzi, M., & Cozzolino, M. (2020). COVID-19 pandemic and maternal mental health: a systematic review and meta-analysis. *Journal of Maternal-Fetal & Neonatal Medicine*, 1-8. https://doi.org/10.1080/14767058.2020.1843155

Hiiragi, K., Obata, S., Misumi, T., Miyagi, E., & Aoki, S. (2021). Psychological stress associated with the COVID-19 pandemic in postpartum women in Yokohama, Japan. *Journal of Obstetrics and Gynaecology Research*. https://doi.org/10.1111/jog.14776

Hillis, Unwin, H. J. T., Chen, Y., Cluver, L., Sherr, L., Goldman, P. S., Ratmann, O., Donnelly, C. A., Bhatt, S., Villaveces, A., Butchart, A., Bachman, G., Rawlings, L., Green, P., Nelson, C. A., & Flaxman, S. (2021). Global minimum estimates of children affected by COVID-19-associated orphanhood and deaths of caregivers: a modelling study. The Lancet (British Edition), 398(10298), 391–402. <https://doi.org/10.1016/S0140-6736(21)01253-8>

Hiraoka, D., & Tomoda, A. (2020). Relationship between parenting stress and school closures due to the COVID-19 pandemic. *Psychiatry and Clinical Neurosciences*, *74*(9), 497-498. https://doi.org/10.1111/pcn.13088

Hjálmsdóttir, A., & Bjarnadóttir, V. S. (2020). "I have turned into a foreman here at home." Families and work-life balance in times of Covid-19 in a gender equality paradise. *Gend Work Organ*. https://doi.org/10.1111/gwao.12552

Hocaoglu, M., Ayaz, R., Gunay, T., Akin, E., Turgut, A., & Karateke, A. (2020). Anxiety and Post-Traumatic Stress Disorder Symptoms in Pregnant Women during the COVID-19 Pandemic's Delay Phase. *Psychiatr Danub*, *32*(3-4), 521-526. https://doi.org/10.24869/psyd.2020.521

Horiuchi, S., Shinohara, R., Otawa, S., Akiyama, Y., Ooka, T., Kojima, R., . . . Yamagata, Z. (2020). Caregivers' mental distress and child health during the COVID-19 outbreak in Japan. *PloS One*, *15*(12), e0243702. https://doi.org/10.1371/journal.pone.0243702

Hsu, A. L., Guan, M., Johannesen, E., Stephens, A. J., Khaleel, N., Kagan, N., . . . Wan, X. F. (2020). Placental SARS-CoV-2 in a pregnant woman with mild COVID-19 disease. *Journal of Medical Virology*. https://doi.org/10.1002/jmv.26386

Hu, X., Gao, J., Wei, Y., Chen, H., Sun, X., Chen, J., . . . Chen, L. (2020). Managing Preterm Infants Born to COVID-19 Mothers: Evidence from a Retrospective Cohort Study in Wuhan, China. *Neonatology*, 1-7. https://doi.org/10.1159/000509141

Huebener, M., Waights, S., Spiess, C. K., Siegel, N. A., & Wagner, G. G. (2021). Parental well-being in times of Covid-19 in Germany. *Rev Econ Househ*, 1-32. https://doi.org/10.1007/s11150-020-09529-4

Hui, P. W., Ma, G., Seto, M. T. Y., & Cheung, K. W. (2020). Effect of COVID-19 on delivery plans and postnatal depression scores of pregnant women. *Hong Kong Medical Journal. Xianggang Yi Xue Za Zhi*. https://doi.org/10.12809/hkmj208774

Huntley, B. J. F., Huntley, E. S., Di Mascio, D., Chen, T., Berghella, V., & Chauhan, S. P. (2020). Rates of Maternal and Perinatal Mortality and Vertical Transmission in Pregnancies Complicated by Severe Acute Respiratory Syndrome Coronavirus 2 (SARS-Co-V-2) Infection: A Systematic Review. *Obstetrics and Gynecology*, *136*(2), 303-312. https://doi.org/10.1097/AOG.0000000000004010

Hussong, A. M., Midgette, A. J., Thomas, T. E., Coffman, J. L., & Cho, S. (2021). Coping and Mental Health in Early Adolescence during COVID-19. *Res Child Adolesc Psychopathol*, 1-11. https://doi.org/10.1007/s10802-021-00821-0

Islam, M. M., Poly, T. N., Walther, B. A., Yang, H. C., Wang, C. W., Hsieh, W. S., . . . Jack Li, Y. C. (2020). Clinical Characteristics and Neonatal Outcomes of Pregnant Patients With COVID-19: A Systematic Review. *Front Med (Lausanne)*, *7*, 573468. https://doi.org/10.3389/fmed.2020.573468

Jafari, M., Pormohammad, A., Sheikh Neshin, S. A., Ghorbani, S., Bose, D., Alimohammadi, S., . . . Zarei, M. (2021). Clinical characteristics and outcomes of pregnant women with COVID-19 and comparison with control patients: A systematic review and meta-analysis. *Reviews in Medical Virology*, e2208. https://doi.org/10.1002/rmv.2208

Jak, B., Zanirati, G., Rodrigues, F. V. F., Grahl, M., Krimberg, F., Pinzetta, G., . . . Marinowic, D. R. (2021). Case Report: Placental Maternal Vascular Malperfusion Affecting Late Fetal Development and Multiorgan Infection Caused by SARS-CoV-2 in Patient With PAI-1 4G/5G Polymorphism. *Front Med (Lausanne)*, *8*, 624166. https://doi.org/10.3389/fmed.2021.624166

Janevic, T., Maru, S., Nowlin, S., McCarthy, K., Bergink, V., Stone, J., . . . Howell, E. A. (2021). Pandemic Birthing: Childbirth Satisfaction, Perceived Health Care Bias, and Postpartum Health During the COVID-19 Pandemic. *Matern Child Health J*, *25*(6), 860-869. https://doi.org/10.1007/s10995-021-03158-8

Jani, S., Jacques, S. M., Qureshi, F., Natarajan, G., Bajaj, S., Velumula, P., . . . Bajaj, M. (2021). Clinical Characteristics of Mother-Infant Dyad and Placental Pathology in COVID-19 Cases in Predominantly African American Population. *AJP Rep*, *11*(1), e15-e20. https://doi.org/10.1055/s-0040-1721673

Jansen, E., Thapaliya, G., Aghababian, A., Sadler, J., Smith, K., & Carnell, S. (2021). Parental stress, food parenting practices and child snack intake during the COVID-19 pandemic. *Appetite*, *161*, 105119. https://doi.org/10.1016/j.appet.2021.105119

Jelly, P., Chadha, L., Kaur, N., Sharma, S., Sharma, R., Stephen, S., & Rohilla, J. (2021). Impact of COVID-19 Pandemic on the Psychological Status of Pregnant Women. *Cureus*, *13*(1), e12875. https://doi.org/10.7759/cureus.12875

Jiang, H., Jin, L., Qian, X., Xiong, X., La, X., Chen, W., . . . Li, M. (2021). Maternal Mental Health Status and Approaches for Accessing Antenatal Care Information During the COVID-19 Epidemic in China: Cross-Sectional Study. *Journal of Medical Internet Research*, *23*(1), e18722. https://doi.org/10.2196/18722

Jiang, Y., He, T., Lin, X., Zhou, Q., & Wu, Q. (2021). Caregivers' joint depressive symptoms and preschoolers' daily routines in Chinese three-generation families: Does household chaos matter? *Current Psychology (New Brunswick, N.J.)*, 1-9. https://doi.org/10.1007/s12144-021-01595-w

Jones, E. A. K., Mitra, A. K., & Bhuiyan, A. R. (2021). Impact of COVID-19 on Mental Health in Adolescents: A Systematic Review. *International Journal of Environmental Research and Public Health*, *18*(5). https://doi.org/10.3390/ijerph18052470

Juan, J., Gil, M. M., Rong, Z., Zhang, Y., Yang, H., & Poon, L. C. (2020). Effect of coronavirus disease 2019 (COVID-19) on maternal, perinatal and neonatal outcome: systematic review. *Ultrasound in Obstetrics and Gynecology*, *56*(1), 15-27. https://doi.org/10.1002/uog.22088

Kachi, Y., Fujiwara, T., Eguchi, H., Inoue, A., Baba, S., Ohta, H., & Tsutsumi, A. (2021). Association between maternity harassment and depression during pregnancy amid the COVID-19 state of emergency. *J Occup Health*, *63*(1), e12196. https://doi.org/10.1002/1348-9585.12196

Kahyaoglu Sut, H., & Kucukkaya, B. (2020). Anxiety, depression, and related factors in pregnant women during the COVID-19 pandemic in Turkey: A web-based cross-sectional study. *Perspectives in Psychiatric Care*. https://doi.org/10.1111/ppc.12627

Kallander, S. W., Gordon, R., & Borzekowski, D. L. G. (2021). "People Will Continue to Suffer If the Virus Is Around": A Qualitative Analysis of Sub-Saharan African Children's Experiences during the COVID-19 Pandemic. *International Journal of Environmental Research and Public Health*, *18*(11). https://doi.org/10.3390/ijerph18115618

Kamali Aghdam, M., Jafari, N., & Eftekhari, K. (2020). Novel coronavirus in a 15-day-old neonate with clinical signs of sepsis, a case report. *Infect Dis (Lond)*, *52*(6), 427-429. https://doi.org/10.1080/23744235.2020.1747634

Kamity, R., Nayak, A., & Dumpa, V. (2021). Pneumothorax in Neonates Born to COVID-19-Positive Mothers: Fact or Fortuity? *AJP Rep*, *11*(1), e49-e53. https://doi.org/10.1055/s-0041-1726020

Kar, P., Tomfohr-Madsen, L., Giesbrecht, G., Bagshawe, M., & Lebel, C. (2021). Alcohol and substance use in pregnancy during the COVID-19 pandemic. *Drug and Alcohol Dependence*, *225*, 108760. https://doi.org/10.1016/j.drugalcdep.2021.108760

Kassaw, C., & Pandey, D. (2020). The prevalence of general anxiety disorder and its associated factors among women's attending at the perinatal service of Dilla University referral hospital, Dilla town, Ethiopia, April, 2020 in Covid pandemic. *Heliyon*, *6*(11), e05593. https://doi.org/10.1016/j.heliyon.2020.e05593

Katayama, Y., Zha, L., Kitamura, T., Hirayama, A., Takeuchi, T., Tanaka, K., . . . On Behalf Of The Covid-Epidemiology Research Group Of Osaka, U. (2021). Characteristics and Outcomes of Pediatric COVID-19 Patients in Osaka, Japan. *International Journal of Environmental Research and Public Health*, *18*(11). https://doi.org/10.3390/ijerph18115911

Kawamura, H., Orisaka, M., & Yoshida, Y. (2021). Mentality of pregnant women and obstetric healthcare workers about prenatal SARS-CoV-2 testing: A regional survey over the first wave of the COVID-19 pandemic in Japan. *Journal of Obstetrics and Gynaecology Research*, *47*(5), 1763-1771. https://doi.org/10.1111/jog.14740

Khamees, R. E., Taha, O. T., & Ali, T. Y. M. (2021). Anxiety and depression during pregnancy in the era of COVID-19. *Journal of Perinatal Medicine*. https://doi.org/10.1515/jpm-2021-0181

Khoury, J. E., Atkinson, L., Bennett, T., Jack, S. M., & Gonzalez, A. (2021). COVID-19 and mental health during pregnancy: The importance of cognitive appraisal and social support. *Journal of Affective Disorders*, *282*, 1161-1169. https://doi.org/10.1016/j.jad.2021.01.027

Khoury, R., Bernstein, P. S., Debolt, C., Stone, J., Sutton, D. M., Simpson, L. L., . . . Avila, K. (2020). Characteristics and Outcomes of 241 Births to Women With Severe Acute Respiratory Syndrome Coronavirus 2 (SARS-CoV-2) Infection at Five New York City Medical Centers. *Obstetrics and Gynecology*, *136*(2), 273-282. https://doi.org/10.1097/AOG.0000000000004025

Kim, L., Whitaker, M., O'Halloran, A., Kambhampati, A., Chai, S. J., Reingold, A., . . . Garg, S. (2020). Hospitalization Rates and Characteristics of Children Aged <18 Years Hospitalized with Laboratory-Confirmed COVID-19 - COVID-NET, 14 States, March 1-July 25, 2020. *MMWR: Morbidity and Mortality Weekly Report*, *69*(32), 1081-1088. https://doi.org/10.15585/mmwr.mm6932e3

Kimura, M., Kimura, K., & Ojima, T. (2021). Relationships between changes due to COVID-19 pandemic and the depressive and anxiety symptoms among mothers of infants and/or preschoolers: a prospective follow-up study from pre-COVID-19 Japan. *BMJ Open*, *11*(2), e044826. https://doi.org/10.1136/bmjopen-2020-044826

King, L. S., Feddoes, D. E., Kirshenbaum, J. S., Humphreys, K. L., & Gotlib, I. H. (2021). Pregnancy during the pandemic: the impact of COVID-19-related stress on risk for prenatal depression. *Psychological Medicine*, 1-11. https://doi.org/10.1017/s003329172100132x

Kinser, P. A., Jallo, N., Amstadter, A. B., Thacker, L. R., Jones, E., Moyer, S., . . . Salisbury, A. L. (2021). Depression, Anxiety, Resilience, and Coping: The Experience of Pregnant and New Mothers During the First Few Months of the COVID-19 Pandemic. *J Womens Health (Larchmt)*, *30*(5), 654-664. https://doi.org/10.1089/jwh.2020.8866

Kolkova, Z., Bjurström, M. F., Länsberg, J. K., Svedas, E., Hamer, M. A., Hansson, S. R., . . . Zaigham, M. (2020). Obstetric and intensive-care strategies in a high-risk pregnancy with critical respiratory failure due to COVID-19: A case report. *Case Reports in Women's Health*, *27*. https://doi.org/10.1016/j.crwh.2020.e00240

Korukcu, O., Ozkaya, M., Boran, O. F., & Bakacak, M. (2021). Factors associated with antenatal depression during the COVID-19 (SARS-CoV2) pandemic: A cross-sectional study in a cohort of Turkish pregnant women. *Perspectives in Psychiatric Care*. https://doi.org/10.1111/ppc.12778

Kotabagi, P., Fortune, L., Essien, S., Nauta, M., & Yoong, W. (2020). Anxiety and depression levels among pregnant women with COVID-19. *Acta Obstetricia et Gynecologica Scandinavica*, *99*(7), 953-954. https://doi.org/10.1111/aogs.13928

Kotabagi, P., Nauta, M., Fortune, L., & Yoong, W. (2020). COVID-19 positive mothers are not more anxious or depressed than non COVID pregnant women during the pandemic: A pilot case-control comparison. *European Journal of Obstetrics, Gynecology, and Reproductive Biology*, *252*, 615-616. https://doi.org/10.1016/j.ejogrb.2020.07.037

Kovler, M. L., Ziegfeld, S., Ryan, L. M., Goldstein, M. A., Gardner, R., Garcia, A. V., & Nasr, I. W. (2020). Increased proportion of physical child abuse injuries at a level I pediatric trauma center during the Covid-19 pandemic. *Child Abuse and Neglect*, 104756. https://doi.org/10.1016/j.chiabu.2020.104756

Koyucu, R. G., & Karaca, P. P. (2021). The Covid 19 outbreak: Maternal Mental Health and Associated Factors. *Midwifery*, *99*, 103013. https://doi.org/10.1016/j.midw.2021.103013

Kracht, C. L., Katzmarzyk, P. T., & Staiano, A. E. (2021). Household chaos, maternal stress, and maternal health behaviors in the United States during the COVID-19 outbreak. *Womens Health (Lond)*, *17*, 17455065211010655. https://doi.org/10.1177/17455065211010655

Kuhrt, K., McMicking, J., Nanda, S., Nelson-Piercy, C., & Shennan, A. (2020). Placental abruption in a twin pregnancy at 32 weeks’ gestation complicated by coronavirus disease 2019 without vertical transmission to the babies. *American Journal of Obstetrics and Gynecology MFM*, *2*(3). https://doi.org/10.1016/j.ajogmf.2020.100135

Kumari, A., Ranjan, P., Sharma, K. A., Sahu, A., Bharti, J., Zangmo, R., & Bhatla, N. (2021). Impact of COVID-19 on psychosocial functioning of peripartum women: A qualitative study comprising focus group discussions and in-depth interviews. *International Journal of Gynaecology and Obstetrics*, *152*(3), 321-327. https://doi.org/10.1002/ijgo.13524

Latorre, G., Martinelli, D., Guida, P., Masi, E., De Benedictis, R., & Maggio, L. (2021). Impact of COVID-19 pandemic lockdown on exclusive breastfeeding in non-infected mothers. *Int Breastfeed J*, *16*(1), 36. https://doi.org/10.1186/s13006-021-00382-4

Lauri Korajlija, A., & Jokic-Begic, N. (2020). COVID-19: Concerns and behaviours in Croatia. *British Journal of Health Psychology*. https://doi.org/10.1111/bjhp.12425

Lawson, M., Piel, M. H., & Simon, M. (2020). Child Maltreatment during the COVID-19 Pandemic: Consequences of Parental Job Loss on Psychological and Physical Abuse Towards Children. *Child Abuse and Neglect*, 104709. https://doi.org/10.1016/j.chiabu.2020.104709

Lebel, C., MacKinnon, A., Bagshawe, M., Tomfohr-Madsen, L., & Giesbrecht, G. (2020). Elevated depression and anxiety symptoms among pregnant individuals during the COVID-19 pandemic. *Journal of Affective Disorders*, *277*, 5-13. https://doi.org/10.1016/j.jad.2020.07.126

Lee, S. J., Ward, K. P., Chang, O. D., & Downing, K. M. (2020). Parenting Activities and the Transition to Home-based Education During the COVID-19 Pandemic. *Child Youth Serv Rev*, 105585. https://doi.org/10.1016/j.childyouth.2020.105585

Lee, S. J., Ward, K. P., Lee, J. Y., & Rodriguez, C. M. (2021). Parental Social Isolation and Child Maltreatment Risk during the COVID-19 Pandemic. *J Fam Violence*, 1-12. https://doi.org/10.1007/s10896-020-00244-3

Leeb, R. T., Bitsko, R. H., Radhakrishnan, L., Martinez, P., Njai, R., & Holland, K. M. (2020). Mental Health-Related Emergency Department Visits Among Children Aged <18 Years During the COVID-19 Pandemic - United States, January 1-October 17, 2020. *MMWR: Morbidity and Mortality Weekly Report*, *69*(45), 1675-1680. https://doi.org/10.15585/mmwr.mm6945a3

Lemieux, R., Garon-Bissonnette, J., Loiselle, M., Martel, É., Drouin-Maziade, C., & Berthelot, N. (2020). [Not Available]. *Canadian Journal of Psychiatry. Revue Canadienne de Psychiatrie*, 706743720963917. https://doi.org/10.1177/0706743720963917

Lemon, L., Edwards, R. P., & Simhan, H. N. (2021). What is driving the decreased incidence of preterm birth during the coronavirus disease 2019 pandemic? *Am J Obstet Gynecol MFM*, *3*(3), 100330. https://doi.org/10.1016/j.ajogmf.2021.100330

Li, C., Huo, L., Wang, R., Qi, L., Wang, W., Zhou, X., . . . Zhang, X. (2021). The prevalence and risk factors of depression in prenatal and postnatal women in China with the outbreak of Corona Virus Disease 2019. *Journal of Affective Disorders*, *282*, 1203-1209. https://doi.org/10.1016/j.jad.2021.01.019

Li, M., Yin, H., Jin, Z., Zhang, H., Leng, B., Luo, Y., & Zhao, Y. (2020). Impact of Wuhan lockdown on the indications of cesarean delivery and newborn weights during the epidemic period of COVID-19. *PloS One*, *15*(8), e0237420. https://doi.org/10.1371/journal.pone.0237420

Li, W., Wang, Z., Wang, G., Ip, P., Sun, X., Jiang, Y., & Jiang, F. (2021). Socioeconomic inequality in child mental health during the COVID-19 pandemic: First evidence from China. *Journal of Affective Disorders*, *287*, 8-14. https://doi.org/10.1016/j.jad.2021.03.009

Li, X., Lu, P., Hu, L., Huang, T., & Lu, L. (2020). Factors Associated with Mental Health Results among Workers with Income Losses Exposed to COVID-19 in China. *International Journal of Environmental Research and Public Health*, *17*(15). https://doi.org/10.3390/ijerph17155627

Liang, P., Wang, Y., Shi, S., Liu, Y., & Xiong, R. (2020). Prevalence and factors associated with postpartum depression during the COVID-19 pandemic among women in Guangzhou, China: a cross-sectional study. *BMC Psychiatry*, *20*(1), 557. https://doi.org/10.1186/s12888-020-02969-3

Liang, Z., Delvecchio, E., Cheng, Y., & Mazzeschi, C. (2021). Parent and Child's Negative Emotions During COVID-19: The Moderating Role of Parental Attachment Style. *Frontiers in Psychology*, *12*, 567483. https://doi.org/10.3389/fpsyg.2021.567483

Limbers, C. A., McCollum, C., & Greenwood, E. (2020). Physical activity moderates the association between parenting stress and quality of life in working mothers during the COVID-19 pandemic. *Ment Health Phys Act*, *19*, 100358. https://doi.org/10.1016/j.mhpa.2020.100358

Lin, W., Wu, B., Chen, B., Lai, G., Huang, S., Li, S., . . . Wang, Y. (2020). Sleep Conditions Associate with Anxiety and Depression Symptoms among Pregnant Women during the Epidemic of COVID-19 in Shenzhen. *Journal of Affective Disorders*. https://doi.org/10.1016/j.jad.2020.11.114

Linos, E., Halley, M. C., Sarkar, U., Mangurian, C., Sabry, H., Olazo, K., . . . Jagsi, R. (2021). Anxiety Levels Among Physician Mothers During the COVID-19 Pandemic. *American Journal of Psychiatry*, *178*(2), 203-204. https://doi.org/10.1176/appi.ajp.2020.20071014

Litmanovitz, I., Silberstein, D., Butler, S., & Vittner, D. (2021). Care of hospitalized infants and their families during the COVID-19 pandemic: an international survey. *Journal of Perinatology*, *41*(5), 981-987. https://doi.org/10.1038/s41372-021-00960-8

Liu, C. H., Erdei, C., & Mittal, L. (2020). Risk factors for depression, anxiety, and PTSD symptoms in perinatal women during the COVID-19 Pandemic. *Psychiatry Research*, 113552. https://doi.org/10.1016/j.psychres.2020.113552

Liu, C. H., Erdei, C., & Mittal, L. (2021). Risk factors for depression, anxiety, and PTSD symptoms in perinatal women during the COVID-19 Pandemic. *Psychiatry Research*, *295*, 113552. https://doi.org/10.1016/j.psychres.2020.113552

Liu, C. H., Mittal, L., & Erdei, C. (2021). COVID-19-related health worries compound the psychiatric distress experienced by families of high-risk infants. *Journal of Perinatology*, *41*(5), 1191-1195. https://doi.org/10.1038/s41372-021-01000-1

Liu, G., Wang, S., Liao, J., Ou, P., Huang, L., Xie, N., . . . Hu, R. (2021). The Efficacy of WeChat-Based Parenting Training on the Psychological Well-being of Mothers With Children With Autism During the COVID-19 Pandemic: Quasi-Experimental Study. *JMIR Ment Health*, *8*(2), e23917. https://doi.org/10.2196/23917

Liu, J., Hung, P., Alberg, A. J., Hair, N. L., Whitaker, K. M., Simon, J., & Taylor, S. K. (2021). Mental health among pregnant women with COVID-19-related stressors and worries in the United States. *Birth*. https://doi.org/10.1111/birt.12554

Liu, P., Zheng, J., Yang, P., Wang, X., Wei, C., Zhang, S., . . . Zhang, Y. (2020). The immunologic status of newborns born to SARS-CoV-2-infected mothers in Wuhan, China. *Journal of Allergy and Clinical Immunology*, *146*(1), 101-109.e101. https://doi.org/10.1016/j.jaci.2020.04.038

Liu, Q., Zhou, Y., Xie, X., Xue, Q., Zhu, K., Wan, Z., . . . Song, R. (2020). The prevalence of behavioral problems among school-aged children in home quarantine during the COVID-19 pandemic in china. *Journal of Affective Disorders*, *279*, 412-416. https://doi.org/10.1016/j.jad.2020.10.008

Liu, W., Cheng, H., Wang, J., Ding, L., Zhou, Z., Liu, S., . . . Rong, Z. (2020). Clinical Analysis of Neonates Born to Mothers with or without COVID-19: A Retrospective Analysis of 48 Cases from Two Neonatal Intensive Care Units in Hubei Province. *American Journal of Perinatology*, *37*(13), 1317-1323. https://doi.org/10.1055/s-0040-1716505

Liu, W., Wang, J., Li, W., Zhou, Z., Liu, S., & Rong, Z. (2020). Clinical characteristics of 19 neonates born to mothers with COVID-19. *Frontiers of Medicine*, *14*(2), 193-198. https://doi.org/10.1007/s11684-020-0772-y

Liu, X., Chen, M., Wang, Y., Sun, L., Zhang, J., Shi, Y., . . . Qi, H. (2020). Prenatal anxiety and obstetric decisions among pregnant women in Wuhan and Chongqing during the COVID-19 outbreak: a cross-sectional study. *BJOG: An International Journal of Obstetrics and Gynaecology*, *127*(10), 1229-1240. https://doi.org/10.1111/1471-0528.16381

Liu, Z., Tang, H., Jin, Q., Wang, G., Yang, Z., Chen, H., . . . Owens, J. (2020). Sleep of preschoolers during the coronavirus disease 2019 (COVID-19) outbreak. *Journal of Sleep Research*, e13142. https://doi.org/10.1111/jsr.13142

Lizama, O., Mucha, J., del Carmen Chincaro, M., Giraldo, G., Salazar, J., Agüero, K., . . . Espinoza, D. (2021). Pre and post-natal epidemiological and clinical features of neonates born from mothers infected with COVID-19 and 14-day follow-up post discharge in Lima, Peru. *Revista Medica Herediana*, *32*(1), 5-11. https://doi.org/10.20453/RMH.V32I1.3942

Lorentz, M. S., Chagas, L. B., Perez, A. V., da Silva Cassol, P. A., Vettorazzi, J., & Lubianca, J. N. (2021). Correlation between depressive symptoms and sexual dysfunction in postpartum women during the COVID-19 pandemic. *European Journal of Obstetrics, Gynecology, and Reproductive Biology*, *258*, 162-167. https://doi.org/10.1016/j.ejogrb.2020.12.039

Loret de Mola, C., Blumenberg, C., Martins, R. C., Martins-Silva, T., Carpena, M. X., Del-Ponte, B., . . . Cesar, J. A. (2021). Increased depression and anxiety during the COVID-19 pandemic in Brazilian mothers: a longitudinal study. *Braz J Psychiatry*, *43*(3), 337-338. https://doi.org/10.1590/1516-4446-2020-1628

Lv, Y., Gu, B., Chen, Y., Hu, S., Ruan, T., Xu, G., . . . Shen, X. (2020). No intrauterine vertical transmission in pregnancy with COVID-19: A case report. *Journal of Infection and Chemotherapy*, *26*(12), 1313-1315. https://doi.org/10.1016/j.jiac.2020.07.015

López-Morales, H., Del Valle, M. V., Canet-Juric, L., Andrés, M. L., Galli, J. I., Poó, F., & Urquijo, S. (2020). Mental health of pregnant women during the COVID-19 pandemic: A longitudinal study. *Psychiatry Research*, 113567. https://doi.org/10.1016/j.psychres.2020.113567

Maggs, J. L., Cassinat, J. R., Kelly, B. C., Mustillo, S. A., & Whiteman, S. D. (2021). Parents Who First Allowed Adolescents to Drink Alcohol in a Family Context During Spring 2020 COVID-19 Emergency Shutdowns. *Journal of Adolescent Health*, *68*(4), 816-818. https://doi.org/10.1016/j.jadohealth.2021.01.010

Mahajan, N. N., Ansari, M., Gaikwad, C., Jadhav, P., Tirkey, D., Pophalkar, M. P., . . . Gajbhiye, R. K. (2020). Impact of SARS-CoV-2 on multiple gestation pregnancy. *International Journal of Gynaecology and Obstetrics*. https://doi.org/10.1002/ijgo.13508

Maharlouei, N., Keshavarz, P., Salemi, N., & Lankarani, K. B. (2021). Depression and anxiety among pregnant mothers in the initial stage of the Coronavirus Disease (COVID-19) pandemic in the southwest of Iran. *Reprod Health*, *18*(1), 111. https://doi.org/10.1186/s12978-021-01167-y

Mahmoud, A. B., Hack-Polay, D., Fuxman, L., & Nicoletti, M. (2021). The Janus-faced effects of COVID-19 perceptions on family healthy eating behavior: Parent's negative experience as a mediator and gender as a moderator. *Scandinavian Journal of Psychology*. https://doi.org/10.1111/sjop.12742

Malkawi, S. H., Almhdawi, K., Jaber, A. F., & Alqatarneh, N. S. (2020). COVID-19 Quarantine-Related Mental Health Symptoms and their Correlates among Mothers: A Cross Sectional Study. *Matern Child Health J*, 1-11. https://doi.org/10.1007/s10995-020-03034-x

Mangiavacchi, L., Piccoli, L., & Pieroni, L. (2021). Fathers matter: Intrahousehold responsibilities and children's wellbeing during the COVID-19 lockdown in Italy. *Economics and Human Biology*, *42*, 101016. https://doi.org/10.1016/j.ehb.2021.101016

Mangolian Shahrbabaki, P., Dehghan, M., Maazallahi, M., & Asadi, N. (2021). Fear and anxiety in girls aged 7 to 11 years old and related factors during the coronavirus pandemic. *Clinical Child Psychology and Psychiatry*, 13591045211013873. https://doi.org/10.1177/13591045211013873

Manja, S. A., Mohamad, I., Ismail, H., & Yusof, N. I. (2020). COVID-19: The investigation on the emotional parental burnout during movement control order in Malaysia. *European Journal of Molecular and Clinical Medicine*, *7*(2), 4912-4929.

Mappa, I., Distefano, F. A., & Rizzo, G. (2020). Effects of coronavirus 19 pandemic on maternal anxiety during pregnancy: a prospectic observational study. *Journal of Perinatal Medicine*, *48*(6), 545-550. https://doi.org/10.1515/jpm-2020-0182

Marchetti, D., Fontanesi, L., Di Giandomenico, S., Mazza, C., Roma, P., & Verrocchio, M. C. (2020). The Effect of Parent Psychological Distress on Child Hyperactivity/Inattention During the COVID-19 Lockdown: Testing the Mediation of Parent Verbal Hostility and Child Emotional Symptoms. *Frontiers in Psychology*, *11*, 567052. https://doi.org/10.3389/fpsyg.2020.567052

Marchetti, D., Fontanesi, L., Mazza, C., Di Giandomenico, S., Roma, P., & Verrocchio, M. C. (2020). Parenting-Related Exhaustion During the Italian COVID-19 Lockdown. *Journal of Pediatric Psychology*, *45*(10), 1114-1123. https://doi.org/10.1093/jpepsy/jsaa093

Mariño-Narvaez, C., Puertas-Gonzalez, J. A., Romero-Gonzalez, B., & Peralta-Ramirez, M. I. (2021). Giving birth during the COVID-19 pandemic: The impact on birth satisfaction and postpartum depression. *International Journal of Gynaecology and Obstetrics*, *153*(1), 83-88. https://doi.org/10.1002/ijgo.13565

Mark, E. G., Golden, W. C., Gilmore, M. M., Sick-Samuels, A., Curless, M. S., Nogee, L. M., . . . Johnson, J. (2021). Community-Onset Severe Acute Respiratory Syndrome Coronavirus 2 Infection in Young Infants: A Systematic Review. *Journal of Pediatrics*, *228*, 94-100.e103. https://doi.org/10.1016/j.jpeds.2020.09.008

Mark, E. G., McAleese, S., Golden, W. C., Gilmore, M. M., Sick-Samuels, A., Curless, M. S., . . . Johnson, J. (2021). Coronavirus Disease 2019 in Pregnancy and Outcomes Among Pregnant Women and Neonates: A Literature Review. *Pediatric Infectious Disease Journal*, *40*(5), 473-478. https://doi.org/10.1097/inf.0000000000003102

Markovic, A., Mühlematter, C., Beaugrand, M., Camos, V., & Kurth, S. (2021). Severe effects of the COVID-19 confinement on young children's sleep: A longitudinal study identifying risk and protective factors. *Journal of Sleep Research*, e13314. https://doi.org/10.1111/jsr.13314

Martins-Filho, P. R., Damascena, N. P., Lage, R. C. M., & Sposato, K. B. (2020). Decrease in child abuse notifications during COVID-19 outbreak: A reason for worry or celebration? *Journal of Paediatrics and Child Health*, *56*(12), 1980-1981. https://doi.org/10.1111/jpc.15213

Martínez Pérez, A., López-Soler, C., Fernández-Fernández, V., Alcántara-López, M., & Castro Sáez, M. (2020). Preliminary results of the impact of COVID-19 on children and adolescents exposed to intrafamily abuse. *Terapia Psicologica*, *38*(3), 427-445. https://doi.org/10.4067/S0718-48082020000300427

Masters, G. A., Asipenko, E., Bergman, A. L., Person, S. D., Brenckle, L., Moore Simas, T. A., . . . Byatt, N. (2021). Impact of the COVID-19 pandemic on mental health, access to care, and health disparities in the perinatal period. *Journal of Psychiatric Research*, *137*, 126-130. https://doi.org/10.1016/j.jpsychires.2021.02.056

Matsushima, M., & Horiguchi, H. (2020). The COVID-19 Pandemic and Mental Well-Being of Pregnant Women in Japan: Need for Economic and Social Policy Interventions. *Disaster Medicine and Public Health Preparedness*, 1-6. https://doi.org/10.1017/dmp.2020.334

Mayeur, A., Binois, O., Gallot, V., Hesters, L., Benoit, A., Oppenheimer, A., . . . Sonigo, C. (2020). First follow-up of art pregnancies in the context of the COVID-19 outbreak. *European Journal of Obstetrics, Gynecology, and Reproductive Biology*, *253*, 71-75. https://doi.org/10.1016/j.ejogrb.2020.07.050

Mayopoulos, G., Ein-Dor, T., Li, K., Chan, S., & Dekel, S. (2020). Giving birth under hospital visitor restrictions: Heightened acute stress in childbirth in COVID-19 positive women. *Res Sq*. https://doi.org/10.21203/rs.3.rs-112882/v1

Mayopoulos, G. A., Ein-Dor, T., Dishy, G. A., Nandru, R., Chan, S. J., Hanley, L. E., . . . Dekel, S. (2021). COVID-19 is associated with traumatic childbirth and subsequent mother-infant bonding problems. *Journal of Affective Disorders*, *282*, 122-125. https://doi.org/10.1016/j.jad.2020.12.101

Mazza, C., Marchetti, D., Ricci, E., Fontanesi, L., Di Giandomenico, S., Verrocchio, M. C., & Roma, P. (2021). The COVID-19 lockdown and psychological distress among Italian parents: Influence of parental role, parent personality, and child difficulties. *International Journal of Psychology. Journal International de Psychologie*. https://doi.org/10.1002/ijop.12755

Mazza, C., Ricci, E., Marchetti, D., Fontanesi, L., Di Giandomenico, S., Verrocchio, M. C., & Roma, P. (2020). How Personality Relates to Distress in Parents during the Covid-19 Lockdown: The Mediating Role of Child's Emotional and Behavioral Difficulties and the Moderating Effect of Living with Other People. *International Journal of Environmental Research and Public Health*, *17*(17). https://doi.org/10.3390/ijerph17176236

McCarty, K. L., Tucker, M., Lee, G., & Pandey, V. (2021). Fetal inflammatory response syndrome associated with maternal SARS-CoV-2 infection. *Pediatrics*, *147*(4). https://doi.org/10.1542/peds.2020-010132

McDonald, H. I., Tessier, E., White, J. M., Woodruff, M., Knowles, C., Bates, C., . . . Edelstein, M. (2020). Early impact of the coronavirus disease (COVID-19) pandemic and physical distancing measures on routine childhood vaccinations in England, January to April 2020. *Eurosurveillance*, *25*(19). https://doi.org/10.2807/1560-7917.ES.2020.25.19.2000848

McFarland, M. J., McFarland, C. A. S., Hill, T. D., & D'Oria, R. (2021). Postpartum Depressive Symptoms during the Beginning of the COVID-19 Pandemic: An Examination of Population Birth Data from Central New Jersey. *Matern Child Health J*, *25*(3), 353-359. https://doi.org/10.1007/s10995-020-03116-w

Meaney, S., Leitao, S., Olander, E. K., Pope, J., & Matvienko-Sikar, K. (2021). The impact of COVID-19 on pregnant womens' experiences and perceptions of antenatal maternity care, social support, and stress-reduction strategies. *Women Birth*. https://doi.org/10.1016/j.wombi.2021.04.013

Medina-Jimenez, V., Bermudez-Rojas, M. L., Murillo-Bargas, H., Rivera-Camarillo, A. C., Muñoz-Acosta, J., Ramirez-Abarca, T. G., . . . Martinez-Portilla, R. J. (2020). The impact of the COVID-19 pandemic on depression and stress levels in pregnant women: a national survey during the COVID-19 pandemic in Mexico. *Journal of Maternal-Fetal & Neonatal Medicine*, 1-3. https://doi.org/10.1080/14767058.2020.1851675

Mehdizadehkashi, A., Chaichian, S., Haghighi, L., Eshraghi, N., Bordbar, A., Hashemi, N., . . . Tahermanesh, K. (2021). The Impact of COVID-19 Pandemic on Stress and Anxiety of Non-infected Pregnant Mothers. *J Reprod Infertil*, *22*(2), 125-132. https://doi.org/10.18502/jri.v22i2.5801

Mehta, H., Ivanovic, S., Cronin, A., VanBrunt, L., Mistry, N., Miller, R., . . . Rezai, F. (2020). Novel coronavirus-related acute respiratory distress syndrome in a patient with twin pregnancy: A case report. *Case Rep Womens Health*, *27*, e00220. https://doi.org/10.1016/j.crwh.2020.e00220

Mejía Jiménez, I., Salvador López, R., García Rosas, E., Rodriguez de la Torre, I., Montes García, J., de la Cruz Conty, M. L., & Martínez Pérez, O. (2021). Umbilical cord clamping and skin-to-skin contact in deliveries from women positive for SARS-CoV-2: a prospective observational study. *BJOG: An International Journal of Obstetrics and Gynaecology*, *128*(5), 908-915. https://doi.org/10.1111/1471-0528.16597

Mendoza-Hernández, M., Huerta-Niño de Rivera, I., Yoldi-Negrete, M., Saviñon-Tejeda, P., Franco-Cendejas, R., López-Jácome, L. E., & Navarro-Castellanos, I. (2021). Probable Case of Vertical Transmission of SARS-CoV-2 in a Newborn in Mexico. *Neonatology*, 1-4. https://doi.org/10.1159/000514710

Metz, T. D., Clifton, R. G., Hughes, B. L., Sandoval, G., Saade, G. R., Grobman, W. A., . . . Macones, G. A. (2021). Disease Severity and Perinatal Outcomes of Pregnant Patients With Coronavirus Disease 2019 (COVID-19). *Obstetrics and Gynecology*, *137*(4), 571-580. https://doi.org/10.1097/aog.0000000000004339

Milan, S., & Dáu, A. (2021). The Role of Trauma in Mothers' COVID-19 Vaccine Beliefs and Intentions. *Journal of Pediatric Psychology*. https://doi.org/10.1093/jpepsy/jsab043

Miller, J. J., Cooley, M. E., & Mihalec-Adkins, B. P. (2020). Examining the Impact of COVID-19 on Parental Stress: A Study of Foster Parents. *Child Adolesc Social Work J*, 1-10. https://doi.org/10.1007/s10560-020-00725-w

Miller, K. A., Mannix, R., Schmitz, G., Monuteaux, M. C., & Lee, L. K. (2020). Impact of COVID-19 on professional and personal responsibilities of Massachusetts physicians. *American Journal of Emergency Medicine*, *38*(11), 2365-2367. https://doi.org/10.1016/j.ajem.2020.08.051

Milne, S. J., Corbett, G. A., Hehir, M. P., Lindow, S. W., Mohan, S., Reagu, S., . . . O'Connell, M. P. (2020). Effects of isolation on mood and relationships in pregnant women during the covid-19 pandemic. *European Journal of Obstetrics, Gynecology, and Reproductive Biology*, *252*, 610-611. https://doi.org/10.1016/j.ejogrb.2020.06.009

Minckas, Medvedev, M. M., Adejuyigbe, E. A., Brotherton, H., Chellani, H., Estifanos, A. S., Ezeaka, C., Gobezayehu, A. G., Irimu, G., Kawaza, K., Kumar, V., Massawe, A., Mazumder, S., Mambule, I., Medhanyie, A. A., Molyneux, E. M., Newton, S., Salim, N., Tadele, H., … Lawn, J. E. (2021). Preterm care during the COVID-19 pandemic: A comparative risk analysis of neonatal deaths averted by kangaroo mother care versus mortality due to SARS-CoV-2 infection. EClinicalMedicine, 33, 100733–100733. <https://doi.org/10.1016/j.eclinm.2021.100733>

Mirlashari, J., Ebrahimpour, F., & Salisu, W. J. (2020). War on two fronts: Experience of children with cancer and their family during COVID-19 pandemic in Iran. *Journal of Pediatric Nursing*, *57*, 25-31. https://doi.org/10.1016/j.pedn.2020.10.024

Mirzaei, N., Jahanian Sadatmahalleh, S., Bahri Khomami, M., Moini, A., & Kazemnejad, A. (2021). Sexual function, mental health, and quality of life under strain of COVID-19 pandemic in Iranian pregnant and lactating women: a comparative cross-sectional study. *Health Qual Life Outcomes*, *19*(1), 66. https://doi.org/10.1186/s12955-021-01720-0

Mizrak Sahin, B., & Kabakci, E. N. (2020). The experiences of pregnant women during the COVID-19 pandemic in Turkey: A qualitative study. *Women Birth*. https://doi.org/10.1016/j.wombi.2020.09.022

Mo, P. K. H., Fong, V. W. I., Song, B., Di, J., Wang, Q., & Wang, L. (2021). Association of Perceived Threat, Negative Emotions, and Self-Efficacy With Mental Health and Personal Protective Behavior Among Chinese Pregnant Women During the COVID-19 Pandemic: Cross-sectional Survey Study. *Journal of Medical Internet Research*, *23*(4), e24053. https://doi.org/10.2196/24053

Moeindarbary, S., Pourhoseini, A., Layegh, P., Shahriari, Z., Fayyaz, F., Bahrami, M., & Rafiee, M. (2021). Neonates with coronavirus disease 2019 acquired from infected mothers: the incompatibility of maternal intensity and infant lung involvement: two case reports. *J Med Case Rep*, *15*(1), 310. https://doi.org/10.1186/s13256-021-02698-5

Molgora, S., & Accordini, M. (2020). Motherhood in the Time of Coronavirus: The Impact of the Pandemic Emergency on Expectant and Postpartum Women's Psychological Well-Being. *Frontiers in Psychology*, *11*, 567155. https://doi.org/10.3389/fpsyg.2020.567155

Moore, S. A., Faulkner, G., Rhodes, R. E., Brussoni, M., Chulak-Bozzer, T., Ferguson, L. J., . . . Tremblay, M. S. (2020). Impact of the COVID-19 virus outbreak on movement and play behaviours of Canadian children and youth: a national survey. *The International Journal of Behavioral Nutrition and Physical Activity*, *17*(1), 85. https://doi.org/10.1186/s12966-020-00987-8

Morelli, M., Cattelino, E., Baiocco, R., Trumello, C., Babore, A., Candelori, C., & Chirumbolo, A. (2020). Parents and Children During the COVID-19 Lockdown: The Influence of Parenting Distress and Parenting Self-Efficacy on Children's Emotional Well-Being. *Frontiers in Psychology*, *11*, 584645. https://doi.org/10.3389/fpsyg.2020.584645

Mortazavi, F., & Ghardashi, F. (2021). The lived experiences of pregnant women during COVID-19 pandemic: a descriptive phenomenological study. *BMC Pregnancy and Childbirth*, *21*(1), 193. https://doi.org/10.1186/s12884-021-03691-y

Mortazavi, F., Mehrabadi, M., & KiaeeTabar, R. (2021). Pregnant women's well-being and worry during the COVID-19 pandemic: a cross-sectional study. *BMC Pregnancy and Childbirth*, *21*(1), 59. https://doi.org/10.1186/s12884-021-03548-4

Moscardino, U., Dicataldo, R., Roch, M., Carbone, M., & Mammarella, I. C. (2021). Parental stress during COVID-19: A brief report on the role of distance education and family resources in an Italian sample. *Current Psychology (New Brunswick, N.J.)*, 1-4. https://doi.org/10.1007/s12144-021-01454-8

Mousavi, S. F. (2020). Psychological Well-Being, Marital Satisfaction, and Parental Burnout in Iranian Parents: The Effect of Home Quarantine During COVID-19 Outbreaks. *Frontiers in Psychology*, *11*, 553880. https://doi.org/10.3389/fpsyg.2020.553880

Moyer, C. A., Compton, S. D., Kaselitz, E., & Muzik, M. (2020). Pregnancy-related anxiety during COVID-19: a nationwide survey of 2740 pregnant women. *Arch Womens Ment Health*, 1-9. https://doi.org/10.1007/s00737-020-01073-5

Moyer, C. A., Sakyi, K. S., Sacks, E., Compton, S. D., Lori, J. R., & Williams, J. E. O. (2020). COVID-19 is increasing Ghanaian pregnant women's anxiety and reducing healthcare seeking. *International Journal of Gynaecology and Obstetrics*. https://doi.org/10.1002/ijgo.13487

Muhidin, S., Behboodi Moghadam, Z., & Vizheh, M. (2020). Analysis of Maternal Coronavirus Infections and Neonates Born to Mothers with 2019-nCoV; a Systematic Review. *Arch Acad Emerg Med*, *8*(1), e49.

Mulale, U. K., Kashamba, T., Strysko, J., & Kyokunda, L. T. (2021). Fatal SARS-CoV-2 and Mycobacterium tuberculosis coinfection in an infant: insights from Botswana. *BMJ Case Reports*, *14*(4). https://doi.org/10.1136/bcr-2020-239701

Muldoon, K. A., Denize, K. M., Talarico, R., Boisvert, C., Frank, O., Harvey, A. L. J., . . . El-Chaar, D. (2021). COVID-19 and perinatal intimate partner violence: a cross-sectional survey of pregnant and postpartum individuals in the early stages of the COVID-19 pandemic. *BMJ Open*, *11*(5), e049295. https://doi.org/10.1136/bmjopen-2021-049295

Mullins, E., Hudak, M. L., Banerjee, J., Getzlaff, T., Townson, J., Barnette, K., . . . Lees, C. C. (2021). Pregnancy and neonatal outcomes of COVID-19: coreporting of common outcomes from PAN-COVID and AAP-SONPM registries. *Ultrasound in Obstetrics and Gynecology*, *57*(4), 573-581. https://doi.org/10.1002/uog.23619

Mumbardó-Adam, C., Barnet-López, S., & Balboni, G. (2021). How have youth with Autism Spectrum Disorder managed quarantine derived from COVID-19 pandemic? An approach to families perspectives. *Research in Developmental Disabilities*, *110*, 103860. https://doi.org/10.1016/j.ridd.2021.103860

Muniraman, H., Ali, M., Cawley, P., Hillyer, J., Heathcote, A., Ponnusamy, V., . . . Clarke, P. (2020). Parental perceptions of the impact of neonatal unit visitation policies during COVID-19 pandemic. *BMJ Paediatrics Open*, *4*(1). https://doi.org/10.1136/bmjpo-2020-000899

Myers, S., & Emmott, E. H. (2021). Communication Across Maternal Social Networks During England's First National Lockdown and Its Association With Postnatal Depressive Symptoms. *Frontiers in Psychology*, *12*, 648002. https://doi.org/10.3389/fpsyg.2021.648002

Naghizadeh, S., Mirghafourvand, M., & Mohammadirad, R. (2021). Domestic violence and its relationship with quality of life in pregnant women during the outbreak of COVID-19 disease. *BMC Pregnancy and Childbirth*, *21*(1), 88. https://doi.org/10.1186/s12884-021-03579-x

Nakstad, B., Kaang, T., Gezmu, A. M., & Strysko, J. (2021). Nosocomial SARS-CoV-2 transmission in a neonatal unit in Botswana: chronic overcrowding meets a novel pathogen. *BMJ Case Reports*, *14*(6). https://doi.org/10.1136/bcr-2021-242421

Nanjundaswamy, M. H., Shiva, L., Desai, G., Ganjekar, S., Kishore, T., Ram, U., . . . Chandra, P. S. (2020). COVID-19-related anxiety and concerns expressed by pregnant and postpartum women-a survey among obstetricians. *Arch Womens Ment Health*, 1-4. https://doi.org/10.1007/s00737-020-01060-w

Nassar, M. F., Allam, M. F., & Shata, M. O. (2021). Effect of COVID-19 Lockdown on Young Egyptian Soccer Players. *Glob Pediatr Health*, *8*, 2333794x211012980. https://doi.org/10.1177/2333794x211012980

Nastro, F. F., Tolone, C., Serra, M. R., Pacella, D., Campanozzi, A., & Strisciuglio, C. (2020). Prevalence of functional gastrointestinal disorders in children with celiac disease during the COVID-19 lockdown. *Digestive and Liver Disease*, *52*(10), 1082-1084. https://doi.org/10.1016/j.dld.2020.06.030

Naurin, E., Markstedt, E., Stolle, D., Enström, D., Wallin, A., Andreasson, I., . . . Sengpiel, V. (2020). Pregnant under the pressure of a pandemic: a large-scale longitudinal survey before and during the COVID-19 outbreak. *European Journal of Public Health*. https://doi.org/10.1093/eurpub/ckaa223

Neece, C., McIntyre, L. L., & Fenning, R. (2020). Examining the impact of COVID-19 in ethnically diverse families with young children with intellectual and developmental disabilities. *Journal of Intellectual Disability Research*, *64*(10), 739-749. https://doi.org/10.1111/jir.12769

Neubauer, A. B., Schmidt, A., Kramer, A. C., & Schmiedek, F. (2021). A Little Autonomy Support Goes a Long Way: Daily Autonomy-Supportive Parenting, Child Well-Being, Parental Need Fulfillment, and Change in Child, Family, and Parent Adjustment Across the Adaptation to the COVID-19 Pandemic. *Child Development*. https://doi.org/10.1111/cdev.13515

Ng, Q. J., Koh, K. M., Tagore, S., & Mathur, M. (2020). Perception and Feelings of Antenatal Women during COVID-19 Pandemic: A Cross-Sectional Survey. *Ann Acad Med Singap*, *49*(8), 543-552.

Nguyen, P. H., Kachwaha, S., Pant, A., Tran, L. M., Ghosh, S., Sharma, P. K., . . . Menon, P. (2021). Impact of COVID-19 on household food insecurity and interlinkages with child feeding practices and coping strategies in Uttar Pradesh, India: a longitudinal community-based study. *BMJ Open*, *11*(4), e048738. https://doi.org/10.1136/bmjopen-2021-048738

Nicholson, E., McDonnell, T., Conlon, C., Barrett, M., Cummins, F., Hensey, C., & McAuliffe, E. (2020). Parental hesitancy and concerns around accessing paediatric unscheduled healthcare during covid-19: A cross-sectional survey. *International Journal of Environmental Research and Public Health*, *17*(24), 1-19. https://doi.org/10.3390/ijerph17249264

Niela-Vilén, H., Auxier, J., Ekholm, E., Sarhaddi, F., Asgari Mehrabadi, M., Mahmoudzadeh, A., . . . Axelin, A. (2021). Pregnant women's daily patterns of well-being before and during the COVID-19 pandemic in Finland: Longitudinal monitoring through smartwatch technology. *PloS One*, *16*(2), e0246494. https://doi.org/10.1371/journal.pone.0246494

Nodoushan, R. J., Alimoradi, H., & Nazari, M. (2020). Spiritual Health and Stress in Pregnant Women During the Covid-19 Pandemic. *SN Compr Clin Med*, 1-7. https://doi.org/10.1007/s42399-020-00582-9

Nomura, R., Tavares, I., Ubinha, A. C., Costa, M. L., Opperman, M. L., Brock, M., . . . Br, A. P. S. C. B. A. D. P. S. G. I. C. (2021). Impact of the COVID-19 Pandemic on Maternal Anxiety in Brazil. *J Clin Med*, *10*(4). https://doi.org/10.3390/jcm10040620

Norman, M., Navér, L., Söderling, J., Ahlberg, M., Hervius Askling, H., Aronsson, B., . . . Stephansson, O. (2021). Association of Maternal SARS-CoV-2 Infection in Pregnancy With Neonatal Outcomes. *JAMA*, *325*(20), 2076-2086. https://doi.org/10.1001/jama.2021.5775

Nurrizka, R. H., Nurdiantami, Y., & Makkiyah, F. A. (2021). Psychological outcomes of the COVID-19 pandemic among pregnant women in Indonesia: a cross-sectional study. *Osong Public Health Res Perspect*, *12*(2), 80-87. https://doi.org/10.24171/j.phrp.2021.12.2.05

Odeh, R., Gharaibeh, L., Daher, A., Kussad, S., & Alassaf, A. (2020). Caring for a child with type 1 diabetes during COVID-19 lockdown in a developing country: Challenges and parents' perspectives on the use of telemedicine. *Diabetes Research and Clinical Practice*, *168*, 108393. https://doi.org/10.1016/j.diabres.2020.108393

O'Driscoll, Ribeiro Dos Santos, G., Wang, L., Cummings, D. A. T., Azman, A. S., Paireau, J., Fontanet, A., Cauchemez, S., & Salje, H. (2020). Age-specific mortality and immunity patterns of SARS-CoV-2. Nature (London), 590(7844), 140–145. <https://doi.org/10.1038/s41586-020-2918-0>

Ogamba, I., Kliss, A., Rainville, N., Chuang, L., Panarelli, E., Petrini, J., & Zilberman, D. (2021). Initial review of pregnancy and neonatal outcomes of pregnant women with COVID-19 infection. *Journal of Perinatal Medicine*, *49*(3), 263-268. https://doi.org/10.1515/jpm-2020-0446

Ollivier, R., Aston, D. M., Price, D. S., Sim, D. M., Benoit, D. B., Joy, D. P., . . . Nassaji, N. A. (2021). Mental Health & Parental Concerns during COVID-19: The Experiences of New Mothers Amidst Social Isolation. *Midwifery*, *94*, 102902. https://doi.org/10.1016/j.midw.2020.102902

Oncel, M. Y., Akın, I. M., Kanburoglu, M. K., Tayman, C., Coskun, S., Narter, F., . . . Koc, E. (2020). A multicenter study on epidemiological and clinical characteristics of 125 newborns born to women infected with COVID-19 by Turkish Neonatal Society. *European Journal of Pediatrics*, 1-10. https://doi.org/10.1007/s00431-020-03767-5

Orsini, A., Corsi, M., Pedrinelli, V., Santangelo, A., Bertelloni, C., Dell'Oste, V., . . . Carmassi, C. (2021). Post-traumatic stress, anxiety, and depressive symptoms in caregivers of children tested for COVID-19 in the acute phase of the Italian outbreak. *Journal of Psychiatric Research*, *135*, 256-263. https://doi.org/10.1016/j.jpsychires.2021.01.024

Oskovi-Kaplan, Z. A., Buyuk, G. N., Ozgu-Erdinc, A. S., Keskin, H. L., Ozbas, A., & Moraloglu Tekin, O. (2020). The Effect of COVID-19 Pandemic and Social Restrictions on Depression Rates and Maternal Attachment in Immediate Postpartum Women: a Preliminary Study. *Psychiatric Quarterly*, 1-8. https://doi.org/10.1007/s11126-020-09843-1

Ostacoli, L., Cosma, S., Bevilacqua, F., Berchialla, P., Bovetti, M., Carosso, A. R., . . . Benedetto, C. (2020). Psychosocial factors associated with postpartum psychological distress during the Covid-19 pandemic: a cross-sectional study. *BMC Pregnancy and Childbirth*, *20*(1), 703. https://doi.org/10.1186/s12884-020-03399-5

Overall, N. C., Chang, V. T., Cross, E. J., Low, R. S. T., & Henderson, A. M. E. (2021). Sexist attitudes predict family-based aggression during a COVID-19 lockdown. *Journal of Family Psychology*. https://doi.org/10.1037/fam0000834

Overbeck, G., Graungaard, A. H., Rasmussen, I. S., Andersen, J. H., Ertmann, R. K., Kragstrup, J., & Wilson, P. (2020). Pregnant women's concerns and antenatal care during COVID-19 lock-down of the Danish society. *Danish Medical Journal*, *67*(12).

Overbeck, G., Rasmussen, I. S., Siersma, V., Andersen, J. H., Kragstrup, J., Wilson, P., . . . Ertmann, R. K. (2021). Depression and anxiety symptoms in pregnant women in Denmark during COVID-19. *Scand J Public Health*, 14034948211013271. https://doi.org/10.1177/14034948211013271

Ozturk Eyimaya, A., & Yalçin Irmak, A. (2020). Relationship between parenting practices and children's screen time during the COVID-19 Pandemic in Turkey. *Journal of Pediatric Nursing*, *56*, 24-29. https://doi.org/10.1016/j.pedn.2020.10.002

Pacheco, F., Sobral, M., Guiomar, R., de la Torre-Luque, A., Caparros-Gonzalez, R. A., & Ganho-Ávila, A. (2021). Breastfeeding during COVID-19: A Narrative Review of the Psychological Impact on Mothers. *Behav Sci (Basel)*, *11*(3). https://doi.org/10.3390/bs11030034

Papapanou, M., Papaioannou, M., Petta, A., Routsi, E., Farmaki, M., Vlahos, N., & Siristatidis, C. (2021). Maternal and Neonatal Characteristics and Outcomes of COVID-19 in Pregnancy: An Overview of Systematic Reviews. *International Journal of Environmental Research and Public Health*, *18*(2). https://doi.org/10.3390/ijerph18020596

Paramanathan, S., Kyng, K. J., Laursen, A. L., Jensen, L. D., Grejs, A. M., & Jain, D. (2021). COVID-19 with severe acute respiratory distress in a pregnant woman leading to preterm caesarean section: A case report. *Case Rep Womens Health*, *30*, e00304. https://doi.org/10.1016/j.crwh.2021.e00304

Pariente, G., Wissotzky Broder, O., Sheiner, E., Lanxner Battat, T., Mazor, E., Yaniv Salem, S., . . . Wainstock, T. (2020). Risk for probable post-partum depression among women during the COVID-19 pandemic. *Arch Womens Ment Health*, 1-7. https://doi.org/10.1007/s00737-020-01075-3

Parra-Saavedra, M., Villa-Villa, I., Pérez-Olivo, J., Guzman-Polania, L., Galvis-Centurion, P., Cumplido-Romero, Á., . . . Miranda, J. (2020). Attitudes and collateral psychological effects of COVID-19 in pregnant women in Colombia. *International Journal of Gynaecology and Obstetrics*, *151*(2), 203-208. https://doi.org/10.1002/ijgo.13348

Pasca, L., Zanaboni, M. P., Grumi, S., Totaro, M., Ballante, E., Varesio, C., & De Giorgis, V. (2021). Impact of COVID-19 pandemic in pediatric patients with epilepsy with neuropsychiatric comorbidities: A telemedicine evaluation. *Epilepsy & Behavior*, *115*, 107519. https://doi.org/10.1016/j.yebeh.2020.107519

Paschke, K., Arnaud, N., Austermann, M. I., & Thomasius, R. (2021). Risk factors for prospective increase in psychological stress during COVID-19 lockdown in a representative sample of adolescents and their parents. *BJPsych Open*, *7*(3), e94. https://doi.org/10.1192/bjo.2021.49

Patabendige, M., Gamage, M. M., Weerasinghe, M., & Jayawardane, A. (2020). Psychological impact of the COVID-19 pandemic among pregnant women in Sri Lanka. *International Journal of Gynaecology and Obstetrics*, *151*(1), 150-153. https://doi.org/10.1002/ijgo.13335

Patel, V. Saxena, S., Lund, C., Thornicroft, G., Baingana, F., Bolton, P., Chisholm, D., Collins, P. Y., Cooper, J. L., Eaton, J., Herrman, H., Herzallah, M. M., Huang, Y., Jordans, M. J. D., Kleinman, A., Medina-Mora, M. E., Morgan, E., Niaz, U., Omigbodun, UnÜtzer, J. (2018). The Lancet Commission on global mental health and sustainable development. The Lancet (British Edition), 392(10157), 1553–1598. https://doi.org/10.1016/S0140-6736(18)31612-X

Patrick, S. W., Henkhaus, L. E., Zickafoose, J. S., Lovell, K., Halvorson, A., Loch, S., . . . Davis, M. M. (2020). Well-being of Parents and Children During the COVID-19 Pandemic: A National Survey. *Pediatrics*, *146*(4). https://doi.org/10.1542/peds.2020-016824

Pearson, C., Levine, M., Messman, A., Chopra, T., Awali, R., Robb, L., . . . Levine, D. L. (2021). Understanding the Impact of COVID-19 on Physician Moms. *Disaster Medicine and Public Health Preparedness*, 1-17. https://doi.org/10.1017/dmp.2021.49

Peltz, J. S., Daks, J. S., & Rogge, R. D. (2020). Mediators of the association between COVID-19-related stressors and parents' psychological flexibility and inflexibility: The roles of perceived sleep quality and energy. *J Contextual Behav Sci*, *17*, 168-176. https://doi.org/10.1016/j.jcbs.2020.07.001

Peng, S., Zhang, Y., Liu, H., Huang, X., Noble, D. J., Yang, L., . . . Narayan, A. (2021). A multi-center survey on the postpartum mental health of mothers and attachment to their neonates during COVID-19 in Hubei Province of China. *Ann Transl Med*, *9*(5), 382. https://doi.org/10.21037/atm-20-6115

Peng, S., Zhu, H., Yang, L., Cao, L., Huang, X., Dynes, M., . . . Xia, S. (2020). A study of breastfeeding practices, SARS-CoV-2 and its antibodies in the breast milk of mothers confirmed with COVID-19. *Lancet Reg Health West Pac*, *4*, 100045. https://doi.org/10.1016/j.lanwpc.2020.100045

Perez, A., Panagiotopoulou, E., Curtis, P., & Roberts, R. (2021). Barriers and facilitators to mood and confidence in pregnancy and early parenthood during COVID-19 in the UK: mixed-methods synthesis survey. *BJPsych Open*, *7*(4), e107. https://doi.org/10.1192/bjo.2021.925

Perlman, J., Oxford, C., Chang, C., Salvatore, C., & Pace, J. D. (2020). Delivery Room Preparedness and Early Neonatal Outcomes during COVID-19 Pandemic in New York City. *Pediatrics*, *146*(2). https://doi.org/10.1542/peds.2020-1567

Perzow, S. E. D., Hennessey, E. P., Hoffman, M. C., Grote, N. K., Davis, E. P., & Hankin, B. L. (2021). Mental health of pregnant and postpartum women in response to the COVID-19 pandemic. *J Affect Disord Rep*, *4*, 100123. https://doi.org/10.1016/j.jadr.2021.100123

Petrocchi, S., Levante, A., Bianco, F., Castelli, I., & Lecciso, F. (2020). Maternal Distress/Coping and Children's Adaptive Behaviors During the COVID-19 Lockdown: Mediation Through Children's Emotional Experience. *Front Public Health*, *8*, 587833. https://doi.org/10.3389/fpubh.2020.587833

Philippe, K., Chabanet, C., Issanchou, S., & Monnery-Patris, S. (2021). Child eating behaviors, parental feeding practices and food shopping motivations during the COVID-19 lockdown in France: (How) did they change? *Appetite*, *161*, 105132. https://doi.org/10.1016/j.appet.2021.105132

Pierce-Williams, R. A. M., Burd, J., Felder, L., Khoury, R., Bernstein, P. S., Avila, K., . . . Berghella, V. (2020). Clinical course of severe and critical coronavirus disease 2019 in hospitalized pregnancies: a United States cohort study. *Am J Obstet Gynecol MFM*, *2*(3), 100134. https://doi.org/10.1016/j.ajogmf.2020.100134

Piersigilli, F., Carkeek, K., Hocq, C., van Grambezen, B., Hubinont, C., Chatzis, O., . . . Danhaive, O. (2020). COVID-19 in a 26-week preterm neonate. *The Lancet Child and Adolescent Health*, *4*(6), 476-478. https://doi.org/10.1016/S2352-4642(20)30140-1

Pınar Senkalfa, B., Sismanlar Eyuboglu, T., Aslan, A. T., Ramaslı Gursoy, T., Soysal, A. S., Yapar, D., & İlhan, M. N. (2020). Effect of the COVID-19 pandemic on anxiety among children with cystic fibrosis and their mothers. *Pediatric Pulmonology*, *55*(8), 2128-2134. https://doi.org/10.1002/ppul.24900

Pope, J., Olander, E. K., Leitao, S., Meaney, S., & Matvienko-Sikar, K. (2021). Prenatal stress, health, and health behaviours during the COVID-19 pandemic: An international survey. *Women Birth*. https://doi.org/10.1016/j.wombi.2021.03.007

Popofsky, S., Noor, A., Leavens-Maurer, J., Quintos-Alagheband, M. L., Mock, A., Vinci, A., . . . Krilov, L. (2020). Impact of Maternal Severe Acute Respiratory Syndrome Coronavirus 2 Detection on Breastfeeding Due to Infant Separation at Birth. *Journal of Pediatrics*, *226*, 64-70. https://doi.org/10.1016/j.jpeds.2020.08.004

Poulain, T., Meigen, C., Sobek, C., Ober, P., Igel, U., Körner, A., . . . Vogel, M. (2021). Loss of childcare and classroom teaching during the Covid-19-related lockdown in spring 2020: A longitudinal study on consequences on leisure behavior and schoolwork at home. *PloS One*, *16*(3), e0247949. https://doi.org/10.1371/journal.pone.0247949

Preis, H., Mahaffey, B., Heiselman, C., & Lobel, M. (2020a). Pandemic-related pregnancy stress and anxiety among women pregnant during the coronavirus disease 2019 pandemic. *Am J Obstet Gynecol MFM*, *2*(3), 100155. https://doi.org/10.1016/j.ajogmf.2020.100155

Preis, H., Mahaffey, B., Heiselman, C., & Lobel, M. (2020b). Vulnerability and resilience to pandemic-related stress among U.S. women pregnant at the start of the COVID-19 pandemic. *Social Science and Medicine*, *266*, 113348. https://doi.org/10.1016/j.socscimed.2020.113348

Preis, H., Mahaffey, B., & Lobel, M. (2020). Psychometric properties of the Pandemic-Related Pregnancy Stress Scale (PREPS). *Journal of Psychosomatic Obstetrics and Gynaecology*, *41*(3), 191-197. https://doi.org/10.1080/0167482x.2020.1801625

Preis, H., Mahaffey, B., & Lobel, M. (2021). The role of pandemic-related pregnancy stress in preference for community birth during the beginning of the COVID-19 pandemic in the United States. *Birth*, *48*(2), 242-250. https://doi.org/10.1111/birt.12533

Preis, H., Mahaffey, B., Pati, S., Heiselman, C., & Lobel, M. (2021). Adverse Perinatal Outcomes Predicted by Prenatal Maternal Stress Among U.S. Women at the COVID-19 Pandemic Onset. *Annals of Behavioral Medicine*, *55*(3), 179-191. https://doi.org/10.1093/abm/kaab005

Preuss, H., Capito, K., van Eickels, R. L., Zemp, M., & Kolar, D. R. (2021). Cognitive reappraisal and self-compassion as emotion regulation strategies for parents during COVID-19: An online randomized controlled trial. *Internet Interv*, *24*, 100388. https://doi.org/10.1016/j.invent.2021.100388

Prikhidko, A., Long, H., & Wheaton, M. G. (2020). The Effect of Concerns About COVID-19 on Anxiety, Stress, Parental Burnout, and Emotion Regulation: The Role of Susceptibility to Digital Emotion Contagion. *Front Public Health*, *8*, 567250. https://doi.org/10.3389/fpubh.2020.567250

Protudjer, J. L. P., Golding, M., Salisbury, M. R., Abrams, E. M., & Roos, L. E. (2020). High anxiety and health-related quality of life in families with children with food allergy during coronavirus disease 2019. *Annals of Allergy, Asthma, and Immunology*, *126*(1), 83-88.e81. https://doi.org/10.1016/j.anai.2020.09.010

Puertas-Gonzalez, J. A., Mariño-Narvaez, C., Peralta-Ramirez, M. I., & Romero-Gonzalez, B. (2021). The psychological impact of the COVID-19 pandemic on pregnant women. *Psychiatry Research*, *301*, 113978. https://doi.org/10.1016/j.psychres.2021.113978

Qi, M., Li, X., Liu, S., Li, Y., & Huang, W. (2020). Impact of the COVID-19 epidemic on patterns of pregnant women's perception of threat and its relationship to mental state: A latent class analysis. *PloS One*, *15*(10), e0239697. https://doi.org/10.1371/journal.pone.0239697

Quandt, S. A., LaMonto, N. J., Mora, D. C., Talton, J. W., Laurienti, P. J., & Arcury, T. A. (2020). COVID-19 Pandemic Among Immigrant Latinx Farmworker and Non-farmworker Families: A Rural-Urban Comparison of Economic, Educational, Healthcare, and Immigration Concerns. *medRxiv*. https://doi.org/10.1101/2020.10.30.20223156

Quenzer-Alfred, C., Schneider, L., Soyka, V., Harbrecht, M., Blume, V., & Mays, D. (2021). No nursery ‘til school–the transition to primary school without institutional transition support due to the COVID-19 shutdown in Germany. *European Journal of Special Needs Education*, *36*(1), 127-141. https://doi.org/10.1080/08856257.2021.1872850

Quílez-Robres, A., Lozano-Blasco, R., Íñiguez-Berrozpe, T., & Cortés-Pascual, A. (2021). Social, Family, and Educational Impacts on Anxiety and Cognitive Empathy Derived From the COVID-19: Study on Families With Children. *Frontiers in Psychology*, *12*, 562800. https://doi.org/10.3389/fpsyg.2021.562800

Racine, N., Hetherington, E., McArthur, B. A., McDonald, S., Edwards, S., Tough, S., & Madigan, S. (2021). Maternal depressive and anxiety symptoms before and during the COVID-19 pandemic in Canada: a longitudinal analysis. *Lancet Psychiatry*, *8*(5), 405-415. https://doi.org/10.1016/s2215-0366(21)00074-2

Rao, Minckas, N., Medvedev, M. M., Gathara, D., Y N, P., Seifu Estifanos, A., Silitonga, A. C., Jadaun, A. S., Adejuyigbe, E. A., Brotherton, H., Arya, S., Gera, R., Ezeaka, C. V., Gai, A., Gobezayehu, A. G., Dube, Q., Kumar, A., Naburi, H., Chiume, M., … Lawn, J. E. (2021). Small and sick newborn care during the COVID-19 pandemic: global survey and thematic analysis of healthcare providers’ voices and experiences. BMJ Global Health, 6(3), e004347. <https://doi.org/10.1136/bmjgh-2020-004347>

Ravaldi, C., Ricca, V., Wilson, A., Homer, C., & Vannacci, A. (2020). Previous psychopathology predicted severe COVID-19 concern, anxiety, and PTSD symptoms in pregnant women during "lockdown" in Italy. *Arch Womens Ment Health*, 1-4. https://doi.org/10.1007/s00737-020-01086-0

Ravaldi, C., Wilson, A., Ricca, V., Homer, C., & Vannacci, A. (2020). Pregnant women voice their concerns and birth expectations during the COVID-19 pandemic in Italy. *Women Birth*. https://doi.org/10.1016/j.wombi.2020.07.002

Ravens-Sieberer, U., Kaman, A., Otto, C., Adedeji, A., Devine, J., Erhart, M., . . . Hurrelmann, K. (2020). Mental health and quality of life in children and adolescents during the COVID-19 pandemic—results of the copsy study. *Deutsches Arzteblatt International*, *117*, 828-829. https://doi.org/10.3238/arztebl.2020.0828

Raviv, T., Warren, C. M., Washburn, J. J., Kanaley, M. K., Eihentale, L., Goldenthal, H. J., . . . Gupta, R. (2021). Caregiver Perceptions of Children's Psychological Well-being During the COVID-19 Pandemic. *JAMA Netw Open*, *4*(4), e2111103. https://doi.org/10.1001/jamanetworkopen.2021.11103

Recto, P., & Lesser, J. (2020). Young Hispanic fathers during COVID-19: Balancing parenthood, finding strength, and maintaining hope. *Public Health Nursing*. https://doi.org/10.1111/phn.12857

Reis, O., Steigmiller, L., Spitzer, C., Kölch, M., & Knabe, A. (2021). [Coping in families with members suffering from a mental disorder]. *Psychotherapeut (Berl)*, 1-7. https://doi.org/10.1007/s00278-021-00492-8

Ren, J., Li, X., Chen, S., & Nie, Y. (2020). The Influence of Factors Such as Parenting Stress and Social Support on the State Anxiety in Parents of Special Needs Children During the COVID-19 Epidemic. *Frontiers in Psychology*, *11*, 565393. https://doi.org/10.3389/fpsyg.2020.565393

Rhodes, A., Kheireddine, S., & Smith, A. D. (2020). A mixed methods investigation into the experiences, attitudes and needs of Baby Buddy pregnancy and parenting app users during the COVID-19 pandemic. *JMIR Mhealth Uhealth*. https://doi.org/10.2196/23157

Ribeiro, F. S., Braun Janzen, T., Passarini, L., & Vanzella, P. (2021). Exploring Changes in Musical Behaviors of Caregivers and Children in Social Distancing During the COVID-19 Outbreak. *Frontiers in Psychology*, *12*, 633499. https://doi.org/10.3389/fpsyg.2021.633499

Rice, K., & Williams, S. (2021). Women's postpartum experiences in Canada during the COVID-19 pandemic: a qualitative study. *CMAJ Open*, *9*(2), E556-e562. https://doi.org/10.9778/cmajo.20210008

Rivera-Hernandez, P., Nair, J., Islam, S., Davidson, L., Chang, A., & Elberson, V. (2020). Coronavirus Disease 2019 in a Premature Infant: Vertical Transmission and Antibody Response or Lack Thereof. *AJP Reports*, *10*(3), E224-E227. https://doi.org/10.1055/s-0040-1715176

Rocha, H. A., Sudfeld, C. R., Leite Á, J., Rocha, S. G., Machado, M. M., Campos, J. S., . . . Correia, L. L. (2021). Coronavirus disease 2019, food security and maternal mental health in Ceará, Brazil: a repeated cross-sectional survey. *Public Health Nutrition*, *24*(7), 1836-1840. https://doi.org/10.1017/s1368980021000628

Rodriguez, C. M., Lee, S. J., Ward, K. P., & Pu, D. F. (2021). The Perfect Storm: Hidden Risk of Child Maltreatment During the Covid-19 Pandemic. *Child Maltreat*, *26*(2), 139-151. https://doi.org/10.1177/1077559520982066

Rogers, G., Perez-Olivas, G., Stenfert Kroese, B., Patel, V., Murphy, G., Rose, J., . . . Willner, P. (2021). The experiences of mothers of children and young people with intellectual disabilities during the first COVID-19 lockdown period. *J Appl Res Intellect Disabil*. https://doi.org/10.1111/jar.12884

Romero, E., López-Romero, L., Domínguez-Álvarez, B., Villar, P., & Gómez-Fraguela, J. A. (2020). Testing the Effects of COVID-19 Confinement in Spanish Children: The Role of Parents' Distress, Emotional Problems and Specific Parenting. *International Journal of Environmental Research and Public Health*, *17*(19). https://doi.org/10.3390/ijerph17196975

Romero-Gonzalez, B., Puertas-Gonzalez, J. A., Mariño-Narvaez, C., & Peralta-Ramirez, M. I. (2020). Confinement variables by COVID-19 predictors of anxious and depressive symptoms in pregnant women. *Medicina Clínica*. https://doi.org/10.1016/j.medcli.2020.10.002

Ronchi, A., Pietrasanta, C., Zavattoni, M., Saruggia, M., Schena, F., Sinelli, M. T., . . . Pugni, L. (2021). Evaluation of Rooming-in Practice for Neonates Born to Mothers With Severe Acute Respiratory Syndrome Coronavirus 2 Infection in Italy. *JAMA Pediatr*, *175*(3), 260-266. https://doi.org/10.1001/jamapediatrics.2020.5086

Roos, L. E., Salisbury, M., Penner-Goeke, L., Cameron, E. E., Protudjer, J. L. P., Giuliano, R., . . . Reynolds, K. (2021). Supporting families to protect child health: Parenting quality and household needs during the COVID-19 pandemic. *PloS One*, *16*(5), e0251720. https://doi.org/10.1371/journal.pone.0251720

Rosen, H., Bart, Y., Zlatkin, R., Ben-Sira, L., Bashat, D. B., Amit, S., . . . Yinon, Y. (2021). Fetal and perinatal outcome following first and second trimester covid-19 infection: Evidence from a prospective cohort study. *Journal of Clinical Medicine*, *10*(10). https://doi.org/10.3390/jcm10102152

Rudrum, S. (2021). Pregnancy During the Global COVID-19 Pandemic: Canadian Experiences of Care. *Front Sociol*, *6*, 611324. https://doi.org/10.3389/fsoc.2021.611324

Russell, B. S., Hutchison, M., Tambling, R., Tomkunas, A. J., & Horton, A. L. (2020). Initial Challenges of Caregiving During COVID-19: Caregiver Burden, Mental Health, and the Parent-Child Relationship. *Child Psychiatry and Human Development*, *51*(5), 671-682. https://doi.org/10.1007/s10578-020-01037-x

Saadati, N., Afshari, P., Boostani, H., Beheshtinasab, M., Abedi, P., & Maraghi, E. (2021). Health anxiety and related factors among pregnant women during the COVID-19 pandemic: a cross-sectional study from Iran. *BMC Psychiatry*, *21*(1), 95. https://doi.org/10.1186/s12888-021-03092-7

Sabharwal, V., Bartolome, R., Hassan, S. A., Levesque, B. M., Camelo, I. Y., Wachman, E. M., . . . Parker, M. G. (2021). Mother-Infant Dyads with COVID-19 at an Urban, Safety-Net Hospital: Clinical Manifestations and Birth Outcomes. *American Journal of Perinatology*, *38*(7), 741-746. https://doi.org/10.1055/s-0041-1726429

Sade, S., Sheiner, E., Wainstock, T., Hermon, N., Yaniv Salem, S., Kosef, T., . . . Pariente, G. (2020). Risk for Depressive Symptoms among Hospitalized Women in High-Risk Pregnancy Units during the COVID-19 Pandemic. *J Clin Med*, *9*(8). https://doi.org/10.3390/jcm9082449

Sahin, B., & Ozturk, D. M. (2021). Evaluation of the level of anxiety among pregnant women during the outbreak of Covid-19. *Journal of Experimental and Clinical Medicine (Turkey)*, *38*(2), 143-149. https://doi.org/10.52142/omujecm.38.2.16

Sahithya, B. R., Kashyap, R. S., & Roopesh, B. N. (2020). Perceived stress, parental stress, and parenting during covid-19 lockdown: A preliminary study. *Journal of Indian Association for Child and Adolescent Mental Health*, *16*(4), 44-63.

Sakalidis, V. S., Rea, A., Perrella, S. L., McEachran, J., Collis, G., Miraudo, J., . . . Geddes, D. T. (2021). Wellbeing of Breastfeeding Women in Australia and New Zealand during the COVID-19 Pandemic: A Cross-Sectional Study. *Nutrients*, *13*(6). https://doi.org/10.3390/nu13061831

Salehi, L., Rahimzadeh, M., Molaei, E., Zaheri, H., & Esmaelzadeh-Saeieh, S. (2020). The relationship among fear and anxiety of COVID-19, pregnancy experience, and mental health disorder in pregnant women: A structural equation model. *Brain Behav*, *10*(11), e01835. https://doi.org/10.1002/brb3.1835

Salik, I., & Mehta, B. (2020). Tetralogy of Fallot palliation in a COVID-19 positive neonate. *Journal of Clinical Anesthesia*, *66*, 109914. https://doi.org/10.1016/j.jclinane.2020.109914

Salmi, H., Heinonen, S., Hästbacka, J., Lääperi, M., Rautiainen, P., Miettinen, P. J., . . . Knip, M. (2021). New-onset type 1 diabetes in Finnish children during the COVID-19 pandemic. *Archives of Disease in Childhood*. https://doi.org/10.1136/archdischild-2020-321220

Salvatori, G., De Rose, D. U., Concato, C., Alario, D., Olivini, N., Dotta, A., & Campana, A. (2020). Managing COVID-19-Positive Maternal-Infant Dyads: An Italian Experience. *Breastfeeding Medicine*, *15*(5), 347-348. https://doi.org/10.1089/bfm.2020.0095

Sama, B. K., Kaur, P., Thind, P. S., Verma, M. K., Kaur, M., & Singh, D. D. (2020). Implications of COVID-19-induced nationwide lockdown on children's behaviour in Punjab, India. *Child: Care, Health and Development*. https://doi.org/10.1111/cch.12816

Sbrilli, M. D., Haigler, K., & Laurent, H. K. (2021). The Indirect Effect of Parental Intolerance of Uncertainty on Perinatal Mental Health via Mindfulness During COVID-19. *Mindfulness (N Y)*, 1-10. https://doi.org/10.1007/s12671-021-01657-x

Scala, M., Marchman, V. A., Brignoni-Pérez, E., Morales, M. C., & Travis, K. E. (2020). Impact of the COVID-19 pandemic on developmental care practices for infants born preterm. *medRxiv*. https://doi.org/10.1101/2020.11.25.20238956

Scarpellini, F., Segre, G., Cartabia, M., Zanetti, M., Campi, R., Clavenna, A., & Bonati, M. (2021). Distance learning in Italian primary and middle school children during the COVID-19 pandemic: a national survey. *BMC Public Health*, *21*(1), 1035. https://doi.org/10.1186/s12889-021-11026-x

Schoenmakers, S., Snijder, P., Verdijk, R. M., Kuiken, T., Kamphuis, S. S. M., Koopman, L. P., . . . Reiss, I. K. M. (2021). Severe Acute Respiratory Syndrome Coronavirus 2 Placental Infection and Inflammation Leading to Fetal Distress and Neonatal Multi-Organ Failure in an Asymptomatic Woman. *J Pediatric Infect Dis Soc*, *10*(5), 556-561. https://doi.org/10.1093/jpids/piaa153

Schwartz, D. A. (2020). An Analysis of 38 Pregnant Women with COVID-19, Their Newborn Infants, and Maternal-Fetal Transmission of SARS-CoV-2: Maternal Coronavirus Infections and Pregnancy Outcomes. *Archives of Pathology and Laboratory Medicine*. https://doi.org/10.5858/arpa.2020-0901-SA

Schwartz, D. A., Baldewijns, M., Benachi, A., Bugatti, M., Collins, R. R. J., De Luca, D., . . . Vivanti, A. J. (2021). Chronic Histiocytic Intervillositis With Trophoblast Necrosis Is a Risk Factor Associated With Placental Infection From Coronavirus Disease 2019 (COVID-19) and Intrauterine Maternal-Fetal Severe Acute Respiratory Syndrome Coronavirus 2 (SARS-CoV-2) Transmission in Live-Born and Stillborn Infants. *Archives of Pathology and Laboratory Medicine*, *145*(5), 517-528. https://doi.org/10.5858/arpa.2020-0771-SA

Shafer, K., Scheibling, C., & Milkie, M. A. (2020). The Division of Domestic Labor before and during the COVID-19 Pandemic in Canada: Stagnation versus Shifts in Fathers' Contributions. *Can Rev Sociol*, *57*(4), 523-549. https://doi.org/10.1111/cars.12315

Shah, R., Raju, V. V., Sharma, A., & Grover, S. (2021). Impact of COVID-19 and Lockdown on Children with ADHD and Their Families-An Online Survey and a Continuity Care Model. *Journal of Neurosciences in Rural Practice*, *12*(1), 71-79. https://doi.org/10.1055/s-0040-1718645

Shahid, A., Javed, A., Rehman, S., Tariq, R., Ikram, M., & Suhail, M. (2020). Evaluation of psychological impact, depression, and anxiety among pregnant women during the COVID-19 pandemic in Lahore, Pakistan. *International Journal of Gynaecology and Obstetrics*, *151*(3), 462-465. https://doi.org/10.1002/ijgo.13398

Shaiba, L. A., Hadid, A., Altirkawi, K. A., Bakheet, H. M., Alherz, A. M., Hussain, S. A., . . . Alzamil, F. A. (2021). Case Report: Neonatal Multi-System Inflammatory Syndrome Associated With SARS-CoV-2 Exposure in Two Cases From Saudi Arabia. *Front Pediatr*, *9*, 652857. https://doi.org/10.3389/fped.2021.652857

Shangguan, F., Wang, R., Quan, X., Zhou, C., Zhang, C., Qian, W., . . . Zhang, X. Y. (2021). Association of Stress-Related Factors With Anxiety Among Chinese Pregnant Participants in an Online Crisis Intervention During COVID-19 Epidemic. *Frontiers in Psychology*, *12*, 633765. https://doi.org/10.3389/fpsyg.2021.633765

Shayganfard, M., Mahdavi, F., Haghighi, M., Sadeghi Bahmani, D., & Brand, S. (2020). Health Anxiety Predicts Postponing or Cancelling Routine Medical Health Care Appointments among Women in Perinatal Stage during the Covid-19 Lockdown. *International Journal of Environmental Research and Public Health*, *17*(21). https://doi.org/10.3390/ijerph17218272

Sheridan, D. C., Cloutier, R., Johnson, K., & Marshall, R. (2021). Where have all the emergency paediatric mental health patients gone during COVID-19? *Acta Paediatrica, International Journal of Paediatrics*, *110*(2), 598-599. https://doi.org/10.1111/apa.15537

Shinomiya, Y., Yoshizaki, A., Murata, E., Fujisawa, T. X., Taniike, M., & Mohri, I. (2021). Sleep and the General Behavior of Infants and Parents during the Closure of Schools as a Result of the COVID-19 Pandemic: Comparison with 2019 Data. *Children (Basel)*, *8*(2). https://doi.org/10.3390/children8020168

Shockley, K. M., Clark, M. A., Dodd, H., & King, E. B. (2020). Work-family strategies during COVID-19: Examining gender dynamics among dual-earner couples with young children. *Journal of Applied Psychology*. https://doi.org/10.1037/apl0000857

Shorey, S. Y., Ng, E. D., & Chee, C. Y. I. (2021). Anxiety and depressive symptoms of women in the perinatal period during the COVID-19 pandemic: A systematic review and meta-analysis. *Scand J Public Health*, 14034948211011793. https://doi.org/10.1177/14034948211011793

Shreffler, K. M., Joachims, C. N., Tiemeyer, S., Simmons, W. K., Teague, T. K., & Hays-Grudo, J. (2021). Childhood Adversity and Perceived Distress from the COVID-19 Pandemic. *Advers Resil Sci*, 1-4. https://doi.org/10.1007/s42844-021-00030-0

Shrestha, D., Saha, R., Manandhar, N., Adhikari, A., & Dahal, J. (2021). Anxiety among pregnant women about corona virus infections during covid-19 pandemic at a tertiary care center in nepal: A descriptive cross-sectional study. *Journal of the Nepal Medical Association*, *59*(234), 152-155. https://doi.org/10.31729/jnma.5377

Siegle, C. B. H., Pombo, A., Luz, C., Rodrigues, L. P., Cordovil, R., & dos Santos Cardoso de Sá, C. (2020). Influences of family and household characteristics on children's level of physical activity during social distancing due to covid-19 in Brazil. *Revista Paulista de Pediatria*, *39*. https://doi.org/10.1590/1984-0462/2021/39/2020297

Silverman, M. E., Burgos, L., Rodriguez, Z. I., Afzal, O., Kalishman, A., Callipari, F., . . . Loudon, H. (2020). Postpartum mood among universally screened high and low socioeconomic status patients during COVID-19 social restrictions in New York City. *Scientific Reports*, *10*(1), 22380. https://doi.org/10.1038/s41598-020-79564-9

Silverman, M. E., Medeiros, C., & Burgos, L. (2020). Early pregnancy mood before and during COVID-19 community restrictions among women of low socioeconomic status in New York City: a preliminary study. *Arch Womens Ment Health*, 1-4. https://doi.org/10.1007/s00737-020-01061-9

Silverstein, J. S., Limaye, M. A., Brubaker, S. G., Roman, A. S., Bautista, J., Chervenak, J., . . . Penfield, C. A. (2020). Acute Respiratory Decompensation Requiring Intubation in Pregnant Women with SARS-CoV-2 (COVID-19). *AJP Rep*, *10*(2), e169-e175. https://doi.org/10.1055/s-0040-1712925

Sinaci, S., Ozden Tokalioglu, E., Ocal, D., Atalay, A., Yilmaz, G., Keskin, H. L., . . . Moraloglu Tekin, O. (2020). Does having a high-risk pregnancy influence anxiety level during the COVID-19 pandemic? *European Journal of Obstetrics, Gynecology, and Reproductive Biology*, *255*, 190-196. https://doi.org/10.1016/j.ejogrb.2020.10.055

Smith, C. L., Waters, S. F., Spellacy, D., Burduli, E., Brooks, O., Carty, C. L., . . . Barbosa-Leiker, C. (2021). Substance use and mental health in pregnant women during the COVID-19 pandemic. *Journal of Reproductive and Infant Psychology*, 1-14. https://doi.org/10.1080/02646838.2021.1916815

Smith, V., Seo, D., Warty, R., Payne, O., Salih, M., Chin, K. L., . . . Wallace, E. (2020). Maternal and neonatal outcomes associated with COVID-19 infection: A systematic review. *PloS One*, *15*(6), e0234187. https://doi.org/10.1371/journal.pone.0234187

Sola, A., Rodríguez, S., Cardetti, M., & Dávila, C. (2020). [Perinatal COVID-19 in Latin America]. *Revista Panamericana de Salud Publica*, *44*, e47. https://doi.org/10.26633/rpsp.2020.47

Solís-García, G., Gutiérrez-Vélez, A., Pescador Chamorro, I., Zamora-Flores, E., Vigil-Vázquez, S., Rodríguez-Corrales, E., & Sánchez-Luna, M. (2021). [Epidemiology, management and risk of SARS-CoV-2 transmission in a cohort of newborns born to mothers diagnosed with COVID-19 infection]. *An Pediatr (Engl Ed)*, *94*(3), 173-178. https://doi.org/10.1016/j.anpedi.2020.12.004

Soumana, A., Samaila, A., Moustapha, L. M., Kamaye, M., Daouda, B., Salifou, I. A., . . . Ibrahim, M. L. (2020). A Fatal Case of COVID-19 in an Infant with Severe Acute Malnutrition Admitted to a Paediatric Ward in Niger. *Case Rep Pediatr*, *2020*, 8847415. https://doi.org/10.1155/2020/8847415

Spinelli, M., Lionetti, F., Pastore, M., & Fasolo, M. (2020). Parents' Stress and Children's Psychological Problems in Families Facing the COVID-19 Outbreak in Italy. *Frontiers in Psychology*, *11*, 1713. https://doi.org/10.3389/fpsyg.2020.01713

Spinelli, M., Lionetti, F., Setti, A., & Fasolo, M. (2020). Parenting Stress During the COVID-19 Outbreak: Socioeconomic and Environmental Risk Factors and Implications for Children Emotion Regulation. *Family Process*. https://doi.org/10.1111/famp.12601

Spinola, O., Liotti, M., Speranza, A. M., & Tambelli, R. (2020). Effects of COVID-19 Epidemic Lockdown on Postpartum Depressive Symptoms in a Sample of Italian Mothers. *Front Psychiatry*, *11*, 589916. https://doi.org/10.3389/fpsyt.2020.589916

Stallard, P., Pereira, A. I., & Barros, L. (2021). Post-traumatic growth during the COVID-19 pandemic in carers of children in Portugal and the UK: cross-sectional online survey. *BJPsych Open*, *7*(1), e37. https://doi.org/10.1192/bjo.2021.1

Stavridou, A., Stergiopoulou, A. A., Panagouli, E., Mesiris, G., Thirios, A., Mougiakos, T., . . . Tsitsika, A. (2020). Psychosocial consequences of COVID-19 in children, adolescents and young adults: A systematic review. *Psychiatry and Clinical Neurosciences*, *74*(11), 615-616. https://doi.org/10.1111/pcn.13134

Steinberg, S., Liu, T., & Lense, M. D. (2021). Musical Engagement and Parent-Child Attachment in Families With Young Children During the Covid-19 Pandemic. *Frontiers in Psychology*, *12*, 641733. https://doi.org/10.3389/fpsyg.2021.641733

Stepowicz, A., Wencka, B., Bieńkiewicz, J., Horzelski, W., & Grzesiak, M. (2020). Stress and Anxiety Levels in Pregnant and Post-Partum Women during the COVID-19 Pandemic. *International Journal of Environmental Research and Public Health*, *17*(24). https://doi.org/10.3390/ijerph17249450

Stojanov, J., Stankovic, M., Zikic, O., & Stojanov, A. (2020). The risk for nonpsychotic postpartum mood and anxiety disorders during the COVID-19 pandemic. *International Journal of Psychiatry in Medicine*, 91217420981533. https://doi.org/10.1177/0091217420981533

Suffren, S., Dubois-Comtois, K., Lemelin, J. P., St-Laurent, D., & Milot, T. (2021). Relations between Child and Parent Fears and Changes in Family Functioning Related to COVID-19. *International Journal of Environmental Research and Public Health*, *18*(4). https://doi.org/10.3390/ijerph18041786

Sun, F., Zhu, J., Tao, H., Ma, Y., & Jin, W. (2020). A systematic review involving 11,187 participants evaluating the impact of COVID-19 on anxiety and depression in pregnant women. *Journal of Psychosomatic Obstetrics and Gynecology*. https://doi.org/10.1080/0167482X.2020.1857360

Sun, G., Wang, Q., Lin, Y., Li, R., Yang, L., Liu, X., . . . Cheng, Y. (2020). Perinatal Depression of Exposed Maternal Women in the COVID-19 Pandemic in Wuhan, China. *Front Psychiatry*, *11*, 551812. https://doi.org/10.3389/fpsyt.2020.551812

Suzuki, S. (2020a). Psychological status during the first trimester of pregnancy under the COVID-19 epidemic in Japan. *Journal of Maternal-Fetal & Neonatal Medicine*, 1-2. https://doi.org/10.1080/14767058.2020.1793319

Suzuki, S. (2020b). Psychological status of postpartum women under the COVID-19 pandemic in Japan. *Journal of Maternal-Fetal & Neonatal Medicine*, 1-3. https://doi.org/10.1080/14767058.2020.1763949

Suárez-Rico, B. V., Estrada-Gutierrez, G., Sánchez-Martínez, M., Perichart-Perera, O., Rodríguez-Hernández, C., González-Leyva, C., . . . Reyes-Muñoz, E. (2021). Prevalence of Depression, Anxiety, and Perceived Stress in Postpartum Mexican Women during the COVID-19 Lockdown. *International Journal of Environmental Research and Public Health*, *18*(9). https://doi.org/10.3390/ijerph18094627

Sweet, L., Bradfield, Z., Vasilevski, V., Wynter, K., Hauck, Y., Kuliukas, L., . . . Wilson, A. N. (2021). Becoming a mother in the 'new' social world in Australia during the first wave of the COVID-19 pandemic. *Midwifery*, *98*, 102996. https://doi.org/10.1016/j.midw.2021.102996

Syed Anwar Aly, S. A., Abdul Rahman, R., Sharip, S., Shah, S. A., Abdullah Mahdy, Z., & Kalok, A. (2021). Pregnancy and COVID-19 Pandemic Perception in Malaysia: A Cross-Sectional Study. *International Journal of Environmental Research and Public Health*, *18*(11). https://doi.org/10.3390/ijerph18115762

Sánchez-Luna, M., Fernández Colomer, B., de Alba Romero, C., Alarcón Allen, A., Baña Souto, A., Camba Longueira, F., . . . Zamora Flores, E. (2021). Neonates Born to Mothers With COVID-19: Data From the Spanish Society of Neonatology Registry. *Pediatrics*, *147*(2). https://doi.org/10.1542/peds.2020-015065

Takaku, R., & Yokoyama, I. (2021). What the COVID-19 school closure left in its wake: Evidence from a regression discontinuity analysis in Japan. *J Public Econ*, *195*, 104364. https://doi.org/10.1016/j.jpubeco.2020.104364

Talbot, J., Charron, V., & Konkle, A. T. (2021). Feeling the Void: Lack of Support for Isolation and Sleep Difficulties in Pregnant Women during the COVID-19 Pandemic Revealed by Twitter Data Analysis. *International Journal of Environmental Research and Public Health*, *18*(2). https://doi.org/10.3390/ijerph18020393

Tambling, R. R., Tomkunas, A. J., Russell, B. S., Horton, A. L., & Hutchison, M. (2021). Thematic Analysis of Parent-Child Conversations About COVID-19: "Playing It Safe". *J Child Fam Stud*, 1-13. https://doi.org/10.1007/s10826-020-01889-w

Tang, S., Xiang, M., Cheung, T., & Xiang, Y. T. (2020). Mental health and its correlates among children and adolescents during COVID-19 school closure: The importance of parent-child discussion. *Journal of Affective Disorders*, *279*, 353-360. https://doi.org/10.1016/j.jad.2020.10.016

Taubman-Ben-Ari, O., & Ben-Yaakov, O. (2020). Distress and apprehension among new parents during the COVID-19 pandemic: The contribution of personal resources. *American Journal of Orthopsychiatry*, *90*(6), 810-816. https://doi.org/10.1037/ort0000497

Taubman-Ben-Ari, O., Ben-Yaakov, O., & Chasson, M. (2021). Parenting stress among new parents before and during the COVID-19 pandemic. *Child Abuse and Neglect*, *117*, 105080. https://doi.org/10.1016/j.chiabu.2021.105080

Taubman-Ben-Ari, O., Chasson, M., Abu Sharkia, S., & Weiss, E. (2020). Distress and anxiety associated with COVID-19 among Jewish and Arab pregnant women in Israel. *Journal of Reproductive and Infant Psychology*, *38*(3), 340-348. https://doi.org/10.1080/02646838.2020.1786037

Taubman-Ben-Ari, O., Chasson, M., & Abu-Sharkia, S. (2020). Childbirth anxieties in the shadow of COVID-19: Self-compassion and social support among Jewish and Arab pregnant women in Israel. *Health Soc Care Community*. https://doi.org/10.1111/hsc.13196

Tchimtchoua Tamo, A. R. (2020). An analysis of mother stress before and during COVID-19 pandemic: The case of China. *Health Care for Women International*, *41*(11-12), 1349-1362. https://doi.org/10.1080/07399332.2020.1841194

Teles Abrao Trad, A., Ibirogba, E. R., Elrefaei, A., Narang, K., Tonni, G., Picone, O., . . . Ruano, R. (2020). Complications and outcomes of SARS-CoV-2 in pregnancy: where and what is the evidence? *Hypertension in Pregnancy*, *39*(3), 361-369. https://doi.org/10.1080/10641955.2020.1769645

TG, W. M., Kassie, B. A., Asratie, M. H., & Abate, A. T. (2021). The Effects of Fear and Knowledge of COVID-19 on Preventive Practice Among Pregnant Women Who Attend Antenatal Care in Northwest Ethiopia, 2020: Institution-Based Cross-Sectional Study. *Int J Womens Health*, *13*, 95-100. https://doi.org/10.2147/ijwh.S286088

Thayer, Z. M., & Gildner, T. E. (2020). COVID-19-related financial stress associated with higher likelihood of depression among pregnant women living in the United States. *American Journal of Human Biology*, e23508. https://doi.org/10.1002/ajhb.23508

Thompson, K. A., & Bardone-Cone, A. M. (2021). 2019-nCOV distress and depressive, anxiety and OCD-type, and eating disorder symptoms among postpartum and control women. *Arch Womens Ment Health*, 1-10. https://doi.org/10.1007/s00737-021-01120-9

Tomfohr-Madsen, L. M., Racine, N., Giesbrecht, G. F., Lebel, C., & Madigan, S. (2021). Depression and anxiety in pregnancy during COVID-19: A rapid review and meta-analysis. *Psychiatry Research*, *300*, 113912. https://doi.org/10.1016/j.psychres.2021.113912

Tso, W. W. Y., Wong, R. S., Tung, K. T. S., Rao, N., Fu, K. W., Yam, J. C. S., . . . Lp, P. (2020). Vulnerability and resilience in children during the COVID-19 pandemic. *European Child and Adolescent Psychiatry*, 1-16. https://doi.org/10.1007/s00787-020-01680-8

Ueda, R., Okada, T., Kita, Y., Ozawa, Y., Inoue, H., Shioda, M., . . . Ozawa, H. (2021). The quality of life of children with neurodevelopmental disorders and their parents during the Coronavirus disease 19 emergency in Japan. *Scientific Reports*, *11*(1), 3042. https://doi.org/10.1038/s41598-021-82743-x

Upendra, S., Devi, S., Kaur, J., Waghmare, S., & Barde, S. (2020). A phenomenological study of pregnant women’s experience: COVID-19 lockdown period. *Indian Journal of Forensic Medicine and Toxicology*, *14*(4), 3939-3942. https://doi.org/10.37506/ijfmt.v14i4.12253

V, C. F., & Iarocci, G. (2020). Child and Family Outcomes Following Pandemics: A Systematic Review and Recommendations on COVID-19 Policies. *Journal of Pediatric Psychology*, *45*(10), 1124-1143. https://doi.org/10.1093/jpepsy/jsaa092

Valero-Moreno, S., Lacomba-Trejo, L., Tamarit, A., Pérez-Marín, M., & Montoya-Castilla, I. (2021). Psycho-emotional adjustment in parents of adolescents: A cross-sectional and longitudinal analysis of the impact of the COVID pandemic. *Journal of Pediatric Nursing*. https://doi.org/10.1016/j.pedn.2021.01.028

Vasilevski, V., Sweet, L., Bradfield, Z., Wilson, A. N., Hauck, Y., Kuliukas, L., . . . Wynter, K. (2021). Receiving maternity care during the COVID-19 pandemic: Experiences of women's partners and support persons. *Women Birth*. https://doi.org/10.1016/j.wombi.2021.04.012

Vaterlaus, J. M., Shaffer, T., Patten, E. V., & Spruance, L. A. (2021). Parent-Child Relationships and the COVID-19 Pandemic: An Exploratory Qualitative Study with Parents in Early, Middle, and Late Adulthood. *J Adult Dev*, 1-13. https://doi.org/10.1007/s10804-021-09381-5

Vazquez-Vazquez, A., Dib, S., Rougeaux, E., Wells, J. C., & Fewtrell, M. S. (2021). The impact of the Covid-19 lockdown on the experiences and feeding practices of new mothers in the UK: Preliminary data from the COVID-19 New Mum Study. *Appetite*, *156*, 104985. https://doi.org/10.1016/j.appet.2020.104985

Vigod, S. N., Brown, H. K., Huang, A., Fung, K., Barker, L. C., Hussain-Shamsy, N., . . . Moineddin, R. (2021). Postpartum mental illness during the COVID-19 pandemic: a population-based, repeated cross-sectional study. *CMAJ: Canadian Medical Association Journal*, *193*(23), E835-e843. https://doi.org/10.1503/cmaj.210151

Vu Hoang, D., Cashin, J., Gribble, K., Marinelli, K., & Mathisen, R. (2020). Misalignment of global COVID-19 breastfeeding and newborn care guidelines with World Health Organization recommendations. *BMJ Nutr Prev Health*, *3*(2), 339-350. https://doi.org/10.1136/bmjnph-2020-000184

Walker, K. F., O'Donoghue, K., Grace, N., Dorling, J., Comeau, J. L., Li, W., & Thornton, J. G. (2020). Maternal transmission of SARS-COV-2 to the neonate, and possible routes for such transmission: a systematic review and critical analysis. *BJOG: An International Journal of Obstetrics and Gynaecology*, *127*(11), 1324-1336. https://doi.org/10.1111/1471-0528.16362

Waller, R., Powell, T., Rodriguez, Y., Corbett, N., Perlstein, S., White, L. K., . . . Wagner, N. J. (2021). The Impact of the COVID-19 Pandemic on Children's Conduct Problems and Callous-Unemotional Traits. *Child Psychiatry and Human Development*, 1-12. https://doi.org/10.1007/s10578-020-01109-y

Wang, J., Li, Y., Musch, D. C., Wei, N., Qi, X., Ding, G., . . . Qian, X. (2021). Progression of Myopia in School-Aged Children After COVID-19 Home Confinement. *JAMA Ophthalmol*, *139*(3), 293-300. https://doi.org/10.1001/jamaophthalmol.2020.6239

Wang, J., Zhou, Y., Qian, W., Han, R., & Liu, Z. (2021). Maternal insomnia during the COVID-19 pandemic: associations with depression and anxiety. *Social Psychiatry and Psychiatric Epidemiology*, 1-9. https://doi.org/10.1007/s00127-021-02072-2

Wang, Q., Mo, P. K. H., Song, B., Di, J. L., Zhou, F. R., Zhao, J., . . . Wang, L. H. (2021). Mental health and preventive behaviour of pregnant women in China during the early phase of the COVID-19 period. *Infect Dis Poverty*, *10*(1), 37. https://doi.org/10.1186/s40249-021-00825-4

Wang, Q., Song, B., Di, J., Yang, X., Wu, A., Lau, J., . . . Mo, P. K. (2021). Intentions to Seek Mental Health Services During the COVID-19 Pandemic Among Chinese Pregnant Women With Probable Depression or Anxiety: Cross-sectional, Web-Based Survey Study. *JMIR Ment Health*, *8*(2), e24162. https://doi.org/10.2196/24162

Wang, S. D., Devjani, S., Chillakanti, M., Dunton, G. F., & Mason, T. B. (2021). The COMET study: Examining the effects of COVID-19-related perceived stress on Los Angeles Mothers' dysregulated eating behaviors, child feeding practices, and body mass index. *Appetite*, *163*, 105209. https://doi.org/10.1016/j.appet.2021.105209

Wang, Y., Chen, L., Wu, T., Shi, H., Li, Q., Jiang, H., . . . Qiao, J. (2020). Impact of Covid-19 in pregnancy on mother's psychological status and infant's neurobehavioral development: a longitudinal cohort study in China. *BMC Medicine*, *18*(1), 347. https://doi.org/10.1186/s12916-020-01825-1

Wdowiak, A., Makara-Studzińska, M., Raczkiewicz, D., Janczyk, P., Słabuszewska-Jóźwiak, A., Wdowiak-Filip, A., & Studzińska, N. (2021). Effect of Excessive Body Weight and Emotional Disorders on the Course of Pregnancy and Well-Being of a Newborn before and during COVID-19 Pandemic. *J Clin Med*, *10*(4). https://doi.org/10.3390/jcm10040656

Wheeler, J. M., Misra, D. P., & Giurgescu, C. (2021). Stress and coping among pregnant black women during the COVID-19 pandemic. *Public Health Nursing*. https://doi.org/10.1111/phn.12909

Wilder, J. L., Hark, C. M., Marcus, C. H., Rabinowitz, E. C., Michelson, C. D., Winn, A. S., & Pingree, E. W. (2021). Pediatric Trainees as Parents: Perspectives From a Pandemic. *Academic Pediatrics*. https://doi.org/10.1016/j.acap.2021.04.006

Wilke, N. G., Howard, A. H., & Goldman, P. (2020). Rapid return of children in residential care to family as a result of COVID-19: Scope, challenges, and recommendations. *Child Abuse and Neglect*, *110*. https://doi.org/10.1016/j.chiabu.2020.104712

Wimberly, C. E., Towry, L., Caudill, C., Johnston, E. E., & Walsh, K. M. (2021). Impacts of COVID-19 on caregivers of childhood cancer survivors. *Pediatric Blood & Cancer*, *68*(4), e28943. https://doi.org/10.1002/pbc.28943

Wolf, J. P., Freisthler, B., & Chadwick, C. (2021). Stress, alcohol use, and punitive parenting during the COVID-19 pandemic. *Child Abuse and Neglect*, *117*, 105090. https://doi.org/10.1016/j.chiabu.2021.105090

Wong, J. Y., Wai, A. K., Wang, M. P., Lee, J. J., Li, M., Kwok, J. Y., . . . Choi, A. W. (2021). Impact of COVID-19 on Child Maltreatment: Income Instability and Parenting Issues. *International Journal of Environmental Research and Public Health*, *18*(4). https://doi.org/10.3390/ijerph18041501

World Bank (2021). World Bank Country and Lending Groups. Website: <https://datahelpdesk.worldbank.org/knowledgebase/articles/906519-world-bank-country-and-lending-groups>

World Health Organization. (2021). COVID-19 and breastfeeding - Position paper, 8 April 2020. Website: <https://www.euro.who.int/en/health-topics/health-emergencies/coronavirus-covid-19/publications-and-technical-guidance/2020/covid-19-and-breastfeeding-position-paper,-8-april-2020-produced-by-whoeurope>

World Health Organization (2021). Global Research on COVID-19 database. Website: <https://www.who.int/emergencies/diseases/novel-coronavirus-2019/global-research-on-novel-coronavirus-2019-ncov>

World Health Organization (2021). New research highlights risks of separating newborns from mothers during COVID-19 pandemic. Website: <https://www.who.int/news/item/16-03-2021-new-research-highlights-risks-of-separating-newborns-from-mothers-during-covid-19-pandemic>

Wu, F., Lin, W., Liu, P., Zhang, M., Huang, S., Chen, C., . . . Chen, Q. (2021). Prevalence and contributory factors of anxiety and depression among pregnant women in the post-pandemic era of COVID-19 in Shenzhen, China. *Journal of Affective Disorders*, *291*, 243-251. https://doi.org/10.1016/j.jad.2021.05.014

Wu, Q., Xu, Y., & Jedwab, M. (2021). Custodial Grandparent's Job Loss During the COVID-19 Pandemic and Its Relationship With Parenting Stress and Mental Health. *Journal of Applied Gerontology*, 7334648211006222. https://doi.org/10.1177/07334648211006222

Wu, Y., Zhang, C., Liu, H., Duan, C., Li, C., Fan, J., . . . Huang, H. F. (2020). Perinatal depressive and anxiety symptoms of pregnant women during the coronavirus disease 2019 outbreak in China. *American Journal of Obstetrics and Gynecology*, *223*(2), 240.e241-240.e249. https://doi.org/10.1016/j.ajog.2020.05.009

Xie, M., Wang, X., Zhang, J., & Wang, Y. (2021). Alteration in the psychologic status and family environment of pregnant women before and during the COVID-19 pandemic. *International Journal of Gynaecology and Obstetrics*, *153*(1), 71-75. https://doi.org/10.1002/ijgo.13575

Xu, K., Zhang, Y., Xu, Q., Lv, L., & Zhang, J. (2021). Mental health among pregnant women under public health interventions during COVID-19 outbreak in Wuhan, China. *Psychiatry Research*, *301*, 113977. https://doi.org/10.1016/j.psychres.2021.113977

Xu, Y., Wu, Q., Jedwab, M., & Levkoff, S. E. (2020). Understanding the Relationships between Parenting Stress and Mental Health with Grandparent Kinship Caregivers' Risky Parenting Behaviors in the Time of COVID-19. *J Fam Violence*, 1-13. https://doi.org/10.1007/s10896-020-00228-3

Xu, Y., Wu, Q., Levkoff, S. E., & Jedwab, M. (2020). Material hardship and parenting stress among grandparent kinship providers during the COVID-19 pandemic: The mediating role of grandparents' mental health. *Child Abuse and Neglect*, 104700. https://doi.org/10.1016/j.chiabu.2020.104700

Xue, A., Oros, V., Marca-Ghaemmaghami, P., Scholkmann, F., Righini-Grunder, F., Natalucci, G., . . . Restin, T. (2021). New Parents Experienced Lower Parenting Self-Efficacy during the COVID-19 Pandemic Lockdown. *Children (Basel)*, *8*(2). https://doi.org/10.3390/children8020079

Xue, B., & McMunn, A. (2021). Gender differences in unpaid care work and psychological distress in the UK Covid-19 lockdown. *PloS One*, *16*(3), e0247959. https://doi.org/10.1371/journal.pone.0247959

Yamamura, E., & Tsustsui, Y. (2021). The impact of closing schools on working from home during the COVID-19 pandemic: evidence using panel data from Japan. *Rev Econ Househ*, 1-20. https://doi.org/10.1007/s11150-020-09536-5

Yaman, A., Kandemir, I., & Varkal, M. A. (2021). Infants infected with SARS-CoV-2 and newborns born to mother diagnosed with COVID-19: clinical experience. *Irish Journal of Medical Science*, 1-6. https://doi.org/10.1007/s11845-021-02662-8

Yan, H., Ding, Y., & Guo, W. (2020). Mental Health of Pregnant and Postpartum Women During the Coronavirus Disease 2019 Pandemic: A Systematic Review and Meta-Analysis. *Frontiers in Psychology*, *11*, 617001. https://doi.org/10.3389/fpsyg.2020.617001

Yan, K., Xiao, F. F., Jiang, Y. W., Xiao, T. T., Zhang, D. J., Yuan, W. H., . . . Zeng, L. K. (2021). Effects of SARS-CoV-2 infection on neuroimaging and neurobehavior in neonates. *World Journal of Pediatrics*, *17*(2), 171-179. https://doi.org/10.1007/s12519-021-00423-2

Yang, H., Hu, B., Zhan, S., Yang, L. Y., & Xiong, G. (2020). Effects of Severe Acute Respiratory Syndrome Coronavirus 2 Infection on Pregnant Women and Their Infants. *Archives of Pathology and Laboratory Medicine*, *144*(10), 1217-1222. https://doi.org/10.5858/arpa.2020-0232-SA

Yang, P., Wang, X., Liu, P., Wei, C., He, B., Zheng, J., & Zhao, D. (2020). Clinical characteristics and risk assessment of newborns born to mothers with COVID-19. *Journal of Clinical Virology*, *127*, 104356. https://doi.org/10.1016/j.jcv.2020.104356

Yang, R., Mei, H., Zheng, T., Fu, Q., Zhang, Y., Buka, S., . . . Zhou, A. (2020). Pregnant women with COVID-19 and risk of adverse birth outcomes and maternal-fetal vertical transmission: a population-based cohort study in Wuhan, China. *BMC Medicine*, *18*(1), 330. https://doi.org/10.1186/s12916-020-01798-1

Yang, X., Song, B., Wu, A., Mo, P. K. H., Di, J., Wang, Q., . . . Wang, L. (2021). Social, Cognitive, and eHealth Mechanisms of COVID-19-Related Lockdown and Mandatory Quarantine That Potentially Affect the Mental Health of Pregnant Women in China: Cross-Sectional Survey Study. *Journal of Medical Internet Research*, *23*(1), e24495. https://doi.org/10.2196/24495

Yassa, M., Yassa, A., Yirmibeş, C., Birol, P., Ünlü, U. G., Tekin, A. B., . . . Tug, N. (2020). Anxiety levels and obsessive compulsion symptoms of pregnant women during the COVID-19 pandemic. *Turk J Obstet Gynecol*, *17*(3), 155-160. https://doi.org/10.4274/tjod.galenos.2020.91455

Yavaş Çelik, M. (2021). The obligation of parents with COVID-19 positivity to stay separated from their children. *Journal of Child and Adolescent Psychiatric Nursing*, *34*(2), 105-111. https://doi.org/10.1111/jcap.12303

Yerkes, M. A., André, S. C. H., Besamusca, J. W., Kruyen, P. M., Remery, C., van der Zwan, R., . . . Geurts, S. A. E. (2020). 'Intelligent' lockdown, intelligent effects? Results from a survey on gender (in)equality in paid work, the division of childcare and household work, and quality of life among parents in the Netherlands during the Covid-19 lockdown. *PloS One*, *15*(11), e0242249. https://doi.org/10.1371/journal.pone.0242249

Yildirim, T. M., & Eslen-Ziya, H. (2020). The Differential Impact of COVID-19 on the Work Conditions of Women and Men Academics during the Lockdown. *Gend Work Organ*. https://doi.org/10.1111/gwao.12529

Yirmiya, K., Yakirevich-Amir, N., Preis, H., Lotan, A., Atzil, S., & Reuveni, I. (2021). Women's Depressive Symptoms during the COVID-19 Pandemic: The Role of Pregnancy. *International Journal of Environmental Research and Public Health*, *18*(8). https://doi.org/10.3390/ijerph18084298

Yoon, S. H., Kang, J. M., & Ahn, J. G. (2020). Clinical outcomes of 201 neonates born to mothers with COVID-19: a systematic review. *European Review for Medical and Pharmacological Sciences*, *24*(14), 7804-7815. https://doi.org/10.26355/eurrev_202007_22285

Yu, N., Li, W., Kang, Q., Xiong, Z., Wang, S., Lin, X., . . . Wu, J. (2020). Clinical features and obstetric and neonatal outcomes of pregnant patients with COVID-19 in Wuhan, China: a retrospective, single-centre, descriptive study. *Lancet Infectious Diseases*, *20*(5), 559-564. https://doi.org/10.1016/s1473-3099(20)30176-6

Yuan, R., Xu, Q. H., Xia, C. C., Lou, C. Y., Xie, Z., Ge, Q. M., & Shao, Y. (2020). Psychological status of parents of hospitalized children during the COVID-19 epidemic in China. *Psychiatry Research*, *288*, 112953. https://doi.org/10.1016/j.psychres.2020.112953

Yue, C., Liu, C., Wang, J., Zhang, M., Wu, H., Li, C., & Yang, X. (2020). Association between social support and anxiety among pregnant women in the third trimester during the coronavirus disease 2019 (COVID-19) epidemic in Qingdao, China: The mediating effect of risk perception. *International Journal of Social Psychiatry*, 20764020941567. https://doi.org/10.1177/0020764020941567

Yue, J., Zang, X., Le, Y., & An, Y. (2020). Anxiety, depression and PTSD among children and their parent during 2019 novel coronavirus disease (COVID-19) outbreak in China. *Current Psychology (New Brunswick, N.J.)*, 1-8. https://doi.org/10.1007/s12144-020-01191-4

Zaigham, M., & Andersson, O. (2020). Maternal and perinatal outcomes with COVID-19: A systematic review of 108 pregnancies. *Acta Obstetricia et Gynecologica Scandinavica*, *99*(7), 823-829. https://doi.org/10.1111/aogs.13867

Zamarro, G., & Prados, M. J. (2021). Gender differences in couples' division of childcare, work and mental health during COVID-19. *Rev Econ Househ*, 1-30. https://doi.org/10.1007/s11150-020-09534-7

Zanardo, V., Manghina, V., Giliberti, L., Vettore, M., Severino, L., & Straface, G. (2020). Psychological impact of COVID-19 quarantine measures in northeastern Italy on mothers in the immediate postpartum period. *International Journal of Gynaecology and Obstetrics*, *150*(2), 184-188. https://doi.org/10.1002/ijgo.13249

Zanardo, V., Tortora, D., Guerrini, P., Garani, G., Severino, L., Soldera, G., & Straface, G. (2020). Infant feeding initiation practices in the context of COVID-19 lockdown. *Early Human Development*, *152*, 105286. https://doi.org/10.1016/j.earlhumdev.2020.105286

Zeng, L., Xia, S., Yuan, W., Yan, K., Xiao, F., Shao, J., & Zhou, W. (2020). Neonatal Early-Onset Infection With SARS-CoV-2 in 33 Neonates Born to Mothers With COVID-19 in Wuhan, China. *JAMA Pediatr*, *174*(7), 722-725. https://doi.org/10.1001/jamapediatrics.2020.0878

Zeng, X., Li, W., Sun, H., Luo, X., Garg, S., Liu, T., . . . Zhang, Y. (2020). Mental Health Outcomes in Perinatal Women During the Remission Phase of COVID-19 in China. *Front Psychiatry*, *11*, 571876. https://doi.org/10.3389/fpsyt.2020.571876

Zhang, C. J. P., Wu, H., He, Z., Chan, N. K., Huang, J., Wang, H., . . . Ming, W. K. (2021). Psychobehavioral Responses, Post-Traumatic Stress and Depression in Pregnancy During the Early Phase of COVID-19 Outbreak. *Psychiatric Research and Clinical Practice*, *3*(1), 46-54. https://doi.org/10.1176/appi.prcp.20200019

Zhang, Y., Deng, R., Chen, M., Cao, R., Chen, S., Chen, K., . . . Tian, K. (2021). Association of Sleep Duration and Screen Time With Anxiety of Pregnant Women During the COVID-19 Pandemic. *Frontiers in Psychology*, *12*, 646368. https://doi.org/10.3389/fpsyg.2021.646368

Zhang, Y., & Ma, Z. F. (2020). Psychological responses and lifestyle changes among pregnant women with respect to the early stages of COVID-19 pandemic. *International Journal of Social Psychiatry*, 20764020952116. https://doi.org/10.1177/0020764020952116

Zheng, Q. X., Jiang, X. M., Lin, Y., Liu, G. H., Lin, Y. P., Kang, Y. L., & Liu, X. W. (2020). The influence of psychological response and security sense on pregnancy stress during the outbreak of coronavirus disease 2019: A mediating model. *Journal of Clinical Nursing*, *29*(21-22), 4248-4257. https://doi.org/10.1111/jocn.15460

Zhou, Y., Shi, H., Liu, Z., Peng, S., Wang, R., Qi, L., . . . Zhang, X. (2020). The prevalence of psychiatric symptoms of pregnant and non-pregnant women during the COVID-19 epidemic. *Transl Psychiatry*, *10*(1), 319. https://doi.org/10.1038/s41398-020-01006-x

Zhou, Y., Wang, R., Liu, L., Ding, T., Huo, L., Qi, L., . . . Dai, G. (2021). The impact of lockdown policy on depressive symptoms among pregnant women in China: mediating effects of internet use and family support. *Glob Health Res Policy*, *6*(1), 11. https://doi.org/10.1186/s41256-021-00193-4

Zhu, H., Wang, L., Fang, C., Peng, S., Zhang, L., Chang, G., . . . Zhou, W. (2020). Clinical analysis of 10 neonates born to mothers with 2019-nCoV pneumonia. *Transl Pediatr*, *9*(1), 51-60. https://doi.org/10.21037/tp.2020.02.06

Zilver, S. J. M., Broekman, B. F. P., Hendrix, Y., de Leeuw, R. A., Mentzel, S. V., van Pampus, M. G., & de Groot, C. J. M. (2021). Stress, anxiety and depression in 1466 pregnant women during and before the COVID-19 pandemic: a Dutch cohort study. *Journal of Psychosomatic Obstetrics and Gynaecology*, *42*(2), 108-114. https://doi.org/10.1080/0167482x.2021.1907338

Zreik, G., Asraf, K., Haimov, I., & Tikotzky, L. (2020). Maternal perceptions of sleep problems among children and mothers during the coronavirus disease 2019 (COVID-19) pandemic in Israel. *Journal of Sleep Research*, e13201. https://doi.org/10.1111/jsr.13201

Çakmak, G., & Öztürk, Z. A. (2021). Being Both a Parent and a Healthcare Worker in the Pandemic: Who Could Be Exhausted More? *Healthcare (Basel)*, *9*(5). https://doi.org/10.3390/healthcare9050564

Çolak, S., Gürlek, B., Önal, Ö., Yılmaz, B., & Hocaoglu, C. (2021). The level of depression, anxiety, and sleep quality in pregnancy during coronavirus disease 2019 pandemic. *Journal of Obstetrics and Gynaecology Research*. https://doi.org/10.1111/jog.14872

Özkan Şat, S., & Yaman Sözbir, Ş. (2021). Use of Mobile Applications by Pregnant Women and Levels of Pregnancy Distress During the COVID-19 (Coronavirus) Pandemic. *Matern Child Health J*, *25*(7), 1057-1068. https://doi.org/10.1007/s10995-021-03162-y
